# Synthesis and Antimicrobial Evaluation of Chroman-4-One and Homoisoflavonoid Derivatives

**DOI:** 10.3390/molecules30173575

**Published:** 2025-08-31

**Authors:** Carlos d. S. M. Bezerra Filho, José L. F. M. Galvão, Edeltrudes O. Lima, Yunierkis Perez-Castillo, Yendrek Velásquez-López, Damião P. de Sousa

**Affiliations:** 1 Department of Pharmaceutical Sciences, Federal University of Paraíba, João Pessoa 58051-900, PB, Brazil; carlosmaia1996@gmail.com (C.d.S.M.B.F.); luksfmgalvao@hotmail.com (J.L.F.M.G.); edeolima@yahoo.com.br (E.O.L.); 2 Bio-Cheminformatics Research Group and Facultad de Ingeniería y Ciencias Aplicadas, Universidad de Las Américas, Quito 170125, Ecuador; yunierkis@gmail.com (Y.P.-C.); yendrek.velasquez.lopez@udla.edu.ec (Y.V.-L.)

**Keywords:** natural products, medicinal plants, chromanone, flavonoid, in silico, biologic activity, antibacterial, antifungal, *Candida*

## Abstract

The continuous increase in microbial resistance to therapeutic agents has become one of the greatest challenges to global health. In this context, the present study investigated the bioactivity of 25 chroman-4-one and homoisoflavonoid derivatives—17 of which are novel—against pathogenic microorganisms, including *Staphylococcus epidermidis*, *Pseudomonas aeruginosa*, *Salmonella enteritidis*, *Candida albicans*, *C. tropicalis*, *Nakaseomyces glabratus* (formerly *C. glabrata)*, *Aspergillus flavus*, and *Penicillium citrinum*. Antimicrobial assay was performed using the microdilution technique in 96-well microplates to determine the minimum inhibitory concentration (MIC). Thirteen compounds exhibited antimicrobial activity, with compounds **1**, **2**, and **21** demonstrating greater potency than the positive control, especially against *Candida* species. Molecular modeling suggested distinct mechanisms of action in *Candida albicans*: **1** potentially inhibits cysteine synthase, while **2** and **21** possibly target HOG1 kinase and FBA1, key proteins in fungal virulence and survival. Our findings indicated that the addition of alkyl or aryl carbon chains at the hydroxyl group at position 7 reduces antimicrobial activity, whereas the presence of methoxy substituents at the *meta* position of ring B in homoisoflavonoids enhances bioactivity. These findings highlight key structural features of these compound classes, which may aid in the development of new bioactive agents against pathogenic microorganisms.

## 1. Introduction

In recent decades, there has been a steady increase in microbial resistance to conventional therapeutic agents, a problem that has become one of the greatest challenges to global health [1,2,3]. One of the main factors driving the emergence of resistant microorganisms is the excessive and inappropriate use of antimicrobials, which compromises the effectiveness of available treatments [4,5]. Additionally, infections caused by resistant microorganisms are associated with higher mortality and morbidity rates, prolonged hospital stays, and increased healthcare costs. It is estimated that antimicrobial-resistant infections are responsible for 700,000 deaths annually worldwide, and studies suggest this number could rise to 10 million deaths per year by 2050. As a result, the search for new compounds with antimicrobial activity has become increasingly critical [6,7].

In this context, natural products and synthetic derivatives emerge as a promising source for the discovery of new bioactive compounds, since most of the drugs available in therapy are natural products or analogs [8]. Among the various classes of natural products, homoisoflavonoids and chroman-4-ones have gained significant attention due to their structural diversity and broad range of biological activities [9,10,11,12,13].

Chroman-4-ones (2,3-dihydro-1-benzopyran-4-one) are heterocyclic compounds characterized by a benzene ring fused to a 2,3-dihydro-*γ*-pyranone system [14]. These compounds differ from chromones due to the absence of a C2–C3 double bond. In organic chemistry, chromanones have been widely used as intermediates in the synthesis of a diverse array of bioactive molecules, including homoisoflavonoids [11,15]. Additionally, chroman-4-ones exhibit a variety of biological activities, including anticancer [16,17], antioxidant [18], and anti-inflammatory effects [19], as well as broad-spectrum antimicrobial properties [20,21,22].

Homoisoflavonoids are a unique subclass of flavonoids characterized by an additional carbon atom in their carbon skeleton. More than 290 naturally occurring homoisoflavonoids have been isolated from plants, including those from the Fabaceae, Asparagaceae, Orchidaceae, Polygonaceae, Portulacaceae, and Gentianaceae families [23,24]. Regarding their biological properties, the literature reports indicate that homoisoflavonoids possess antidiabetic [25]; cytotoxic and antiangiogenic [26]; anti-inflammatory [27]; and antimutagenic activities [28]. In addition, studies have demonstrated their antimicrobial potential against fungi, as well as Gram-negative and Gram-positive bacteria [29,30,31,32,33].

**Figure 1:**
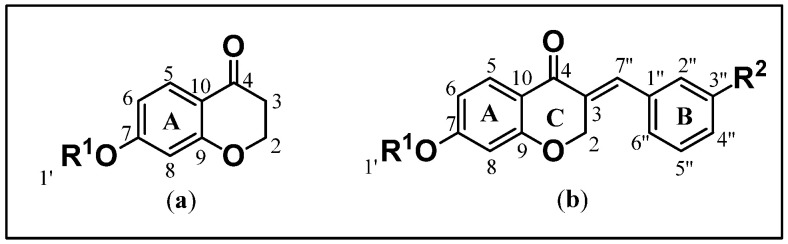
Basic chemical structure of the derivatives synthesized: (**a**) chroman-4-one derivatives; (**b**) homoisoflavonoid derivatives.

Considering the bioactive potential of these classes of natural products, the present study aimed to design and synthesize a series of structurally related chroman-4-one and homoisoflavonoid derivatives (Figure 1) for the evaluation of their antimicrobial activity.

## 2. Results

### 2.1. Synthesis of Compounds (***1***–***25***)

Compound **1** (7-Hydroxychroman-4-one) was prepared as a precursor for the synthesis of the other compounds in this study. To achieve this, resorcinol and a carboxylic acid (3-bromopropionic acid) were used in the presence of a Lewis acid (trifluoromethanesulfonic acid); through Friedel–Crafts acylation, 3-bromo-1-(2,4-dihydroxyphenyl)propan-1-one was obtained. This product was stirred in 2 M NaOH to promote an intramolecular cyclization via bimolecular nucleophilic substitution, resulting in compound **1** without requiring rigorous chromatography [34]. The formation of chroman-4-one was confirmed by ^1^H Nuclear Magnetic Resonance Spectroscopy (NMR), with the phenolic hydroxyl hydrogen appearing as a singlet at δ_H_ 10.54 ppm (*s*, 7-OH; 1H), three aromatic hydrogens in the range of δ_H_ 7.61–6.30 ppm (H-5; H-6 and H-8), and signals at δ_H_ 4.45 (*t*, *J* = 6.1 Hz; 2H; H-2) and 2.66 (*t*, *J* = 6.7 Hz; 2H; H-3) ppm, indicating the absence of the C2–C3 double bond. These findings were corroborated by ^13^C NMR-APT spectroscopy, which showed signals at δ_C_ 189.8 (C=O), 164.4 (C-7), 128.5 (C-5), 110.4 (C-6), 102.4 (C-8), 66.9 (C-2), and 36.9 (C-3).

**Scheme 1:**
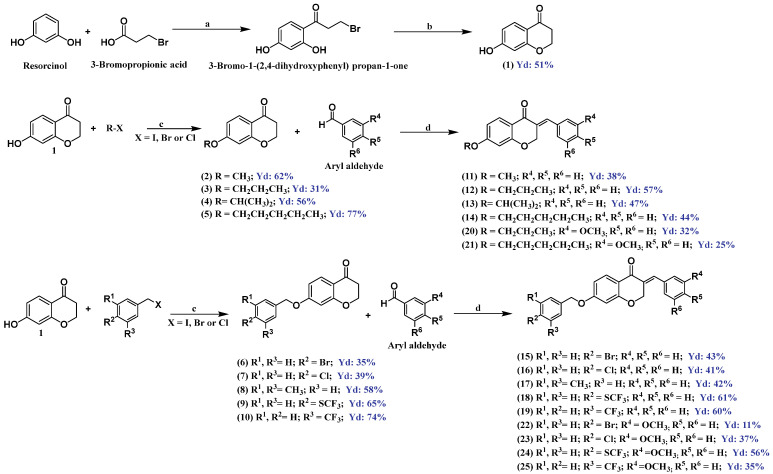
Synthesis of compounds **1**–**25**. a: trifluoromethanesulfonic acid, 80 °C; b: NaOH 2 M, 5 –r.t; c: K_2_CO_3_, DMF, room temperature (r.t); d: pyrrolidine, methanol/dicloromethane (1:1), r.t; aryl aldehyde: benzaldehyde or 3-methoxybenzaldehyde. Yd: Yield.

The chroman-4-one derivatives (**2**–**10**) were prepared through bimolecular nucleophilic substitution reactions at the phenolic hydroxyl group (position 7) of compound **1** [34] (Scheme 1). To obtain the *O*-alkylated derivatives, various alkyl and aryl halides were used, with reaction yields ranging from 31% to 77%. The ^1^H and ^13^C NMR-APT spectra of compound **2** differed from **1** due to the absence of the phenolic hydroxyl signal at δ_H_ 10.54 ppm and the presence of a methoxy group at δ_H_ 3.80 ppm (*s*, 3H) and δ_C_ 56.0 ppm.

Compounds **3**–**5** exhibited hydrogens attached to *sp*^3^ carbons in the range of δ_H_ 2.74–0.93 ppm and *sp*^3^ carbons signals at δ_C_ 56.0–10.3 ppm. For the benzyl derivatives (**6**–**10**), at least two aromatic hydrogens were observed between δ_H_ 7.82–6.63 ppm, along with six aromatic carbons between δ_C_ 153.5–104.7 ppm. Additionally, a signal corresponding to the benzylic *sp*^3^ carbon appeared at approximately δ_C_ 70 ppm and δ_H_ 5.16 (*s*, 2H). Compounds **5**, **8**, **9**, and **10** have not been previously reported in the literature, and their structures were confirmed using high-resolution mass spectrometry (HRMS). All obtained spectra are described in the Supplementary Material.

The homoisoflavonoid derivatives (**11**–**25**) were synthesized by base-catalyzed condensation of chroman-4-one derivatives (**2**–**10**) with benzaldehyde or derivatives [29,35,36]. The yields ranged from 11% to 61%. In the ^1^H NMR and ^13^C NMR-APT spectra of these derivatives, the absence of the signal at δ_H_ 2.66 (*t*, *J* = 6.7 Hz; 2H), previously assigned to hydrogen at C-3, was observed. Instead, a new signal at δ_H_ 7.85 (*t*, *J* = 2.0 Hz, 1H; H-7″) appeared, corresponding to hydrogen bonded to olefinic carbon (δ_C_ 136.9). Additionally, the δ_H_ 4.45 (*t*, *J* = 6.1 Hz; 2H) ppm signal, originally associated with hydrogen at C-2, shifted to δ_H_ 5.33 (*d*, *J* = 2.0 Hz; 2H; H-2). All these data indicate the formation of a homoisoflavonoid featuring an *α*,*β*-unsaturation adjacent to the carbonyl group.

The geometric stereochemistry of homoisoflavonoid derivatives was determined through detailed analysis of their ^1^H NMR spectra, following established criteria reported in the literature. In α,β-unsaturated carbonyl systems, the olefinic proton (=CH; H-7″) typically appears at a lower field (δ_H_ 7.90–7.70), for the *E*-isomer (*trans*), and at a higher field (δ_H_ 7.10–6.80) for the *Z*-isomer(*cis*). Additionally, the methylene protons (–CH_2_–; H-2) adjacent to the double bond also show distinct chemical shifts: δ_H_ 5.20–5.40 for *E*-isomers and δ_H_ 5.00–4.80 for *Z*-isomers [37,38].

For all homoisoflavonoid derivatives (**11**–**25**), the olefinic proton consistently appeared at approximately δ_H_ 7.85, and the adjacent methylene protons were observed around δ_H_ 5.33. Therefore, based on these consistent spectral features and the absence of signals typical of *Z*-isomers, the double bond geometry of all target compounds was assigned as (*E*).

For compounds **11**–**19**, five aromatic hydrogens were detected in the δ_H_ 7.50–7.20 ppm range, while aromatic carbons were observed in the δ_C_ 132.0–128.0 ppm range. For compounds **20**–**25**, four aromatic hydrogen signals were detected in the δ_H_ 7.34–6.82 ppm range; an aromatic carbon bonded to a methoxy group at δ_C_ 159.8 ppm; five aromatic carbons between δ_C_ 132–114 ppm; a methoxy group at δ_H_ 3.83 (*s*, H = 3); and a corresponding ^13^C signal at δ_C_ 55.5 ppm. Compounds **12**, **14**–**25** have not been previously reported in the literature, and their structures were confirmed by HRMS. All obtained spectra are provided in the Supplementary Material. Scheme 1 illustrates the general synthesis of the compounds.

### 2.2. Antimicrobial Activity of Compounds ***1***–***25***

The antimicrobial activity of compounds (**1**–**25**) was evaluated against medically significant bacteria and fungi [37,38,39,40,41,42], including strains of *Sthaphylococcus epidermidis*, *Pseudomonas aeruginosa*, *Salmonella enteritidis*, *Candida albicans*, *C. tropicalis*, *Nakaseomyces glabratus* (formerly *C. glabrata*), *Aspergillus flavus*, *Penicillium citrinum*. The results were expressed based on the minimum inhibitory concentration (MIC), and activity was classified according to the following criteria: < 100 μg/mL = good activity; 100–500 μg/mL = moderate activity; and 500–1000 μg/mL = weak activity [43,44]. Table 1 and Table 2 show the MIC values of compounds **1**–**25** against bacteria and fungi, respectively, as well the minimum bactericidal concentration (MBC) and the minimum fungicidal concentration (MFC).

Regarding antifungal activity, only compound **1** exhibited moderate activity against filamentous fungi (*A. flavus* and *P. citrinum*), while the other compounds showed weak or no antifungal effect. In contrast, against *Candida* species and *N. glabratus*, compounds **3**, **8**, **12**, **20**, **21**, and **22** demonstrated moderate inhibition, whereas compounds **1** and **2** showed good antifungal activity. Remarkably, compounds **1**, **2,** and **21** were more potent than the positive control (fluconazole), as indicated by their lower MIC values (<417.9 µM). In light of these findings, a molecular modeling study was conducted for compounds **1**, **2**, and **21** to predict their potential molecular targets against *C. albicans.*

### 2.3. Molecular Modeling Study

Modeling studies were conducted to investigate the probable antifungal mechanism of action of the most active compounds against *C. albicans*, as the best bioactivity results were obtained against this fungus. The modeling methodology, as described in the Materials and Methods Section, involved identifying potential molecular targets for compounds **1**, **2**, and **21**. The first step of this methodology consisted of applying a homology-based target fishing approach, rather than selecting a random set of proteins without a clear rationale. These three compounds were selected because they exhibited the highest antifungal activity among all evaluated derivatives.

The compounds were subsequently docked into the predicted protein targets to generate initial ligand–receptor binding hypotheses. While docking algorithms offer a rapid means to estimate binding poses and affinities, their simplified scoring functions and static treatment of receptor flexibility can lead to inaccuracies [45]. Post-processing these docking results with molecular dynamics simulations and rigorous free energy calculations, such as MM-PBSA, allows for a more accurate assessment of binding stability by incorporating receptor dynamics and solvation effects, thereby overcoming key limitations of traditional docking approaches [46]. Therefore, the best-scoring docking complexes were subjected to MD-based binding free energy estimation, which was then used to select the most probable targets of each compound in *C. albicans.*

The predicted targets are summarized in Table 3, which includes the UniProt accession number, a functional description, the protein ID used throughout the study, and the associated compound. Notably, despite structural similarities among the three compounds, no protein target was shared by all of them. Moreover, compound **1** had no targets in common with compounds **2** or **21**.

The docking protocol was validated by redocking the ligand bound to FBA1, the only protein among those studied for which an experimental structure is available. The complex was downloaded from the Protein Data Bank (code 7V6G), and the procedure described in the Materials and Methods Section was followed. The ligand was then removed, converted to SMILES notation, and a 3D structure was generated. This processing step, starting from the 2D representation, helps to avoid biases introduced when using a bound conformer as the initial structure for docking calculations. Subsequently, docking and rescoring were performed, yielding three solutions with aggregated scores greater than 1.0. The top-scoring conformer had an RMSD of 2.5 Å relative to the crystallized ligand, which can be considered moderate. However, visual inspection of this docking solution revealed a similar orientation to the experimental ligand. Given that our approach includes the refinement of docking solutions using MD tools, we consider that this validation step confirms the docking protocol’s ability to generate predictions close to the actual binding modes.

Compounds **1**, **2**, and **21** were docked into all the proteins listed in Table 3, except for NAD1, which was excluded due to its involvement in the large multicomponent complex I and limited structural information [47]. The molecular docking calculations resulted in 131 compound–protein complexes that satisfied the selection criteria outlined in the Materials and Methods Section. The detailed scores for these docking solutions are provided as Supporting Information in Table S1. The predicted docking complexes are provided as Supplementary Materials in PDB format within the file Complexes-PDBs.zip.

**Figure 2:**
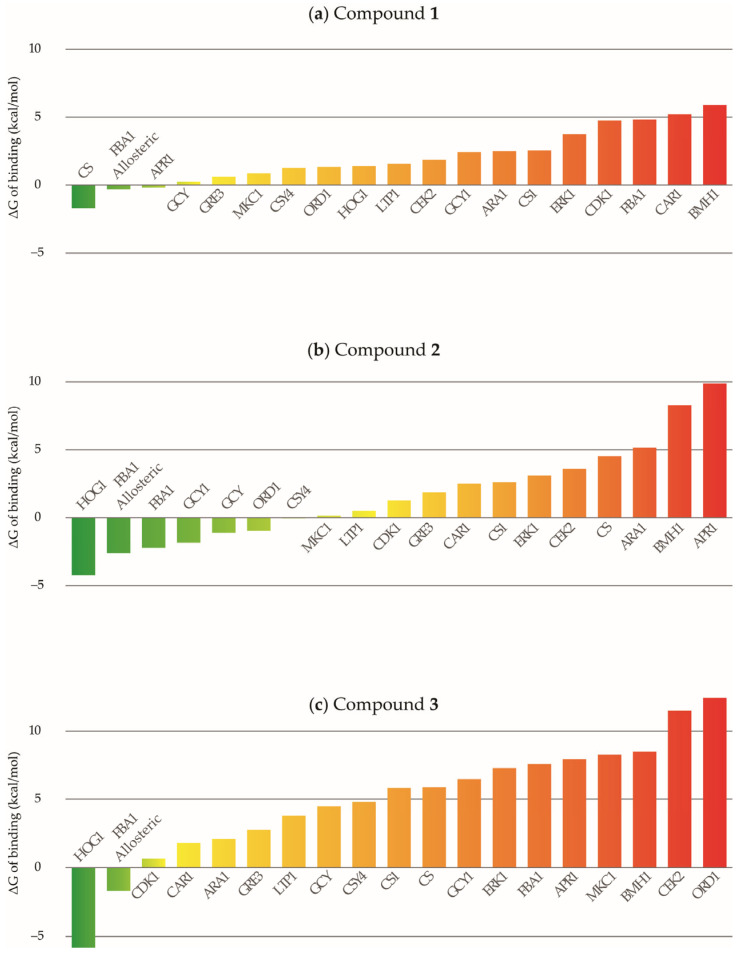
Predicted free energies of binding for the studied compounds to their potential targets in *C. albicans*: (**a**) compound 1, (**b**) compound 2, and (**c**) compound 3.

All these complexes were next subject to the MD-guided calculation of free energies of binding, which are summarized in Figure 2. In this figure only the best (lowest-energy) docking solution per protein is presented for each compound, while the full results are given as Supporting Information in Table S2. This approach resulted in a total MD simulation time of 2.62 µs. We opted for five short MD production runs per complex to achieve a better exploration of the system’s conformational space over a single trajectory approach, considering that literature reports establish these short simulations are effective for MM-PBSA calculations [46,48]. The MD trajectories obtained in this research as well as the Amber topology files are publicly available in the repository available at https://doi.org/10.5281/zenodo.16555424 (accessed on 8 August 2025).

The free energy results suggest that the compounds may act through distinct inhibitory mechanisms in *C. albicans*. Compound **1** is predicted to preferentially bind CS, which is not among the top targets of the other compounds. In contrast, HOG1 and the allosteric site of FBA1 emerged as the top predicted targets for compounds **2** and **21**. To investigate whether the compounds could share common binding sites, their binding poses in CS, HOG1, and the FBA1 allosteric site were examined in detail.

In CS, the 4-chromanone moieties of compounds **1**, **2**, and **21** occupy different regions of the binding cavity. To address potential inaccuracies in docking, the lowest-energy pose of compound **1** was used as a reference. Overlapping conformers were generated for the other compounds. For compound **21**, no overlapping pose could be obtained due to steric hindrance from bulky substituents. Compound **2** yielded an overlapping pose, but it had a poor predicted free energy of binding (8.60 kcal/mol).

For HOG1, the top docking poses of compounds **1** and **2** overlap with that of compound **21**. The larger substituents in compound **21** appear essential for optimal binding. An alternate pose of compound **2**, featuring a different chromanone orientation, also yielded favorable energy and was used to guide a similar pose for compound **1**. However, the predicted energy for this model was less favorable (2.45 kcal/mol).

An analogous analysis was performed for the allosteric inhibition site of FBA1. Given the similar binding energies of the two poses studied for **2**, both were used as references for generating additional overlapping binding hypotheses of the other compounds. These calculations showed no improvement on the predicted binding energy of **1** to this cavity (0.09 kcal/mol and 3.27 kcal/mol). In contrast, one of the overlapping conformers of compound **21** showed improved binding energy (−4.68 kcal/mol) that suggests a binding to the allosteric site of FBA1 resembling the predicted conformation for **2**.

**Figure 3:**
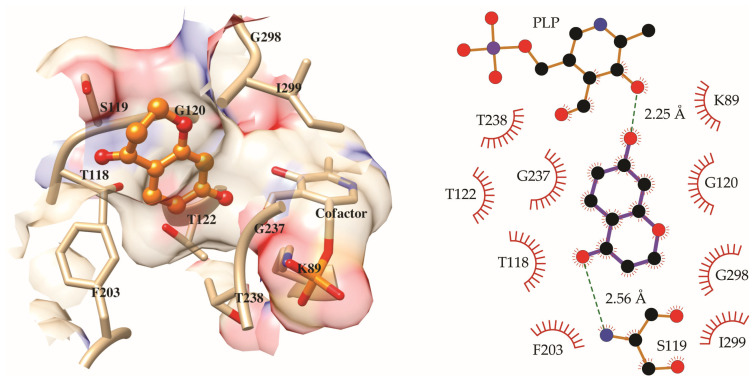
Predicted binding mode of compound **1** to CS and network of intermolecular interactions.

**Figure 4:**
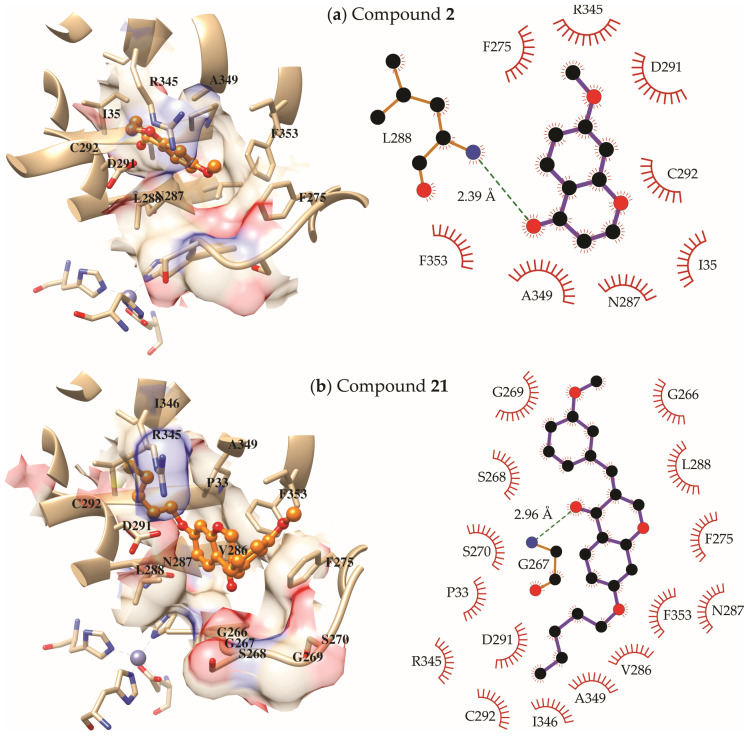
Predicted binding modes and networks of observed intermolecular interactions for the allosteric site of FBA1 for (**a**) compound **2** and (**b**) compound **21**.

**Figure 5:**
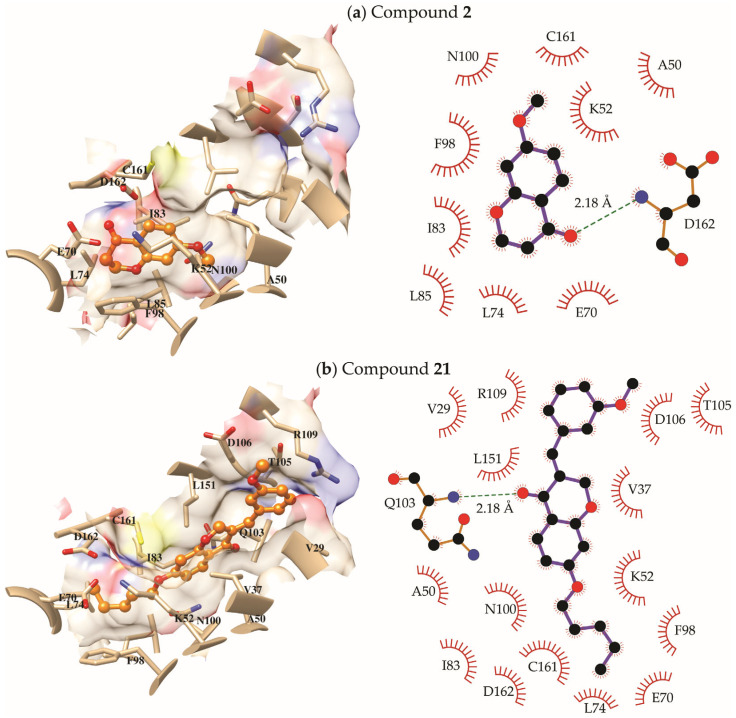
Predicted orientations and networks of intermolecular interactions for HOG1 for (**a**) compound **2** and (**b**) compound **21**.

These additional analyses support the conclusion that the antifungal activity of compound **1** is primarily mediated through its interaction with cysteine synthase (CS), whereas compounds **2** and **21** are more likely to exert their effects via binding to HOG1 and the allosteric site of FBA1. To gain deeper insights into the binding modes of these compounds to their most probable targets in *C. albicans*, the predicted ligand–receptor complexes of compound **1** with CS, and compounds **2** and **21** with HOG1 and the allosteric site of FBA1, were further examined. These binding modes are shown in Figure 3, Figure 4, and Figure 5, respectively. In each figure, only the residues that interact with the ligand in at least 50% of the MD snapshots used for MM-PBSA calculations are labeled and displayed in the interaction diagrams. The interaction networks were generated using Cytoscape 3.10.3 (https://cytoscape.org/, accessed on 14 June 2025) [49] and UCSF Chimera 1.19 (https://www.cgl.ucsf.edu/chimera/, accessed on 14 June 2025) [50]. The structures shown represent the most populated cluster obtained from the grouping of 100 MD snapshots and thus correspond to the representative ligand conformer from the simulations.

As shown in Figure 3, the predicted binding mode of **1** to CS is stabilized by two key hydrogen bonds: one between the carbonyl oxygen of the chromanone ring and the amino group of S119, and a second between the hydroxyl substituent of the ligand and the PLP (pyridoxal phosphate) cofactor. This latter interaction may account for the decreased stability of compound **2** in CS, as the methoxy group present in **2** cannot act as a hydrogen bond donor. In addition to these interactions, the hydroxyl group of **1** interacts with K89, T122, and T239, while the remainder of the molecule fits tightly within a narrow cavity formed by residues T118, S119, G120, F203, G237, G298, and I299.

In the allosteric site of FBA1, MD simulations starting from overlapping poses of compounds **2** and **21** revealed different final binding modes (Figure 4). Both form a hydrogen bond via the chromanone carbonyl, compound **2** to the backbone, and L288 and compound **21** to G267. Compound **2**’s chromanone nucleus occupies a hydrophobic region formed by I35, N287, L288, D291, C292, R345, and A349. Its methoxy group interacts with F275 and F353. In contrast, compound **21**’s pentyloxy group occupies this same hydrophobic region, while the G266–S270 loop adapts to accommodate its larger core and methoxybenzylidene substituent, which also interacts with F275 and F353.

Finally, the predicted binding modes of **2** and **21** to HOG1 (Figure 5) present a similar scenario as in the allosteric site of FBA1. For this protein, compound **2** overlaps with the pentyloxy moiety of **21**, orientating parallel to F98 in a position favorable for π–π stacking interactions. The compound is located in a mainly hydrophobic region also defined by A50, K52, E70, L74, I83, L85, N100, C161, and D162, with the carbonyl oxygen of the ligand accepting a hydrogen bond from the backbone of D162. Interestingly, the methoxy group of **2** occupies a small sub-pocket lined by A50, K52, F98, and N100, making numerous hydrophobic contacts with their side chains. We speculate that the lack of this substituent in compound **1** (a smaller hydroxyl group is present) might contribute to the unfavorable free energy of binding predicted for it when forced to overlap with **2** in HOG1. In the case of **21**, additionally to the previously described interactions with the pentyloxy group, one hydrogen bond between the carbonyl oxygen and the backbone of Q103 is observed. Furthermore, the 4-chromanone core occupies a region delimited by V37 and L151, while the rest of the compound makes contacts with V29, T105, D106, and R109.

One additional experiment was conducted to assess the conformational stability of the complexes identified as the most probable binding modes for compounds **1**, **2** and **21**, as discussed above. For this purpose, each 4 ns MD replica was extended to a total production time of 20 ns, totaling 100 ns of simulation time per complex. The root mean square deviation (RMSD) plots for the ligand and protein backbone from these extended simulations are provided in the Supplementary Material (Figures S93–S97). This analysis shows that, even at longer simulation times, the ligand remains stable in all systems, with RMSD values below 2 Å relative to its starting conformation. A similarly stable behavior was observed for the proteins in almost all systems, except for the two complexes with FBA1 where, despite overall stability, RMSD values reached up to 5 Å.

To investigate whether these higher RMSD values could affect complex stability, root mean square fluctuation (RMSF) values were calculated for each protein residue. Visual representations of these five complexes, showing the RMSF values per amino acid, are provided in Supplementary Material (Figures S98–S102). These figures reveal that, for all three proteins, CS, HOG1, and FBA1, the most flexible regions are located away from the ligand-binding site. In other words, although higher RMSD values are observed for the FBA1 backbone, the flexibility is primarily localized in regions that do not directly interfere with ligand–receptor interactions. Overall, these analyses indicate that, even at longer simulation times than those used for the MM-PBSA calculations, the complexes of CS with compound **1**, and of HOG1 and FBA1 with compounds **2** and **21**, exhibit structural indicators of stability.

Overall, these findings suggest that compound **1** may exert antifungal activity primarily through inhibition of CS, while compounds **2** and **21** are more likely to target HOG1 and the allosteric site of FBA1. Although little is known about CS in *Candida* species, studies indicate that disruptions in cysteine metabolism impair growth, sulfur assimilation, biofilm formation, and virulence [51,52,53]. This suggests that this enzyme could be a promising but still underexplored target for antifungal agents that deserve future investigations. On the contrary, compounds **2** and **21** are more likely to inhibit the HOG1 kinase and FBA1 by binding to their allosteric modulation sites. In contrast to CS, both HOG1 and FBA1 have been previously explored as targets for antifungal compounds. FBA1 is essential for *C. albicans* survival and virulence, and it has been successfully targeted by covalent and allosteric inhibitors, validating FBA1 as a viable and emerging antifungal drug target, especially against resistant strains [54,55,56]. Likewise, HOG1 is a validated antifungal target in *C. albicans*, regulating key virulence traits such as morphogenesis, immune evasion, and stress resistance [57,58,59,60].

## 3. Discussion

All compounds prepared in this study share chemical similarities, allowing an evaluation of structural features influencing antimicrobial activity, such as the extension of the carbon chain, presence of branching, insertion of activating or deactivating groups on the aromatic ring, and other factors [61].

Of the 25 compounds prepared, 13 exhibited antibacterial and antifungal activity, specifically compounds **1**–**3**, **8**, **10**–**14**, **18**, and **20**–**22**. In general, the compounds exhibited MIC values ranging from 64 to 1024 μg/mL (359.2 to 3845.5 μM), depending on the group of microorganisms. Notably, compounds **1**–**3**, **8**, **12**, and **20**–**22** demonstrated inhibitory effects on microbial growth against bacteria and moderate antimicrobial activity for fungi [62].

Compounds **1** and **2** exhibited an MIC of 128 μg/mL (779.7 μM and 718.4 μM, respectively) against *S. epidermidis* and *P. aeruginosa* and of 256 μg/mL (1559.5 μM and 1436.8 μM, respectively) against *S. enteritidis*. Meanwhile, against fungi of the genus *Candida* and *N. glabratus* they had good activity, 64 μg/mL (389.9 μM and 359.2 μM, respectively). The data shows that the substitution of the hydroxyl group with a methoxy group did not affect antimicrobial activity, except against filamentous fungi, where the phenolic compound (**1**) displayed an MIC of 256 μg/mL (1559.5 μM), whereas the methoxylated derivative (**2**) exhibited an MIC of 512 μg/mL (2873.6 μM). The MFC/MBC values for these two compounds ranged from 256 to 512 μg/mL (1436.8 μM to 3119.0 μM). Compounds **1** and **2** were more potent than fluconazole against *Candida* species and *N. glabratus*, as their MIC values were lower than 417.9 μM. Xie at al. (2014), concluded that hydroxyl groups on aromatic ring A of flavonoids and isoflavonoids can improve antimicrobial activity. Additionally, methylation of the hydroxyl groups generally decreases this activity [63]. However, in another study, a series of 2-vinylchroman-4-one was prepared, and its antimicrobial activity against Gram-positive and Gram-negative bacteria was evaluated. The results indicated that the *para*-hydroxylated analog did not exhibit bacterial activity, while the *meta*-methoxylated derivative showed potent activity against microorganisms, including *S. epidermidis* and methicillin-resistant *S. aureus* [64].

Upon replacing the hydroxyl group (**1**) with a propyl group (**3**), a significant decrease in antimicrobial activity was observed against all tested microorganisms. This compound exhibited an MIC of 128 μg/mL (620.6 μM) against *C. albicans*, 256 μg/mL (1241.3 μM) against *C. tropicalis* and *N. glabratus*, and 512 μg/mL (2482.6 μM) against filamentous fungi. Compound **3** also inhibited bacteria *S. epidermidis* (256 μg/mL; 1241.3 μM), *P. aeruginosa*, and *S. enteritidis* (512 μg/mL; 2482.6 μM). The MFC/MBC values for this compound ranged from 512 to 1024 μg/mL (2482.6 to 4965.2 μM). Finally, when the phenolic hydroxyl group (**1**) was replaced by a branched chain (isopropyl) (**4**) or by a longer and more apolar carbon chain (pentyl) (**5**), antimicrobial activity was completely abolished.

When analyzing chromanones with benzyl substituents introduced at the phenolic hydroxyl group (**6**–**10**), it was observed that most compounds did not exhibit antimicrobial activity, except for compounds **8** and **10**, which feature substituents at the *meta* positions of the introduced aromatic ring. The disubstituted compound (**8**), containing two methyl groups—known activators of the aromatic ring—demonstrated antimicrobial activity. It had an MIC of 256 μg/mL (906.7 μM) against Gram-positive/Gram-negative bacteria. At this concentration, it also had action against yeasts and an MIC of 512 μg/mL (1813.4 μM) against filamentous fungi. The MFC/MBC values were established as 512 μg/mL (1813.4 μM). In contrast, compound **10**, which carries a CF_3_ group—a known deactivator of the aromatic ring—exhibited weak antimicrobial activity against bacteria, yeasts, and filamentous fungi, with an MIC of 512 μg/mL to bacteria and fungi (1588.7 μM) and an MIC of 1024 μg/mL (3177.4 μM) against filamentous fungi.

For homoisoflavonoid derivatives (**11**–**25**), all compounds with alkyl substitutions at the phenolic hydroxyl group (**11**–**14**, **20**, and **21**) exhibited antimicrobial activity. In contrast, homoisoflavonoid derivatives with aryl substituents introduced at the phenolic hydroxyl group showed no antimicrobial activity, except for compounds **18** and **22**. When evaluating the influence of adding a benzyl group to chromanones with alkyl substitutions at the phenolic hydroxyl group (**2**–**5**), the antimicrobial activity varied. Compounds **11** and **12** exhibited reduced activity compared to their precursors **2** and **3**, respectively. Compound **11** exhibited an MIC of 512 μg/mL (1922.7 μM) against Gram-positive/Gram-negative bacteria and yeasts, while **12** exhibited an MIC of 256 μg/mL (869.7 μM). Both compounds showed MIC values of 1024 μg/mL (3845.4 μM and 3478.8 μM, respectively) against filamentous fungi. Moreover, MFC/MBC values, which were only determined for compound **12**, were 1024 μg/mL (3478.8 μM). However, for compounds **13** and **14**, the addition of a benzyl group promoted antimicrobial activity, as these compounds exhibited MIC values ranging from 512 to 1024 μg/mL (1588.1 to 3478.8 μM), whereas their precursors (**4** and **5**, respectively) showed no activity. Similarly, compound **18**, which features a *para*-positioned SCF_3_ group—known as a deactivator of the aromatic ring—exhibited MIC values of 512 μg/mL (1161.7 μM) against all tested microorganisms. Notably, its precursor (**9**) displayed no antimicrobial activity, indicating that the introduction of the benzyl group conferred biological activity. Similar results were reported in a study in which a series of homoisoflavonoids was prepared from chroman-4-one, and it was observed that the homoisoflavonoid derivative exhibited better antifungal activity than its precursor, especially against *C. albicans*, *N. glabratus* (formerly *Candida glabrata*), and *C. tropicalis* [29].

Compounds **20** and **21** demonstrated enhanced antimicrobial activity compared to their precursors, compounds **3** and **5**, respectively. Compound **20** had an MIC of 128 μg/mL (394.6 μM) against *S. epidermidis*, while compound **21** had inhibitory action at 128 μg/mL (363.2 μM) against bacteria, being more potent than the positive control (gentamicin) against these microorganisms, except for *S. epidermidis*.

Compound **20** was bioactive against yeast at a concentration of 256 μg/mL (789.2 μM). In addition, compound **21** was more potent, with an MIC of 128 μg/mL (363.2 μM) against yeast, being more potent than the positive control (fluconazole) against these fungi. Both compounds exhibited an MIC of 512 μg/mL (1578.4 μM and 1452.8 μM, respectively) against filamentous fungi, with the same MFC/MBC values.

Likewise, compound **22** exhibited MIC values ranging from 256 to 512 μg/mL (567.2 to 1134.4 μM) against all tested microorganisms, with MFC/MBC values of 512 μg/mL (1134.4 μM). A comparison with its chromanone precursor (**6**) and the structurally related homoisoflavonoid (**15**) revealed that the incorporation of a 3-methoxybenzyl group promoted biological activity. These findings indicate that the addition of an activating group (methoxy) at the *meta* position of the aromatic ring significantly improved antimicrobial activity when compared to the unsubstituted derivatives (**12**, **14,** and **15**). These data corroborate the findings of Ferreira et al., (2022), which demonstrated that the addition of a methoxyl group to the aromatic ring B of homoisoflavonoids contributes to an improvement in antifungal activity, especially when they are added in the *meta* position [29].

## 4. Materials and Methods

### 4.1. Chemistry

All chemical reagents used were of analytical grade and purchased from Merck (Ames, USA or Darmstadt, Germany). Adsorption column chromatography (CC) was performed using silica gel 60 (ART 7734, Merck, Darmstadt, Germany) as the stationary phase. The ^1^H NMR and ^13^C NMR-APT spectra were obtained using Ascend™ Bruker spectrometers (400 MHz for ^1^H and 100 MHz for ^13^C) and a Varian NMR System (500 MHz for ^1^H and 125 MHz for ^13^C). The compounds were dissolved in deuterated chloroform (CDCl_3_) or deuterated dimethyl sulfoxide (DMSO-d_6_), Ames, USA. Infrared (IR) spectra were obtained using a Cary 630 FTIR Agilent spectrometer operating in the spectral range of 4000–400 cm^−1^. Mass spectra were acquired using an Ultraflex Speed MALDI TOF/TOF spectrometer (Bruker Daltonik GmbH, Bremen, Germany) equipped with a high-performance solid-state laser (*λ* = 355 nm) and a reflector and operated in positive ion mode. Melting points were determined using a Microquímica Equipamentos apparatus (Model MQAPF 302, MicroQuímica Ltd, Santa Catarina, Brazil). All reactions were monitored by analytical thin-layer chromatography (TLC) using aluminum sheets precoated with silica gel 60 as the stationary phase (silica gel 60 F254-Merck, Darmstadt, Germany). The mobile phase consisted of mixtures of hexane and ethyl acetate in various ratios. Visualization was performed under UV light at 254 nm and by spraying with a 5% sulfuric acid solution in ethanol, followed by gentle heating. The purity of all synthesized compounds was assessed by ^1^H NMR spectral analysis, with estimated purities ranging from 95% to 99%. ^1^H NMR spectra for each compound are provided in the Supporting Information (Figures S1–S92), which contain all spectrometric data of the compounds.

#### 4.1.1. General Procedure for Obtaining 7-Hydroxychroman-4-One (**1**)

Resorcinol (4147 mg, 37.67 mmol), 5820 mg (38.05 mmol) of 3-bromopropionic acid, and 10 mL (113 mmol) of triflic acid were added to a round-bottom flask. The reaction flask was fitted with a condenser, and the reaction was carried out under magnetic stirring and heating (80 °C) for 1 h. After this period, the product was cooled to room temperature for 15 min. Subsequently, 100 mL of chloroform was added, and the reaction mixture was extracted in a separatory funnel using 100 mL of distilled water. The aqueous phase was treated with 2 × 100 mL of chloroform, and the organic phase was treated with 100 mL of water and dried over anhydrous sodium sulfate. The mixture was filtered, and the reaction product was concentrated under reduced pressure to obtain 3-bromo-1-(2,4-dihydroxyphenyl)propan-1-one (2402 mg; 9.80 mmol) [34]. This was then added to a NaOH solution (2 M, 80 mL) at 5 °C. The reaction was stirred at room temperature for 2 h. Then, it was cooled to 5 °C, and the pH was adjusted to 2 using H_2_SO_4_ (6 M). The reaction product was extracted using chloroform (3 × 50 mL), and the organic phase was dried with anhydrous sodium sulfate and concentrated under reduced pressure to obtain compound **1** (821 mg; 5.00 mmol) [34].

#### 4.1.2. General Procedure for Obtaining Chroman-4-Ones (**2**–**10**)

To a round-bottom flask, 100 mg of compound **1** (0.61 mmol), 0.91 mmol (1.5 eq.) of K_2_CO_3_, and 2 mL of dimethylformamide (DMF) were added. Subsequently, 0.73 mmol (1.2 eq.) of the corresponding alkyl or benzyl halide was added. The reaction was conducted at room temperature under magnetic stirring for 24 h. Then, 10 mL of water was added, and the product was extracted with dichloromethane (3 × 15 mL) in a separatory funnel. The organic phase was dried with anhydrous sodium sulfate, filtered, and concentrated under reduced pressure. The compounds were purified by column chromatography using a hexane and ethyl acetate mixture as the mobile phase in a gradient of increasing polarity [18].

7-Pentyloxychroman-4-one (**5**): Colorless oil. Yield: 77% (0.47 mmol; 109.0 mg); TLC (8:2 hexane/EtOAc, R_f_ = 0.66); IR υmax (cm^−1^): 2934, 1681, 1603, 1438, 1382, 1331, 1250, 1161, 1117; ^1^H NMR (500 MHz, CDCl_3_): δ_H_ 7.82 (*d*, *J* = 8.7 Hz, 1H; H-5); 6.56 (*dd*, *J* = 8.9, 2.4 Hz, 1H; H-6); 6.38 (*d*, *J* = 2.4 Hz, 1H; H-8); 4.50 (*t*, *J* = 6.2 Hz, 2H; H-2); 3.97 (*t*, *J* = 6.6 Hz, 2H; H-1′); 2.74 (*t*, *J* = 6.4 Hz, 2H; H-3); 1.80 (*quint*, *J* = 6.5 Hz, 2H; H-2′); 1.48–1.32 (*m*, 4H; H-3′; H-4′); 0.93 (*t*, *J* = 7.2 Hz, 3H; H-5′). ^13^C NMR-APT(125 MHz, CDCl_3_): δ_C_ 190.91; 166.00; 164.19; 129.22; 115.49; 110.68; 101.57; 68.84; 67.75; 37.83; 29.06; 28.47; 22.78; 14.37. MALDI-TOF (*m*/*z*): C_14_H_18_O_3_, theoretical value calculated for [M + H]^+^ = 235.1329, found [M + H]^+^ = 235.1329.

7-((3,5-Dimethylbenzyl)oxy)chroman-4-one (**8**): Colorless oil. Yield: 58% (0.35 mmol; 99.0 mg); TLC (8:2 hexane/EtOAc, R_f_ = 0.52); IR υmax (cm^−1^): 3016, 2917, 1680, 1602, 1438, 1379, 1251, 1160, 1117; ^1^H NMR (500 MHz, DMSO-d_6_): δ_H_ 7.68 (*d*, *J* = 8.8 Hz, 1H; H-5); 7.03 (*s*, 2H; H-2′; H-6′); 6.97 (*s*, 1H; H-4′); 6.70 (*dd*, *J* = 8.8, 2.4 Hz, 1H; H-6); 6.60 (*d*, *J* = 2.4 Hz, 1H; H-8); 5.08 (*s*, 2H; H-7′); 4.50 (*t*, *J* = 6.8 Hz, 2H; H-2); 2.70 (*t*, *J* = 5.8 Hz, 2H; H-3); 2.27 (*s*, 6H; H-8′; H-9′). ^13^C NMR-APT (125 MHz, DMSO-d_6_): δ_C_ 190.47; 164.98; 163.82; 138.04; 136.57; 129.92; 128.64; 125.96; 115.43; 110.68; 102.19; 70.28; 67.57; 37.34; 21.34. MALDI-TOF (*m*/*z*): C_18_H_18_O_3_, theoretical value calculated for [M + H]^+^ = 283.1329, found [M + H]^+^ = 283.1330.

7-((4-((Trifluoromethyl)thio)benzyl)oxy)chroman-4-one (**9**): White solid. Yield: 65% (0.40 mmol; 140.1 mg); M.P.: 137.2–137.9 °C; TLC (8:2 hexane/EtOAc, R_f_ = 0.44; IR υmax (cm^−1^): 2889, 1683, 1605, 1494, 1439, 1380, 1334, 1255, 1110; ^1^H NMR (500 MHz, DMSO-d_6_): ^1^H NMR (500 MHz, DMSO-d_6_): δ_H_ 7.75 (*d*, *J* = 8.2 Hz, 2H; H-3′; H-5′); 7.70 (*d*, *J* = 8.8 Hz, 1H; H-5); 7.60 (*d*, *J* = 8.5 Hz, 2H; H-2′; H-6′); 6.72 (*dd*, *J* = 8.8, 2.4 Hz, 1H; H-6); 6.63 (*d*, *J* = 2.4 Hz, 1H; H-8); 5.27 (*s*, 2H; H-7′); 4.50 (*t*, *J* = 6.8 Hz, 2H; H-2); 2.71 (*t*, *J* = 5.9 Hz, 2H; H-3). ^13^C NMR-APT (125 MHz, DMSO-d_6_): δ_C_ 190.03; 164.14; 163.35; 140.18; 136.39; 128.89; 128.29; 122.47; 115.20; 110.15; 101.89; 68.73; 67.14; 36.87. MALDI-TOF (*m*/*z*): C_17_H_13_F_3_O_3_S, theoretical value calculated for [M + H]^+^ = 355.0610, found [M + H]^+^ = 355.0611.

7-((3-(Trifluoromethyl)benzyl)oxy)chroman-4-one (**10**): White solid. Yield: 74% (0.45 mmol; 145.3 mg); M.P.: 132.6–133.3 °C; TLC (8:2 hexane/EtOAc, R_f_ = 0.44); IR υmax (cm^−1^): 2883, 1681, 1604, 1440, 1380, 1328, 1251, 1160, 1117; ^1^H NMR (500 MHz, DMSO-d_6_): δ_H_ 7.82 (*brs*, 1H; H-2′); 7.76 (*d*, *J* = 7.7 Hz, 1H; H-4′); 7.72–7.69 (*m*, 2H; H-5; H-6′); 7.65 (*t*, *J* = 7.7 Hz, 1H; H-5′); 6.73 (*dd*, *J* = 8.8, 2.4 Hz, 1H; H-6); 6.65 (*d*, *J* = 2.4 Hz, 1H; H-8); 5.29 (*s*, 2H; H-7′); 4.51 (*t*, *J* = 6.1 Hz, 2H; H-2); 2.71 (*t*, *J* = 6.0 Hz, 2H; H-3). ^13^C NMR-APT (125 MHz, DMSO-d6): δ_C_ 190.04; 164.14; 163.36; 137.85; 131.79; 129.69; 128.27; 124.78 (*q*, ^3^*J*_C,F_ = 3.7 Hz; C-2′); 124.17 (*q*, ^3^*J*_C,F_ = 3.9 Hz; C-4′); 115.20; 110.17; 101.87; 68.77; 67.14; 36.88. MALDI-TOF (*m*/*z*): C_17_H_13_F_3_O_3_, theoretical value calculated for [M + H]^+^ = 323.0890, found [M + H]^+^ = 323.0889.

#### 4.1.3. General Procedure for Obtaining Homoisoflavonoids (**11**–**25**)

The previously obtained chromanone (**2**–**10**; 100 mg) was added to a reaction flask along with 1.1 equivalents of benzaldehyde or 3-methoxybenzaldehyde, followed by 2 equivalents of pyrrolidine and 2 mL of a 1:1 mixture of methanol and dichloromethane. The reaction was conducted under magnetic stirring at room temperature for 24 h when using benzaldehyde and for 48 h when using 3-methoxybenzaldehyde. The solvent was partially removed under reduced pressure, and the mixture was transferred to a separatory funnel for extraction with 10 mL of distilled water and dichloromethane (3 × 10 mL). The organic phase was dried with anhydrous sodium sulfate, and the product was filtered. The solvent was then removed under reduced pressure, and the reaction product was purified by column chromatography using silica gel 60 with a hexane and ethyl acetate mobile phase in a gradient of increasing polarity [35].

(*E*)-3-Benzylidene-7-propoxychroman-4-one (**12**): White solid. Yield: 57% (0.28 mmol; 81.1 mg); M.P.: 67.4–68.4 °C; TLC (8:2 hexane/EtOAc, R_f_ = 0.82); IR υmax (cm^−1^): 3027, 2965, 1667, 1601, 1437, 1384, 1335, 1254, 1160, 1103; ^1^H NMR (500 MHz, CDCl_3_): δ_H_ 7.96 (*d*, *J* = 8.9 Hz, 1H; H-5); 7.84 (*brs*, 1H; H-7″); 7.46–7.38 (*m*, 3H; H-2″; H-6″; H-4″); 7.31–7.28 (*m*, 2H; H-3″; H-5″); 6.63 (*dd*, *J* = 8.8, 2.4 Hz, 1H; H-6); 6.38 (*d*, *J* = 2.4 Hz, 1H; H-8); 5.32 (*d*, *J* = 2.0 Hz, 2H; H-2); 3.95 (*t*, *J* = 6.6 Hz, 2H; H-1′); 1.82 (*sext*, *J* = 7.0 Hz, 2H; H-2′); 1.03 (*t*, *J* = 7.4 Hz, 3H; H-3′). ^13^C NMR-APT (125 MHz, CDCl_3_): δ_C_ 181.11; 165.81; 163.23; 136.78; 134.72; 131.08; 130.01; 129.78; 129.34; 128.79; 115.69; 110.98; 101.34; 70.06; 67.96; 22.47; 10.55. MALDI-TOF (*m*/*z*): C_19_H_18_O_3_, theoretical value calculated for [M + H]^+^ = 295.1329, found [M + H]^+^ = 295.1329.

(*E*)-3-Benzylidene-7-pentoxychroman-4-one (**14**): White solid. Yield: 44% (0.19 mmol; 60.0 mg); M.P.: 59.7–60.2 °C; TLC (8:2 hexane/EtOAc, R_f_ = 0.84); IR υmax (cm^−1^): 3030, 2930, 1668, 1605, 1439, 1383, 1256, 1166, 1105; ^1^H NMR (400 MHz, CDCl_3_): δ_H_ 7.96 (*d*, *J* = 8.8 Hz, 1H; H-5); 7.85 (*t*, *J* = 2.0 Hz, 1H; H-7″); 7.49–7.33 (*m*, 3H; H-2″, H-6″; H-4″); 7.33–7.26 (*m*, 2H; H-3″; H-5″); 6.62 (*dd*, *J* = 8.9, 2.4 Hz, 1H; H-6); 6.39 (*d*, *J* = 2.4 Hz, 1H; H-8); 5.33 (*d*, *J* = 1.8 Hz, 2H; H-2); 3.99 (*t*, *J* = 6.6 Hz, 2H; H-1′); 1.80 (*quint*, *J* = 6.8 Hz, 2H; H-2′); 1.50–1.31 (*m*, 4H; H-3′; H-4′); 0.93 (*t*, *J* = 7.2 Hz, 3H; H-5′). ^13^C NMR-APT (100 MHz, CDCl_3_): δ_C_ 181.15; 165.85; 163.25; 136.81; 134.76; 131.11; 130.03; 129.81; 129.36; 128.81; 115.71; 111.02; 101.35; 68.63; 67.98; 28.82; 28.23; 22.54; 14.13. MALDI-TOF (*m*/*z*): C_21_H_22_O_3_, theoretical value calculated for [M + H]^+^ = 323.1641, found [M + H]^+^ = 323.1642.

(*E*)-3-Benzylidene-7-((4-bromobenzyl)oxy)chroman-4-one (**15**): White solid. Yield: 43% (0.13 mmol; 54.1 mg); M.P.: 141.2–142.1 °C; TLC (8:2 hexane/EtOAc, R_f_ = 0.68); IR υmax (cm^−1^): 3030, 2923, 1660, 1602, 1459, 1382, 1255, 1169, 1100; ^1^H NMR (400 MHz, CDCl_3_): δ_H_ 7.98 (*d*, *J* = 8.8 Hz, 1H; H-5); 7.85 (*t*, *J* = 1.9 Hz, 1H; H-7″); 7.56–7.48 (*m*, 2H; H-3′; H-5′); 7.46–7.39 (*m*, 3H; H-2″; H-6″; H-4″); 7.31–7.28 (*m*, 4H; H-6′; H-2′; H-3″; H-5″); 6.69 (*dd*, *J* = 8.9, 2.4 Hz, 1H; H-6); 6.45 (*d*, *J* = 2.3 Hz, 1H; H-8); 5.33 (*d*, *J* = 1.8 Hz, 2H; H-2); 5.05 (*s*, 2H; H-7′). ^13^C NMR-APT (100 MHz, CDCl_3_): δ_C_ 181.10; 164.93; 163.16; 137.07; 135.10; 134.67; 132.01; 130.91; 130.04; 130.01; 129.45; 129.21; 128.84; 122.42; 116.26; 111.04; 101.99; 69.66; 68.03. MALDI-TOF (*m*/*z*): C_23_H_17_O_3_Br, theoretical value calculated for [M + H]^+^ = 421.0434, found [M + H]^+^ = 421.0434.

(*E*)-3-Benzylidene-7-((4-chlorobenzyl)oxy)chroman-4-one (**16**): White solid. Yield: 41% (0.14 mmol; 53.1 mg); M.P.: 132.4–133.3 °C; TLC (8:2 hexane/EtOAc, R_f_ = 0.66); IR υmax (cm^−1^): 3039, 2924, 1663, 1602, 1459, 1382, 1251, 1169, 1094; ^1^H NMR (400 MHz, CDCl_3_): δ_H_ 7.98 (*d*, *J* = 8.8 Hz, 1H; H-5); 7.85 (*t*, *J* = 2.0 Hz, 1H; H-7″); 7.48–7.39 (*m*, 3H; H-2″, H-6″; H-4″); 7.37–7.33 (*m*, 4H; H-3′; H-5′; H-3″; H-5″); 7.31–7.29 (*m*, 2H; H-2′; H-6′); 6.69 (*dd*, *J* = 8.8, 2.4 Hz, 1H; H-6); 6.46 (*d*, *J* = 2.3 Hz, 1H; H-8); 5.33 (*d*, *J* = 1.8 Hz, 2H; H-2); 5.02 (*s*, 2H; H-7′). ^13^C NMR-APT (100 MHz, CDCl_3_): δ_C_ 181.11; 164.95; 163.16; 137.08; 134.66; 134.56; 134.31; 130.91; 130.04; 130.00; 129.45; 129.06; 128.94; 128.84; 116.24; 111.05; 101.97; 69.64; 68.03. MALDI-TOF (*m*/*z*): C_23_H_17_O_3_Cl, theoretical value calculated for [M + H]^+^ = 377.0939, found [M + H]^+^ = 377.0937.

(*E*)-3-Benzylidene-7-((3,5-dimethylbenzyl)oxy)chroman-4-one (**17**): Yellow solid. Yield: 42% (0.15 mmol; 55.4 mg); M.P.: 134.2–135.1 °C; TLC (8:2 hexane/EtOAc, R_f_ = 0.76); IR υmax (cm^−1^): 3010, 2920, 1669, 1602, 1437, 1382, 1256, 1160, 1103; ^1^H NMR (400 MHz, CDCl_3_): δ_H_ 7.98 (*d*, *J* = 8.8 Hz, 1H; H-5); 7.86 (*t*, *J* = 2.0 Hz, 1H; H-7″); 7.48–7.39 (*m*, 3H; H-2″; H-6″; H-4″); 7.31–7.29 (*m*, 2H; H-3″; H-5″); 7.03 (*s*, 2H; H-2′; H6′); 6.99 (*s*, 1H; H-4′); 6.72 (*dd*, *J* = 8.9, 2.4 Hz, 1H; H-6); 6.36 (*d*, *J* = 2.3 Hz, 1H; H-8); 5.33 (*d*, *J* = 2.0 Hz, 2H; H-2); 5.02 (*s*, 2H; H-7′); 2.34 (*s*, 6H; H-8′; H-9′). ^13^C NMR-APT (100 MHz, CDCl_3_): δ_C_ 181.12; 165.41; 163.17; 138.47; 136.89; 135.84; 134.70; 131.02; 130.12; 130.02; 129.87; 129.38; 128.81; 125.55; 116.00; 111.20; 101.87; 70.60; 67.97; 21.41. MALDI-TOF (*m*/*z*): C_25_H_22_O_3_, theoretical value calculated for [M + H]^+^ = 371.1642, found [M + H]^+^ = 371.1642.

(*E*)-3-Benzylidene-7-((4-((trifluoromethyl)thio)benzyl)oxy)chroman-4-one (**18**): White solid. Yield: 61% (0.17 mmol; 76.2 mg); M.P.: 141.6–142.6 °C; TLC (8:2 hexane/EtOAc, R_f_ = 0.66); IR υmax (cm^−1^): 2924, 1660, 1603, 1462, 1384, 1252, 1166, 1119; ^1^H NMR (500 MHz, CDCl_3_): δ_H_ 8.00 (*d*, *J* = 8.9 Hz, 1H; H-5); 7.86 (*t*, *J* = 2.1 Hz, 1H; H-7″); 7.69 (*d*, *J* = 8.2 Hz, 2H; H-3′; H-5′); 7.48 (*d*, *J* = 8.5 Hz, 2H; H-2′; H-6′); 7.47–7.40 (*m*, 3H; H2″; H-6″; H-4″); 7.31–7.28 (*m*, 2H; H-3″; H-5″); 6.71 (*dd*, *J* = 8.8, 2.4 Hz, 1H; H-6); 6.47 (*d*, *J* = 2.4 Hz, 1H; H-8); 5.34 (*d*, *J* = 1.9 Hz, 2H; H-2); 5.14 (*s*, 2H; H-7′). ^13^C NMR-APT (125 MHz, CDCl_3_): δ_C_ 180.99; 164.67; 163.04; 139.10; 137.03; 136.61; 134.51; 130.73; 129.95; 129.92; 129.35; 128.72; 128.17; 116.24; 110.83; 101.86; 69.34; 67.92. MALDI-TOF (*m*/*z*): C_24_H_17_F_3_O_3_S, theoretical value calculated for [M + H]^+^ = 443.0923, found [M + H]^+^ = 443.0923.

(*E*)-3-Benzylidene-7-((3-(trifluoromethyl)benzyl)oxy)chroman-4-one (**19**): White solid. Yield: 60% (0.19 mmol, 76.0 mg); M.P.: 135.6–136.4 °C; TLC (8:2 hexane/EtOAc, R_f_ = 0.64); IR υmax (cm^−1^): 2925, 1663, 1605, 1443, 1384, 1330, 1254, 1166, 1118; ^1^H NMR (500 MHz, DMSO-d_6_): δ_H_ 7.85–7.83 (*m*, 2H; H-5; H-2′); 7.78–7.76 (*m*, 1H; H-4′); 7.73–7.72 (*m*, 2H; H-7″; H-6′); 7.66 (*t*, *J* = 7.8 Hz, 1H; H-5′); 7.52–7.44 (*m*, 5H; H-2″; H-3″; H-4″; H-5″; H-6″); 6.80 (*dd*, *J* = 6.8, 2.4 Hz, 1H; H-6); 6.69 (*d*, *J* = 2.4 Hz, 1H; H-8); 5.41 (*d*, *J* = 2.0 Hz, 2H; H-2); 5.31 (*s*, 2H; H-7′). ^13^C NMR-APT (125 MHz, DMSO-d_6_): δ_C_ 179.81; 164.49; 162.60; 137.78; 135.91; 133.91; 131.86; 130.69; 130.23; 129.72; 129.58; 129.20; 128.81; 124.81 (*q*, ^3^*J*_C,F_ = 3.6 Hz; C-2′); 124.26 (*q*, ^3^*J*_C,F_ = 3.9 Hz; C-4′); 115.43; 111.09; 101.94; 68.90; 67.64. MALDI-TOF (*m*/*z*): C_24_H_17_F_3_O_3_, theoretical value calculated for [M + H]^+^ = 411.1202, found [M + H]^+^ = 411.1201.

(*E*)-3-(3-Methoxybenzylidene)-7-propoxychroman-4-one (**20**): Yellow oil. Yield: 32% (0.16 mmol; 51.0 mg); TLC (8:2 hexane/EtOAc, R_f_ = 0.66); IR υmax (cm^−1^): 2927, 1601, 1438, 1384, 1242, 1161, 1104; ^1^H NMR (500 MHz, CDCl_3_): δ_H_ 7.95 (*d*, *J* = 8.8 Hz, 1H; H-5); 7.80 (*t*, *J* = 2.1 Hz, 1H; H-7″); 7.34 (*t*, *J* = 7.9 Hz, 1H; H-5″); 6.93 (*dd*, *J* = 8.3, 2.6 Hz, 1H; H-6″); 6.88–6.85 (*m*, 1H; H-2″); 6.83–6.82 (*m*, 1H; H-4″); 6.62 (*dd*, *J* = 8.8, 2.3 Hz, 1H; H-6); 6.38 (*d*, *J* = 2.3 Hz, 1H; H-8); 5.32 (*d*, *J* = 1.9 Hz, 2H; H-2); 3.95 (*t*, *J* = 6.6 Hz, 2H; H-1′); 3.83 (*s*, 3H; 3″-OCH_3_); 1.82 (*sext*, *J* = 7.0 Hz, 2H; H-2′); 1.03 (*t*, *J* = 7.4 Hz, 3H; H-3′). ^13^C NMR-APT (125 MHz, CDCl_3_): δ_C_ 181.02; 165.83; 163.28; 159.81; 136.63; 136.06; 131.34; 129.81; 129.79; 122.32; 115.72; 115.49; 114.94; 110.99; 101.38; 70.08; 68.02; 55.46; 22.48; 10.54. MALDI-TOF (*m*/*z*): C_20_H_20_O_4_ theoretical value calculated for [M + H]^+^ = 325.1434, found [M + H]^+^ = 325.1433.

(*E*)-3-(3-Methoxybenzylidene)-7-pentyloxychroman-4-one (**21**): Yellow oil. Yield: 25% (0.10 mmol; 37.1 mg); TLC (8:2 hexane/EtOAc, R_f_ = 0.74); IR υmax (cm^−1^): 2929, 1668, 1600, 1434, 1380, 1244, 1159, 1103;^1^H NMR (400 MHz, CDCl_3_): δ_H_ 7.95 (*d*, *J* = 8.8 Hz, 1H; H-5); 7.80 (*t*, *J* = 2.0 Hz, 1H; H-7″); 7.35 (*t*, *J* = 7.9 Hz, 1H; H-5″); 6.94 (*dd*, *J* = 8.3, 2.6 Hz, 1H; H-6″); 6.87–6.85 (*m*, 1H; H-2″); 6.83–6.82 (*m*, 1H; H-4″); 6.62 (*dd*, *J* = 8.8, 2.4 Hz, 1H; H-6); 6.38 (*d*, *J* = 2.4 Hz, 1H; H-8); 5.32 (*d*, *J* = 1.9 Hz, 2H; H-2); 3.99 (*t*, *J* = 6.6 Hz, 2H; H-1′); 3.84 (*s*, 3H; 3″-OCH_3_); 1.79 (*quint*, *J* = 6.8 Hz, 2H; H-2′); 1.48–1.36 (*m*, 4H; H-3′; H-4′); 0.93 (*t*, *J* = 7.1 Hz, 3H; H-5′). ^13^C NMR-APT (100 MHz, CDCl_3_): δ_C_ 181.06; 165.85; 163.29; 159.81; 136.66; 136.06; 131.35; 129.82; 129.81; 122.34; 115.69; 115.49; 114.95; 111.02; 101.36; 68.63; 68.03; 55.47; 28.82; 28.23; 22.53; 14.11. MALDI-TOF (*m*/*z*): C_22_H_24_O_4_, theoretical value calculated for [M + H]^+^ = 353.1747, found [M + H]^+^ = 353.1747.

(*E*)-3-(3-Methoxybenzylidene)-7-((4-bromobenzyl)oxy)chroman-4-one (**22**): White solid. Yield: 11% (0.03 mmol; 15.0 mg); M.P.: 133.7–134.5 °C; TLC (8:2 hexane/EtOAc, R_f_ = 0.56); IR υmax (cm^−1^): 3007, 2925, 1669, 1601, 1435, 1376, 1244, 1160, 1106; ^1^H NMR (500 MHz, CDCl_3_): δ_H_ 7.98 (*d*, *J* = 8.9 Hz, 1H; H-5); 7.81 (*t*, *J* = 2.1 Hz, 1H; H-7″); 7.54–7.50 (*m*, 2H; H-3′; H-5′); 7.35 (*t*, *J* = 7.9 Hz, 1H; H-5″); 7.30–7.28 (*m*, 2H; H-2′; H-6′); 6.95 (*dd*, *J* = 8.0, 2.8 Hz, 1H; H-6″); 6.87–6.86 (*m*, 1H; H-2″); 6.82 (*m*, 1H; H-4″); 6.69 (*dd*, *J* = 8.8, 2.4 Hz, 1H; H-6); 6.45 (*d*, *J* = 2.4 Hz, 1H; H-8); 5.33 (*d*, *J* = 2.0 Hz, 2H; H-2); 5.05 (*s*, 2H; H-7′); 3.84 (*s*, 3H; 3″-OCH_3_). ^13^C NMR-APT (125 MHz, CDCl_3_): δ_C_ 181.06; 164.94; 163.19; 159.81; 136.96; 135.96; 135.08; 132.03; 131.14; 130.00; 129.86; 129.22; 124.69; 122.35; 119.93; 116.23; 115.52; 115.02; 111.06; 101.97; 69.66; 68.08; 55.49. MALDI-TOF (*m*/*z*): C_24_H_19_O_4_Br, theoretical value calculated for [M + H]^+^ = 451.0539, found [M + H]^+^ = 451.0542.

(*E*)-3-(3-Methoxybenzylidene)-7-((4-chlorobenzyl)oxy)chroman-4-one (**23**): White solid. Yield: 37% (0.13 mmol; 52.4 mg); M.P.: 130.8–131.7 °C; TLC (8:2 hexane/EtOAc, R_f_ = 0.54); IR υmax (cm^−1^): 3001, 2925, 1663, 1660, 1460, 1380, 1243, 1165, 1094; ^1^H NMR (500 MHz, CDCl_3_): ^1^H NMR (500 MHz, CDCl_3_): δ_H_ 7.98 (*d*, *J* = 8.9 Hz, 1H; H-5); 7.81 (*t*, *J* = 2.1 Hz, 1H; H-7″); 7.38–7.33 (*m*, 5H; H-5″; H-2′; H-3′; H-5′; H-6′); 6.95 (*dd*, *J* = 8.3, 2.7 Hz, 1H; H-6″); 6.87–6.86 (*m*, 1H; H-2″); 6.83–6.82 (*m*, 1H; H-4″); 6.69 (*dd*, *J* = 8.9, 2.4 Hz, 1H; H-6); 6.45 (*d*, *J* = 2.4 Hz, 1H; H-8); 5.33 (*d*, *J* = 2.0 Hz, 2H; H-2); 5.06 (*s*, 2H; H-7′); 3.84 (*s*, 3H; 3″-OCH_3_). ^13^C NMR-APT (125 MHz, CDCl_3_): δ_C_ 181.04; 164.97; 163.20; 159.84; 136.93; 135.99; 134.58; 134.32; 131.16; 130.00; 129.86; 129.06; 128.94; 122.35; 116.25; 115.53; 115.03; 111.06; 101.99; 69.65; 68.09; 55.49. MALDI-TOF (*m*/*z*): C_24_H_19_O_4_Cl, theoretical value calculated for [M + H]^+^ = 407.1044, found [M + H]^+^ = 407.1045.

(*E*)-3-(3-Methoxybenzylidene)-7-((4-((trifluoromethyl)thio)benzyl)oxy)chroman-4-one (**24**): White solid. Yield: 56% (0.16 mmol; 74.4 mg); M.P.: 132.5–133.5 °C; TLC (8:2 hexane/EtOAc, R_f_ = 0.58); IR υmax (cm^−1^): 3017, 2926, 1665, 1602, 1460, 1381, 1243, 1160, 1115; ^1^H NMR (500 MHz, CDCl_3_): δ_H_ 7.99 (*d*, *J* = 8.7 Hz, 1H; H-5); 7.81 (*brs*, 1H; H-7″); 7.69 (*d*, *J* = 8.2 Hz, 2H; H-3′; H-5′); 7.48 (*d*, *J* = 8.2 Hz, 2H; H-2′; H-6′); 7.35 (*t*, *J* = 7.9 Hz, 1H; H-5″); 6.95 (*dd*, *J* = 8.3, 2.8 Hz, 1H; H-6″); 6.87–6.86 (*m*, 1H; H-2″); 6.83–6.82 (*m*, 1H; H-4″); 6.71 (*dd*, *J* = 8.9, 2.6 Hz, 1H; H-6); 6.47 (*d*, *J* = 2.6 Hz, 1H; H-8); 5.33 (*s*, 2H; H-2); 5.13 (*s*, 2H; H-7′); 3.84 (*s*, 3H; 3″-OCH_3_). ^13^C NMR-APT (125 MHz, CDCl_3_): δ_C_ 181.03; 164.82; 163.21; 159.85; 139.24; 137.01; 136.72; 135.96; 131.11; 130.07; 129.87; 128.33; 122.36; 116.37; 115.55; 115.04; 110.97; 102.01; 69.47; 68.10; 55.49. MALDI-TOF (*m*/*z*): C_25_H_19_F_3_O_4_S, theoretical value calculated for [M + H]^+^ = 473.1029, found [M + H]^+^ = 473.1028.

(*E*)-3-(3-Methoxybenzylidene)-7-((3-(trifluoromethyl)benzyl)oxy)chroman-4-one (**25**): White solid. Yield: 35% (0.11 mmol; 47.4 mg); M.P.: 110.2–111.1 °C; TLC (8:2 hexane/EtOAc, R_f_ = 0.60); IR υmax (cm^−1^): 3004, 2925, 1663, 1602, 1460, 1381, 1243, 1161, 1118; ^1^H NMR (500 MHz, CDCl_3_): ^1^H NMR (500 MHz, CDCl_3_): δ_H_ 7.99 (*d*, *J* = 8.9 Hz, 1H; H-5); 7.82 (*t*, *J* = 2.0 Hz, 1H; H-7″); 7.69 (*s*, 1H; H-2′); 7.61 (*d*, *J* = 7.6 Hz, 2H; H-4′; H-6′); 7.55 (*t*, *J* = 7.5 Hz, 1H; H-5′); 7.35 (*t*, *J* = 7.5 Hz, 1H; H-5″); 6.95 (*dd*, *J* = 8.3, 3.1 Hz, 1H; H-6″); 6.87–6.86 (*m*, 1H; H-2″); 6.84–6.82 (*m*, 1H; H-4″); 6.72 (*dd*, *J* = 8.9, 2.6 Hz, 1H; H-6); 6.48 (*d*, *J* = 2.6 Hz, 1H; H-8,); 5.34 (*d*, *J* = 2.0 Hz, 2H; H-2); 5.14 (*s*, 2H; H-7′); 3.84 (*s*, 3H; 3″-OCH^3^). ^13^C NMR-APT (125 MHz, CDCl_3_): δ_C_ 181.05; 164.82; 163.23; 159.86; 137.14; 137.01; 135.98; 131.14; 130.76; 130.08; 129.87; 129.36; 125.26 (*q*, ^3^*J*_C,F_ = 3.7 Hz; C-2′); 124.26 (*q*, ^3^*J*_C,F_ = 3.7 Hz; C-4′); 122.36; 116.40; 115.54; 115.05; 110.97; 102.01; 69.59; 68.11; 55.49. MALDI-TOF (*m*/*z*): C_25_H_19_F_3_O_4_, theoretical value calculated for [M + H]^+^ = 441.1308, found [M + H]^+^ = 441.1307.

### 4.2. Biological Evaluation

#### 4.2.1. In Vitro Antimicrobial Assay

The antimicrobial activity of the compounds was evaluated against strains of *S. epidermidis* ATCC-12228, *P. aeruginosa* ATCC-25853, *S. enteritidis* ATCC-6017, *C. albicans* ATCC-76445, *C. albicans* LM-92, *C. tropicalis* ATCC-13803, *N. glabratus* ATCC-90030, *A. flavus* ATCC-13013, and *P. citrinum* ATCC-4001. The strains were part of the microorganism collection of the Mycology Laboratory at the Department of Pharmaceutical Sciences (DCF), Federal University of Paraíba. Fungal strains were maintained on Sabouraud dextrose agar (SDA), while bacterial strains were kept in brain heart infusion broth (BHI), both at 4 °C. The strains were used for assays after 24–48 h of incubation at 35 ± 2 °C in SDA/BHI. Microbial colonies were suspended in a 0.9% sterile saline solution and adjusted according to tube 0.5 of the McFarland standard scale to obtain 10^6^ CFU/mL [65,66,67].

#### 4.2.2. Determination of the Minimum Inhibitory Concentration (MIC), Minimum Fungicidal Concentration (MFC), and Minimum Bactericidal Concentration (MBC)

The MIC determination of the samples against bacterial and fungal strains was performed using the broth microdilution technique in a 96-well U-shaped cell culture plate (TPP, Switzerland/Europe). Initially, 100 μL of doubly concentrated Roswell Park Memorial Institute (RPMI 1640) broth was distributed into the wells of the microdilution plates. Then, 100 μL of solubilized compound was added to the wells of the first row of the plate. The products were weighed to an initial concentration of 1024 µg/mL and duly solubilized in 150 µL (3%) of dimethyl sulfoxide (DMSO) and 100 µL (2%) of Tween 80. Through a serial dilution at a 1:2 ratio, concentrations ranging from 1024 to 4 µg/mL were obtained. Finally, 10 µL of microorganism suspensions were added to the wells, with each column of the plate corresponding to a specific species. Controls were conducted in parallel, including microorganisms (RPMI + yeasts) to confirm strain viability, culture medium (RPMI) to ensure sterility, and amphotericin B as a control for fungal inhibition. The plates were aseptically sealed and incubated at 35 ± 2 °C for 24–48 h for yeast and bacteria assays and at 28–30 °C for 5–7 days for filamentous fungi. A compound was considered active if it inhibited at least 50% of the microorganisms used in the biological activity assays [65,68].

After MIC determination, MFC and MBC assays were performed. Aliquots of 10 μL from the wells where complete microbial growth inhibition was observed (MIC, MICx2, and MICx4) were added to 100 μL of RPMI broth in new 96-well microdilution plates and incubated for 24–48 h at 35 ± 2 °C for yeasts/bacteria and at 28–30 °C for 5–7 days for filamentous fungi. MFC was defined as the lowest concentration of the test compound capable of inhibiting microbial growth. A solution with 3% of dimethyl sulfoxide (DMSO) and 2% of Tween 80 was used as a control. Gentamicin (128 or 256 μg/mL) and fluconazole (128 or 256 μg/mL) were used as positive controls [69,70]. Additionally, sterility control (RPMI 1640 broth) and viability control for microorganism species (RPMI 1640 plus inoculum of each microorganism) were performed. The mode of action of the test substance was also evaluated using the MFC/MIC ratio to specify the nature of the antimicrobial effect [68]. The compound was considered fungicidal when the MFC/MIC ratio was between 1:1 and 2:1; if the ratio exceeded 2:1, the mode of action was more likely fungistatic.

The assays were conducted in triplicate, and the result was expressed as the arithmetic mean of the minimum inhibitory concentration (MIC), minimum fungicidal concentration (MFC), and minimum bactericidal concentration (MBC) values obtained in the three assays.

### 4.3. Molecular Modeling Study

#### 4.3.1. Target Selection

To identify the potential molecular targets of compounds **1**, **2**, and **21**, a homology-based target fishing strategy was employed as previously described [71]. The Similarity Ensemble Approach (SEA) web server [72] was initially used to generate a list of candidate targets based on chemical similarity. These targets were then queried using BLAST 2.16.0 (https://blast.ncbi.nlm.nih.gov/Blast.cgi, accessed on 21 April 2025) [73] against the *C. albicans* proteome (taxid: 5476) available in the RefSeq Protein database. The BLAST searches were conducted using the NCBI server (https://blast.ncbi.nlm.nih.gov/). Proteins from the fungus were considered plausible targets when they shared at least 35% sequence identity and 75% alignment coverage with the SEA-derived proteins.

#### 4.3.2. Molecular Docking

One three-dimensional conformer was generated for each compound using OpenEye Omega 6.0.0.1 (https://www.eyesopen.com/omega, accessed on 28 April 2025) [74,75], and partial atomic charges were assigned via the AM1-BCC method using MolCharge 2.2.5.1 (https://www.eyesopen.com/quacpac, accessed on 28 April 2025) [76]. Since experimental structure was only available for FBA1, the AlphaFold [77,78] models were selected for modeling the rest of the receptors. Metal ions and cofactors were added to the receptors from homologous proteins identified through template searches performed with the Swiss-Model 3.5 web server (https://swissmodel.expasy.org/, accessed on 23 April 2025) [79].

Docking calculations were carried out with Gold [80] as previously described [81]. A total of 30 docking solutions were generated per compound. Primary scoring was performed with the ChemPLP scoring function and all poses were rescored with the ChemScore, GoldScore, and ASP scoring functions. Docking scores were scaled to Z-scores at a compound-target pair level. Any docking solution with a Z-score larger than 1.0 was selected for additional studies.

#### 4.3.3. Molecular Dynamics Simulations and Free Energies of Binding

Molecular dynamics (MD) simulations and MM-PBSA free energy calculations were conducted using the AMBER 24 software suite [82], following protocols previously applied [83]. Each ligand–target complex underwent a full MD simulation workflow, including system preparation, energy minimization, thermal equilibration, and production dynamics. Protein residues were parameterized using the ff19SB force field, while ligand parameters were assigned via GAFF2. Complexes were solvated in truncated octahedral TIP3P water boxes, and Na^+^ or Cl^−^ ions were added to neutralize charge and achieve a final salt concentration of 0.15 M according to the previously reported protocol [84]. For metalloproteins, parameters were obtained via the MCPB method as implemented in the MCPB.py package of AMBER [85], while parameters for cofactors were retrieved from the repository maintained by the Bryce group at the University of Manchester (http://amber.manchester.ac.uk/, accessed on 23 April 2025).

Energy minimization proceeded in two stages. The first stage applied 500 steps of steepest descent followed by 500 cycles of conjugate gradient, with positional restraints (500 kcal/mol·Å^2^) applied to all solute atoms. In the second stage, restraints were removed, and minimization was repeated with 500 steps of steepest descent and 1000 cycles of conjugate gradient. Electrostatics were treated with the Particle Mesh Ewald (PME) method using a 12 Å cutoff. Following minimization, systems were heated from 0 to 300 K over 20 ps under constant volume conditions, with solute atoms restrained using a 10 kcal/mol·Å^2^ force constant. A Langevin thermostat (collision frequency 1.0 ps^−1^) regulated temperature, and PME was applied with a 10 Å cutoff. Hydrogen-containing bonds were constrained using SHAKE, allowing for longer integration time steps.

After heating, equilibration was performed at 300 K and 1 atm for 100 ps using constant pressure and temperature (NPT ensemble). Pressure was maintained via isotropic position scaling (relaxation time 2 ps), and the PME cutoff remained at 10 Å. Subsequently, five independent 4 ns production runs were performed using the same parameters, with random velocity initialization at the start of each run for a better exploration of the complexes’ conformational space.

Binding free energies were computed using the MM-PBSA approach implemented in Amber’s MM-PBSA.py script [86]. From the combined production trajectories, 100 snapshots were extracted (one every 40 ps) for energy evaluation from the 1 ns–4 ns time interval. A salt concentration of 150 mM was applied in MM-PBSA calculations to reflect physiological conditions.

## 5. Conclusions

This study investigated the antimicrobial activity of 25 compounds derived from chroman-4-ones and homoisoflavonoids, 17 of which are novel compounds. Thirteen exhibited bioactivity against microorganisms of human importance, including Gram-positive and Gram-negative bacteria as well as fungi. Compounds **1**, **2**, and **21** stood out, as they exhibited higher potency than the positive controls (gentamicin and fluconazole) against certain microorganisms, with MIC values ranging from 64 to 256 μg/mL (359.2–1559.5 μM) against bacteria and yeasts and from 256 to 512 μg/mL (1452.8–2873.6 μM) against filamentous fungi. Molecular modeling suggested that compounds **1**, **2**, and **21** act via distinct targets in *Candida albicans*: **1** inhibits cysteine synthase, while **2** and **21** target HOG1 kinase and FBA1, key proteins in fungal virulence and survival. Our findings suggest that the addition of alkyl or aryl carbon chains to the hydroxyl group at position 7 reduces the antimicrobial activity of chroman-4-ones. In addition, introducing an activating group (methoxy) at the *meta* position of ring B in homoisoflavonoids significantly enhanced antimicrobial activity. This preliminary study revealed structural characteristics of these compounds that are important for antibacterial activity, which may contribute to the development of new bioactive agents against microorganisms. Further studies will be conducted, including toxicological evaluation and other bioactivities, to advance the investigation of the therapeutic potential of these chemical classes.

## Figures and Tables

**Table 1:** Minimum inhibitory concentration (MIC) of compounds **1**–**25** against bacteria. The results are expressed in μg/mL (µM).

Compound	*S. epidermidis* ATCC-12228	*P. aeruginosa* ATCC-25853	*S. enteritidis* ATCC-6017	MBC
**1**	128 (779.7)	128 (779.7)	256 (1559.5)	512 (3119.0)
**2**	128 (718.40)	128 (718.40)	256 (1436.8)	512 (2873.6)
**3**	256 (1241.3)	512 (2482.6)	512 (2482.6)	1024 (4965.2)
**4**	+	+	+	X
**5**	+	+	+	X
**6**	+	+	+	X
**7**	+	+	+	X
**8**	256 (906.7)	256 (906.7)	256 (906.7)	512 (1813.4)
**9**	+	+	+	X
**10**	512 (1588.7)	512 (1588.7)	512 (1588.7)	X
**11**	512 (1922.7)	512 (1922.7)	512 (1922.7)	X
**12**	256 (869.7)	256 (869.7)	256 (869.7)	1024 (3478.8)
**13**	1024 (3478.8)	1024 (3478.8)	1024 (3478.8)	X
**14**	512 (1588.1)	512 (1588.1)	512 (1588.1)	X
**15**	+	+	+	X
**16**	+	+	+	X
**17**	+	+	+	X
**18**	512 (1161.7)	512 (1161.7)	512 (1161.7)	X
**19**	+	+	+	X
**20**	128 (394.6)	256 (789.2)	256 (789.2)	512 (1578.4)
**21**	128 (363.2)	128 (363.2)	128 (363.2)	512 (1452.8)
**22**	256 (567.2)	512 (1134.4)	512 (1134.4)	512 (1134.4)
**23**	+	+	+	X
**24**	+	+	+	X
**25**	+	+	+	X
Control: Culture medium	-	-	-	X
Control: Microorganism *	+	+	+	X
Control: Gentamicin	128 (268.0)	256 (536.0)	256 (536.0)	X

(+): Growth of the microorganism; (X): not applicable. * 3% of DMSO and 2% of Tween 80.

**Table 2:** Minimum inhibitory concentration (MIC) of compounds **1**–**25** against fungi. The results are expressed in μg/mL (µM).

Compound	*C. albicans* ATCC-76445	*C. albicans* LM-92	*C. tropicalis* ATCC-13803	*N. glabratus* ATCC-90030	*A. flavus* ATCC-13013	*P. citrinum* ATCC-4001	MFC
**1**	64 (389.9)	64 (389.9)	64 (389.9)	64 (389.9)	256 (1559.5)	256 (1559.5)	256 (1559.5)
**2**	64 (359.2)	64 (359.2)	64 (359.2)	64 (359.2)	512 (2873.6)	512 (2873.6)	256 (1436.8)
**3**	128 (620.6)	128 (620.6)	256 (1241.3)	256 (1241.3)	512 (2482.6)	512 (2482.6)	512 (2482.6)
**4**	+	+	+	+	+	+	X
**5**	+	+	+	+	+	+	X
**6**	+	+	+	+	+	+	X
**7**	+	+	+	+	+	+	X
**8**	256 (906.7)	256 (906.7)	256 (906.7)	256 (906.7)	512 (1813.4)	512 (1813.4)	512 (1813.4)
**9**	+	+	+	+	+	+	X
**10**	512 (1588.7)	512 (1588.7)	512 (1588.7)	512 (1588.7)	1024 (3177.4)	1024 (3177.4)	X
**11**	512 (1922.7)	512 (1922.7)	512 (1922.7)	512 (1922.7)	1024 (3845.4)	1024 (3845.4)	X
**12**	256 (869.7)	256 (869.7)	256 (869.7)	256 (869.7)	1024 (3478.8)	1024 (3478.8)	1024 (3478.8)
**13**	1024 (3478.8)	1024 (3478.8)	1024 (3478.8)	1024 (3478.8)	1024 (3478.8)	1024 (3478.8)	X
**14**	512 (1588.1)	512 (1588.1)	512 (1588.1)	512 (1588.1)	1024 (3176.2)	1024 (3176.2)	X
**15**	+	+	+	+	+	+	X
**16**	+	+	+	+	+	+	X
**17**	+	+	+	+	+	+	X
**18**	512 (1161.7)	512 (1161.7)	512 (1161.7)	512 (1161.7)	512 (1161.7)	512 (1161.7)	X
**19**	+	+	+	+	+	+	X
**20**	256 (789.2)	256 (789.2)	256 (789.2)	256 (789.2)	512 (1578.4)	512 (1578.4)	512 (1578.4)
**21**	128 (363.2)	128 (363.2)	128 (363.2)	128 (363.2)	512 (1452.8)	512 (1452.8)	512 (1452.8)
**22**	256 (567.2)	256 (567.2)	256 (567.2)	256 (567.2)	512 (1134.4)	512 (1134.4)	512 (1134.4)
**23**	+	+	+	+	+	+	X
**24**	+	+	+	+	+	+	X
**25**	+	+	+	+	+	+	X
Control: Culture medium	-	-	-	-	-	-	X
Control: Microorganism *	+	+	+	+	+	+	X
Control: Fluconazole	128 (417.9)	128 (417.9)	128 (417.9)	128 (417.9)	256 (835.8)	256 (835.8)	X

(+): Growth of the microorganism; (X): not applicable. * 3% of DMSO and 2% of Tween 80. The biological evaluation revealed that 13 compounds exhibited antimicrobial activity. In the antibacterial assays, compounds **1**, **2**, **8**, **12**, **20**, and **21** showed moderate activity against the tested bacterial strains. Notably, compound **21** stood out by demonstrating greater potency than the positive control (gentamicin) against Gram-negative bacteria strains, as indicated by lower MIC values (in µM), whereas against the Gram-positive bacterium *S. epidermidis*, the positive control exhibited greater potency. The remaining compounds showed weak or no antibacterial activity.

**Table 3:** Potential targets of compounds **1**, **2**, and **21** in *C. albicans*.

UniProt Accession	Description	ID	Compound ^(a)^
A0A1D8PE91	Tyrosine protein phosphatase	LTP1	**1**
A0A1D8PP00	Arginase	CAR1	**1**
O42766	14-3-3 protein	BMH1	**1**
A0A1D8PI24	D-arabinose 1-dehydrogenase	ARA1	**2**, **21**
A0A1D8PNK3	D-xylose reductase	GRE3	**2**, **21**
A0A1D8PQ13	Cysteine synthase	CS	**2**
C4YT51	Cysteine synthase	CS1	**2**
P78599	Ornithine decarboxylase	ORD1	**2**
Q59T95	Cystathionine beta-synthase	CSY4	**2**
Q59U59	Proteinase A	APR1	**2**
Q5ADT3	Glycerol dehydrogenase	GCY	**2**, **21**
Q5ADT4	Glycerol 2-dehydrogenase	GCY1	**2**, **21**
Q9URB4	Fructose-bisphosphate aldolase	FBA1	**2**
A0A1D8PT38	Mitogen-activated protein kinase	CEK2	**21**
P43063	Cyclin-dependent kinase 1	CDK1	**21**
Q5A1D3	Extracellular signal-regulated kinase 1	ERK1	**21**
Q5AAG6	Mitogen-activated protein kinase	MKC1	**21**
Q92207	Mitogen-activated protein kinase	HOG1	**21**
Q9B8D6	NADH-ubiquinone oxidoreductase	NAD1	**21**

^(a)^ Compound for which the protein is predicted as a potential target.

## Data Availability

All data generated or analyzed in this study are included in this article.

## Supplementary Materials

The following supporting information can be downloaded at: https://www.mdpi.com/article/10.3390/molecules30173575/s1, Figure S1: ^1^H NMR spectrum (500 MHz, DMSO-d_6_) of compound **1**; Figure S2: ^13^C NMR-APT spectrum (125 MHz, DMSO-d_6_) of compound **1**; Figure S3: Infrared spectrum ʋmax of compound **1**; Figure S4: ^1^H NMR spectrum (400 MHz, DMSO-d_6_) of compound **2**; Figure S5: ^13^C NMR-APT spectrum (100 MHz, DMSO-d_6_) of compound **2**; Figure S6: Infrared spectrum ʋmax of compound **2**; Figure S7: ^1^H NMR spectrum (400 MHz, DMSO-d_6_) of compound **3**; Figure S8: ^13^C NMR-APT spectrum (100 MHz, DMSO-d_6_) of compound **3**; Figure S9: Infrared spectrum ʋmax of compound **3**; Figure S10: ^1^H NMR spectrum (500 MHz, CDCl_3_) of compound **4**; Figure S11: ^13^C NMR-APT spectrum (125 MHz, CDCl_3_) of compound **4**; Figure S12: Infrared spectrum ʋmax of compound **4**; Figure S13: ^1^H NMR spectrum (500 MHz, CDCl_3_) of compound **5**; Figure S14: ^13^C NMR-APT spectrum (125 MHz, CDCl_3_) of compound **5**; Figure S15: Infrared spectrum ʋmax of compound **5**; Figure S16: HRMS spectrum of compound **5**; Figure S17: ^1^H NMR spectrum (400 MHz, DMSO-d_6_) of compound **6**; Figure S18: ^13^C NMR-APT spectrum (100 MHz, DMSO-d_6_) of compound **6**; Figure S19: Infrared spectrum ʋmax of compound **6**; Figure S20: ^1^H NMR spectrum (400 MHz, DMSO-d_6_) of compound **7**; Figure S21: ^13^C NMR-APT spectrum (100 MHz, DMSO-d_6_) of compound **7**; Figure S22: Infrared spectrum ʋmax of compound **7**; Figure S23: ^1^H NMR spectrum (500 MHz, DMSO-d_6_) of compound **8**; Figure S24: ^13^C NMR-APT spectrum (125 MHz, DMSO-d_6_) of compound **8**; Figure S25: Infrared spectrum ʋmax of compound **8**; Figure S26: HRMS spectrum of compound **8**; Figure S27: ^1^H NMR spectrum (500 MHz, DMSO-d_6_) of compound **9**; Figure S28: ^13^C NMR-APT spectrum (125 MHz, DMSO-d_6_) of compound **9**; Figure S29: Infrared spectrum ʋmax of compound **9**; Figure S30: HRMS spectrum of compound **9**; Figure S31: ^1^H NMR spectrum (500 MHz, DMSO-d_6_) of compound **10**; Figure S32: ^13^C NMR-APT spectrum (125 MHz, DMSO-d_6_) of compound **10**; Figure S33: Infrared spectrum ʋmax of compound **10**; Figure S34: HRMS spectrum of compound **10**; Figure S35: ^1^H NMR spectrum (400 MHz, CDCl_3_) of compound **11**; Figure S36: ^13^C NMR-APT spectrum (100 MHz, CDCl_3_) of compound **11**; Figure S37: Infrared spectrum ʋmax of compound **11**; Figure S38: ^1^H NMR spectrum (500 MHz, CDCl_3_) of compound **12**; Figure S39: ^13^C NMR-APT spectrum (125 MHz, CDCl_3_) of compound **12**; Figure S40: Infrared spectrum ʋmax of compound **12**; Figure S41: HRMS spectrum of compound **12**; Figure S42: ^1^H NMR spectrum (400 MHz, CDCl_3_) of compound **13**; Figure S43: ^13^C NMR-APT spectrum (100 MHz, CDCl_3_) of compound **13**; Figure S44: Infrared spectrum ʋmax of compound **13**; Figure S45: ^1^H NMR spectrum (400 MHz, CDCl_3_) of compound **14**; Figure S46: ^13^C NMR-APT spectrum (100 MHz, CDCl_3_) of compound **14**; Figure S47: Infrared spectrum ʋmax of compound **14**; Figure S48: HRMS spectrum of compound **14**; Figure S49: ^1^H NMR spectrum (400 MHz, CDCl_3_) of compound **15**; Figure S50: ^13^C NMR-APT spectrum (100 MHz, CDCl_3_) of compound **15**; Figure S51: Infrared spectrum ʋmax of compound **15**; Figure S52: HRMS spectrum of compound **15**; Figure S53: ^1^H NMR spectrum (400 MHz, CDCl_3_) of compound **16**; Figure S54: ^13^C NMR-APT spectrum (100 MHz, CDCl_3_) of compound **16**; Figure S55: Infrared spectrum ʋmax of compound **16**; Figure S56: HRMS spectrum of compound **16**; Figure S57: ^1^H NMR spectrum (400 MHz, CDCl_3_) of compound **17**; Figure S58: ^13^C NMR-APT spectrum (100 MHz, CDCl_3_) of compound **17**; Figure S59: Infrared spectrum ʋmax of compound **17**; Figure S60: HRMS spectrum of compound **17**; Figure S61: ^1^H NMR spectrum (500 MHz, CDCl_3_) of compound **18**; Figure S62: ^13^C NMR-APT spectrum (125 MHz, CDCl_3_) of compound **18**; Figure S63: Infrared spectrum ʋmax of compound **18**; Figure S64: HRMS spectrum of compound **18**; Figure S65: ^1^H NMR spectrum (500 MHz, DMSO-d_6_) of compound **19**; Figure S66: ^13^C NMR-APT spectrum (125 MHz, DMSO-d_6_) of compound **19**; Figure S67: Infrared spectrum ʋmax of compound **19**; Figure S68: HRMS spectrum of compound **19**; Figure S69: ^1^H NMR spectrum (500 MHz, CDCl_3_) of compound **20**; Figure S70: ^13^C NMR-APT spectrum (125 MHz, CDCl_3_) of compound **20**; Figure S71: Infrared spectrum ʋmax of compound **20**; Figure S72: HRMS spectrum of compound **20**; Figure S73: ^1^H NMR spectrum (400 MHz, CDCl_3_) of compound **21**; Figure S74: ^13^C NMR-APT spectrum (100 MHz, CDCl_3_) of compound **21**; Figure S75: Infrared spectrum ʋmax of compound **21**; Figure S76: HRMS spectrum of compound **21**; Figure S77: ^1^H NMR spectrum (500 MHz, CDCl_3_) of compound **22**; Figure S78: ^13^C NMR-APT spectrum (125 MHz, CDCl_3_) of compound **22**; Figure S79: Infrared spectrum ʋmax of compound **22**; Figure S80: HRMS spectrum of compound **22**; Figure S81: ^1^H NMR spectrum (500 MHz, CDCl_3_) of compound **23**; Figure S82: ^13^C NMR-APT spectrum (125 MHz, CDCl_3_) of compound **23**; Figure S83: Infrared spectrum ʋmax of compound **23**; Figure S84: HRMS spectrum of compound **23**; Figure S85: ^1^H NMR spectrum (500 MHz, CDCl_3_) of compound **24**; Figure S86: ^13^C NMR-APT spectrum (125 MHz, CDCl_3_) of compound **24**; Figure S87: Infrared spectrum ʋmax of compound **24**; Figure S88: HRMS spectrum of compound **24**; Figure S89: ^1^H NMR spectrum (500 MHz, CDCl_3_) of compound **25**; Figure S90: ^13^C NMR-APT spectrum (125 MHz, CDCl_3_) of compound **25**; Figure S91: Infrared spectrum ʋmax of compound **25**; Figure S92: HRMS spectrum of compound **25**; Figure S93: RMSD plots for the predicted complex of compound **1** with CS. Separate plots are provided for the compound (top) and protein (bottom). The five MD replicas are labelled R1 to R5; Figure S94: RMSD plots for the predicted complex of compound **2** with HOG1. Separate plots are provided for the compound (top) and protein (bottom). The five MD replicas are labelled R1 to R5; Figure S95: RMSD plots for the predicted complex of compound **21** with HOG1. Separate plots are provided for the compound (top) and protein (bottom). The five MD replicas are labelled R1 to R5; Figure S96: RMSD plots for the predicted complex of compound **2** with FBA1. Separate plots are provided for the compound (top) and protein (bottom). The five MD replicas are labelled R1 to R5; Figure S97: RMSD plots for the predicted complex of compound **21** with FBA1. Separate plots are provided for the compound (top) and protein (bottom). The five MD replicas are labelled R1 to R5; Figure S98: RMSFs of the protein in the complex of compound **1** with CS. One figure is provided per MD replica. The compound is represented as orange spheres; Figure S99: RMSFs of the protein in the complex of compound **2** with HOG1. One figure is provided per MD replica. The compound is represented as orange spheres; Figure S100: RMSFs of the protein in the complex of compound **21** with HOG1. One figure is provided per MD replica. The compound is represented as orange spheres; Figure S101: RMSFs of the protein in the complex of compound **2** with FBA1. One figure is provided per MD replica. The compound is represented as orange spheres; Figure S102: RMSFs of the protein in the complex of compound **21** with FBA1. One figure is provided per MD replica. The compound is represented as orange spheres; Table S1: Docking scores for the selected complexes; Table S2: Results of MM-PBSA calculations; File Complexes-PDBs.zip: Complexes predicted from the molecular docking calculations; repository https://doi.org/10.5281/zenodo.16555424 containing the MD trajectories and topologies employed in this research. References [87,88] are cited in the supplementary materials.
