# Supplementary material for: Synthesis and Antimicrobial Evaluation of Chroman-4-One and Homoisoflavonoid Derivatives
Source: Molecules. 2025 Aug 31;30(17):3575. doi: 10.3390/molecules30173575 (PMC12430254; doi:10.3390/molecules30173575)
Supplement: Supplementary file 1 [file molecules-30-03575-s001.zip › molecules-3774710-supplementary.pdf]

Supplementary Material

Article

Synthesis and Antimicrobial Evaluation of Chroman-4-One and Homoisoflavonoid Derivatives

Carlos d. S. M. Bezerra Filho [1], José L. F. M. Galvão [1], Edeltrudes O. Lima [1], Yunierkis Perez-Castillo [2], Yendrek Velásquez-López [2] and Damião P. de Sousa [1,*]

[1] Department of Pharmaceutical Sciences, Federal University of Paraíba, João Pessoa 58051-900, PB, Brazil; carlosmaia1996@gmail.com (C.d.S.M.B.F.); luksfmgalvao@hotmail.com (J.L.F.M.G.); edeolima@yahoo.com.br (E.O.L.)

[2] Bio-cheminformatics Research Group and Facultad de Ingeniería y Ciencias Aplicadas, Universidad de Las Américas, Quito 170125, Ecuador; yunierkis@gmail.com (Y.P.-C.); yendrek.velasquez.lopez@udla.edu.ec (Y.V.-L.)

* Correspondence: damiao_desousa@yahoo.com.br

Academic Editors: Ivan Kosalec and David Barker

Received: 8 July 2025
Revised: 25 August 2025
Accepted: 26 August 2025
Published: 31 August 2025

Citation: Filho, C.d.S.M.B.; Galvão, J.L.F.M.; Lima, E.O.; Perez-Castillo, Y.; Velásquez-López, Y.; de Sousa, D.P. Synthesis and Antimicrobial Evaluation of Chroman-4-One and Homoisoflavonoid Derivatives. *Molecules* 2025, *30*, 3575. https://doi.org/10.3390/molecules30173575

Copyright: © 2025 by the authors. Licensee MDPI, Basel, Switzerland. This article is an open access article distributed under the terms and conditions of the Creative Commons Attribution (CC BY) license (https://creativecommons.org/licenses/by/4.0/).

Figure S1: ^{1}H NMR spectrum (500 MHz, DMSO-d_6) of compound **1**. 6
Figure S2: ^{13}C NMR-APT spectrum (125 MHz, DMSO-d_6) of compound **1**. 7
Figure S3: Infrared spectrum υmax of compound **1**. 7
Figure S4: ^{1}H NMR spectrum (400 MHz, DMSO-d_6) of compound **2**. 8
Figure S5: ^{13}C NMR-APT spectrum (100 MHz, DMSO-d_6) of compound **2**. 9
Figure S6: Infrared spectrum υmax of compound **2**. 9
Figure S7: ^{1}H NMR spectrum (400 MHz, DMSO-d_6) of compound **3**. 10
Figure S8: ^{13}C NMR-APT spectrum (100 MHz, DMSO-d_6) of compound **3**. 10
Figure S9: Infrared spectrum υmax of compound **3**. 10
Figure S10: ^{1}H NMR spectrum (500 MHz, $CDCl_3$) of compound **4**. 11
Figure S11: ^{13}C NMR-APT spectrum (125 MHz, $CDCl_3$) of compound **4**. 11
Figure S12: Infrared spectrum υmax of compound **4**. 12
Figure S13: ^{1}H NMR spectrum (500 MHz, $CDCl_3$) of compound **5**. 13
Figure S14: ^{13}C NMR-APT spectrum (125 MHz, $CDCl_3$) of compound **5**. 13
Figure S15: Infrared spectrum υmax of compound **5**. 14
Figure S16: HRMS spectrum of compound **5**. 14
Figure S17: ^{1}H NMR spectrum (400 MHz, DMSO-d_6) of compound **6**. 15
Figure S18: ^{13}C NMR-APT spectrum (100 MHz, DMSO-d_6) of compound **6**. 15
Figure S19: Infrared spectrum υmax of compound **6**. 16
Figure S20: ^{1}H NMR spectrum (400 MHz, DMSO-d_6) of compound **7**. 16
Figure S21: ^{13}C NMR-APT spectrum (100 MHz, DMSO-d_6) of compound **7**. 17
Figure S22: Infrared spectrum υmax of compound **7**. 17
Figure S23: ^{1}H NMR spectrum (500 MHz, DMSO-d_6) of compound **8**. 18
Figure S24: ^{13}C NMR-APT spectrum (125 MHz, DMSO-d_6) of compound **8**. 18
Figure S25: Infrared spectrum υmax of compound **8**. 19
Figure S26: HRMS spectrum of compound **8**. 19
Figure S27: ^{1}H NMR spectrum (500 MHz, DMSO-d_6) of compound **9**. 20
Figure S28: ^{13}C NMR-APT spectrum (125 MHz, DMSO-d_6) of compound **9**. 20
Figure S29: Infrared spectrum υmax of compound **9**. 21
Figure S30: HRMS spectrum of compound **9**. 21
Figure S31: ^{1}H NMR spectrum (500 MHz, DMSO-d_6) of compound **10**. 22
Figure S32: ^{13}C NMR-APT spectrum (125 MHz, DMSO-d_6) of compound **10**. 22
Figure S33: Infrared spectrum υmax of compound **10**. 23
Figure S34: HRMS spectrum of compound **10**. 23
Figure S35: ^{1}H NMR spectrum (400 MHz, $CDCl_3$) of compound **11**. 24
Figure S36: ^{13}C NMR-APT spectrum (100 MHz, $CDCl_3$) of compound **11**. 24
Figure S37: Infrared spectrum υmax of compound **11**. 25
Figure S38: ^{1}H NMR spectrum (500 MHz, $CDCl_3$) of compound **12**. 25
Figure S39: ^{13}C NMR-APT spectrum (125 MHz, $CDCl_3$) of compound **12**. 26
Figure S40: Infrared spectrum υmax of compound **12**. 26
Figure S41: HRMS spectrum of compound **12**. 27
Figure S42: ^{1}H NMR spectrum (400 MHz, $CDCl_3$) of compound **13**. 27
Figure S43: ^{13}C NMR-APT spectrum (100 MHz, $CDCl_3$) of compound **13**. 28
Figure S44: Infrared spectrum υmax of compound **13**. 28
Figure S45: ^{1}H NMR spectrum (400 MHz, $CDCl_3$) of compound **14**. 29
Figure S46: ^{13}C NMR-APT spectrum (100 MHz, $CDCl_3$) of compound **14**. 29
Figure S47: Infrared spectrum υmax of compound **14**. 30
Figure S48: HRMS spectrum of compound **14**. 30

Figure S49: ^{1}H NMR spectrum (400 MHz, $CDCl_3$) of compound **15**. 31
Figure S50: ^{13}C NMR-APT spectrum (100 MHz, $CDCl_3$) of compound **15**. 31
Figure S51: Infrared spectrum υmax of compound **15**. 32
Figure S52: HRMS spectrum of compound **15**. 32
Figure S53: ^{1}H NMR spectrum (400 MHz, $CDCl_3$) of compound **16**. 33
Figure S54: ^{13}C NMR-APT spectrum (100 MHz, $CDCl_3$) of compound **16**. 33
Figure S55: Infrared spectrum υmax of compound **16**. 34
Figure S56: HRMS spectrum of compound **16**. 34
Figure S57: ^{1}H NMR spectrum (400 MHz, $CDCl_3$) of compound **17**. 35
Figure S58: ^{13}C NMR-APT spectrum (100 MHz, $CDCl_3$) of compound **17**. 35
Figure S59: Infrared spectrum υmax of compound **17**. 36
Figure S60: HRMS spectrum of compound **17**. 36
Figure S61: ^{1}H NMR spectrum (500 MHz, $CDCl_3$) of compound **18**. 37
Figure S62: ^{13}C NMR-APT spectrum (125 MHz, $CDCl_3$) of compound **18**. 37
Figure S63: Infrared spectrum υmax of compound **18**. 38
Figure S64: HRMS spectrum of compound **18**. 38
Figure S65: ^{1}H NMR spectrum (500 MHz, DMSO-d_6) of compound **19**. 39
Figure S66: ^{13}C NMR-APT spectrum (125 MHz, DMSO-d_6) of compound **19**. 39
Figure S67: Infrared spectrum υmax of compound **19**. 40
Figure S68: HRMS spectrum of compound **19**. 40
Figure S69: ^{1}H NMR spectrum (500 MHz, $CDCl_3$) of compound **20**. 41
Figure S70: ^{13}C NMR-APT spectrum (125 MHz, $CDCl_3$) of compound **20**. 41
Figure S71: Infrared spectrum υmax of compound **20**. 42
Figure S72: HRMS spectrum of compound **20**. 42
Figure S73: ^{1}H NMR spectrum (400 MHz, $CDCl_3$) of compound **21**. 43
Figure S74: ^{13}C NMR-APT spectrum (100 MHz, $CDCl_3$) of compound **21**. 43
Figure S75: Infrared spectrum υmax of compound **21**. 44
Figure S76: HRMS spectrum of compound **21**. 44
Figure S77: ^{1}H NMR spectrum (500 MHz, $CDCl_3$) of compound **22**. 45
Figure S78: ^{13}C NMR-APT spectrum (125 MHz, $CDCl_3$) of compound **22**. 45
Figure S79: Infrared spectrum υmax of compound **22**. 46
Figure S80: HRMS spectrum of compound **22**. 46
Figure S81: ^{1}H NMR spectrum (500 MHz, $CDCl_3$) of compound **23**. 47
Figure S82: ^{13}C NMR-APT spectrum (125 MHz, $CDCl_3$) of compound **23**. 47
Figure S83: Infrared spectrum υmax of compound **23**. 48
Figure S84: HRMS spectrum of compound **23**. 48
Figure S85: ^{1}H NMR spectrum (500 MHz, $CDCl_3$) of compound **24**. 49
Figure S86: ^{13}C NMR-APT spectrum (125 MHz, $CDCl_3$) of compound **24**. 49
Figure S87: Infrared spectrum υmax of compound **24**. 50
Figure S88: HRMS spectrum of compound **24**. 50
Figure S89: ^{1}H NMR spectrum (500 MHz, $CDCl_3$) of compound **25**. 51
Figure S90: ^{13}C NMR-APT spectrum (125 MHz, $CDCl_3$) of compound **25**. 51
Figure S91: Infrared spectrum υmax of compound **25**. 52
Figure S92: HRMS spectrum of compound **25**. 52
Figure S93: RMSD plots for the predicted complex of compound **1** with CS. Separate plots are provided for the compound (top) and protein (bottom). The five MD replicas are labelled R1 to R5. 53

Figure S94: RMSD plots for the predicted complex of compound **2** with HOG1. Separate plots are provided for the compound (top) and protein (bottom). The five MD replicas are labelled R1 to R5. 54
Figure S95: RMSD plots for the predicted complex of compound **21** with HOG1. Separate plots are provided for the compound (top) and protein (bottom). The five MD replicas are labelled R1 to R5. 55
Figure S96: RMSD plots for the predicted complex of compound **2** with FBA1. Separate plots are provided for the compound (top) and protein (bottom). The five MD replicas are labelled R1 to R5. 56
Figure S97: RMSD plots for the predicted complex of compound **21** with FBA1. Separate plots are provided for the compound (top) and protein (bottom). The five MD replicas are labelled R1 to R5. 57
Figure S98: RMSFs of the protein in the complex of compound **1** with CS. One figure is provided per MD replica. The compound is represented as orange spheres. 58
Figure S99: RMSFs of the protein in the complex of compound **2** with HOG1. One figure is provided per MD replica. The compound is represented as orange spheres. 59
Figure S100: RMSFs of the protein in the complex of compound **21** with HOG1. One figure is provided per MD replica. The compound is represented as orange spheres. 60
Figure S101: RMSFs of the protein in the complex of compound **2** with FBA1. One figure is provided per MD replica. The compound is represented as orange spheres. 61
Figure S102: RMSFs of the protein in the complex of compound **21** with FBA1. One figure is provided per MD replica. The compound is represented as orange spheres. 62

Table S1. Docking scores for the selected complexes. 63
Table S2. Results of MM-PBSA calculations. 68

Spectroscopic data of compounds

7-Hydroxychroman-4-one (**1**): Yellow solid. Yield: 51% (5.0 mmol; 821 mg). M.P.: 140.5-141.2°C (lit. = 143 °C [1]); TLC (8:2 hexane/EtOAc, R_f = 0.25); IR υmax (cm^{-1}): 3261, 2999, 1662, 1584, 1480, 1384, 1336, 1281, 1228, 1118 ^1H NMR (500 MHz, DMSO-d_6): δ_H = 10.54 (*s*, 7-OH; 1H), 7.61 (*d*, *J*=8.7 Hz; 1H; H-5), 6.48 (*dd*, *J* = 8.7; 2.3 Hz; 1H; H-6), 6.30 (*d*, *J*=2.3 Hz; 1H; H-8), 4.45 (*t*, *J*=6.1 Hz; 2H; H-2), 2.66 (*t*, *J*=6.7 Hz; 2H; H-3). ^{13}C NMR-APT (125 MHz, DMSO-d_6): δ_C = 189.79, 164.42, 163.37, 128.54, 113.97, 110.41, 102.38, 66.94, 36.92 [1].

7-Methoxychroman-4-one (**2**): Yellow oil. Yield: 62% (0.38 mmol; 67.4 mg); TLC (8:2 hexane/EtOAc, R_f = 0.52); IR υmax (cm^{-1}): 2945, 1677, 1602, 1438, 1384, 1334, 1253, 1157, 1117; ^1H NMR (400 MHz, DMSO-d_6): δ_H 7.68 (*d*, *J*=8.8 Hz, 1H; H-5); 6.63 (*dd*, *J*=8.8, 2.4 Hz, 1H; H-6); 6.53 (*d*, *J*=2.4 Hz, 1H; H-8); 4.50 (*t*, *J*=5.9 Hz, 2H; H-2); 3.80 (*s*, 3H; H-1′); 2.70 (*t*, *J*=6.0 Hz, 2H; H-3). ^{13}C NMR-APT (100 MHz, DMSO-d_6): δ_C 190.12; 165.50; 163.50; 128.22; 114.90; 109.72; 100.88; 67.16; 55.82; 36.92 [2].

7-Propoxychroman-4-one (**3**): Yellow oil. Yield: 31% (0.19 mmol; 38.4 mg); TLC (8:2 hexane/EtOAc, R_f = 0.62); IR υmax (cm^{-1}): 2967, 1679, 1602, 1438, 1383, 1331, 1251, 1161, 1117; ^1H NMR (400 MHz, DMSO-d_6): δ_H 7.67 (*d*, *J*=8.8 Hz, 1H; H-5); 6.63 (*dd*, *J*=8.8, 2.4 Hz, 1H; H-6); 6.52 (*d*, *J*=2.4 Hz, 1H; H-8); 4.50 (*t*, *J*=5.9 Hz, 2H; H-2); 3.99 (*t*, *J*=6.6 Hz, 2H; H-1′); 2.70 (*t*, *J*=6.1 Hz, 2H; H-3); 1.80-1.62 (*m*, 2H; H-2′); 0.97 (*t*, *J*=7.4 Hz, 3H; H-3′). ^{13}C NMR-APT (100 MHz, DMSO-d_6): δ_C 189.98; 164.87; 163.42; 128.15; 114.75; 109.93; 101.24; 69.55; 67.08; 36.88; 21.80; 10.27 [2].

7-Isopropoxychroman-4-one (**4**): Yellow oil. Yield: 56% (0.34 mmol; 83.6 mg); TLC (8:2 hexane/EtOAc, R_f = 0.62); IR υmax (cm^{-1}): 2978, 1663, 1596, 1488, 1383, 1331, 1258, 1143, 1110; ^1H NMR (500 MHz, $CDCl_3$): δ_H 7.82 (*d*, *J*=8.8 Hz, 1H; H-5); 6.54 (*dd*, *J*=8.8, 2.4 Hz, 1H; H-6); 6.38 (*d*, *J*=2.4 Hz, 1H; H-8); 4.59 (*sept*, *J*=6.0 Hz, 1H; H-1′); 4.50 (*t*, *J*=6.2 Hz, 2H; H-2); 2.73 (*t*, *J*=6.5 Hz, 2H; H-3); 1.35 (*d*, *J*=6.1 Hz, 6H; H-2′; H-3′). ^{13}C NMR-APT (125 MHz, $CDCl_3$): δ_C 190.63; 164.65; 163.94; 129.07; 115.11; 111.07; 102.20; 70.56; 67.49; 37.61; 22.01 [3].

7-((4-Bromobenzyl)oxy)chroman-4-one (**6**): White solid. Yield: 35% (0.21 mmol; 71.4 mg); M.P.: 146.1-147.0°C (lit: 144.8-144.9 °C [4]); TLC (8:2 hexane/EtOAc, R_f = 0.48); IR υmax (cm^{-1}): 3035, 2954, 1664, 1608, 1494, 1439, 1381, 1254, 1164, 1119; ^1H NMR (400 MHz, DMSO-d_6): δ_H 7.69 (*d*, *J*=8.80 Hz, 1H; H-5); 7.64-7.56 (*m*, 2H; H-3′; H-5′); 7.44-7.36 (*m*, 2H; H-2′; H-6′); 6.70 (*dd*, *J*=8.80, 2.40 Hz, 1H; H-6); 6.61 (*d*, *J*=2.40 Hz, 1H; H-8); 5.16 (*s*, 2H; H-7′); 4.50 (*t*, *J*=5.9 Hz, 2H; H-2); 2.70 (*t*, *J*=6.6 Hz, 2H; H-3). ^{13}C NMR-APT (100 MHz, DMSO-d_6): δ_C 190.04; 164.20; 163.34; 135.79; 131.47; 129.90; 128.24; 121.21; 115.11; 110.21; 101.88; 68.86; 67.13; 36.88 [4].

7-((4-Chlorobenzyl)oxy)chroman-4-one (**7**): White solid. Yield: 39% (0.24 mmol; 68.1 mg); M.P.: 119.8-120.2°C (lit: 121.3-121.5°C [4]); TLC (8:2 hexane/EtOAc, R_f = 0.46); IR υmax (cm^{-1}): 3039, 2953, 1677, 1604, 1494, 1439, 1380, 1251, 1162, 1120. ^1H NMR (400 MHz, DMSO-d_6): δ_H 7.69 (*d*, *J*=8.8 Hz, 1H; H-5); 7.47 (*m*, 4H; H-2′; H-3′; H-5′; H-6′); 6.70 (*dd*, *J*=8.8, 2.4 Hz, 1H; H-6); 6.62 (*d*, *J*=2.4 Hz, 1H; H-8); 5.18 (*s*, 2H; H-7′); 4.50 (*t*, *J*=5.9 Hz, 2H; H-2); 2.70 (*t*, *J*=5.7 Hz, 2H; H-3). ^{13}C NMR-APT (100 MHz, DMSO-d_6): δ_C 190.01; 164.21; 163.33; 135.36; 132.65; 129.61; 128.53; 128.22; 115.10; 110.20; 101.86; 68.81; 67.12; 36.87 [4].

(*E*)-3-Benzylidene-7-methoxychroman-4-one (**11**): Yellow solid. Yield: 38% (0.22 mmol; 57.5 mg); M.P.: 73.2-74.1°C (lit. 69°C [5]); TLC (8:2 hexane/EtOAc, R_f = 0.68); IR υmax (cm^{-1}): 3008, 2926,

1666, 1601, 1435, 1384, 1336, 1256, 1157, 1101; ^{1}H NMR (400 MHz, $CDCl_3$): δ_H 7.97 (*d*, *J*=8.9 Hz, 1H; H-5); 7.85 (*t*, *J*=2.0 Hz, 1H; H-7''); 7.49-7.39 (*m*, 3H; H-2''; H-6''; H-4''); 7.31-7.29 (*m*, 2H; H-3''; H-5''); 6.63 (*dd*, *J*=8.9, 2.4 Hz, 1H; H-6); 6.40 (*d*, *J*=2.4 Hz, 1H; H-8); 5.33 (*d*, *J*=2.0 Hz, 2H; H-2); 3.84 (*s*, 3H; H-1'). ^{13}C NMR-APT (100 MHz, $CDCl_3$): δ_C 181.11; 166.18; 163.24; 136.89; 134.69; 130.99; 130.02; 129.84; 129.38; 128.80; 115.87; 110.61; 100.90; 67.98; 55.77 [5].

(*E*)-3-Benzylidene-7-isopropoxychroman-4-one (**13**): Yellow solid. Yield: 47% (0.23 mmol; 66.65 mg); M.P.: 65.3-66.1°C (lit. 68°C [3]; TLC (8:2 hexane/EtOAc, R_f = 0.80); IR υmax (cm^{-1}): 3030, 2978, 1667, 1600, 1437, 1383, 1255, 1159, 1101; ^{1}H NMR (400 MHz, $CDCl_3$): δ_H 7.94 (*d*, *J*=8.9 Hz, 1H; H-5); 7.83 (*t*, *J*=2.0 Hz, 1H; H-7''); 7.47-7.35 (*m*, 3H; H-2''; H-6''; H-4''); 7.31-7.27 (*m*, 2H; H-3''; H-5''); 6.59 (*dd*, *J*=8.8, 2.4 Hz, 1H; H-6); 6.36 (*d*, *J*=2.4 Hz, 1H; H-8); 5.31 (*d*, *J*=2.0 Hz, 2H; H-2); 4.59 (*sept*, *J*=6.0 Hz, 1H; H-1'); 1.35 (*d*, *J*=6.0 Hz, 6H; H-2'; H-3'). ^{13}C NMR-APT (100 MHz, $CDCl_3$): δ_C 181.06; 164.70; 163.21; 136.72; 134.69; 131.08; 129.97; 129.84; 129.32; 128.77; 115.50; 111.60; 102.16; 70.56; 67.87; 21.92 [3].

Spectra of the obtained compounds (**1-25**)

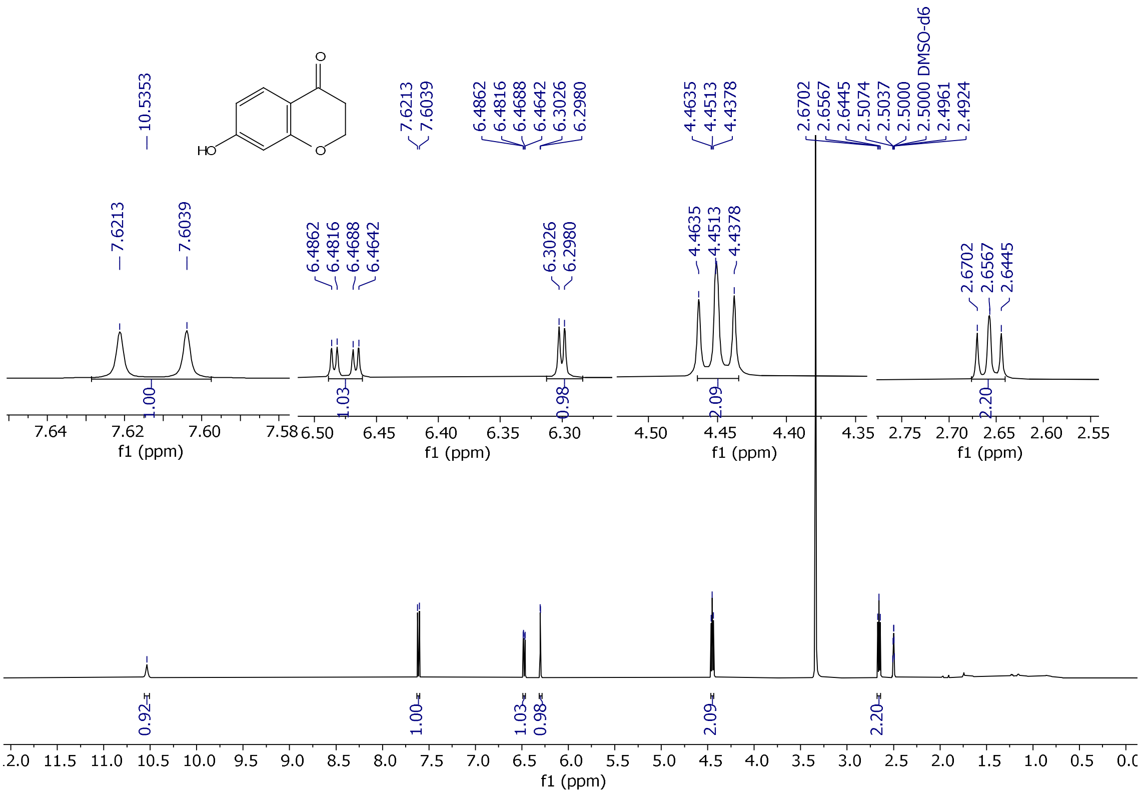

Figure S1: ^{1}H NMR spectrum (500 MHz, DMSO-d_6) of compound **1**.

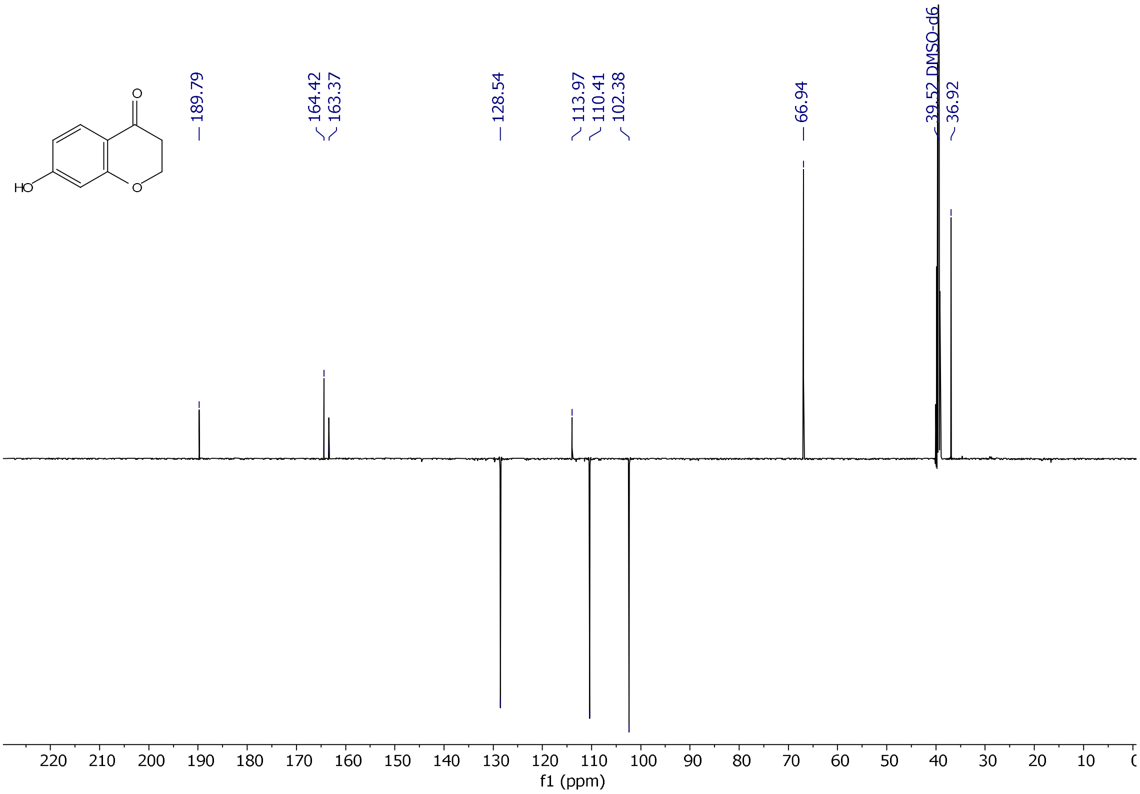

Figure S2: ^{13}C NMR-APT spectrum (125 MHz, DMSO-d_6) of compound **1**.

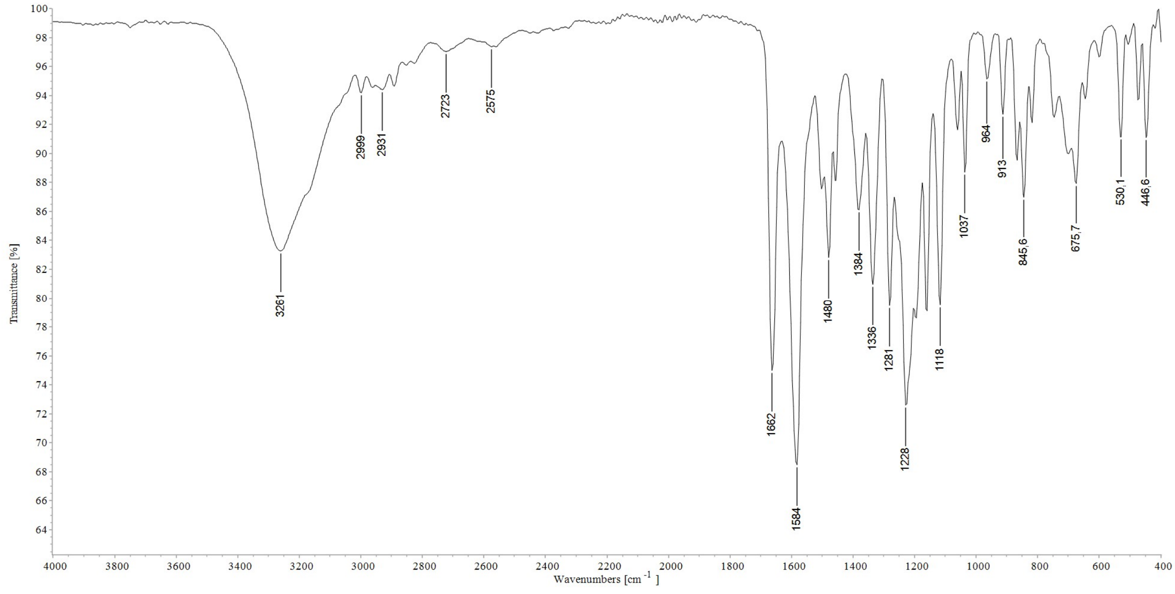

Figure S3: Infrared spectrum υmax of compound **1**.

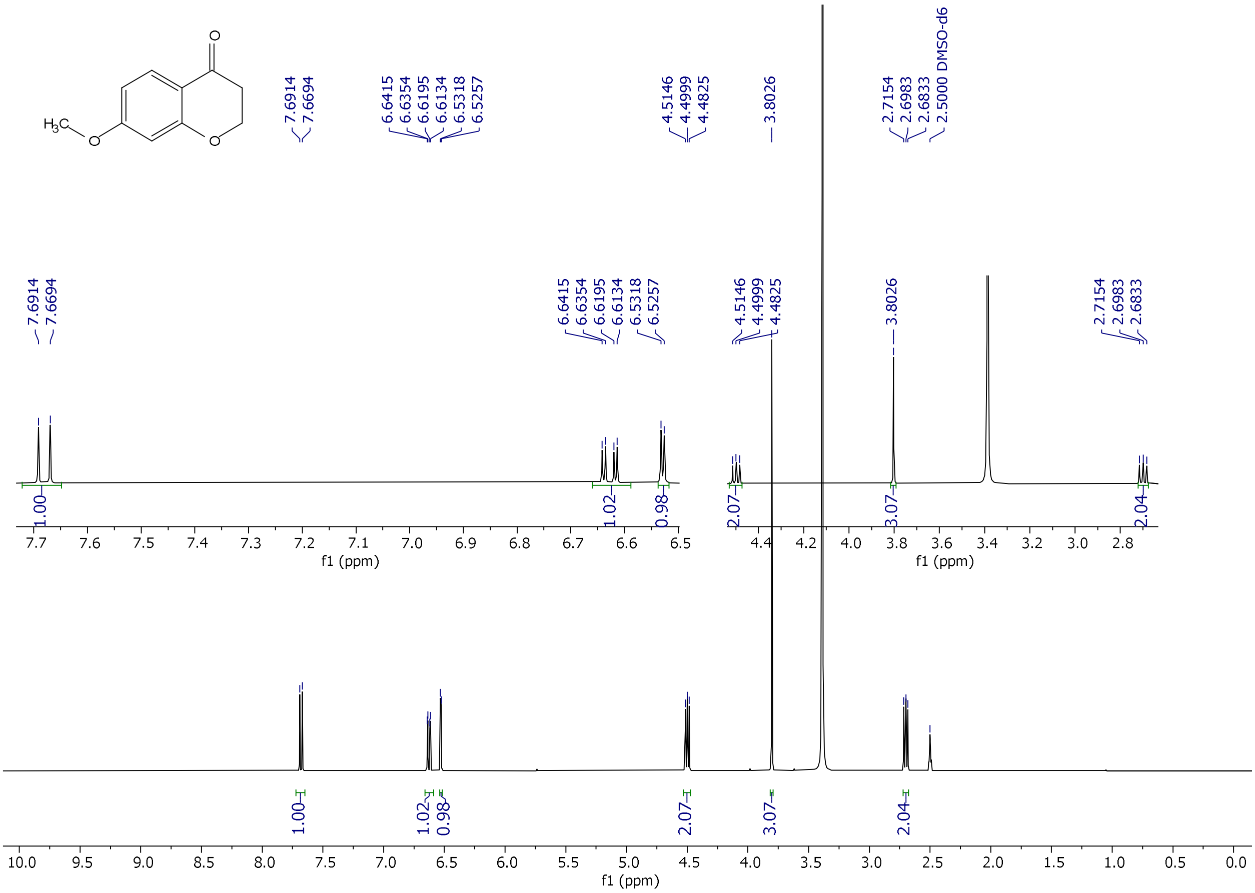

Figure S4: ^{1}H NMR spectrum (400 MHz, DMSO-d_6) of compound **2**.

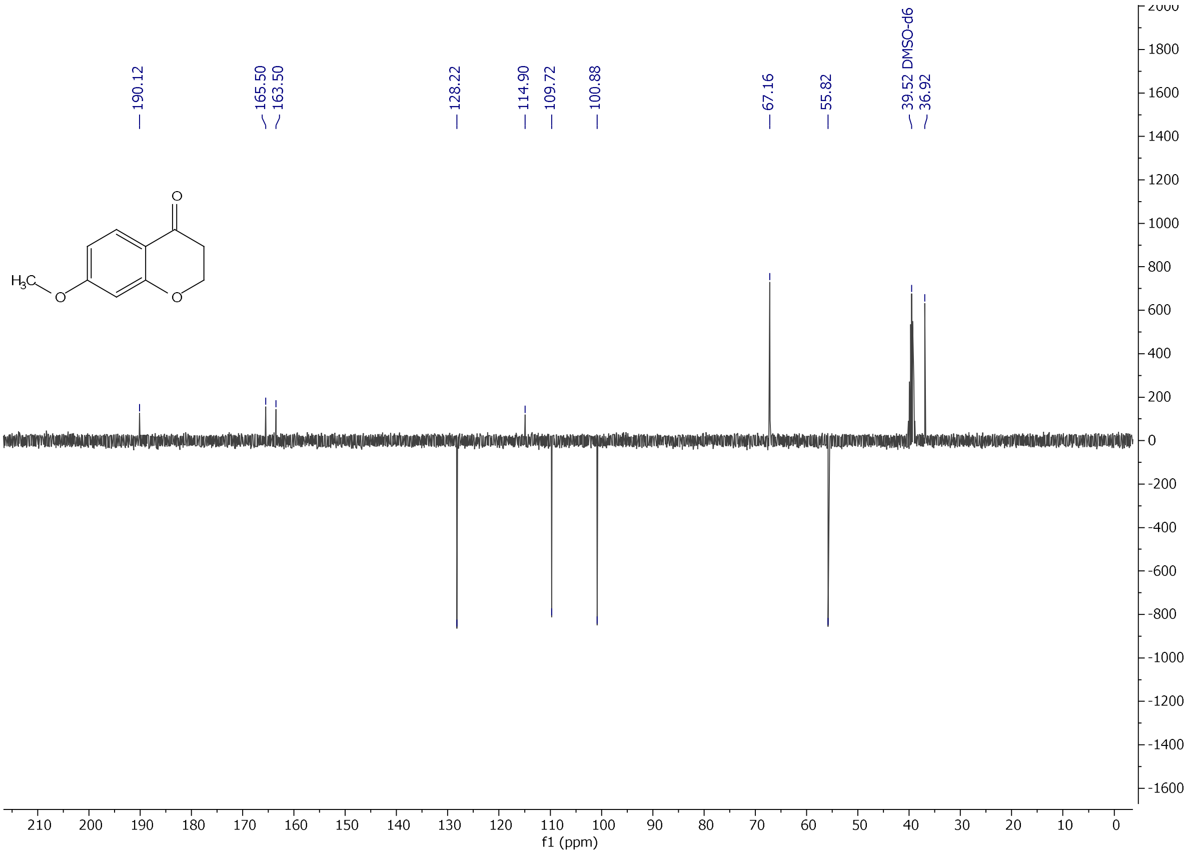

Figure S5: ^{13}C NMR-APT spectrum (100 MHz, DMSO-d_6) of compound **2**.

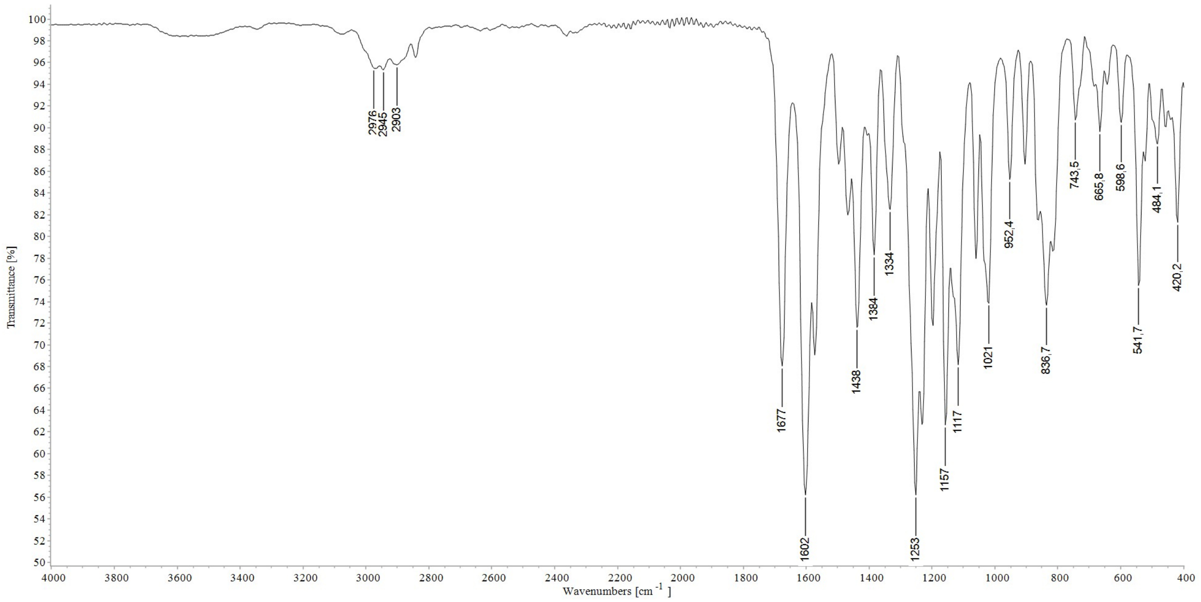

Figure S6: Infrared spectrum υmax of compound **2**.

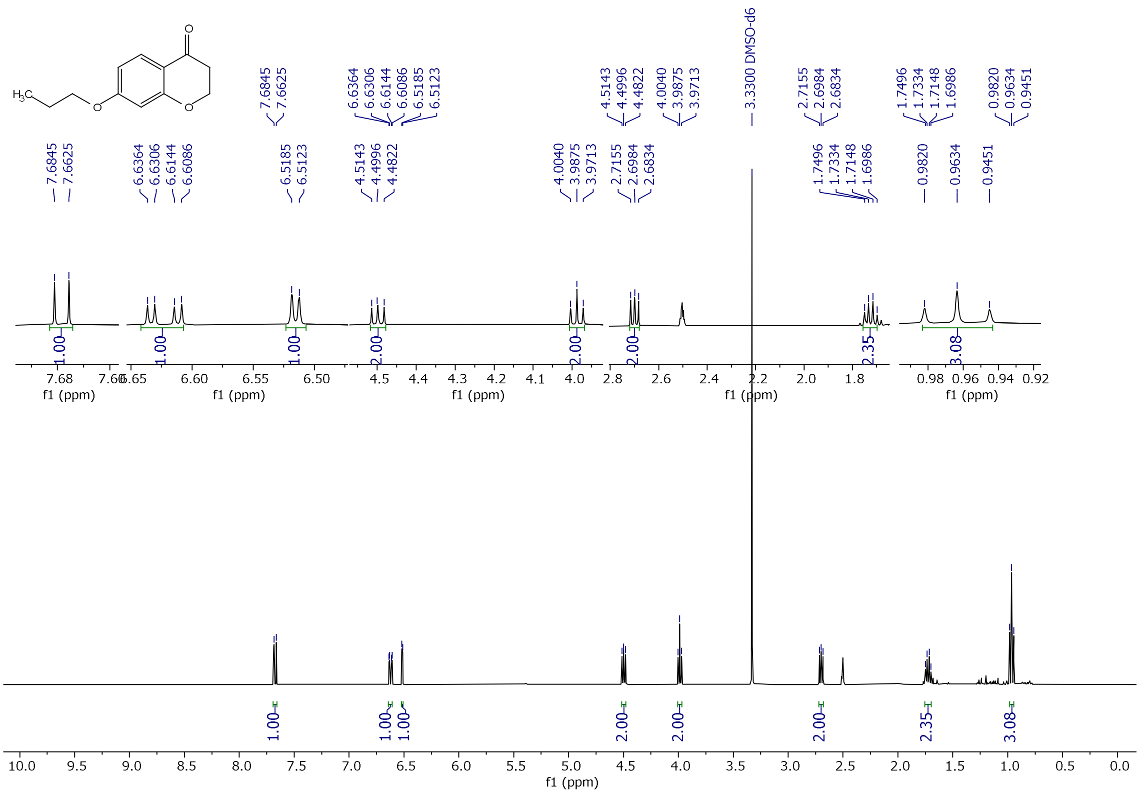

Figure S7: ^{1}H NMR spectrum (400 MHz, DMSO-d_6) of compound **3**.

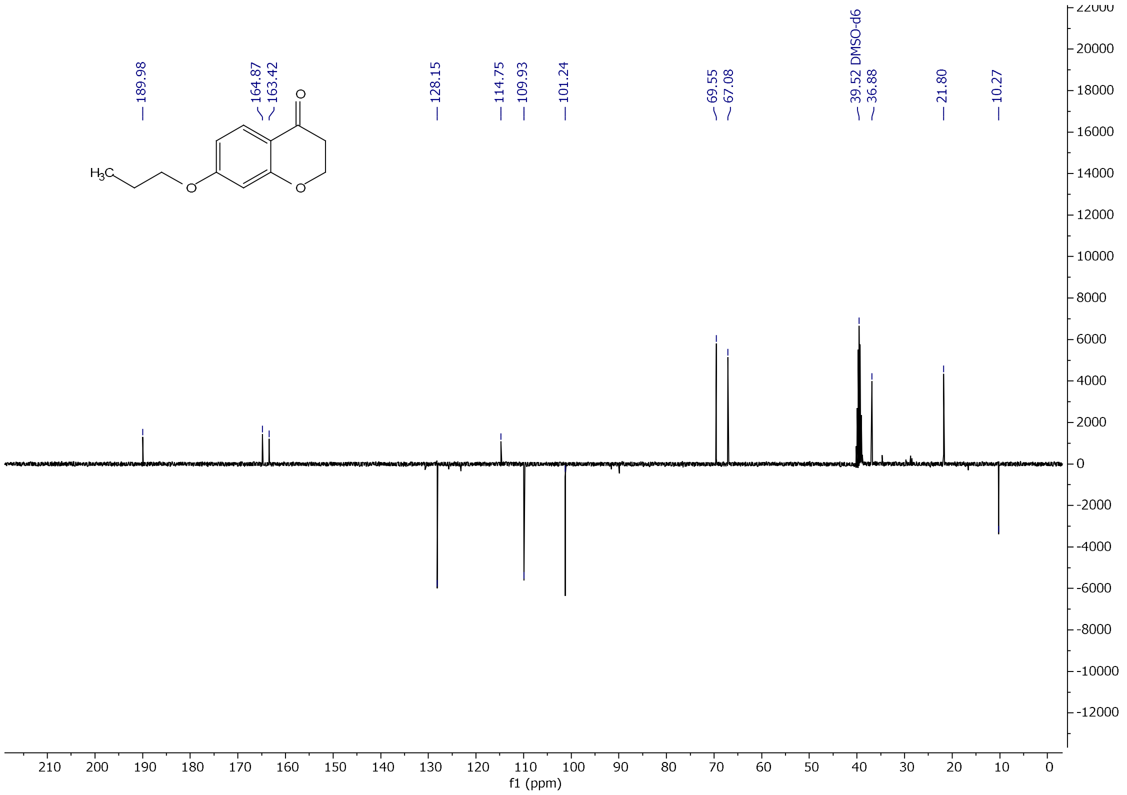

Figure S8: ^{13}C NMR-APT spectrum (100 MHz, DMSO-d_6) of compound **3**.

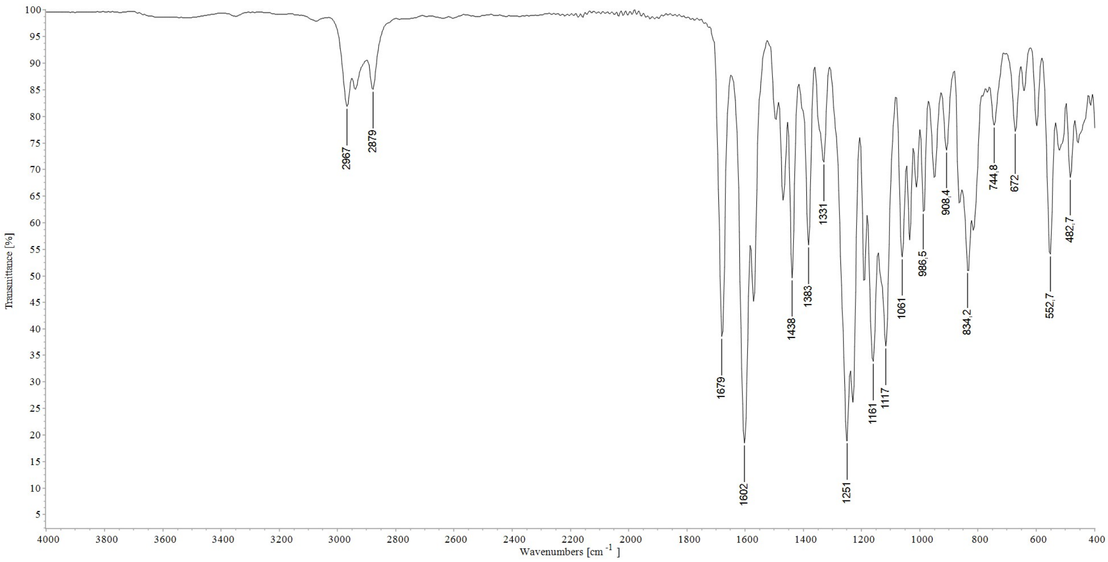

Figure S9: Infrared spectrum υmax of compound **3**.

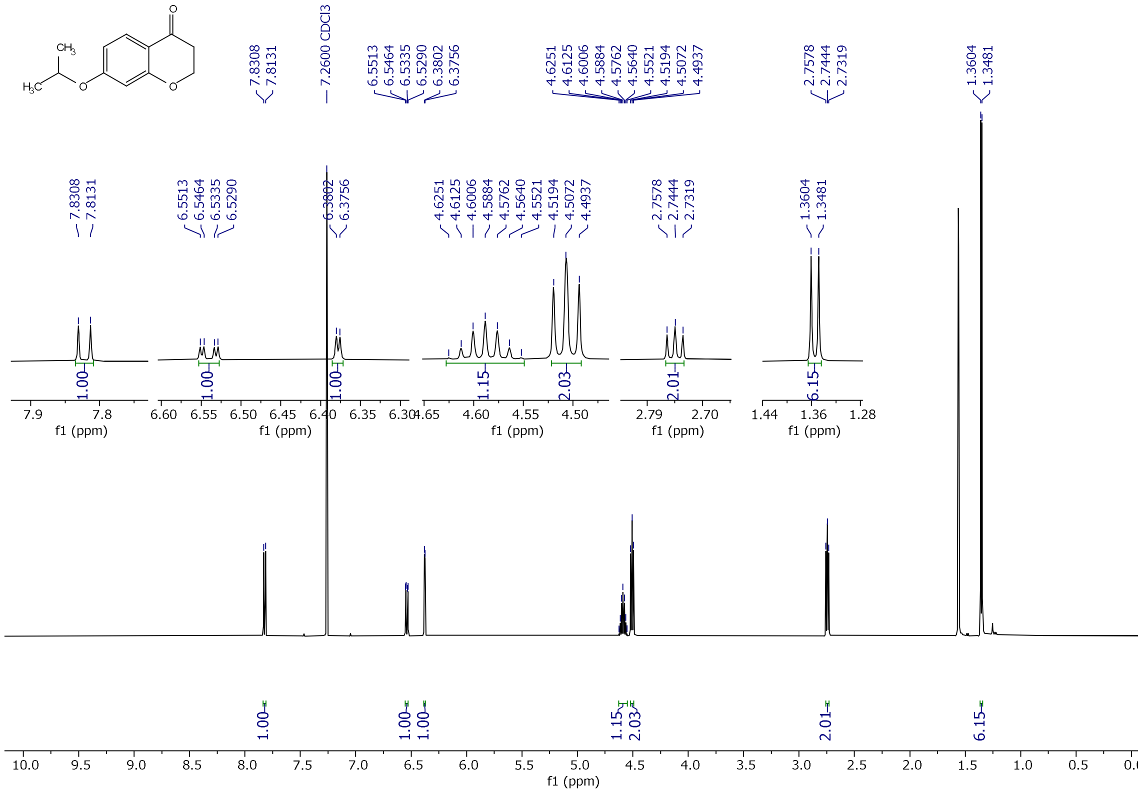

Figure S10: ^{1}H NMR spectrum (500 MHz, $CDCl_3$) of compound **4**.

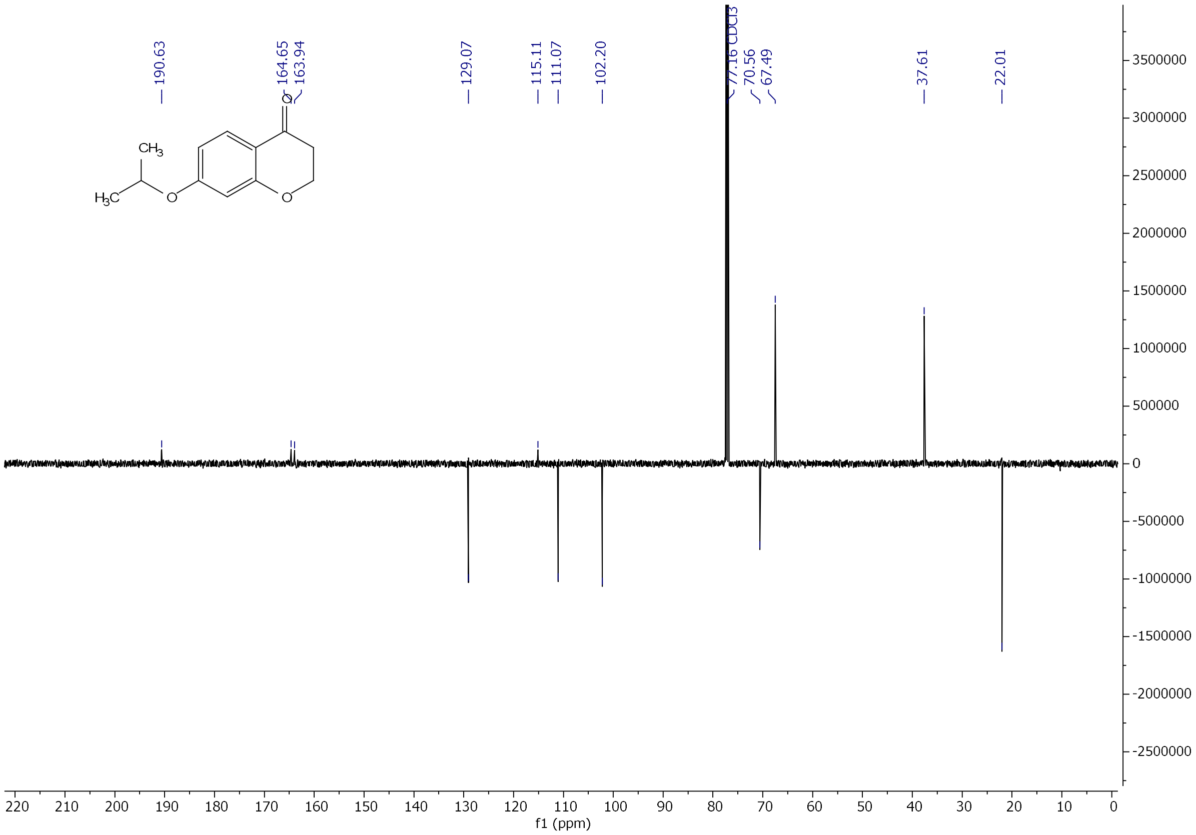

Figure S11: ^{13}C NMR-APT spectrum (125 MHz, $CDCl_3$) of compound **4**.

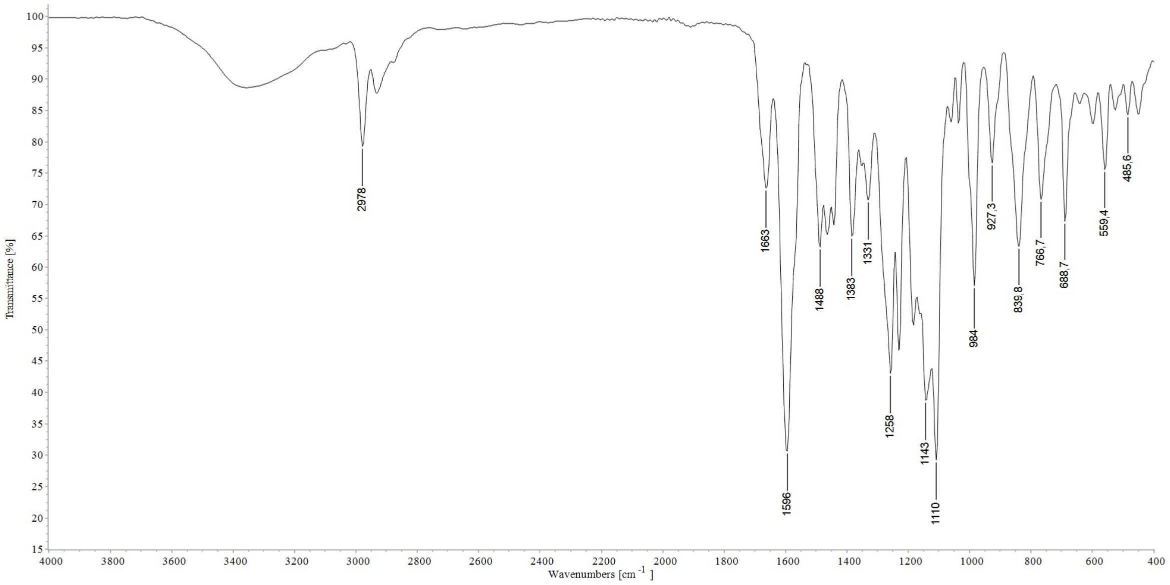

Figure S12: Infrared spectrum υmax of compound **4**.

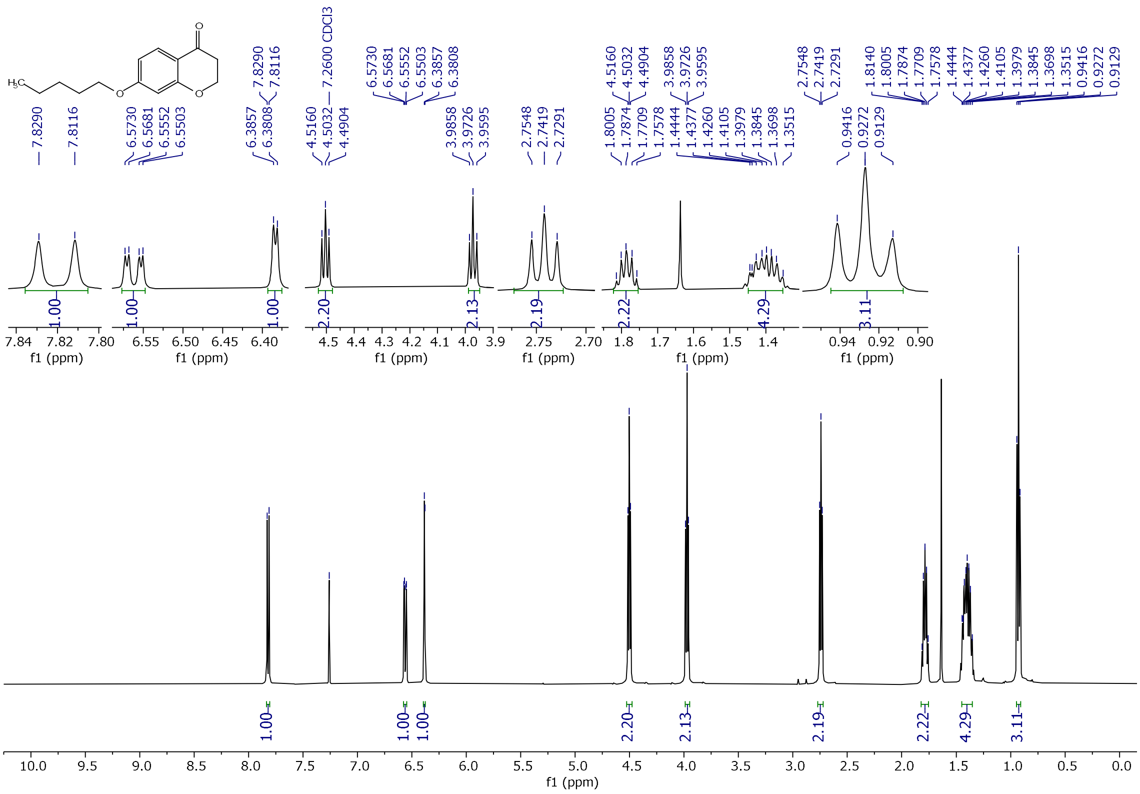

Figure S13: ^{1}H NMR spectrum (500 MHz, $CDCl_3$) of compound **5**.

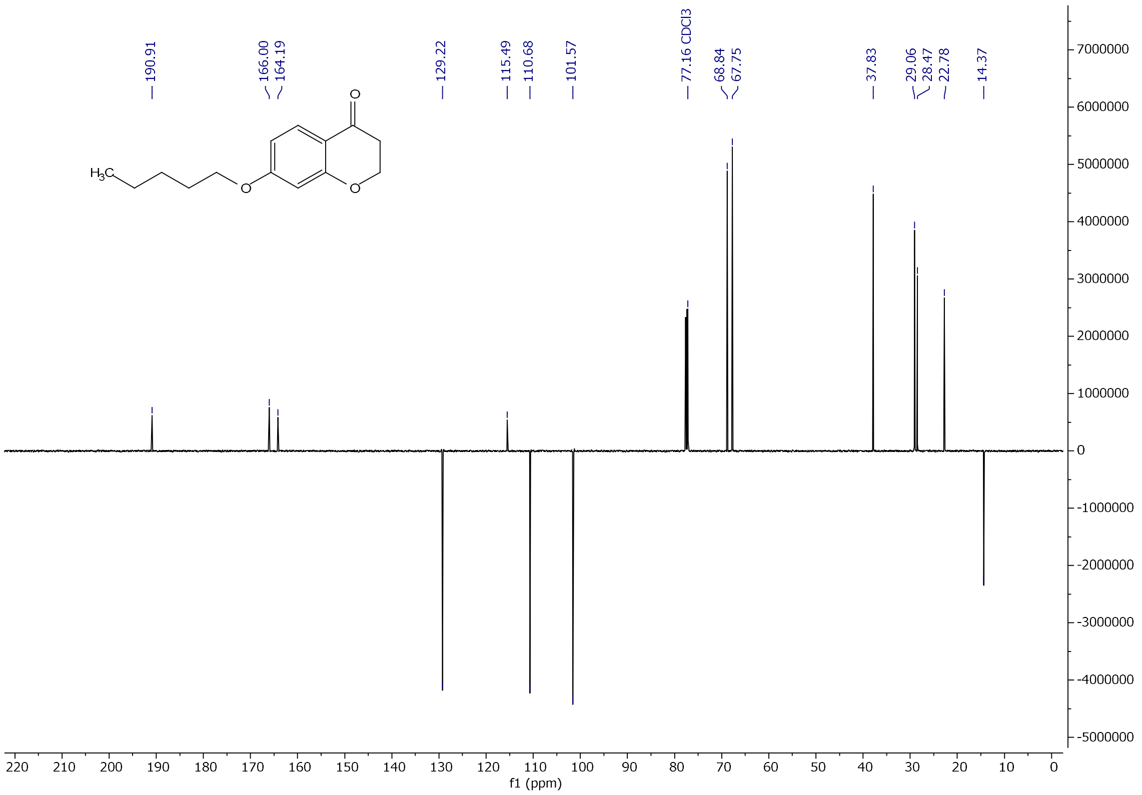

Figure S14: ^{13}C NMR-APT spectrum (125 MHz, $CDCl_3$) of compound **5**.

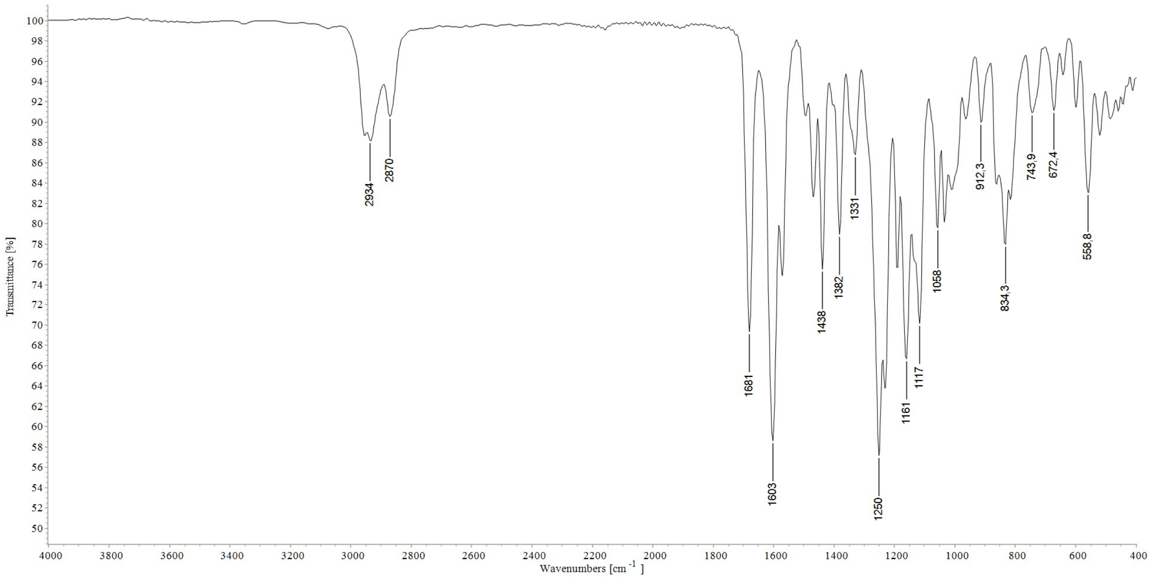

Figure S15: Infrared spectrum υmax of compound **5**.

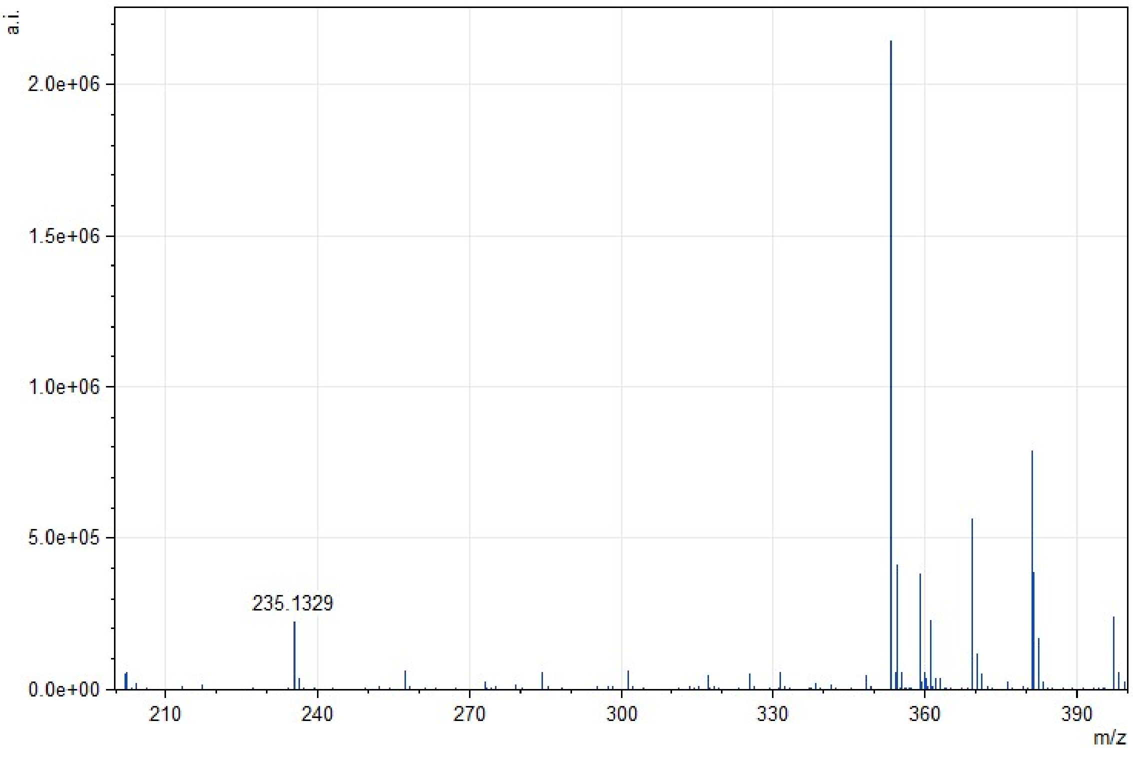

Figure S16: HRMS spectrum of compound **5**.

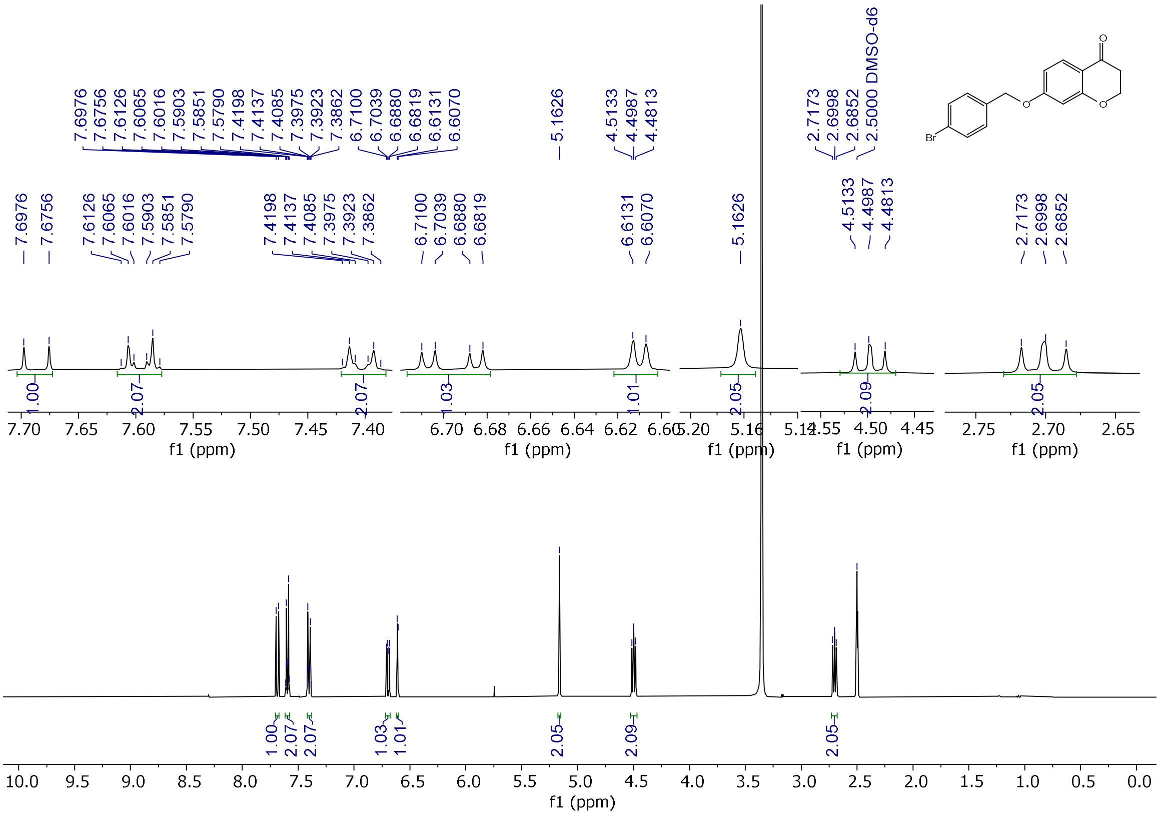

Figure S17: ^{1}H NMR spectrum (400 MHz, DMSO-d$_6$) of compound **6**.

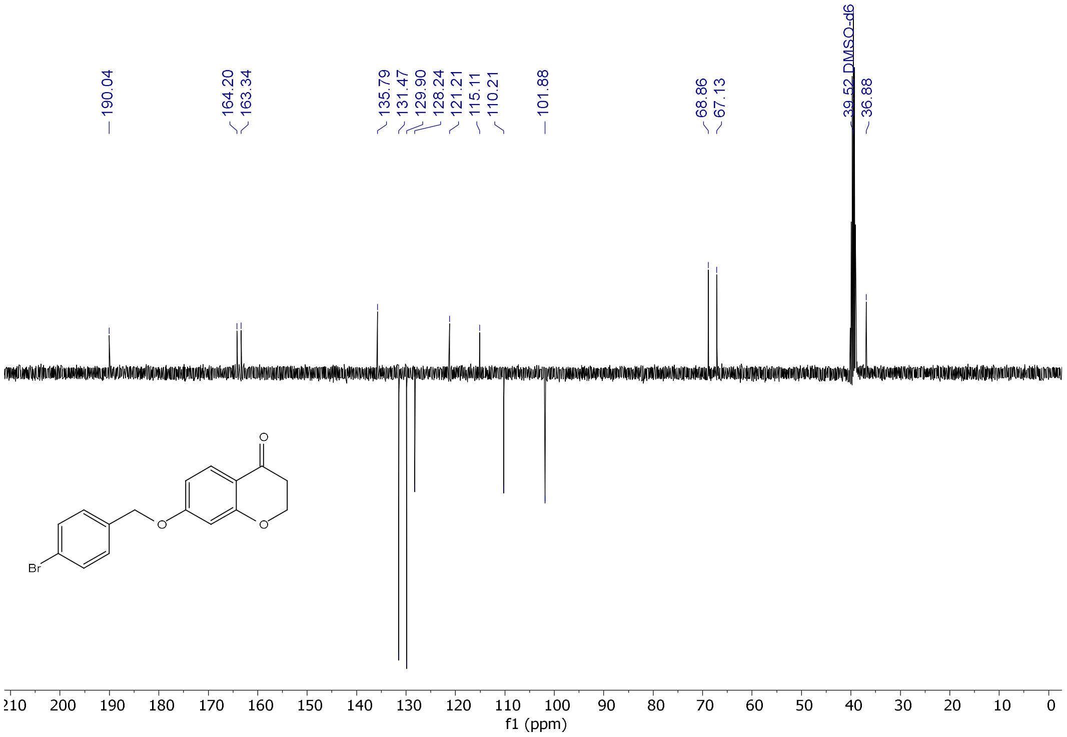

Figure S18: ^{13}C NMR-APT spectrum (100 MHz, DMSO-d$_6$) of compound **6**.

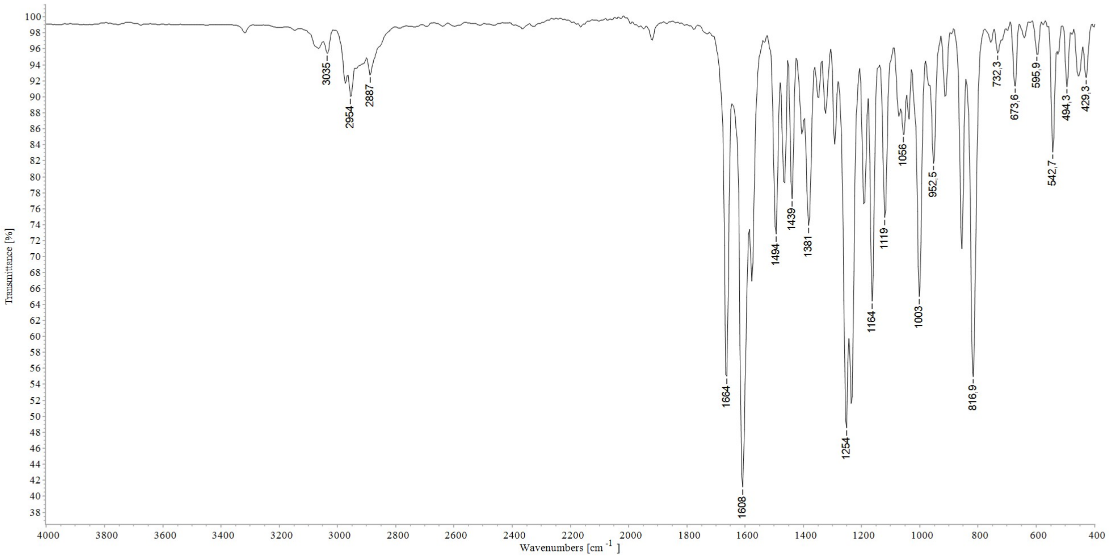

Figure S19: Infrared spectrum υmax of compound **6**.

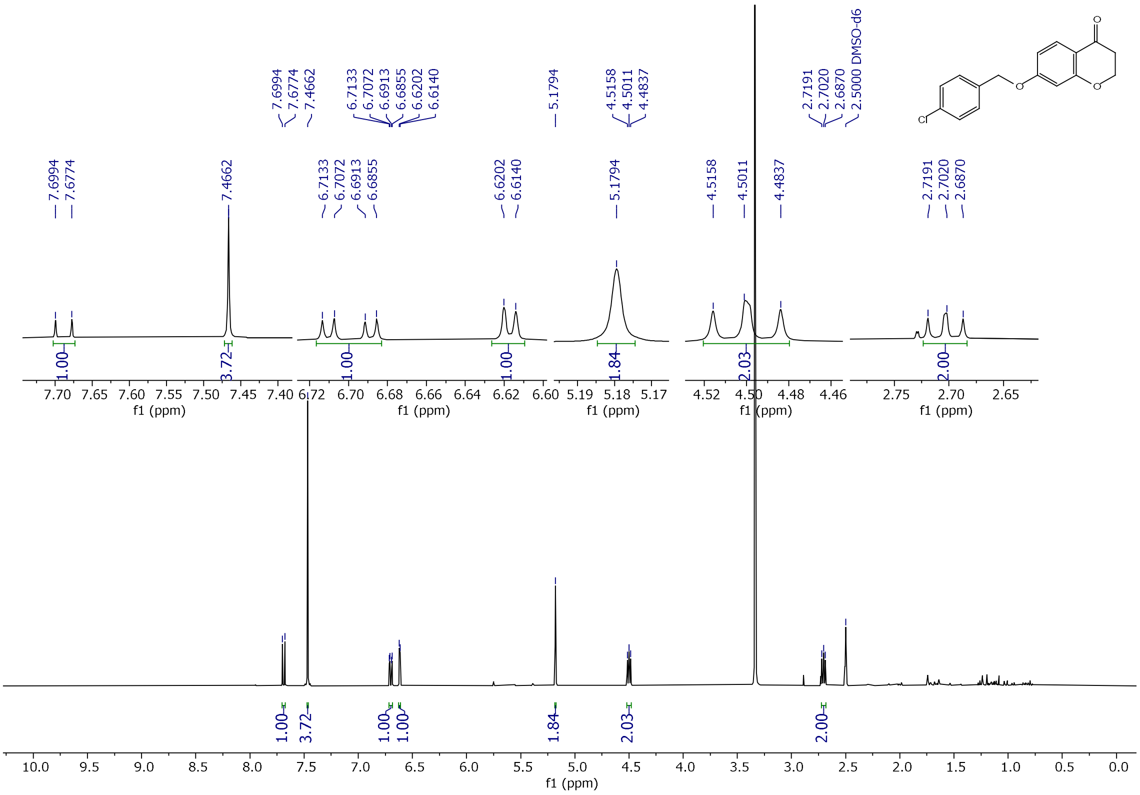

Figure S20: ^{1}H NMR spectrum (400 MHz, DMSO-d$_6$) of compound **7**.

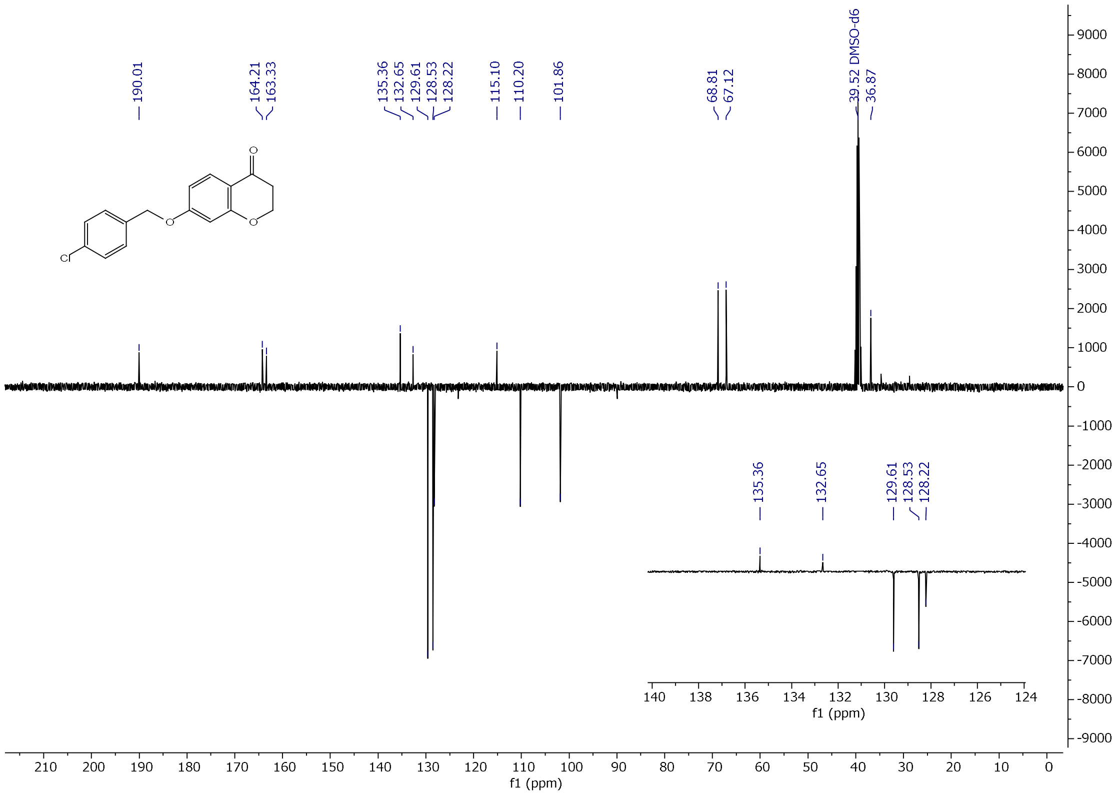

Figure S21: ^{13}C NMR-APT spectrum (100 MHz, DMSO-d_6) of compound **7**.

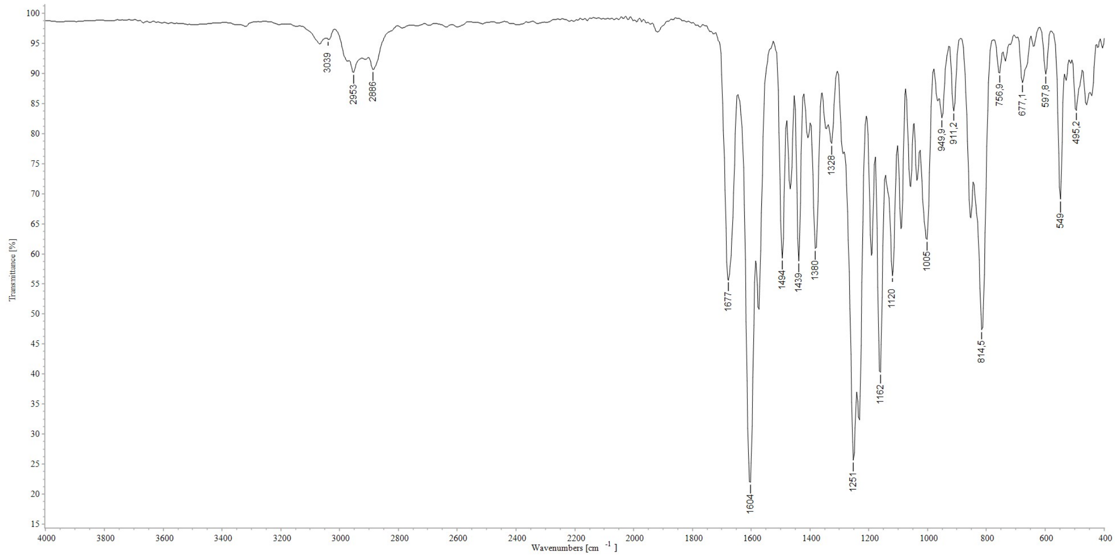

Figure S22: Infrared spectrum υmax of compound **7**.

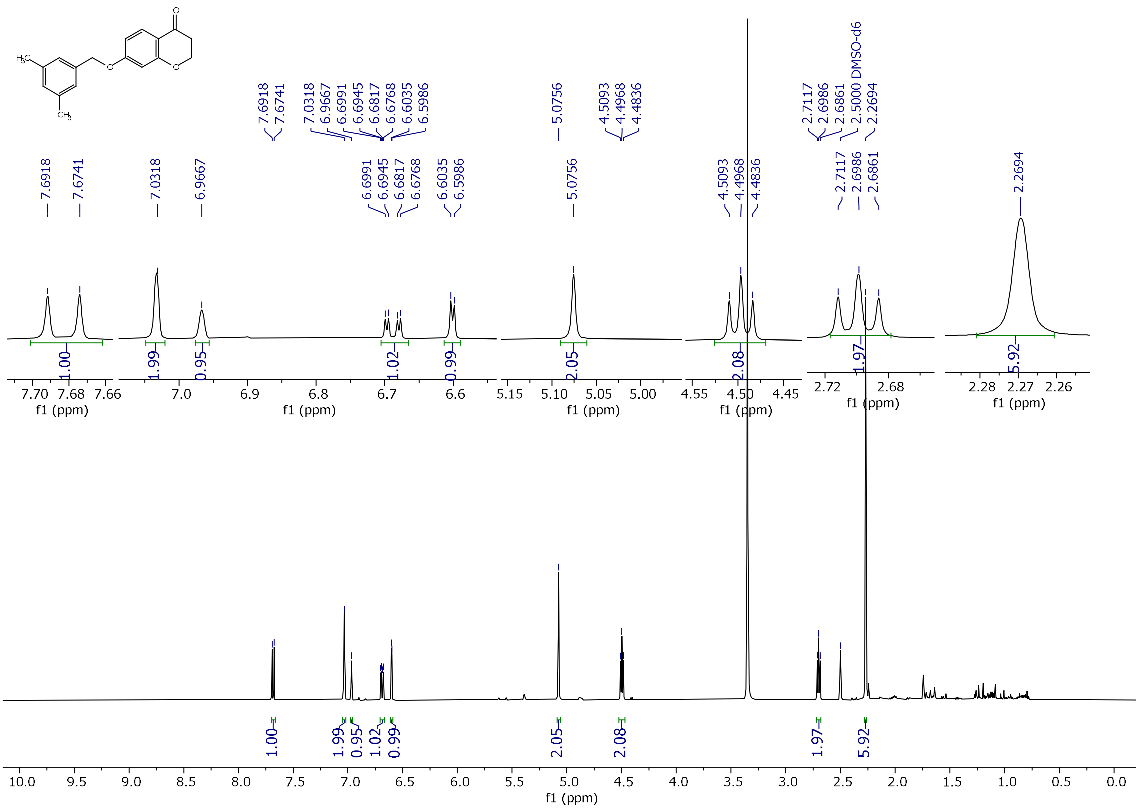

Figure S23: ^{1}H NMR spectrum (500 MHz, DMSO-d₆) of compound **8**.

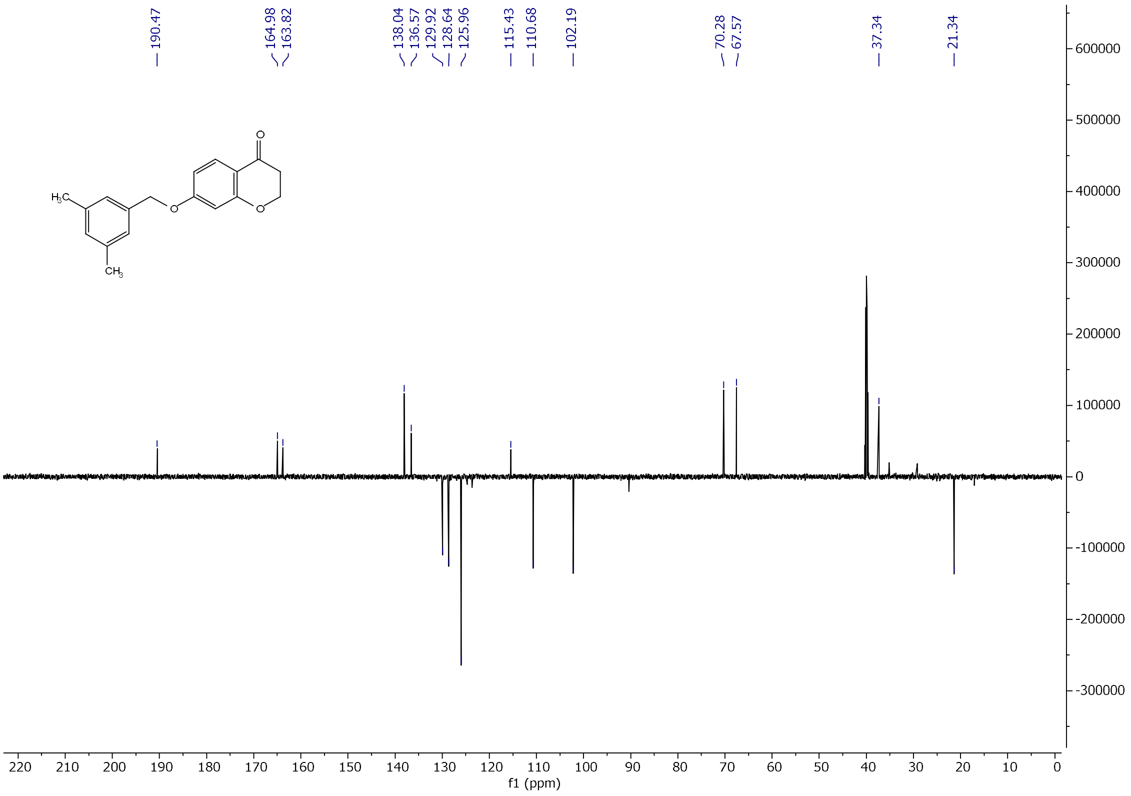

Figure S24: ^{13}C NMR-APT spectrum (125 MHz, DMSO-d₆) of compound **8**.

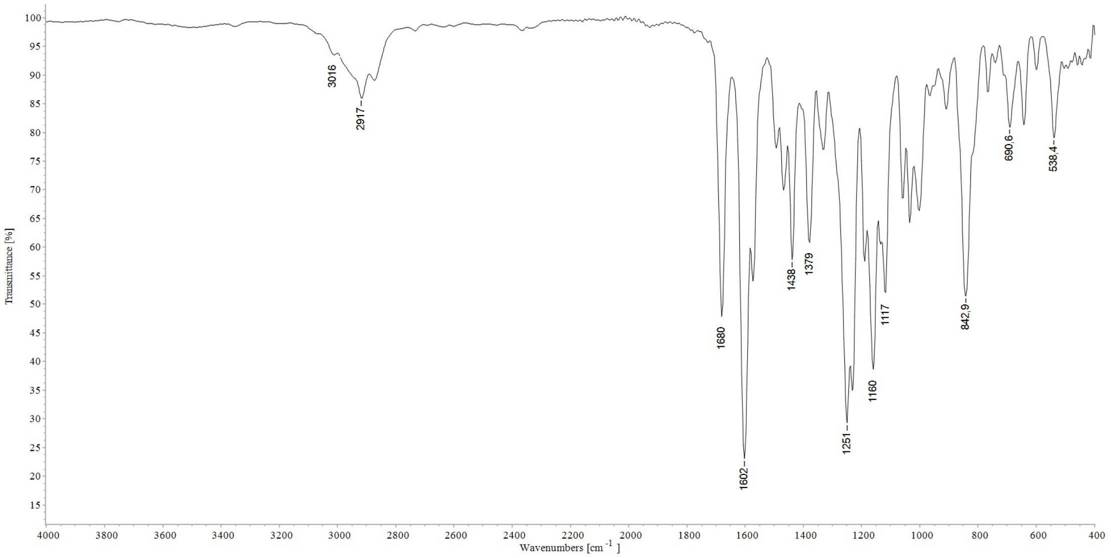

Figure S25: Infrared spectrum υmax of compound **8**.

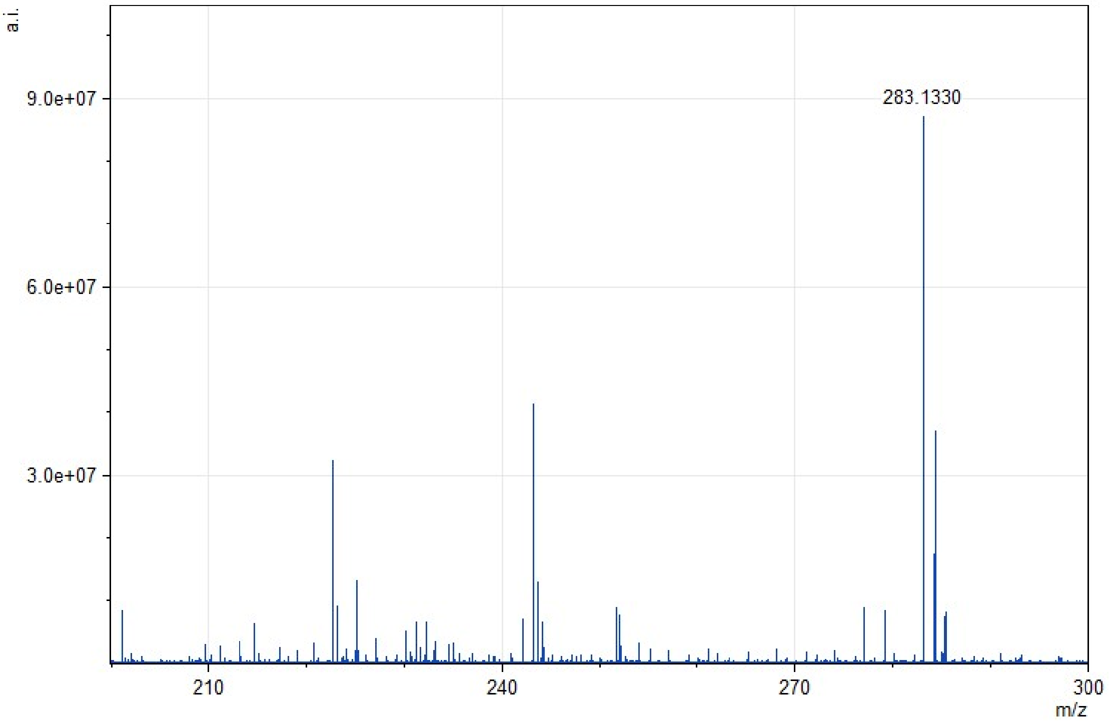

Figure S26: HRMS spectrum of compound **8**.

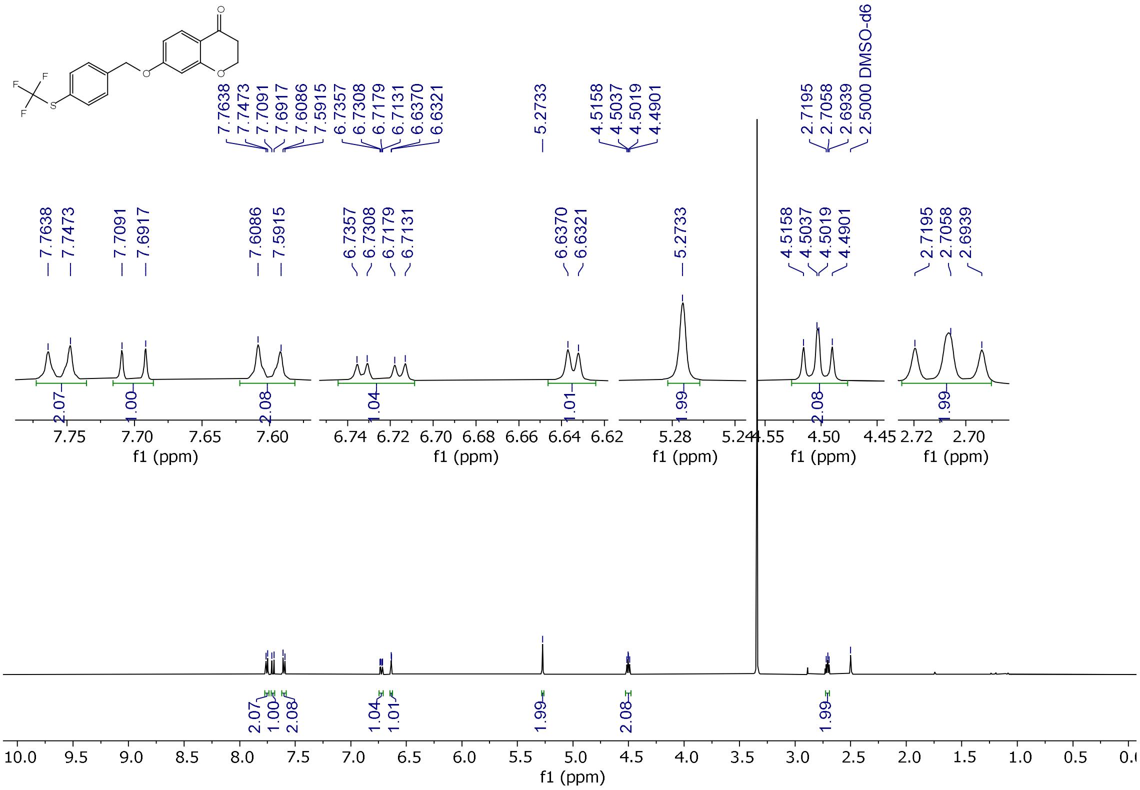

Figure S27: ^{1}H NMR spectrum (500 MHz, DMSO-d_6) of compound **9**.

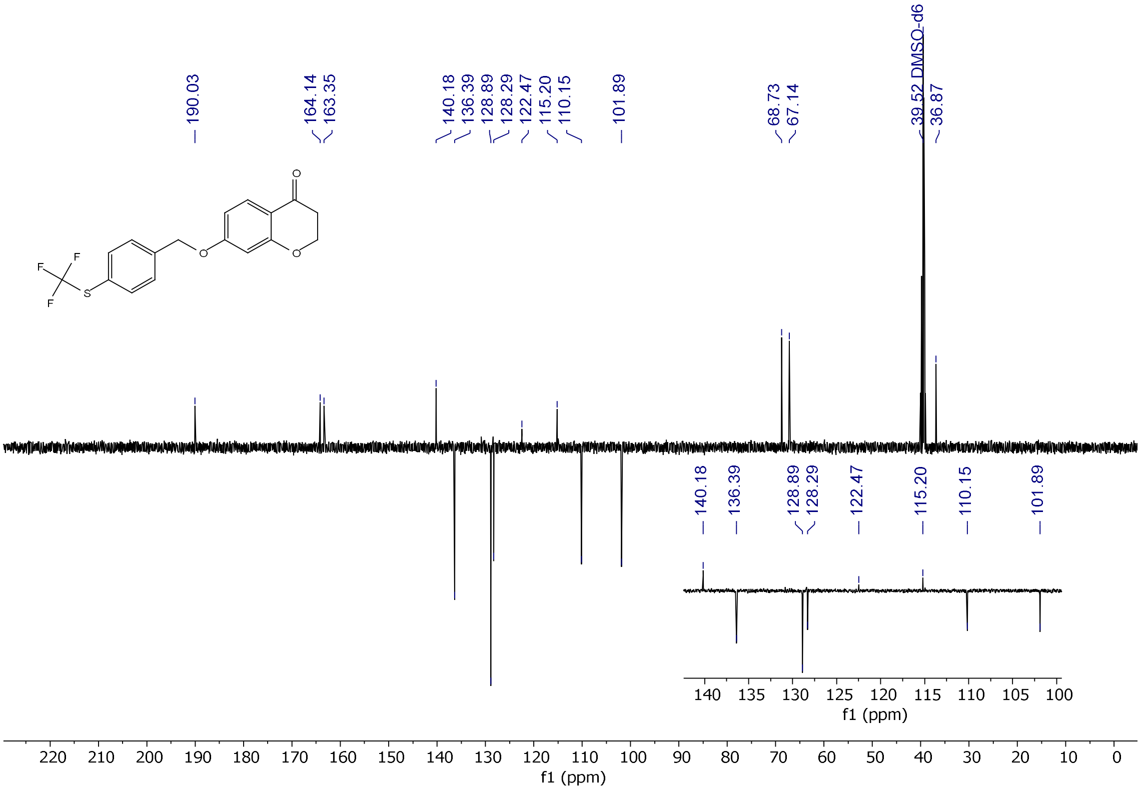

Figure S28: ^{13}C NMR-APT spectrum (125 MHz, DMSO-d_6) of compound **9**.

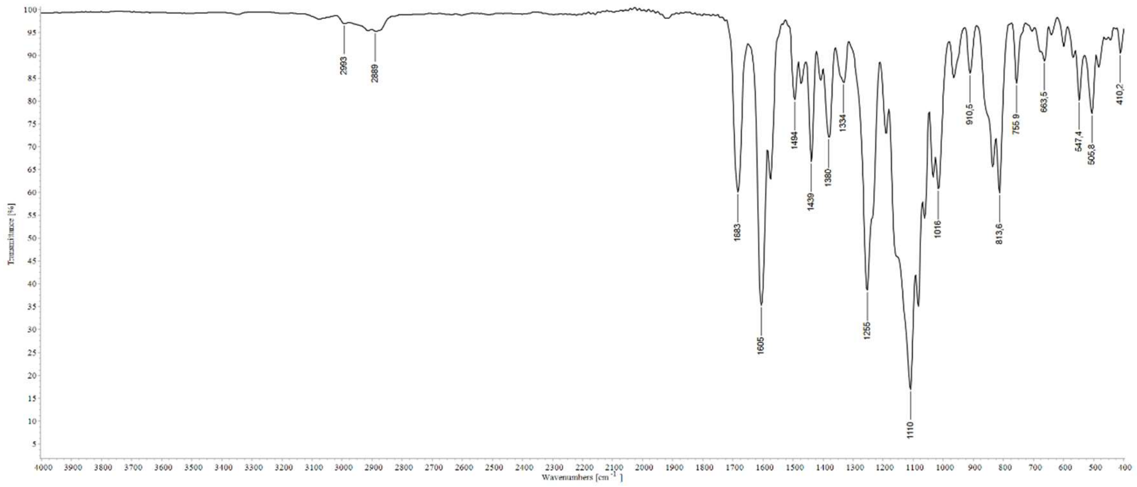

Figure S29: Infrared spectrum υmax of compound **9**.

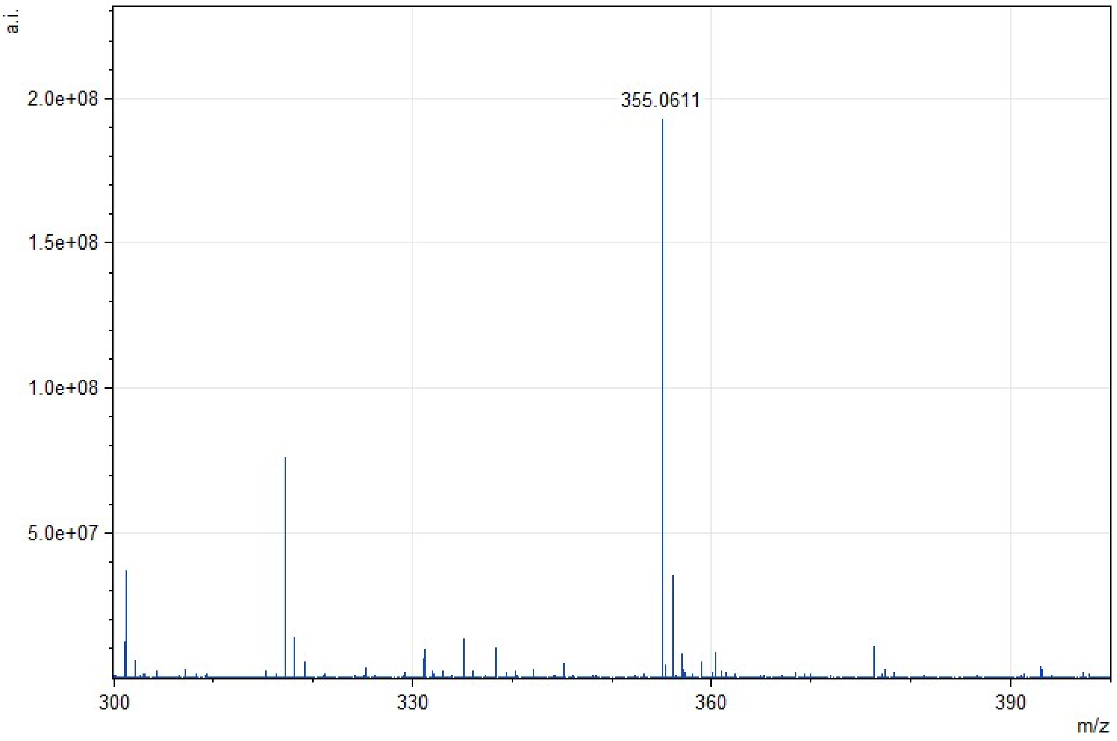

Figure S30: HRMS spectrum of compound **9**.

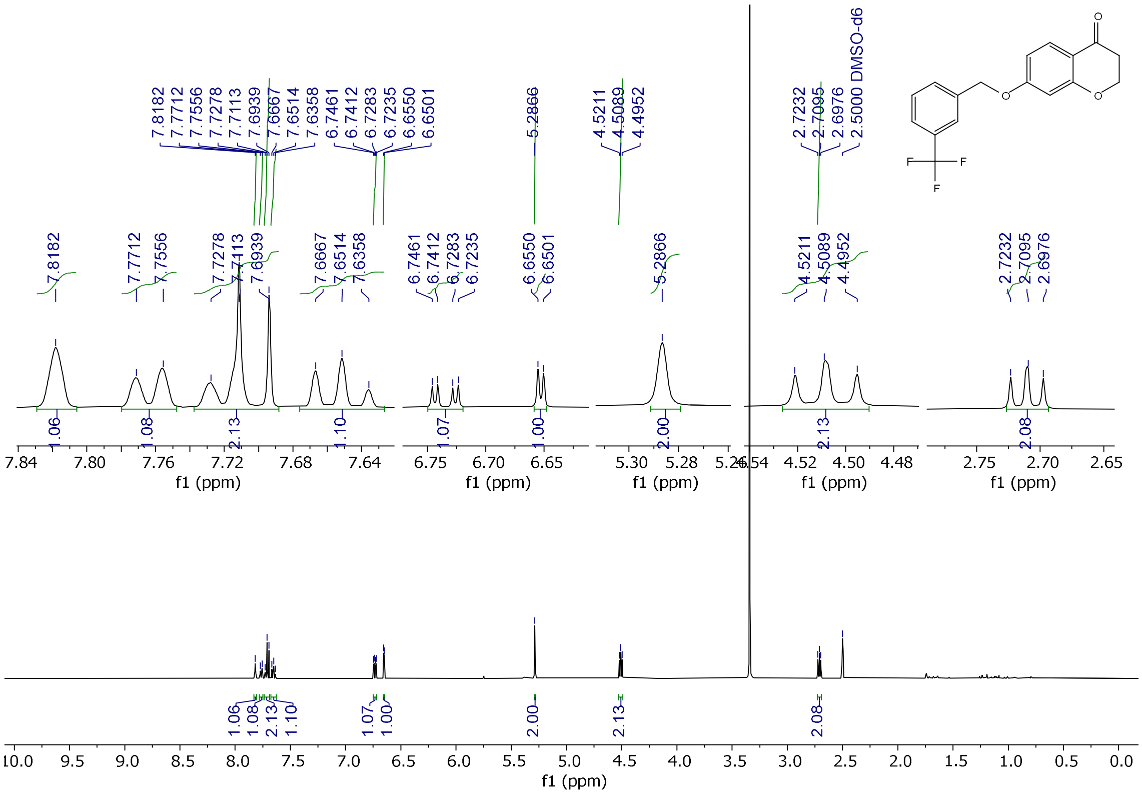

Figure S31: ^{1}H NMR spectrum (500 MHz, DMSO-d_6) of compound **10**.

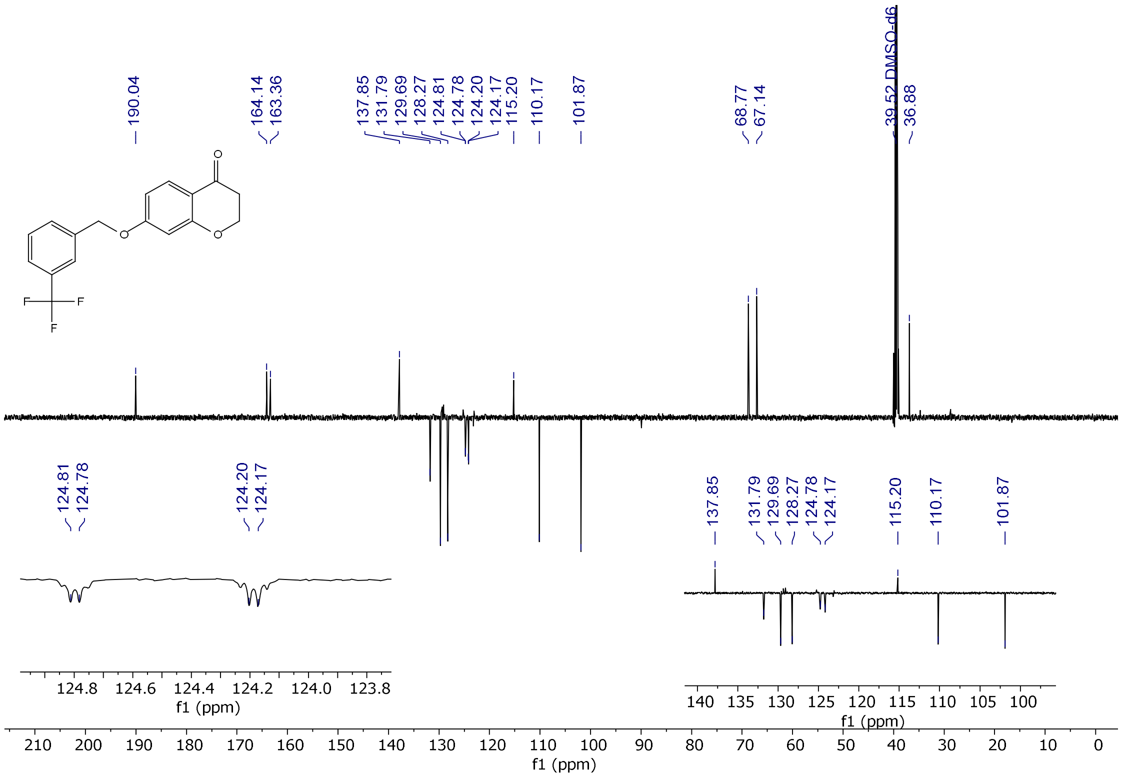

Figure S32: ^{13}C NMR-APT spectrum (125 MHz, DMSO-d_6) of compound **10**.

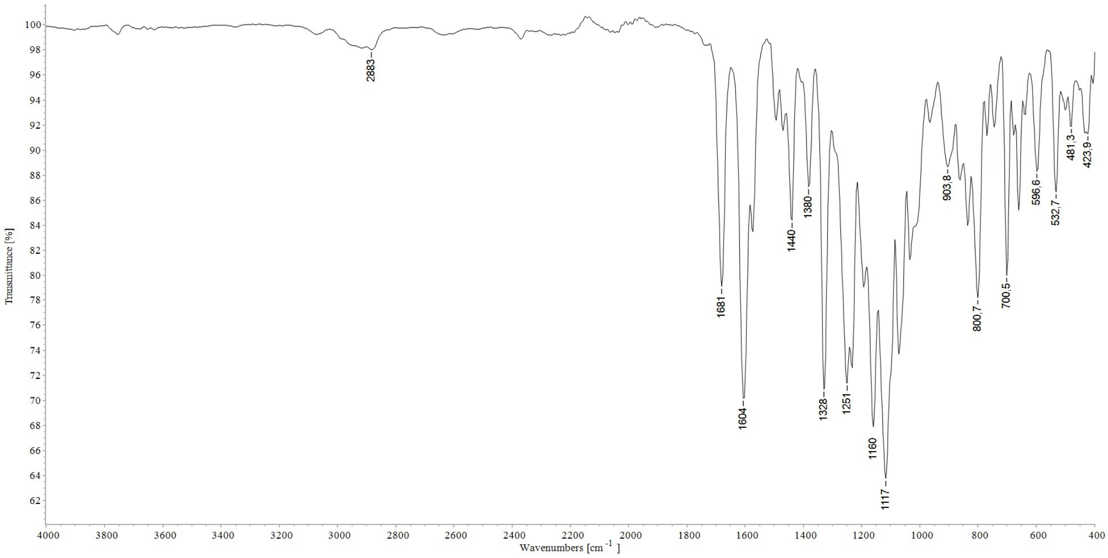

Figure S33: Infrared spectrum *υ*max of compound **10**.

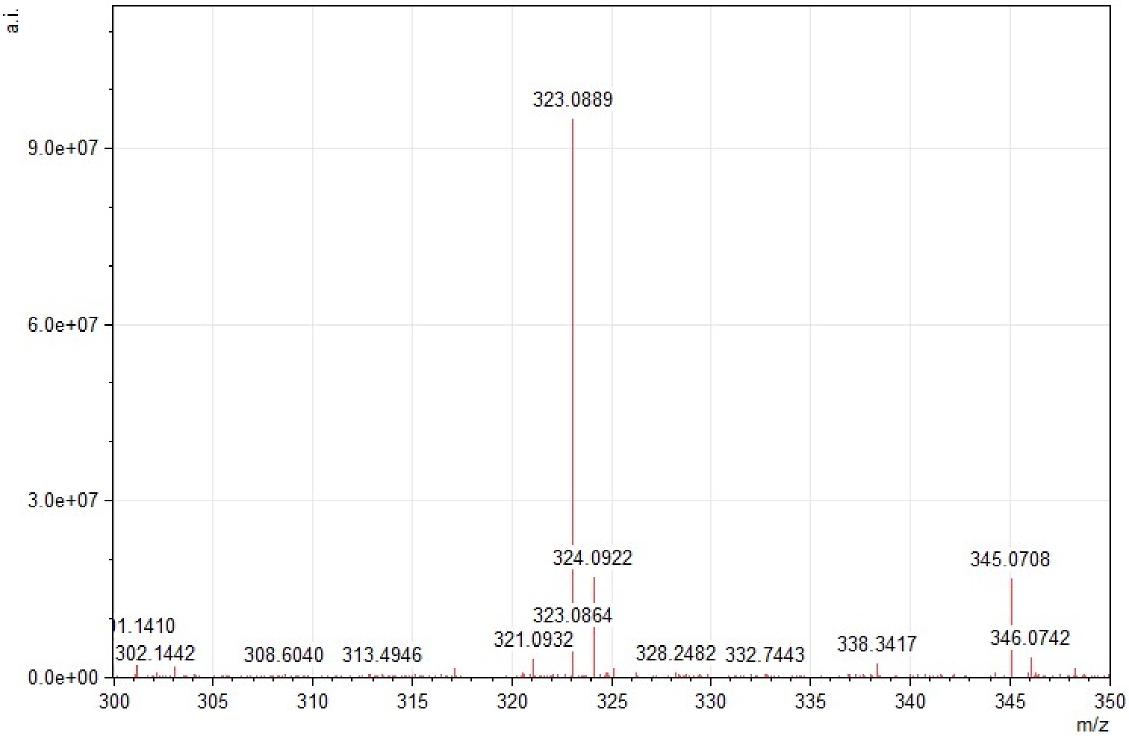

Figure S34: HRMS spectrum of compound **10**.

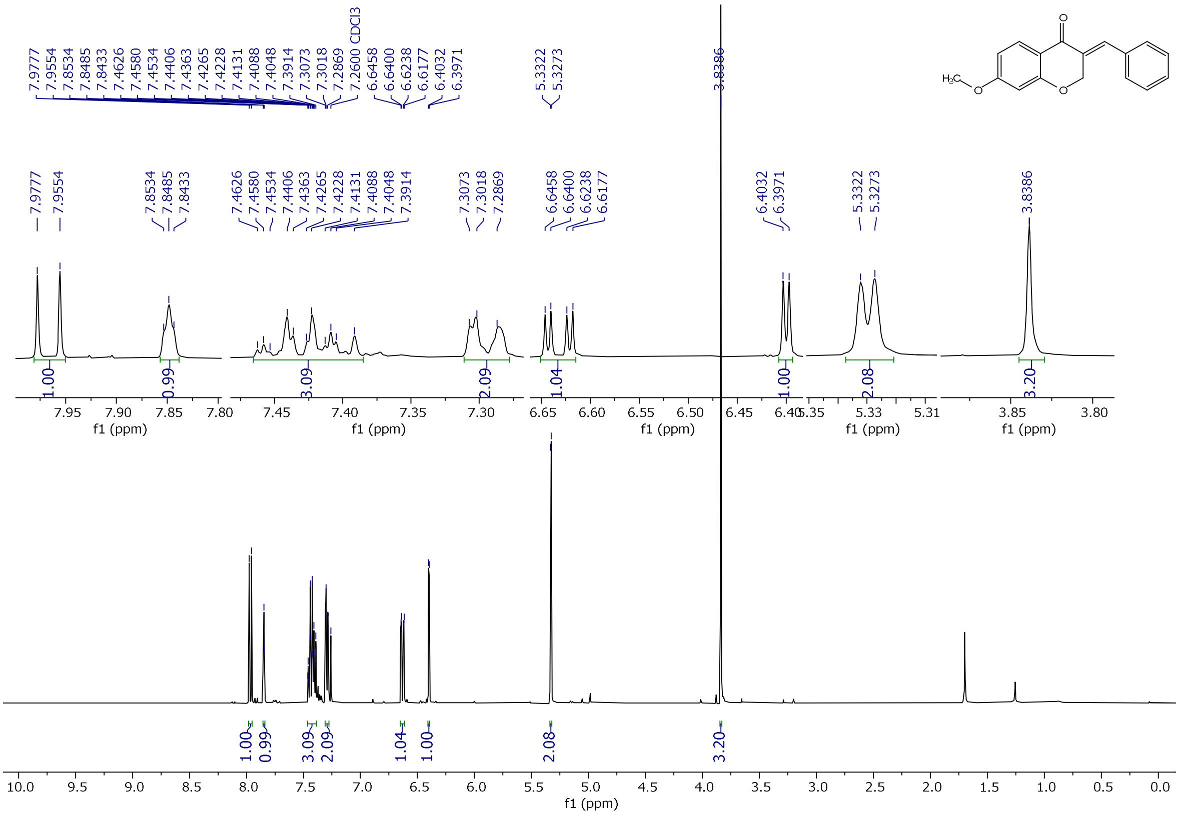

Figure S35: ^{1}H NMR spectrum (400 MHz, $CDCl_3$) of compound **11**.

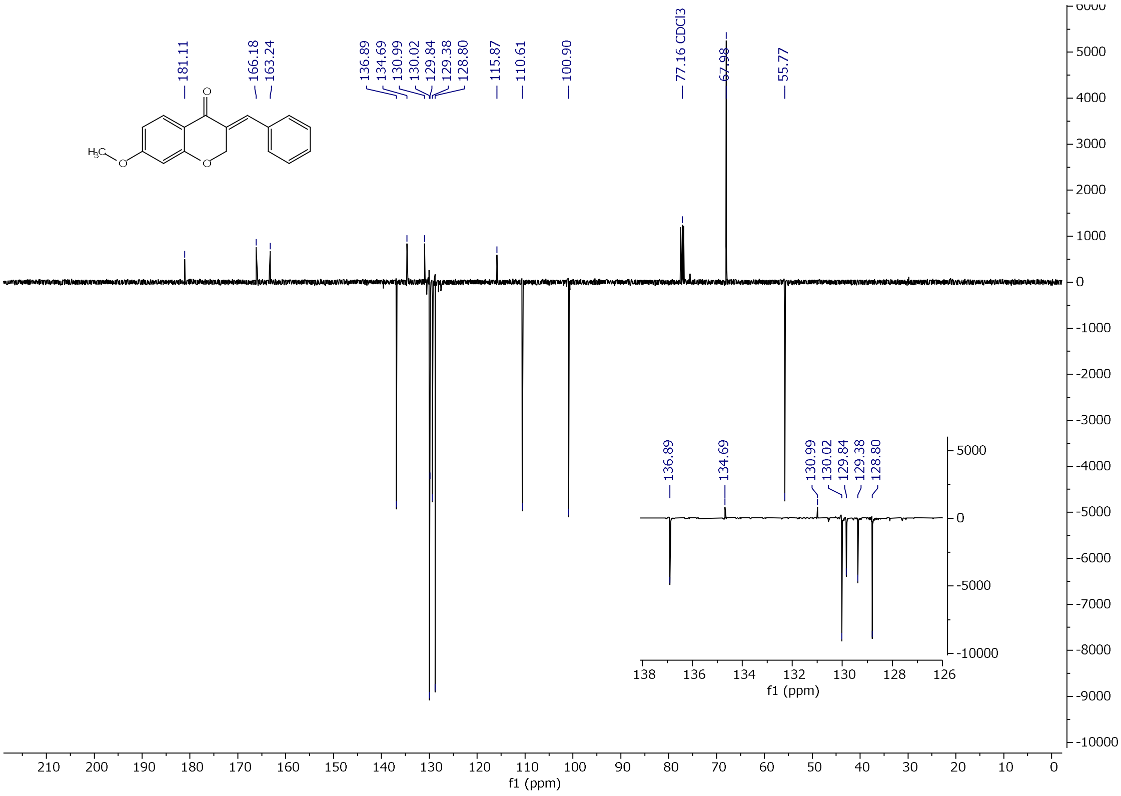

Figure S36: ^{13}C NMR-APT spectrum (100 MHz, $CDCl_3$) of compound **11**.

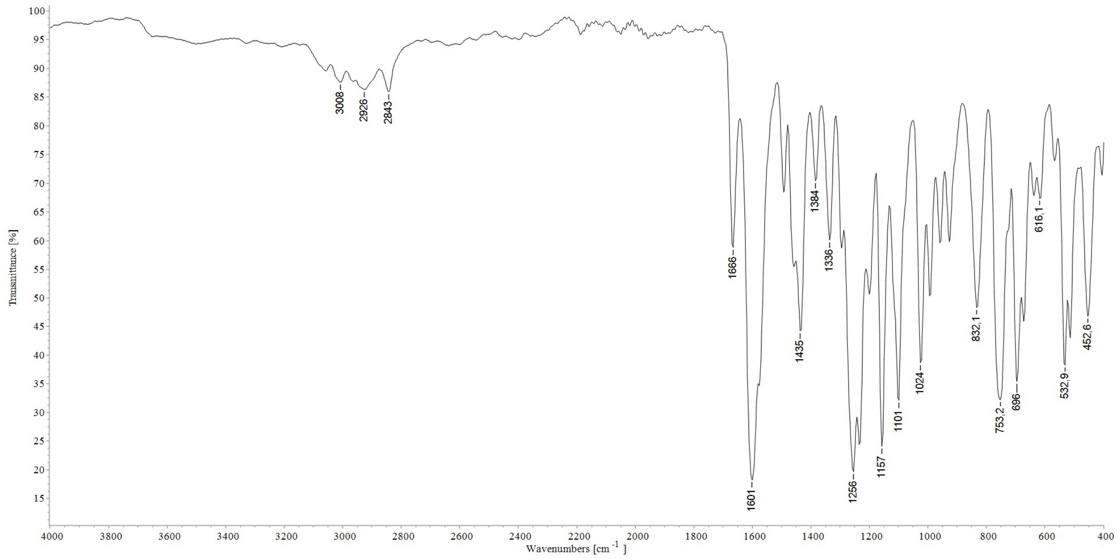

Figure S37: Infrared spectrum υmax of compound **11**.

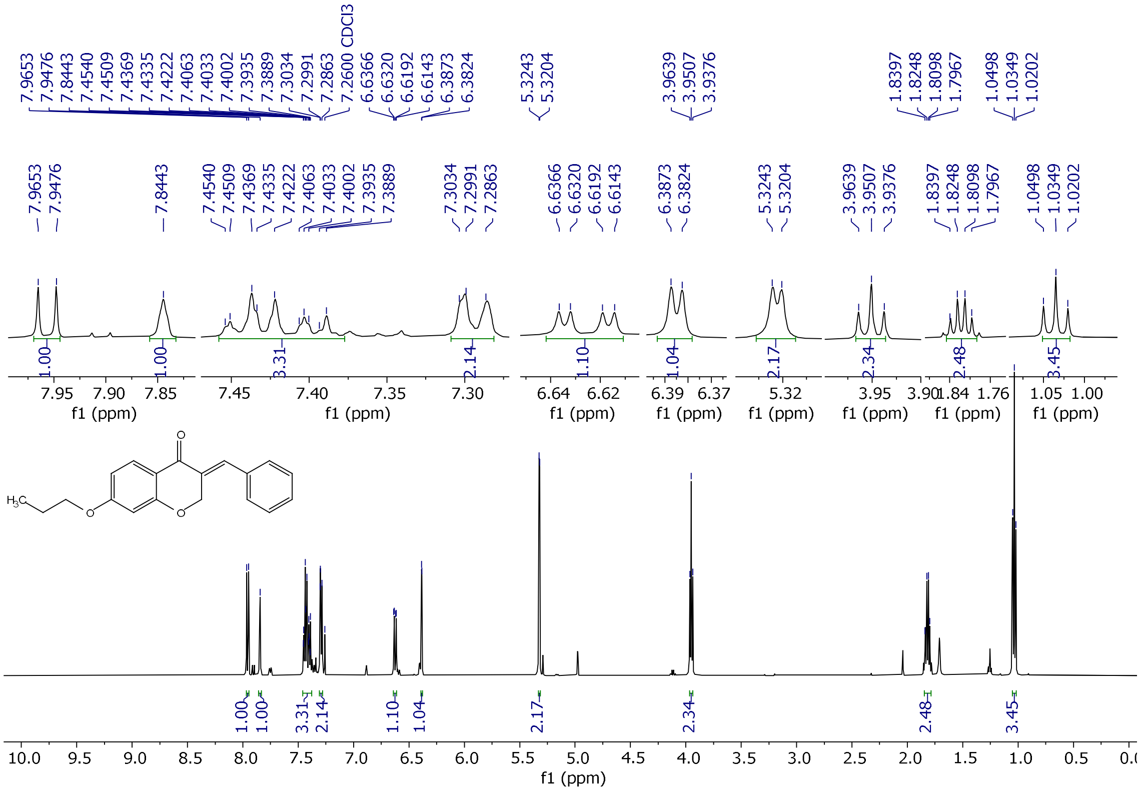

Figure S38: ^{1}H NMR spectrum (500 MHz, $CDCl_3$) of compound **12**.

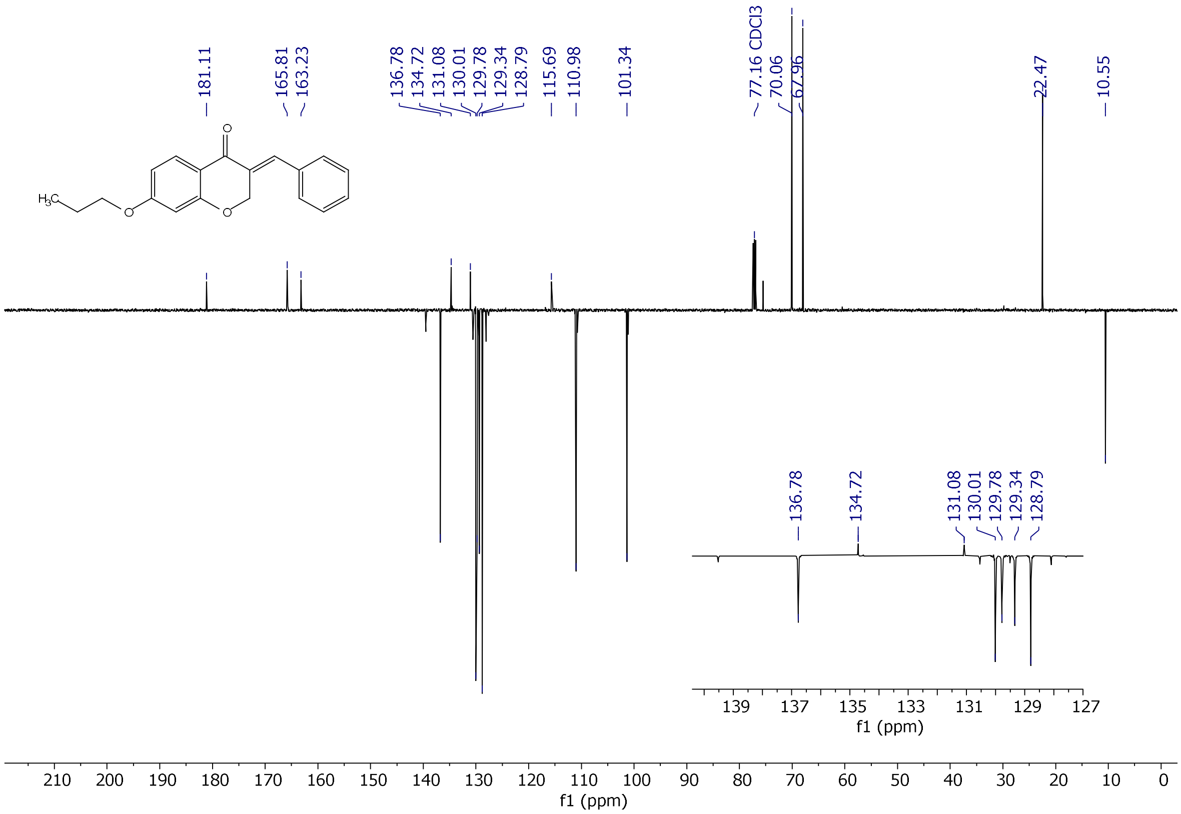

Figure S39: ^{13}C NMR-APT spectrum (125 MHz, $CDCl_3$) of compound **12**.

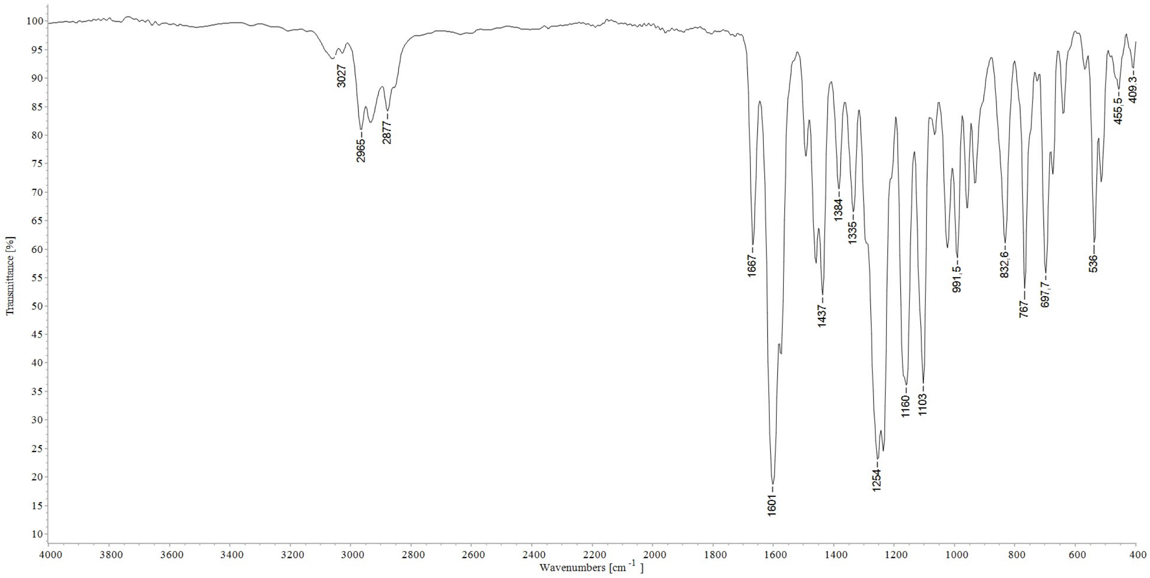

Figure S40: Infrared spectrum υmax of compound **12**.

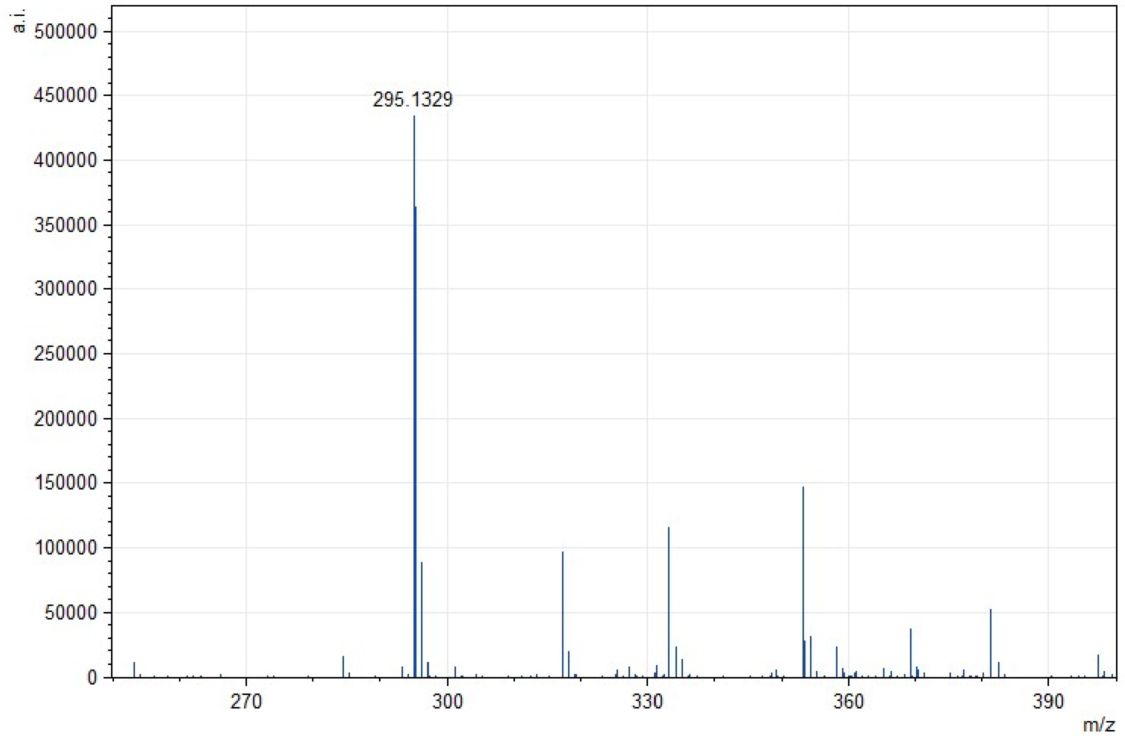

Figure S41: HRMS spectrum of compound **12**.

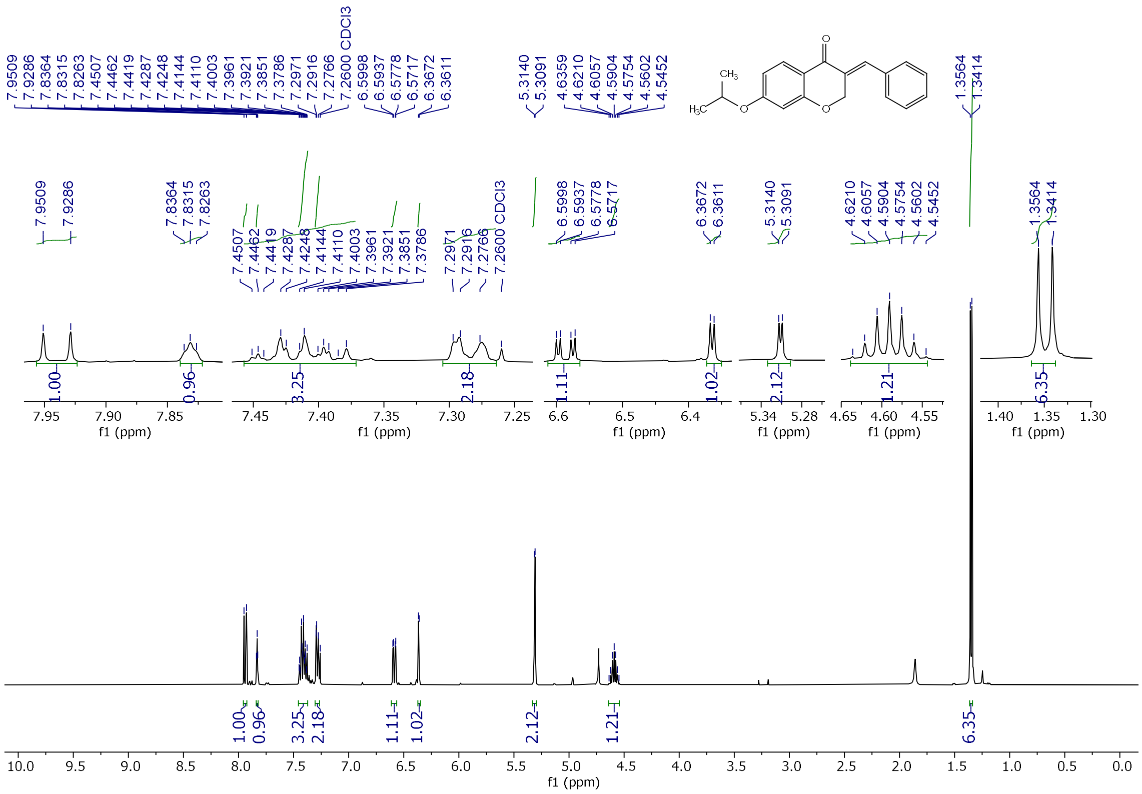

Figure S42: ^{1}H NMR spectrum (400 MHz, $CDCl_3$) of compound **13**.

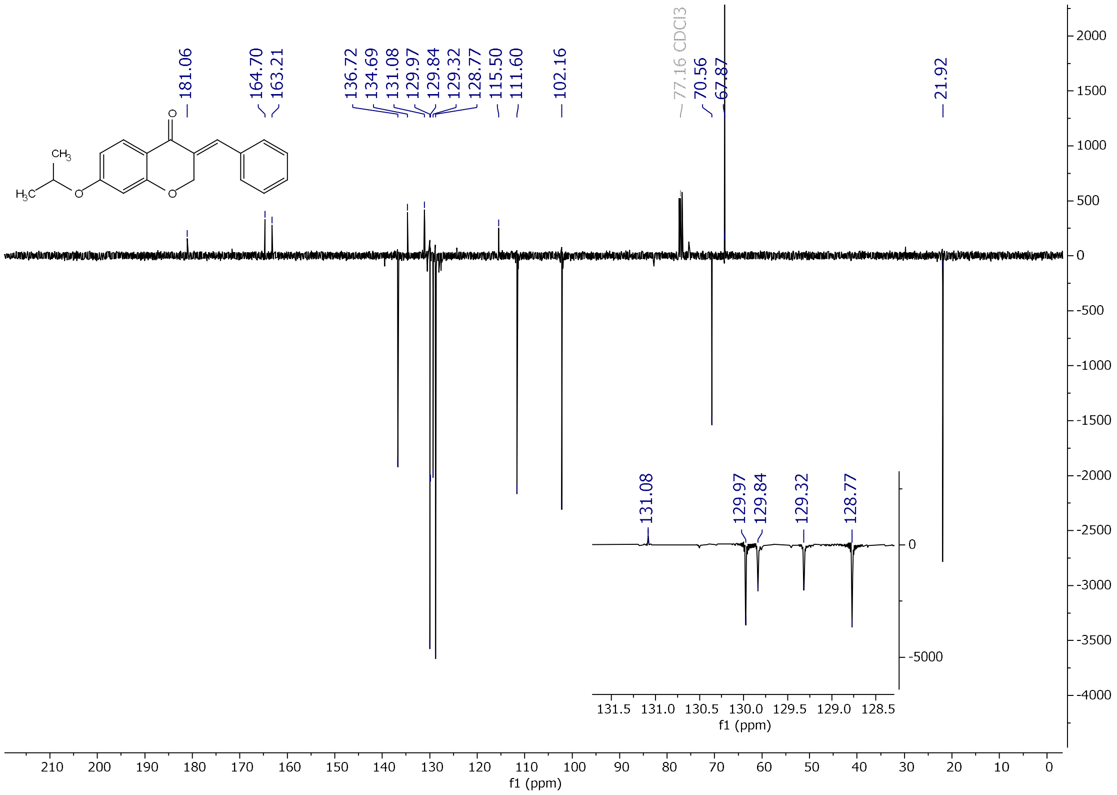

Figure S43: ^{13}C NMR-APT spectrum (100 MHz, $CDCl_3$) of compound **13**.

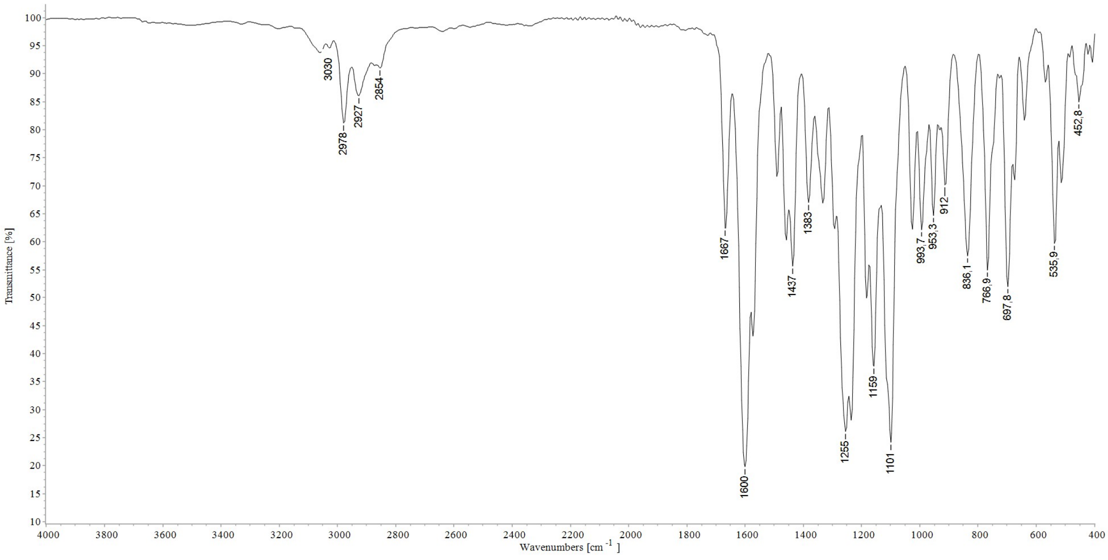

Figure S44: Infrared spectrum υmax of compound **13**.

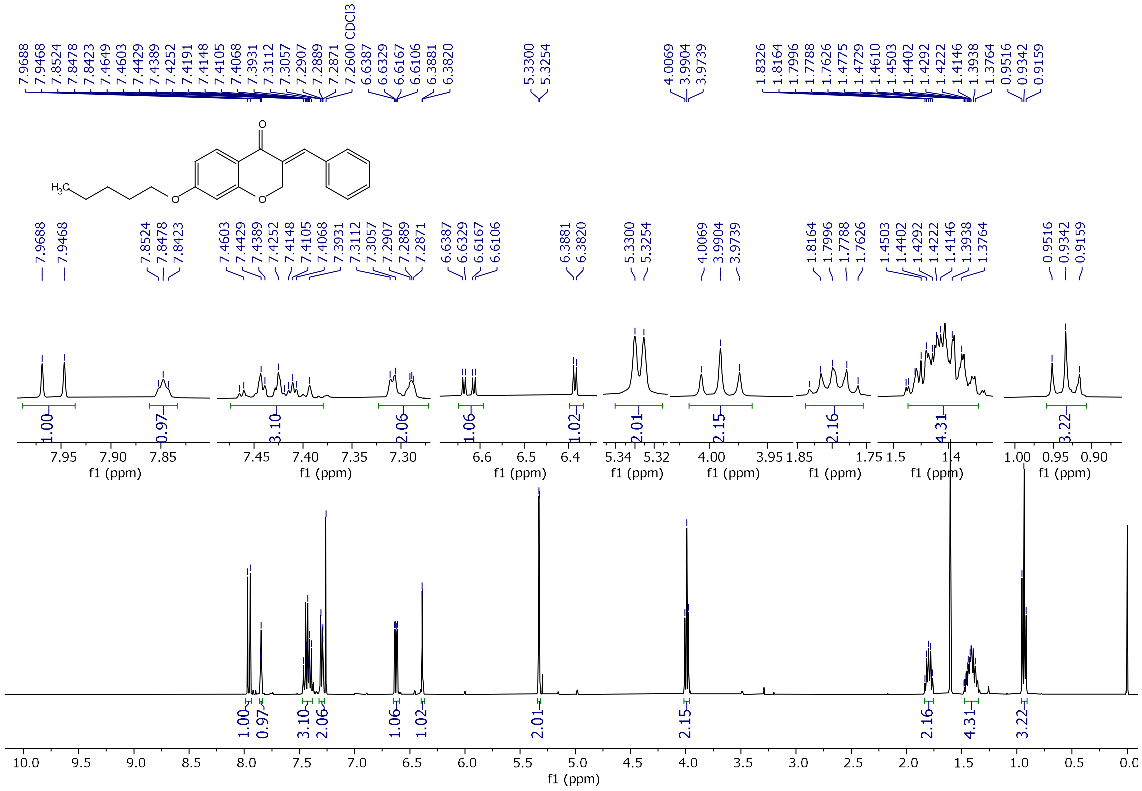

Figure S45: ^{1}H NMR spectrum (400 MHz, $CDCl_3$) of compound **14**.

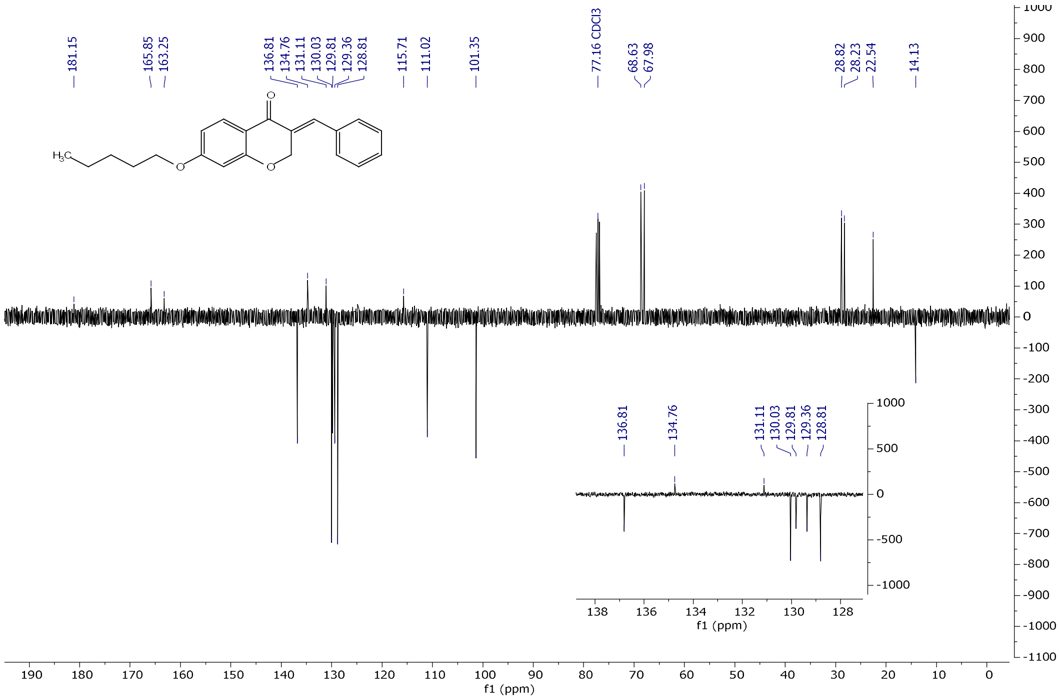

Figure S46: ^{13}C NMR-APT spectrum (100 MHz, $CDCl_3$) of compound **14**.

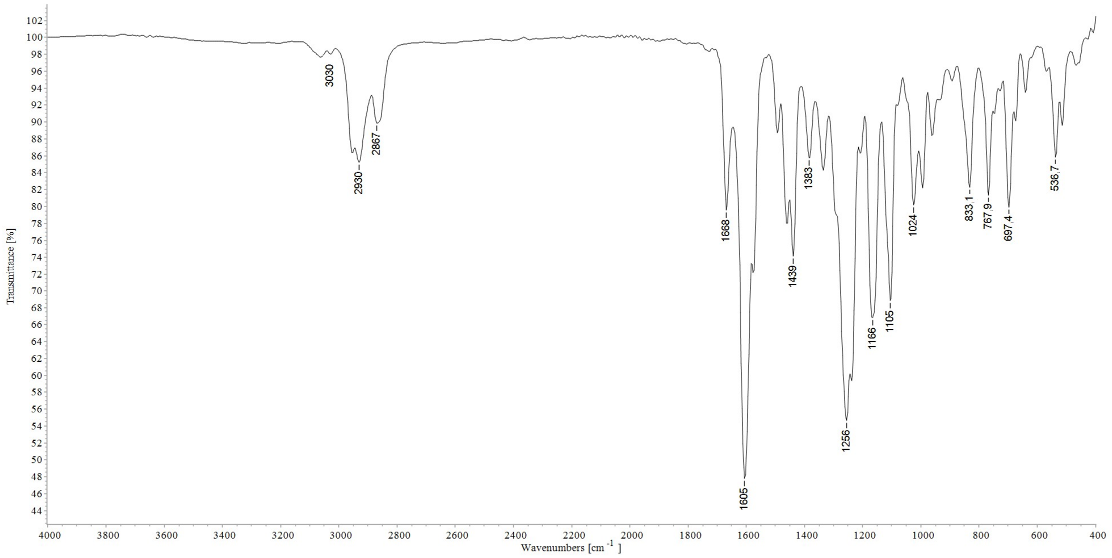

Figure S47: Infrared spectrum υmax of compound **14**.

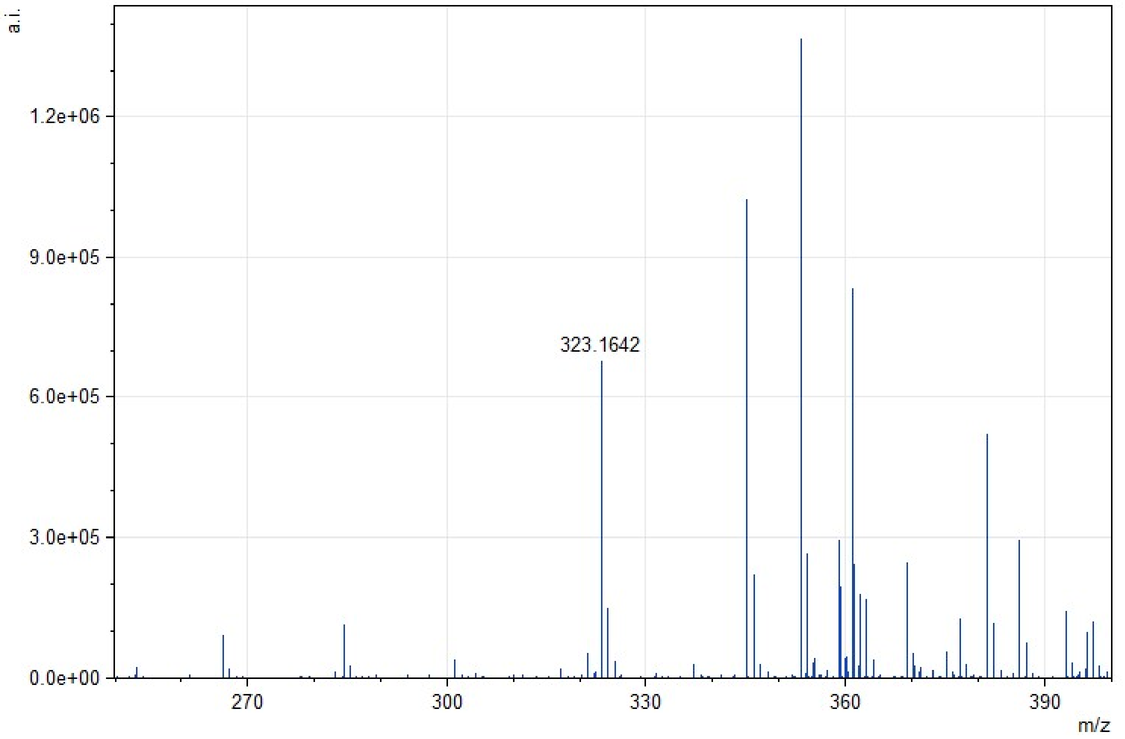

Figure S48: HRMS spectrum of compound **14**.

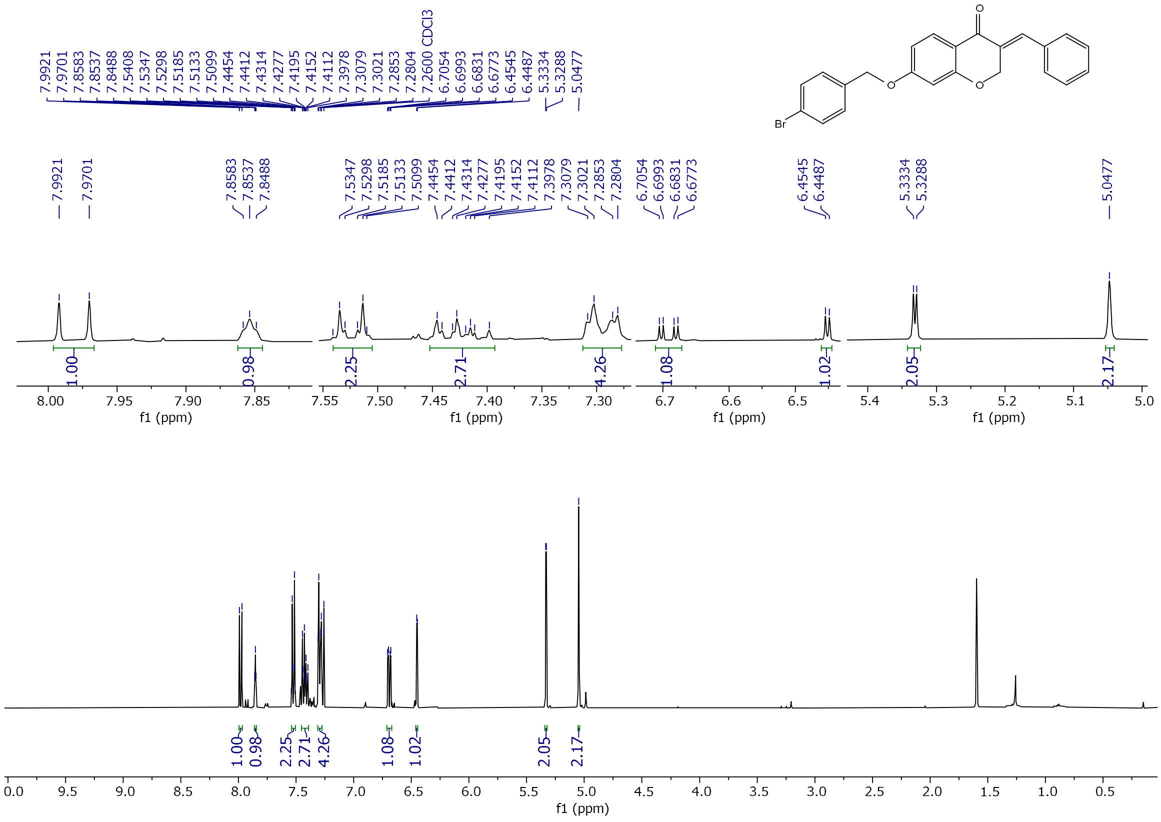

Figure S49: ^{1}H NMR spectrum (400 MHz, $CDCl_3$) of compound **15**.

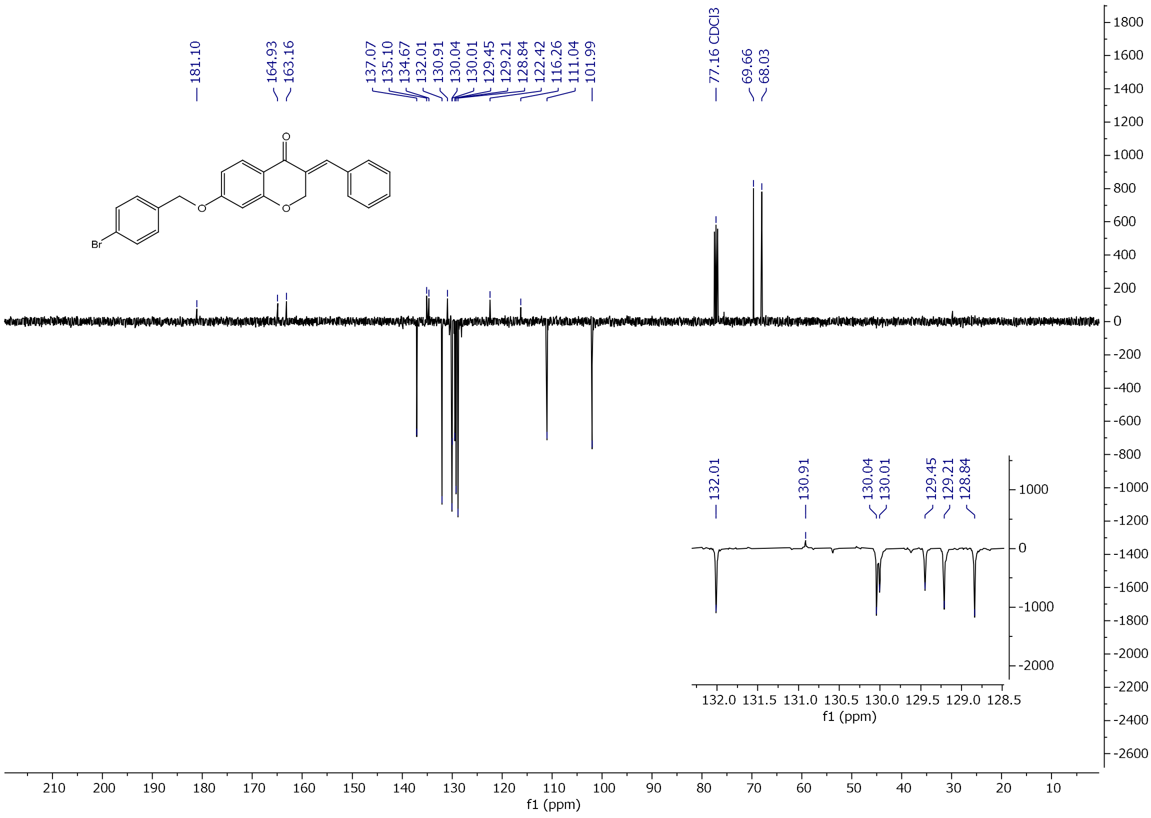

Figure S50: ^{13}C NMR-APT spectrum (100 MHz, $CDCl_3$) of compound **15**.

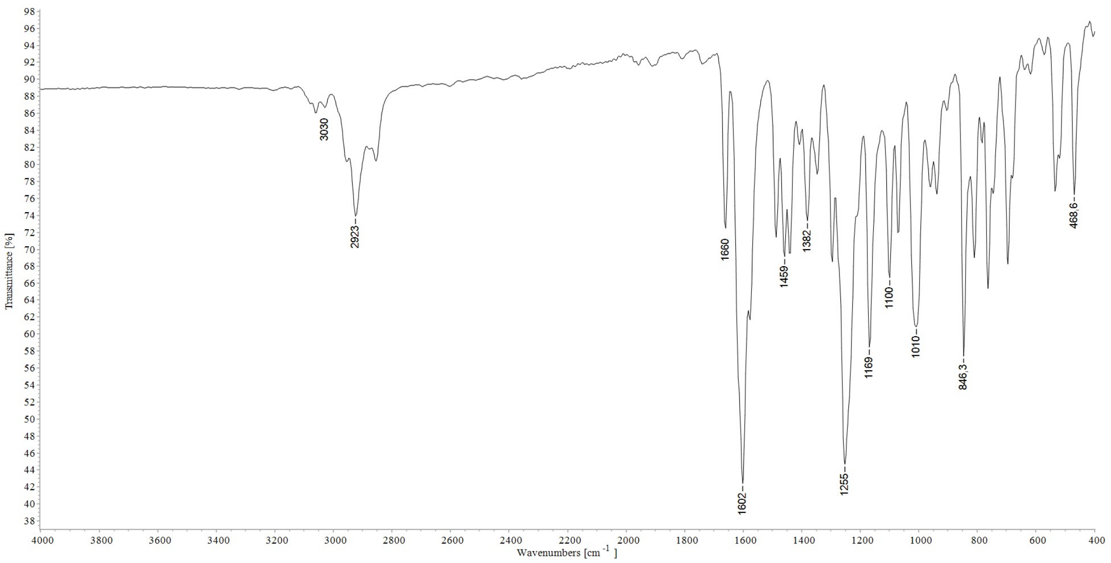

Figure S51: Infrared spectrum υmax of compound **15**.

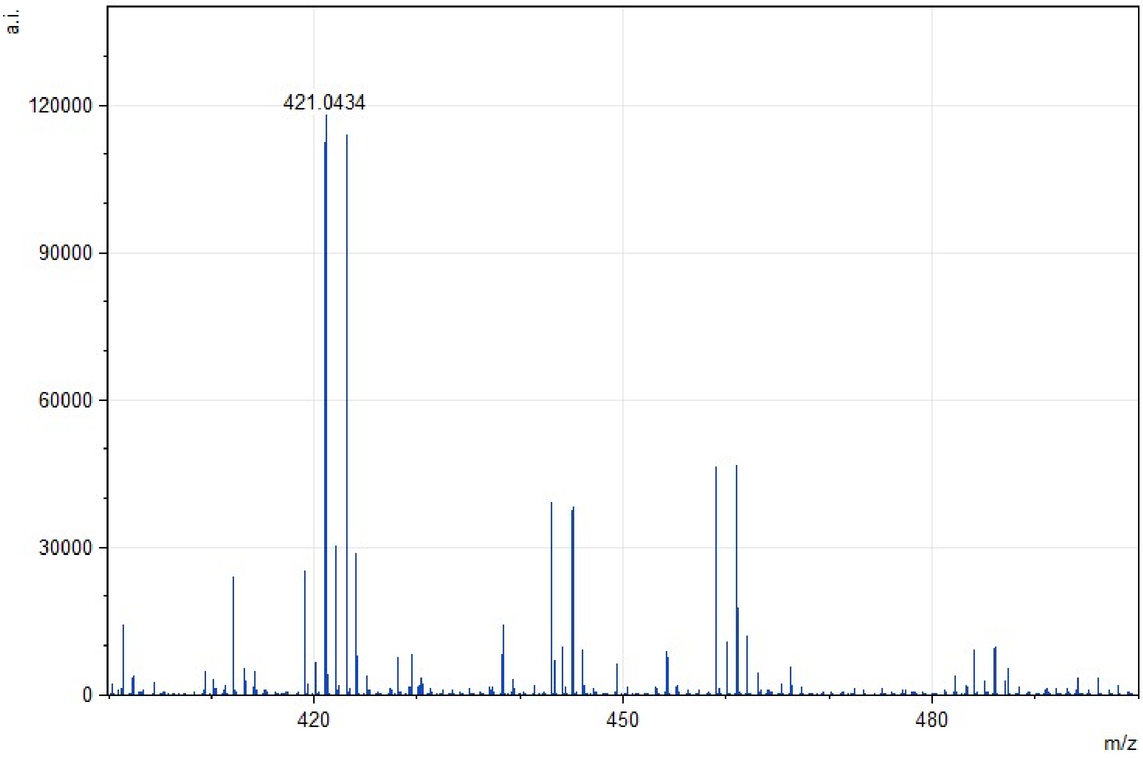

Figure S52: HRMS spectrum of compound **15**.

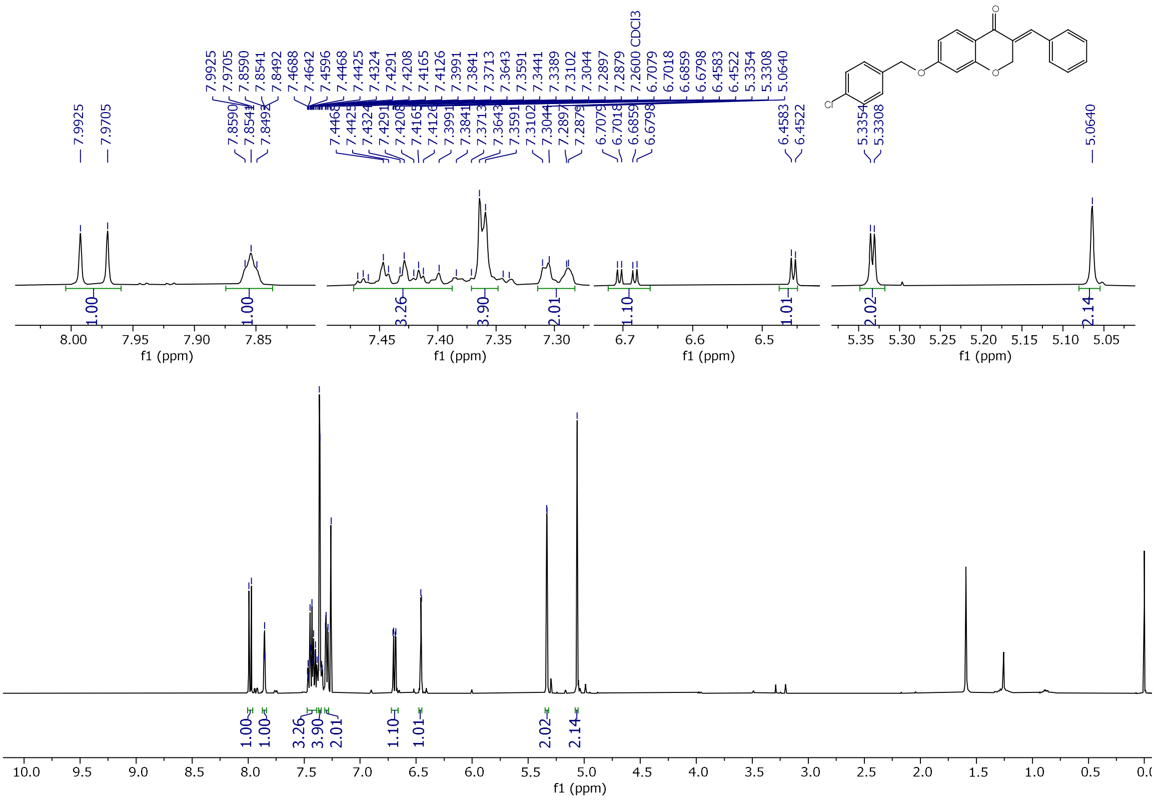

Figure S53: ^{1}H NMR spectrum (400 MHz, $CDCl_3$) of compound **16**.

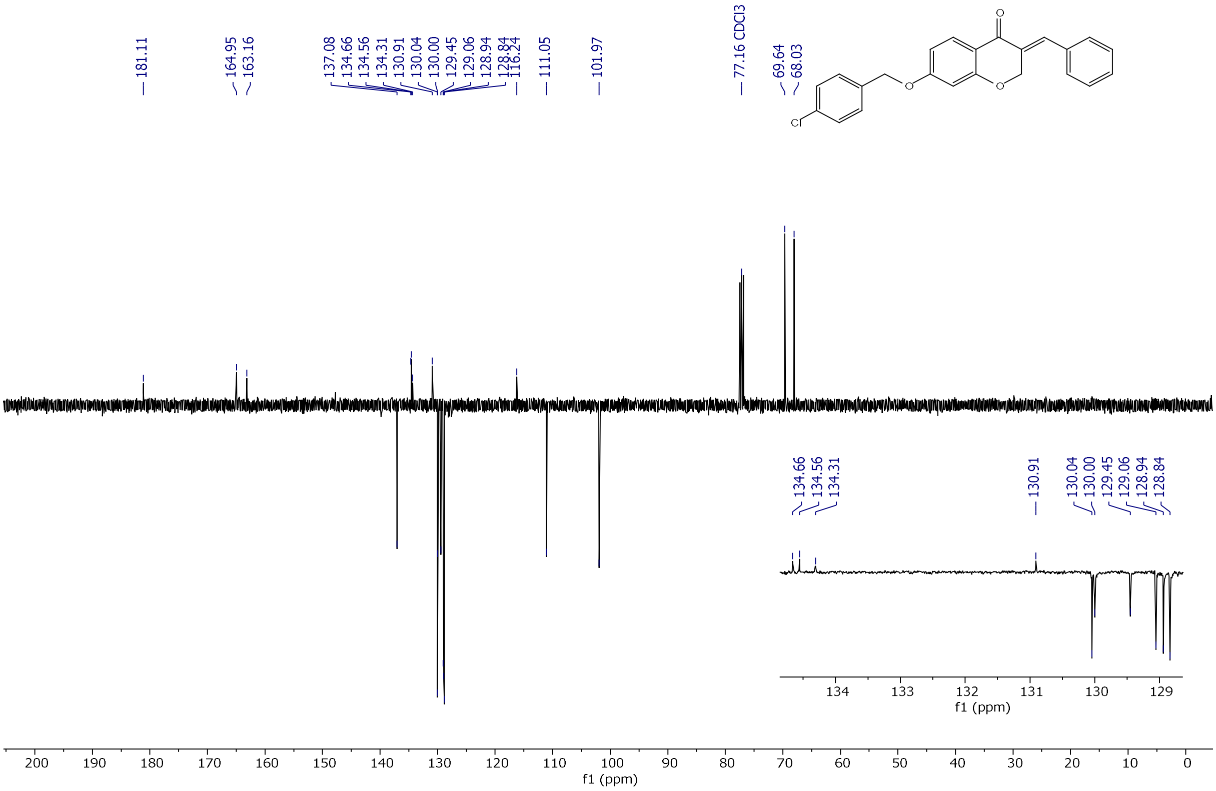

Figure S54: ^{13}C NMR-APT spectrum (100 MHz, $CDCl_3$) of compound **16**.

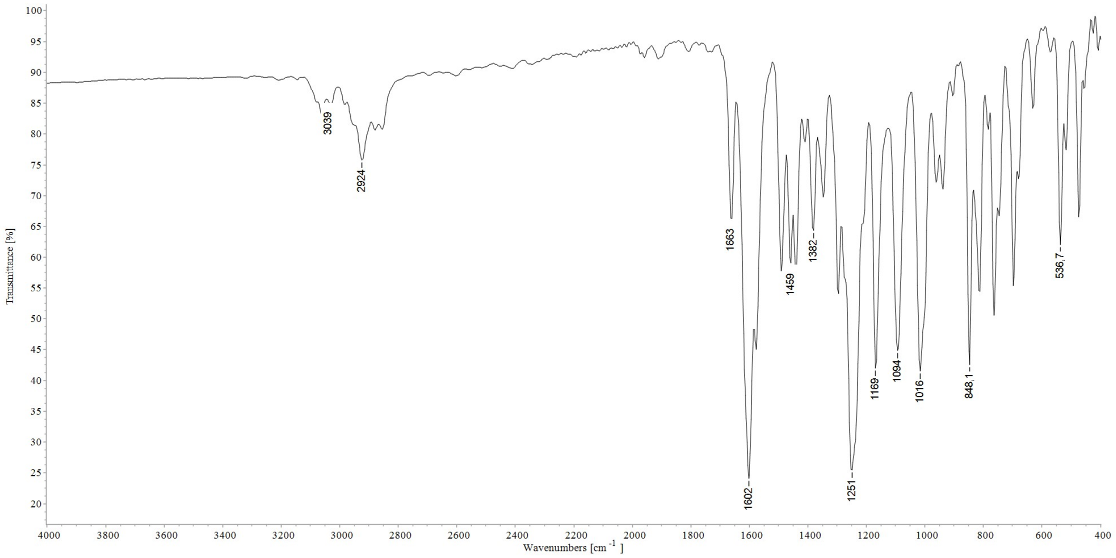

Figure S55: Infrared spectrum υmax of compound **16**.

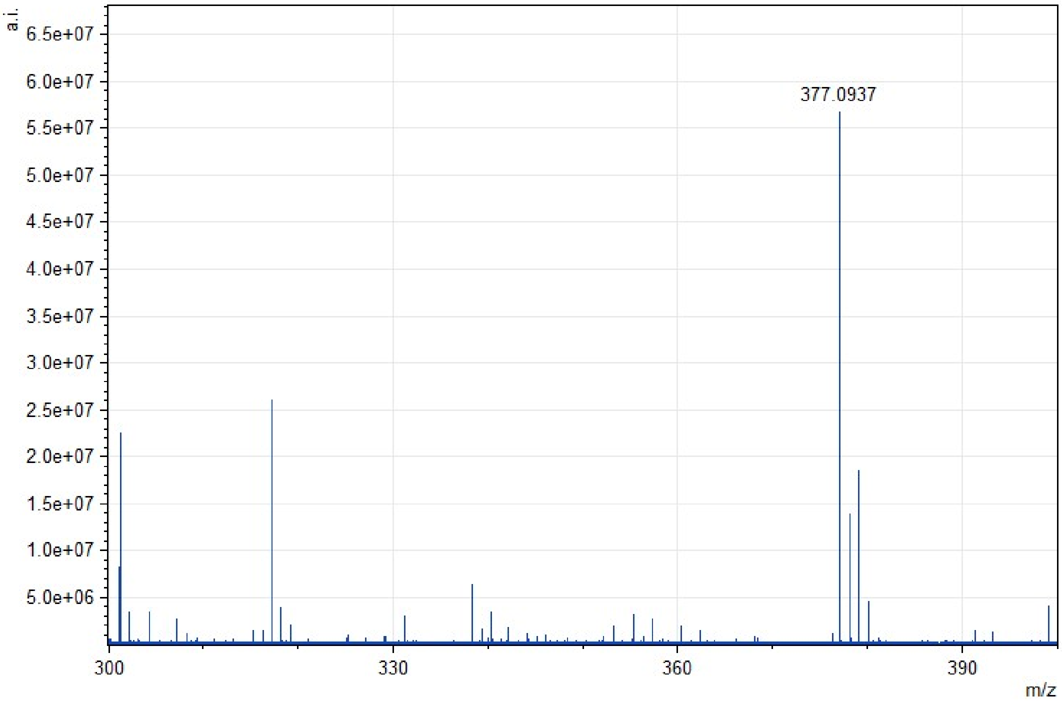

Figure S56: HRMS spectrum of compound **16**.

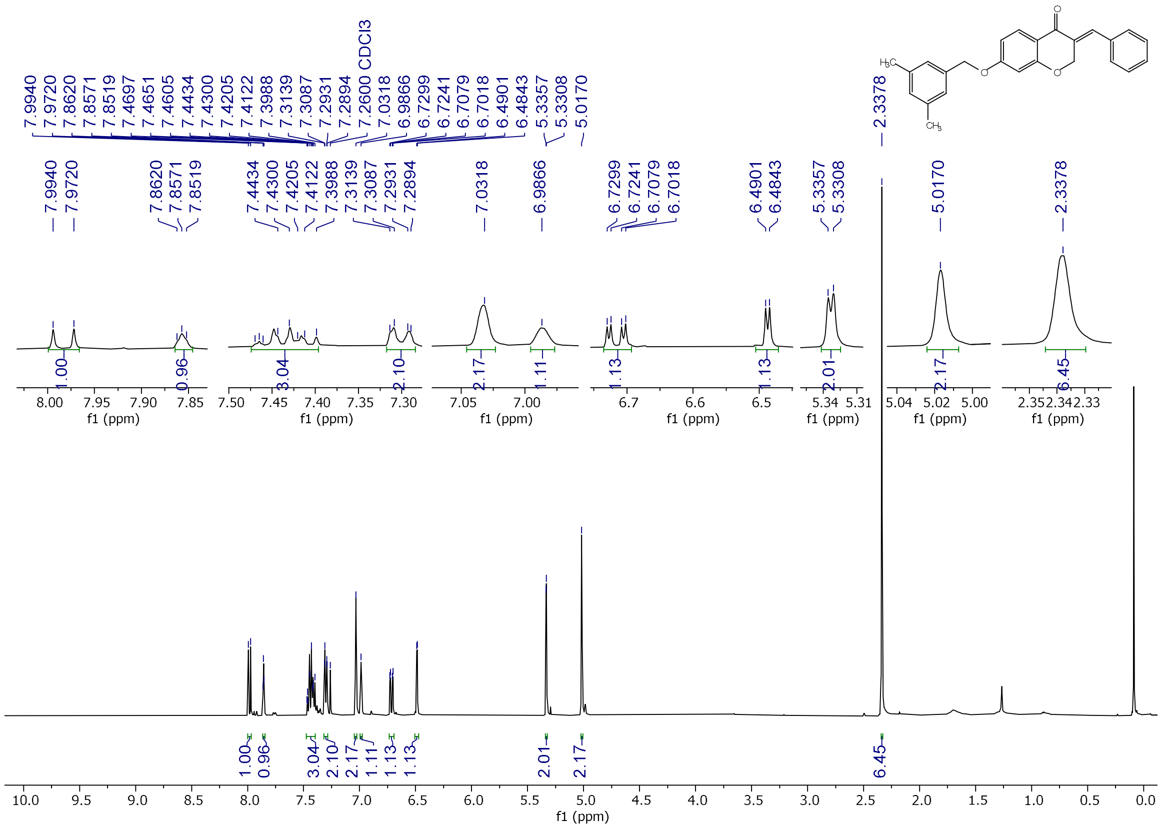

Figure S57: ^{1}H NMR spectrum (400 MHz, $CDCl_3$) of compound **17**.

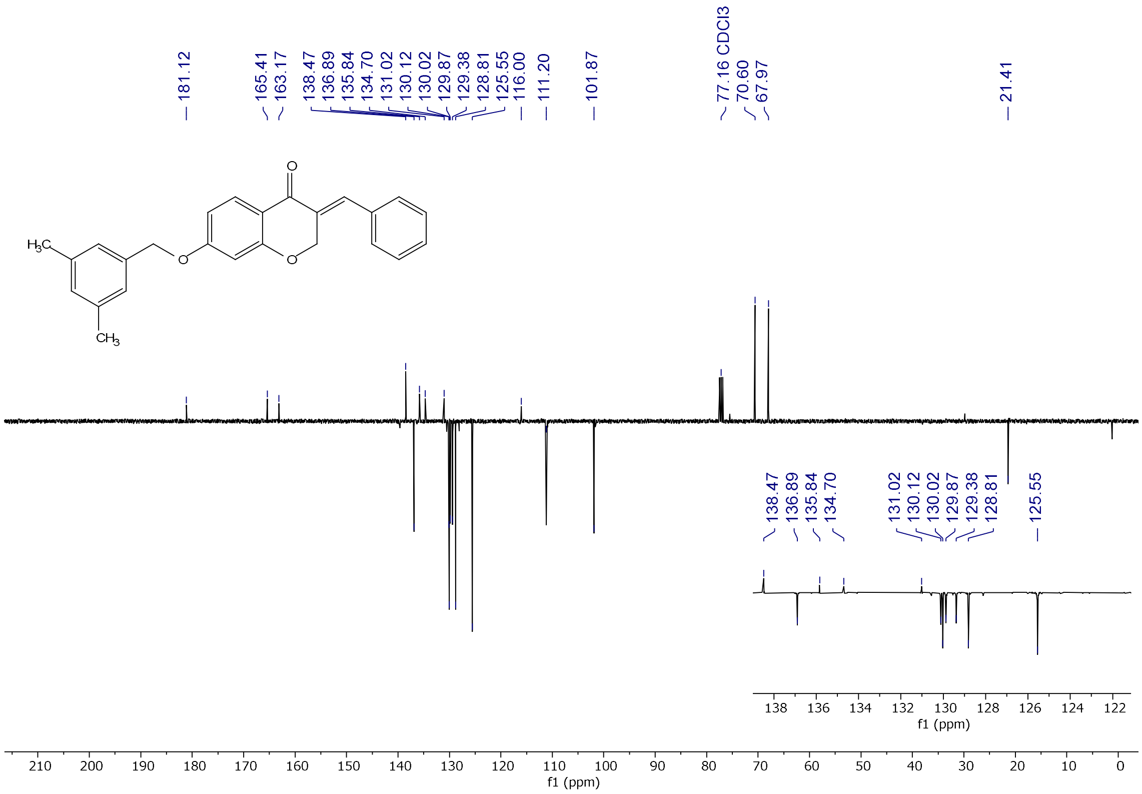

Figure S58: ^{13}C NMR-APT spectrum (100 MHz, $CDCl_3$) of compound **17**.

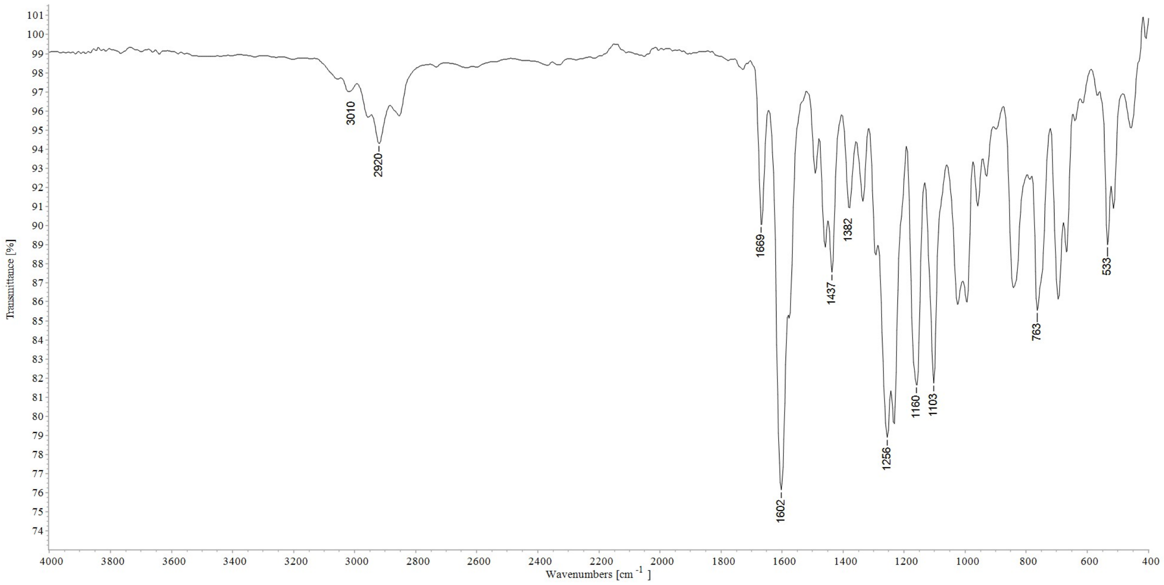

Figure S59: Infrared spectrum υmax of compound **17**.

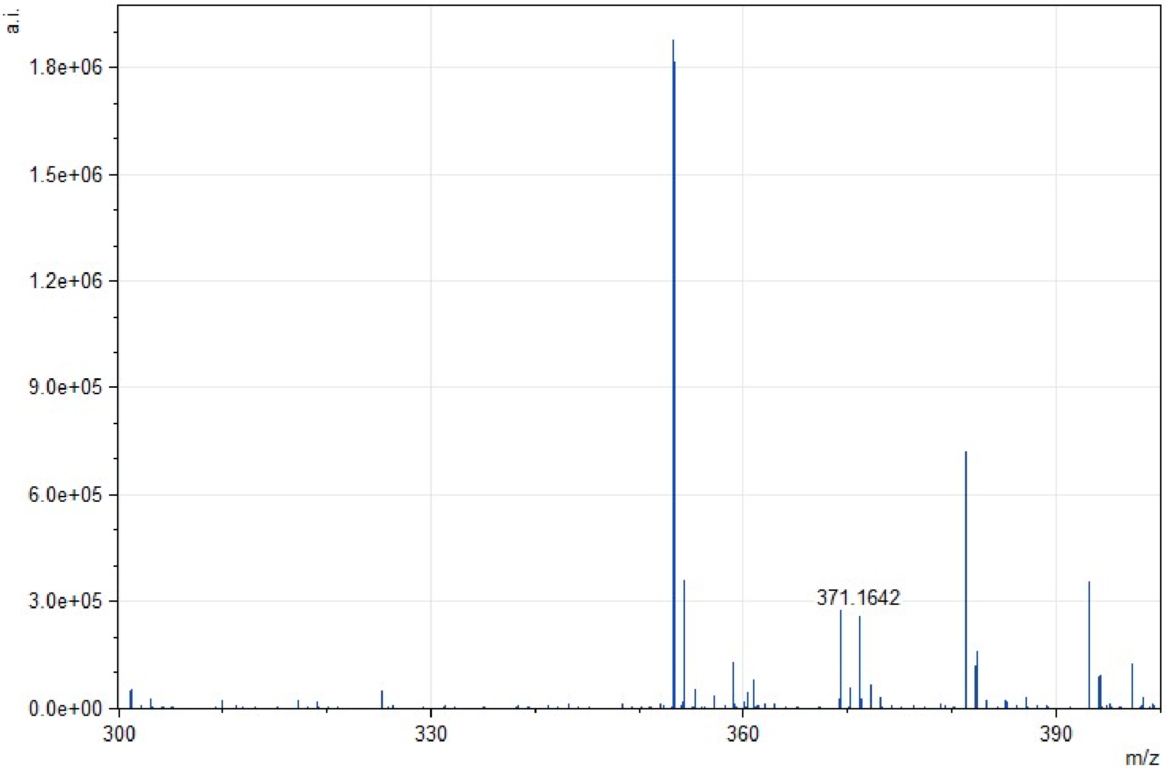

Figure S60: HRMS spectrum of compound **17**.

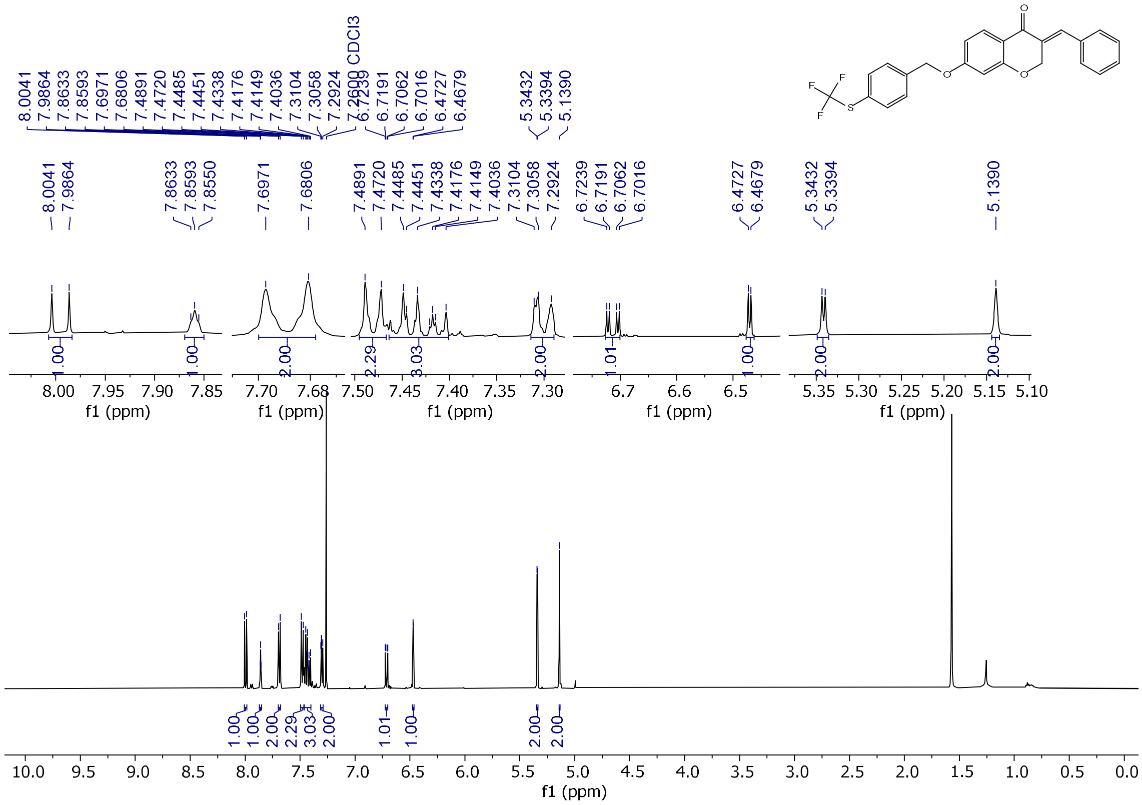

Figure S61: ^{1}H NMR spectrum (500 MHz, $CDCl_3$) of compound **18**.

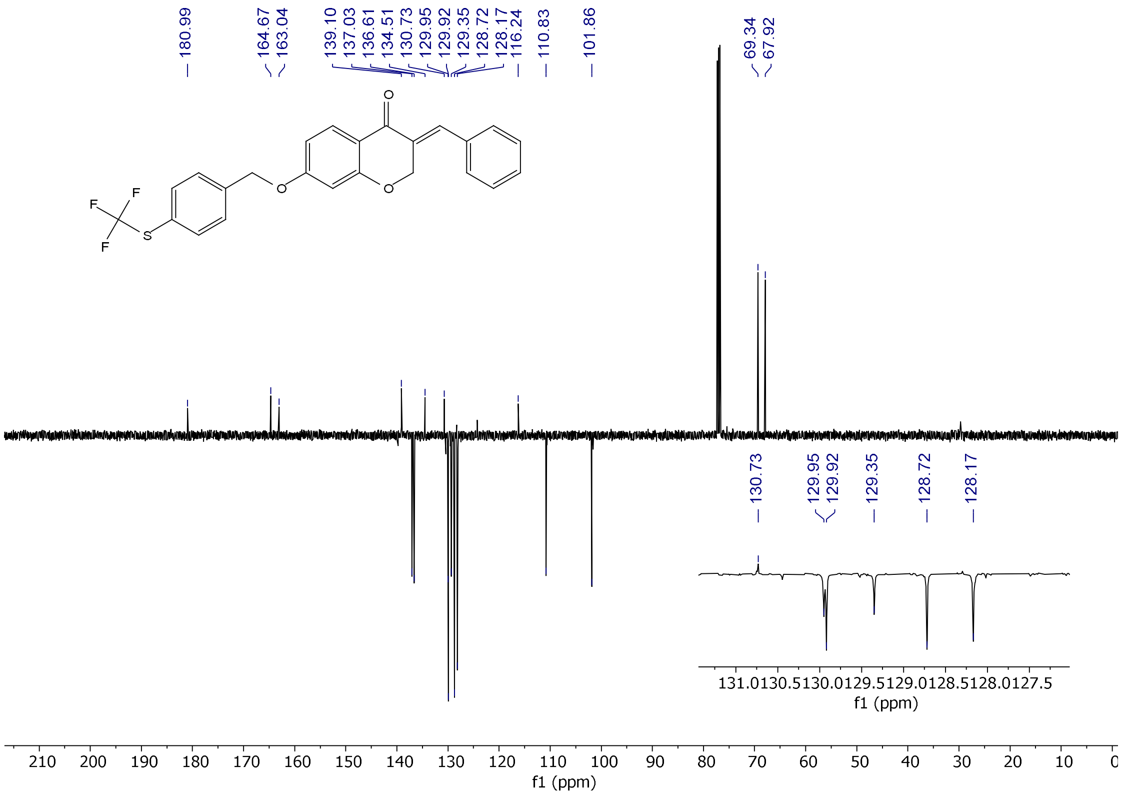

Figure S62: ^{13}C NMR-APT spectrum (125 MHz, $CDCl_3$) of compound **18**.

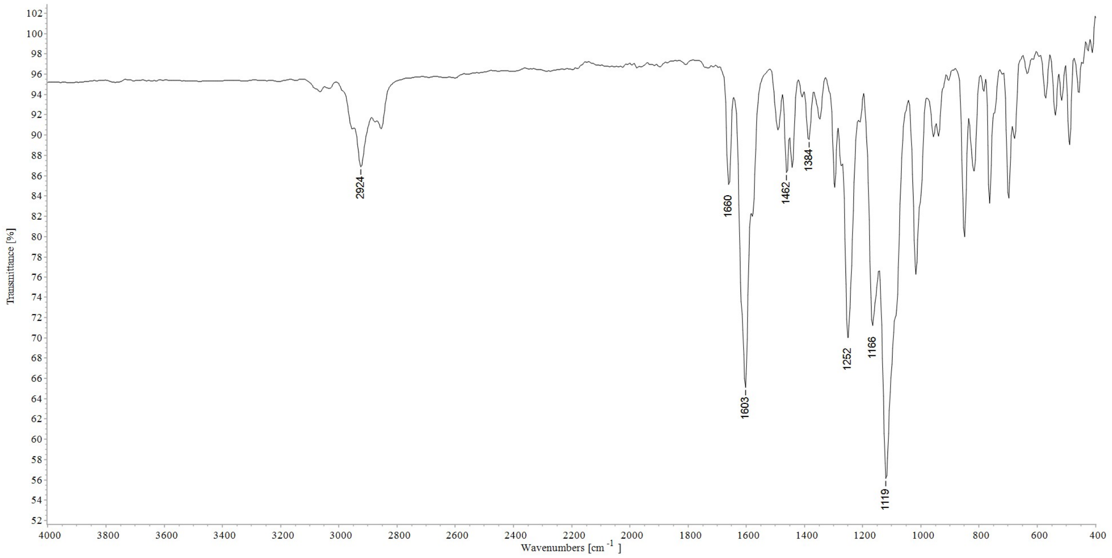

Figure S63: Infrared spectrum υmax of compound **18**.

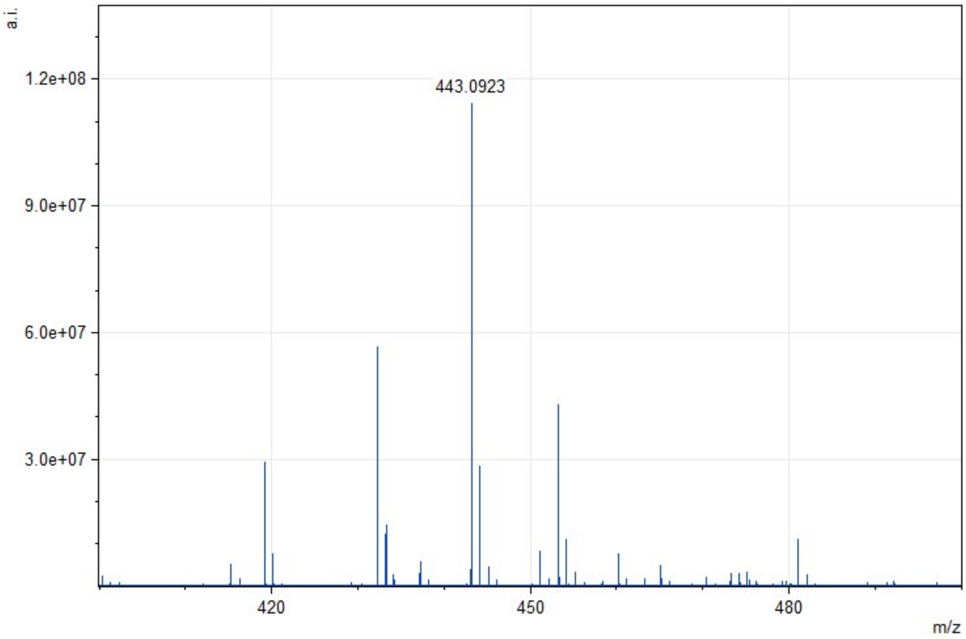

Figure S64: HRMS spectrum of compound **18**.

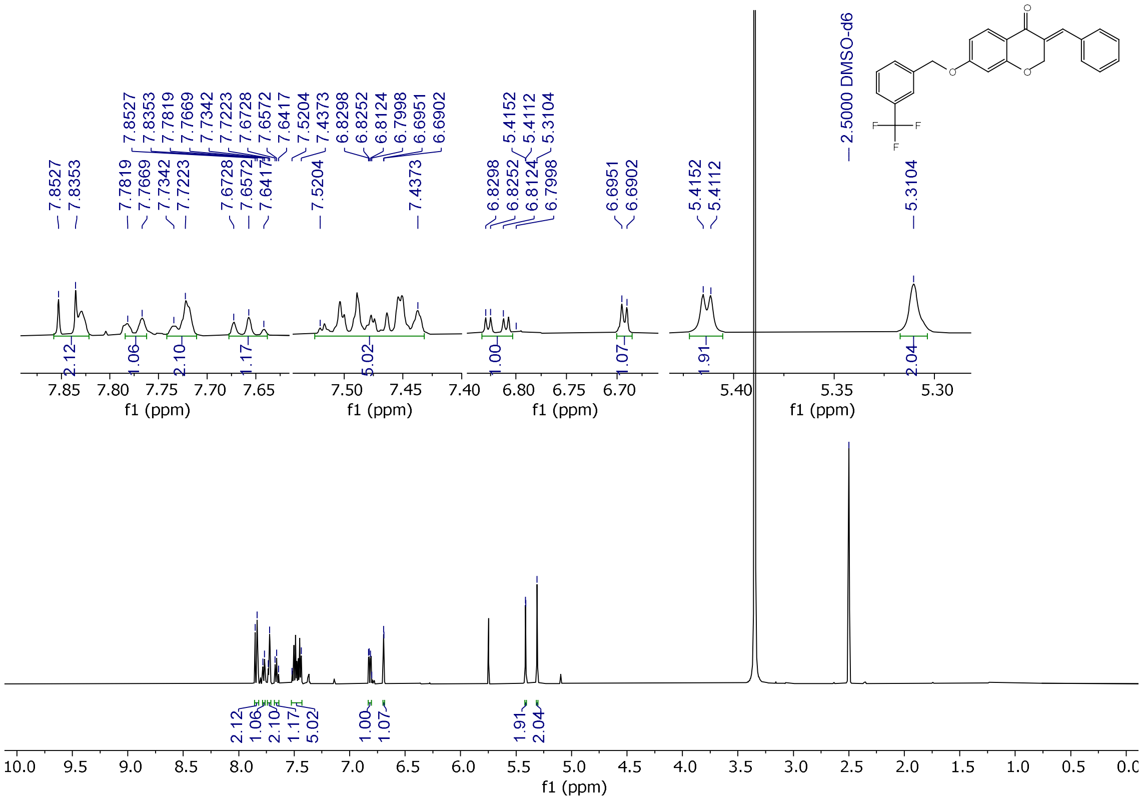

Figure S65: ^{1}H NMR spectrum (500 MHz, DMSO-d6) of compound **19**.

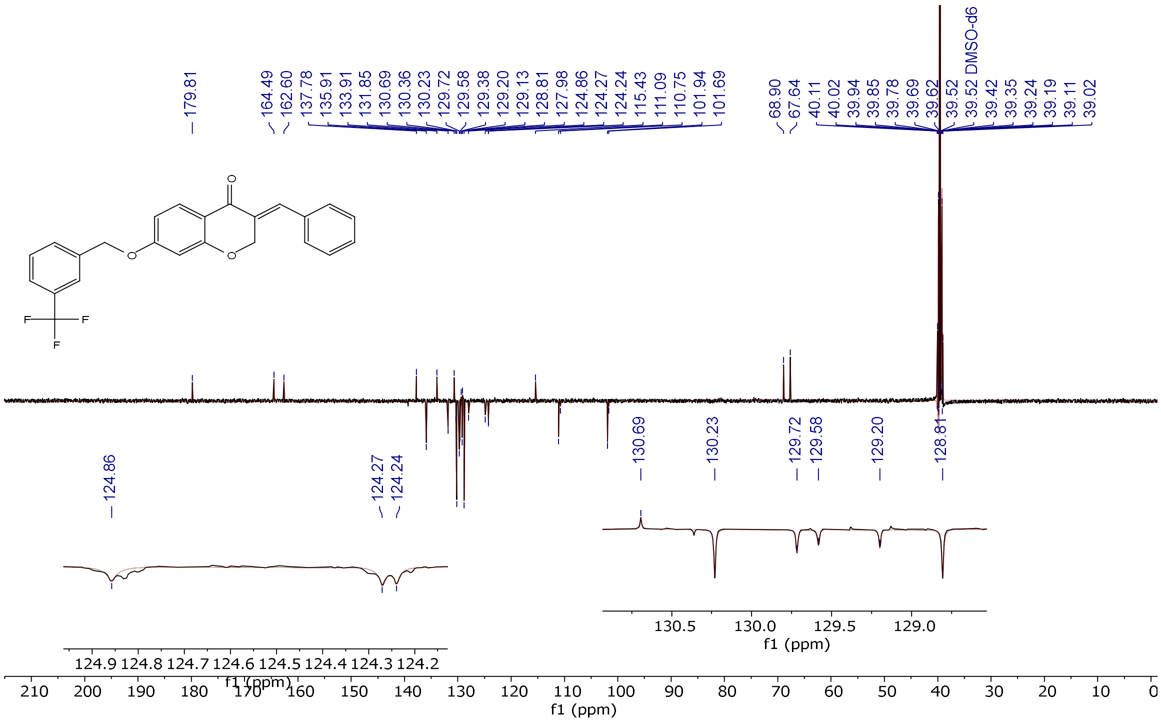

Figure S66: ^{13}C NMR-APT spectrum (125 MHz, DMSO-d6) of compound **19**.

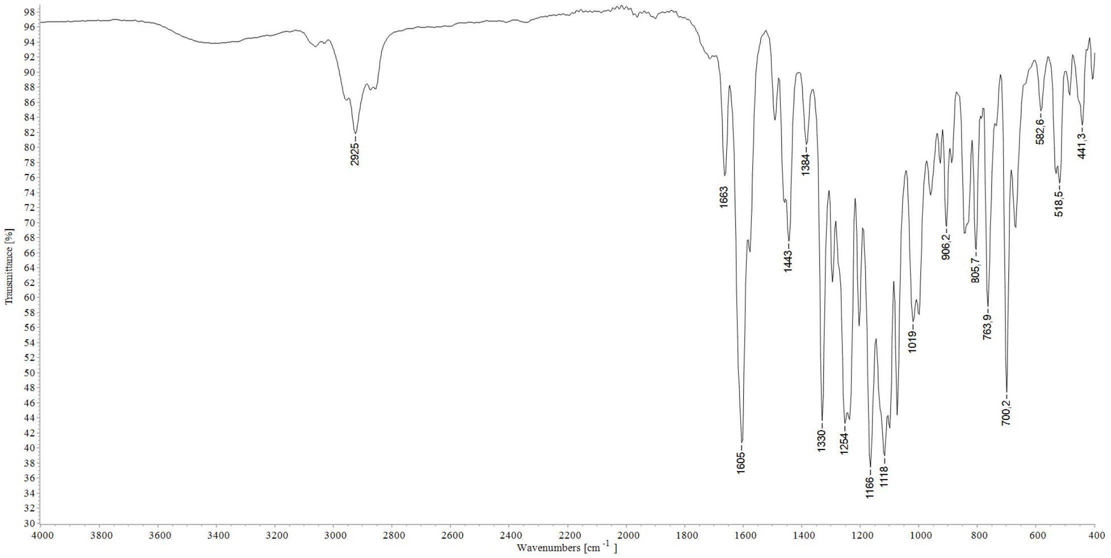

Figure S67: Infrared spectrum υmax of compound **19**.

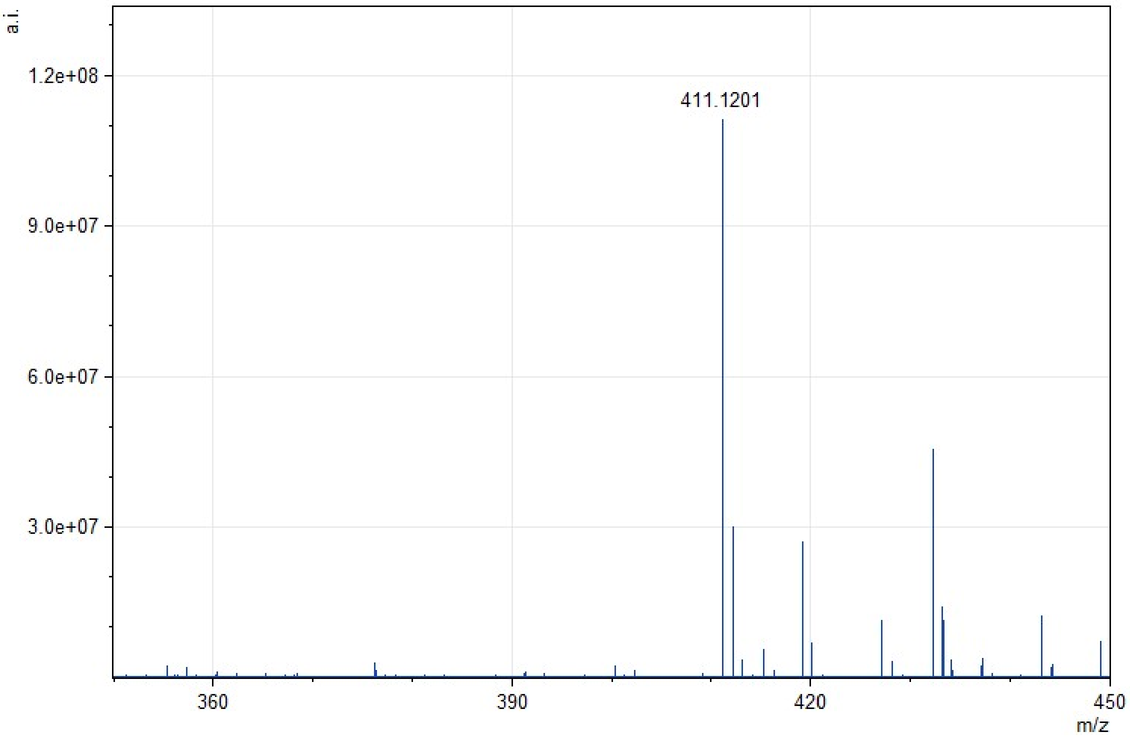

Figure S68: HRMS spectrum of compound **19**.

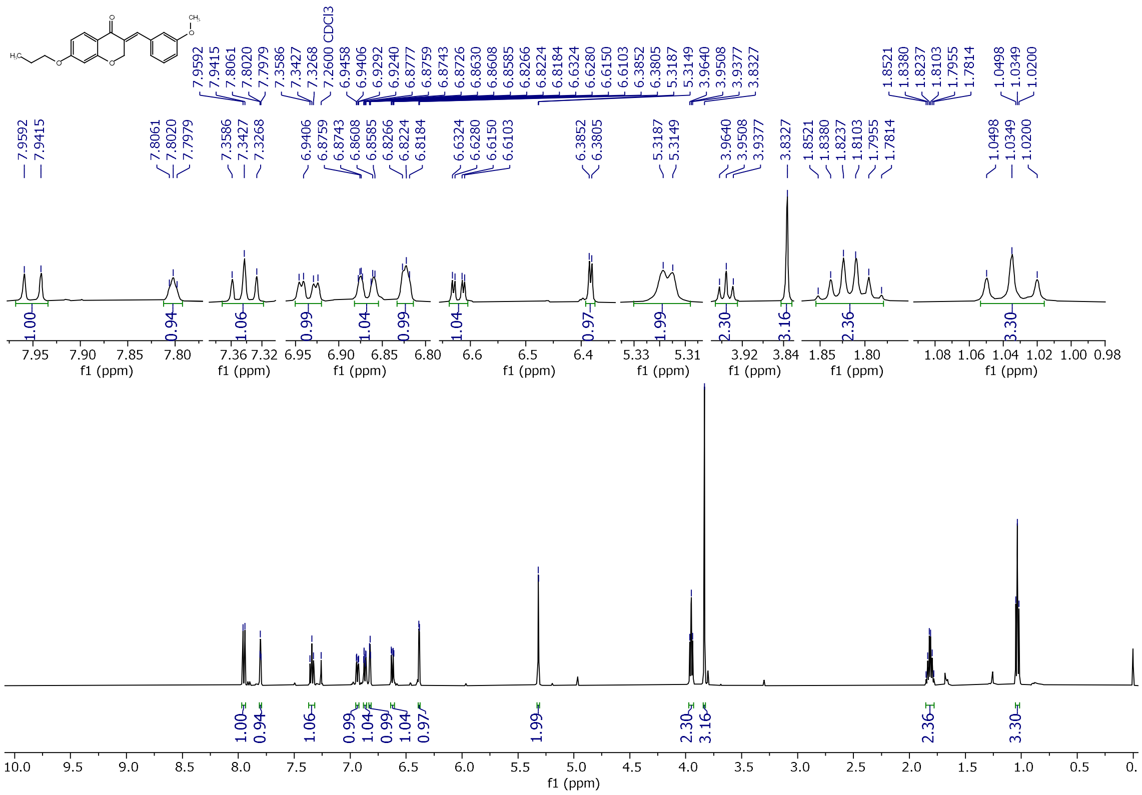

Figure S69: ^{1}H NMR spectrum (500 MHz, $CDCl_3$) of compound **20**.

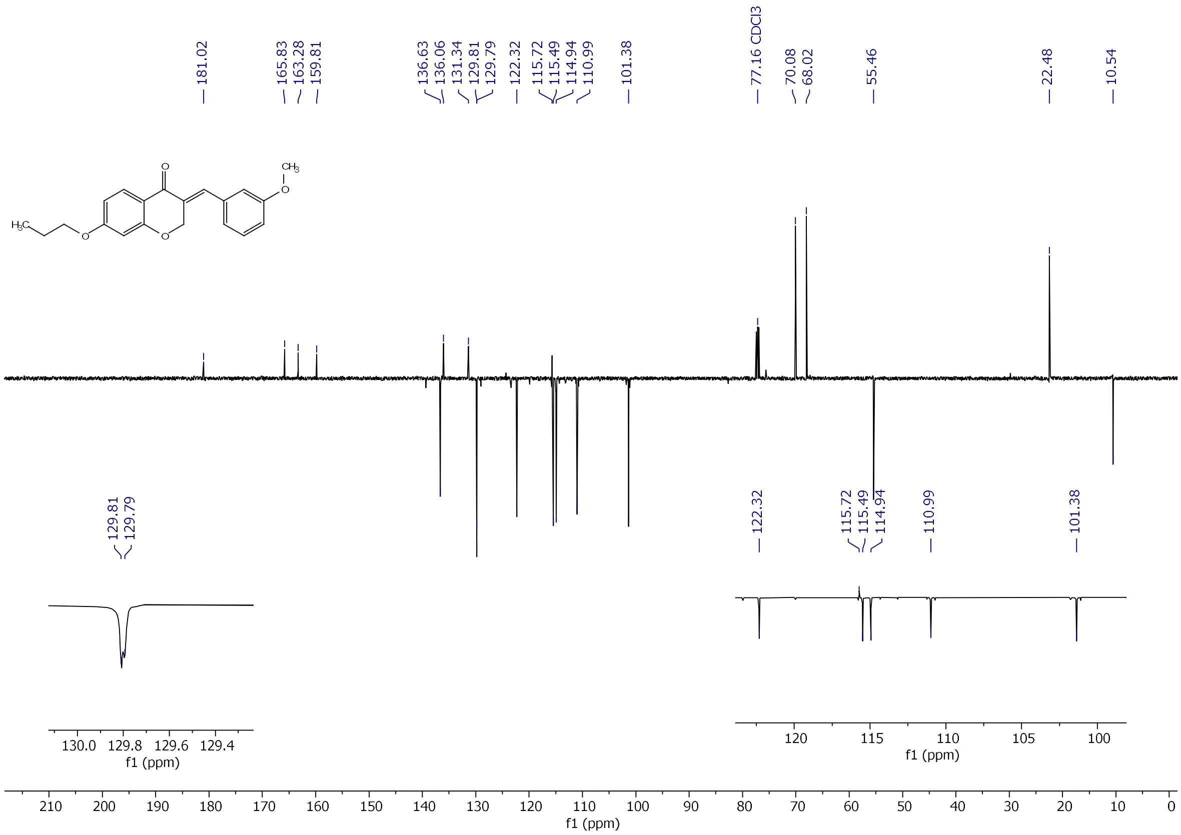

Figure S70: ^{13}C NMR-APT spectrum (125 MHz, $CDCl_3$) of compound **20**.

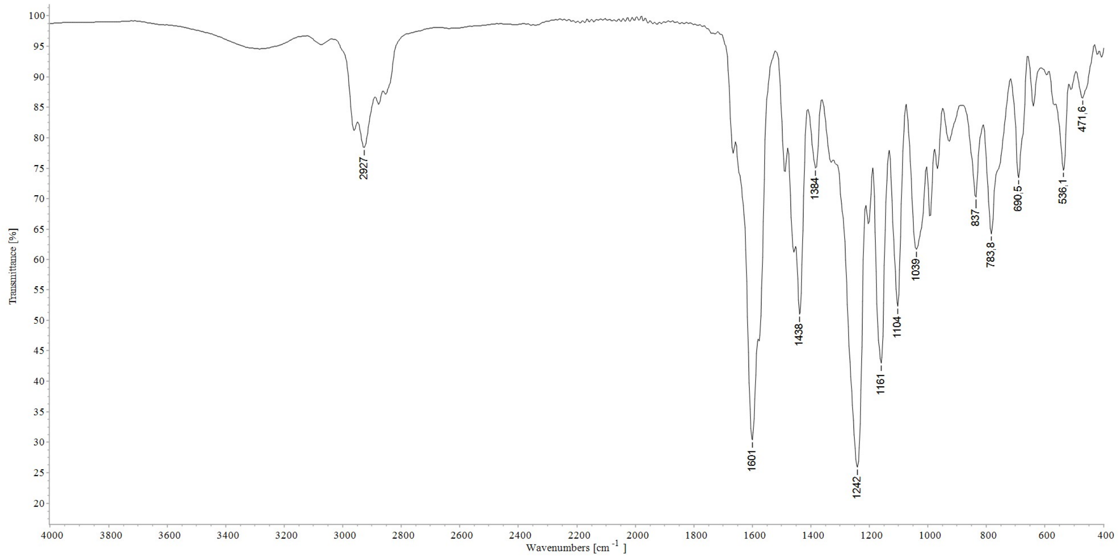

Figure S71: Infrared spectrum υmax of compound **20**.

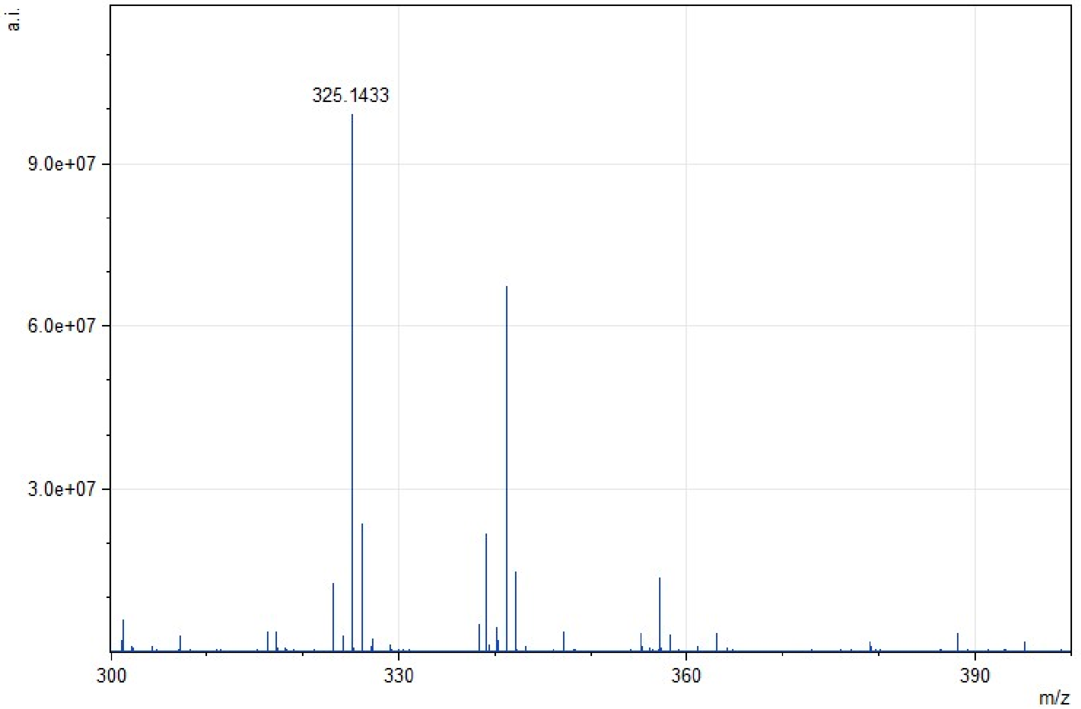

Figure S72: HRMS spectrum of compound **20**.

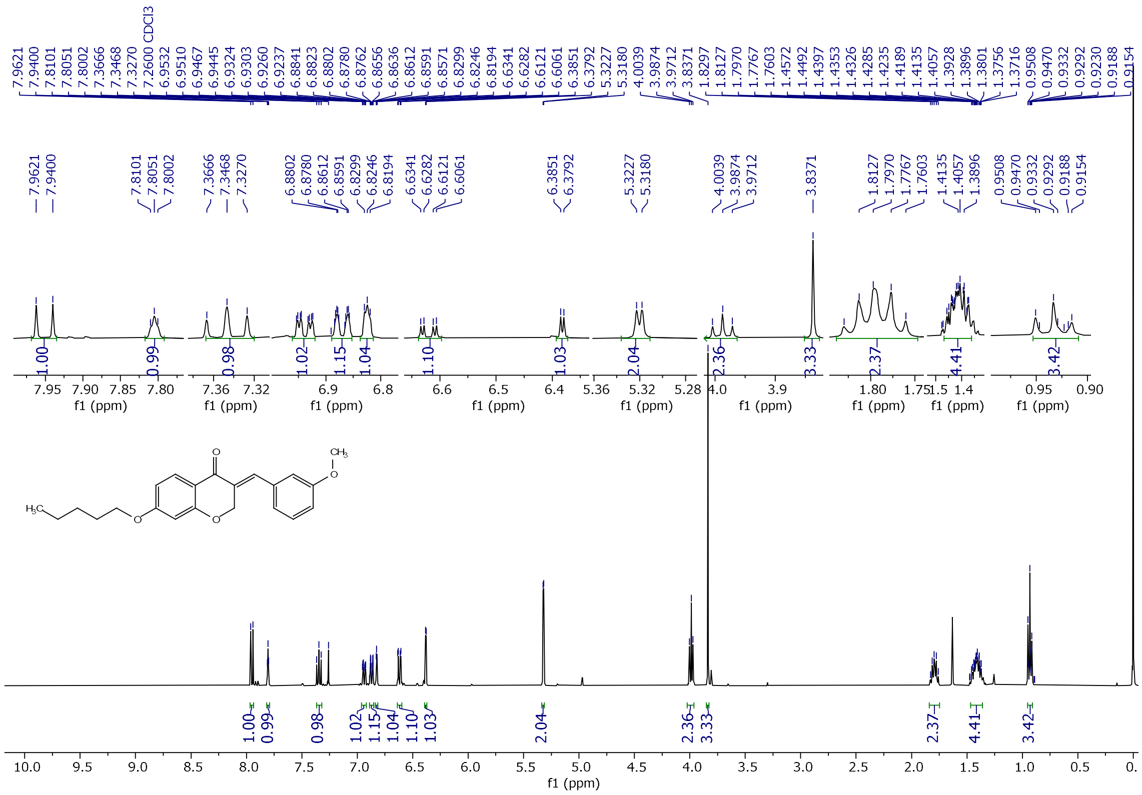

Figure S73: ^{1}H NMR spectrum (400 MHz, $CDCl_3$) of compound **21**.

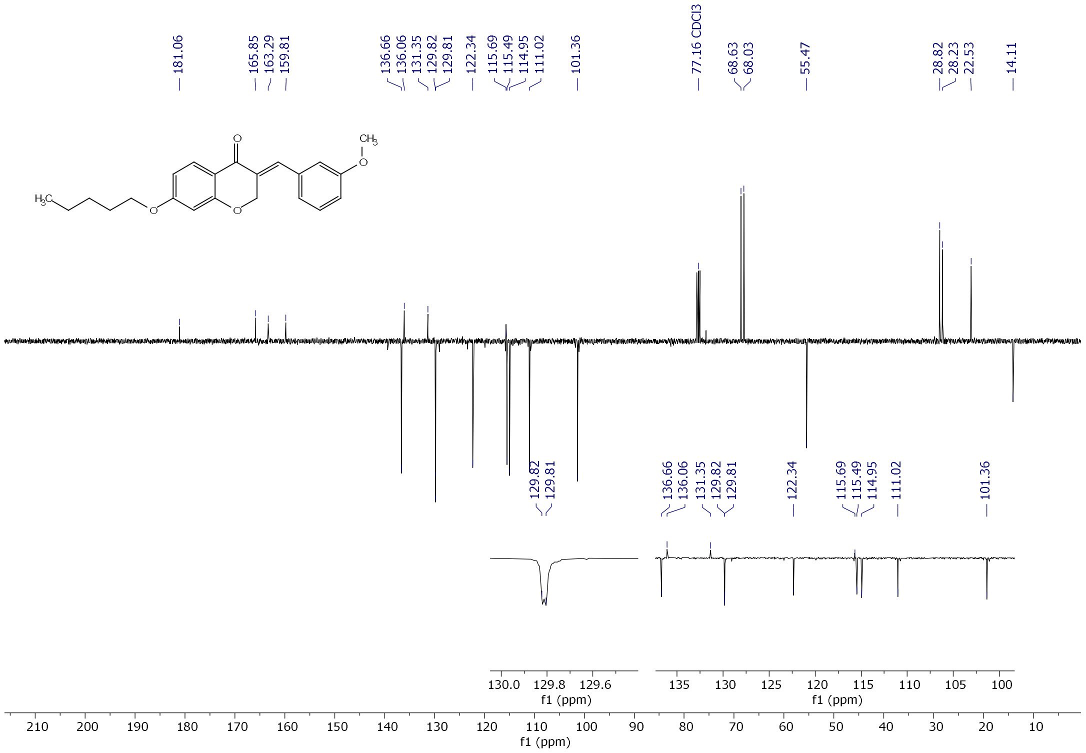

Figure S74: ^{13}C NMR-APT spectrum (100 MHz, $CDCl_3$) of compound **21**.

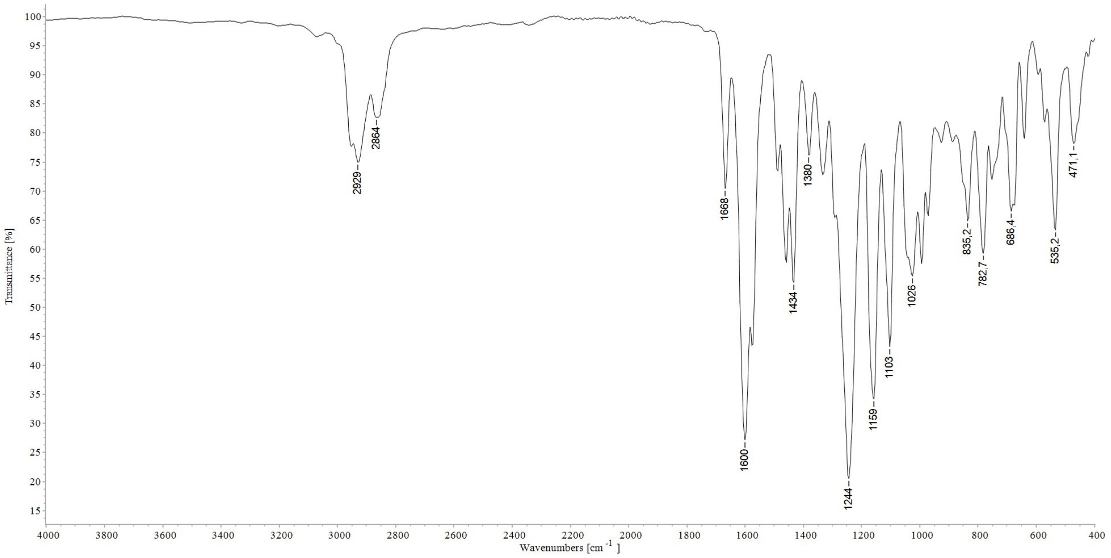

Figure S75: Infrared spectrum υmax of compound **21**.

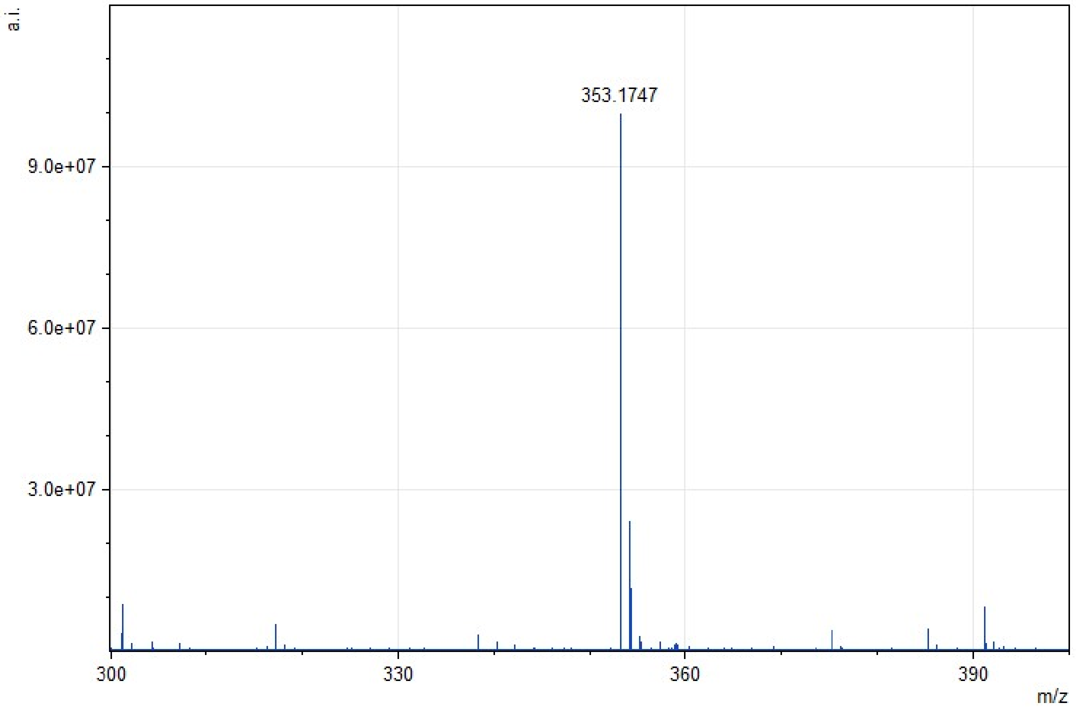

Figure S76: HRMS spectrum of compound **21**.

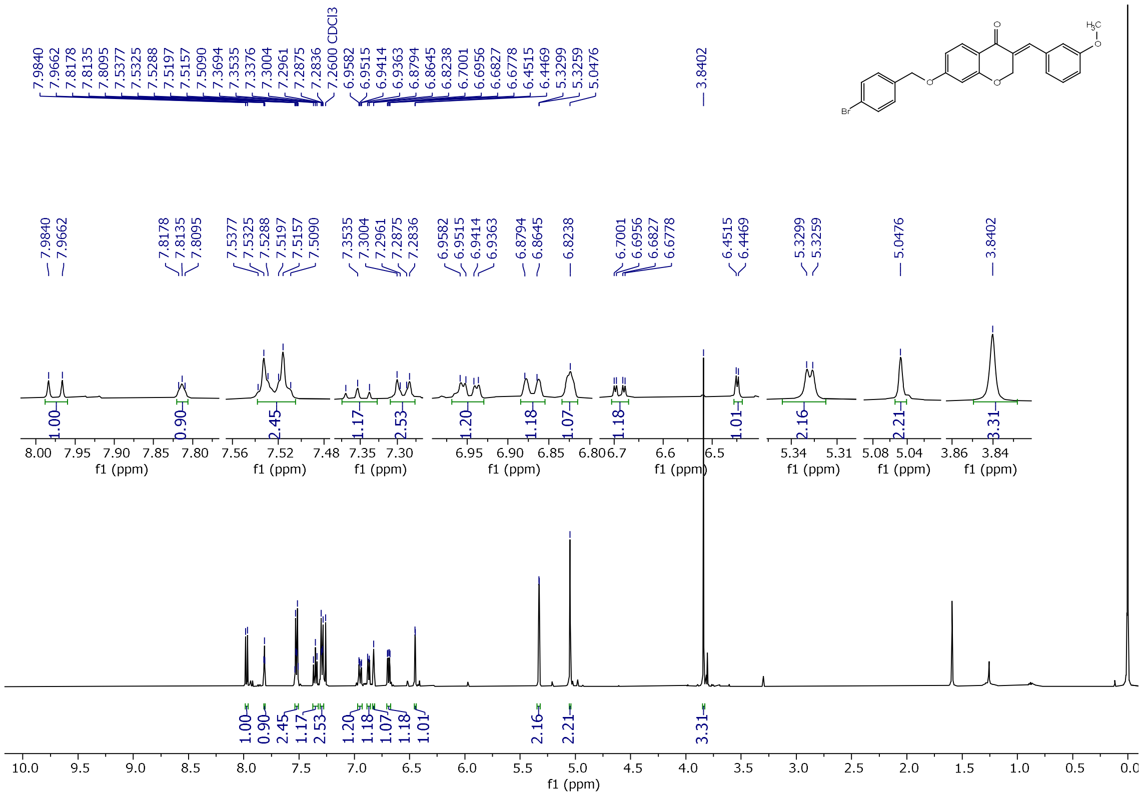

Figure S77: ^{1}H NMR spectrum (500 MHz, $CDCl_3$) of compound **22**.

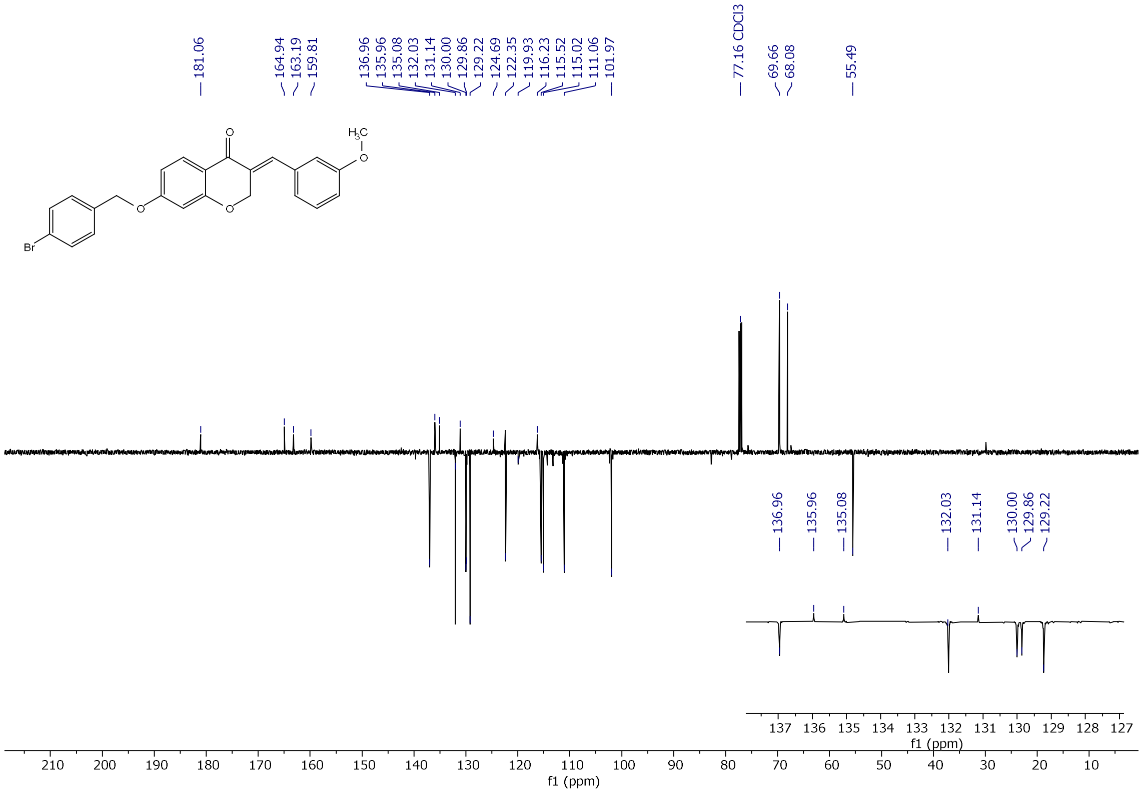

Figure S78: ^{13}C NMR-APT spectrum (125 MHz, $CDCl_3$) of compound **22**.

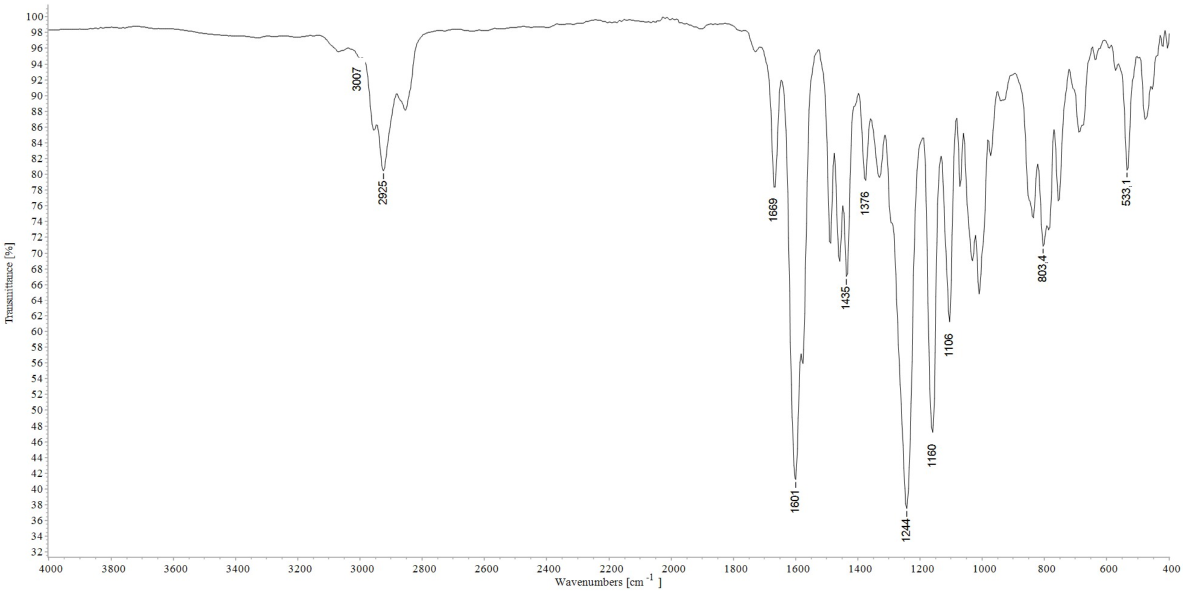

Figure S79: Infrared spectrum υmax of compound **22**.

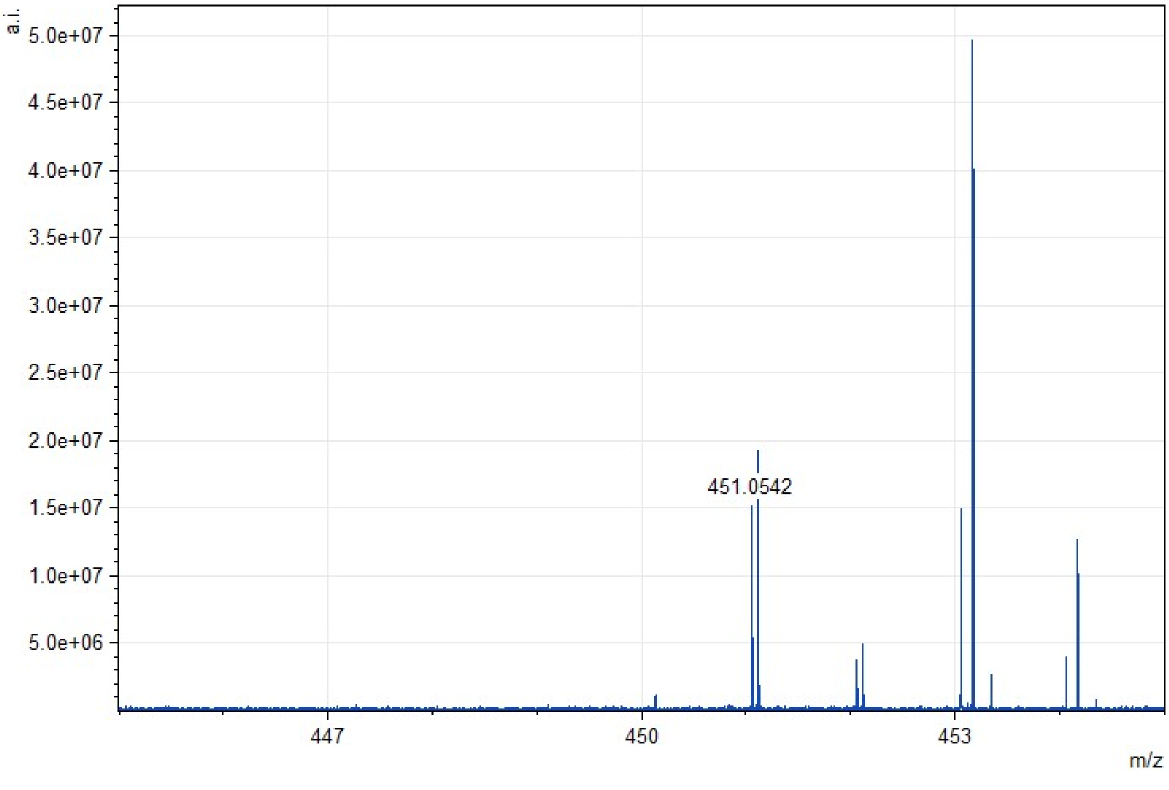

Figure S80: HRMS spectrum of compound **22**.

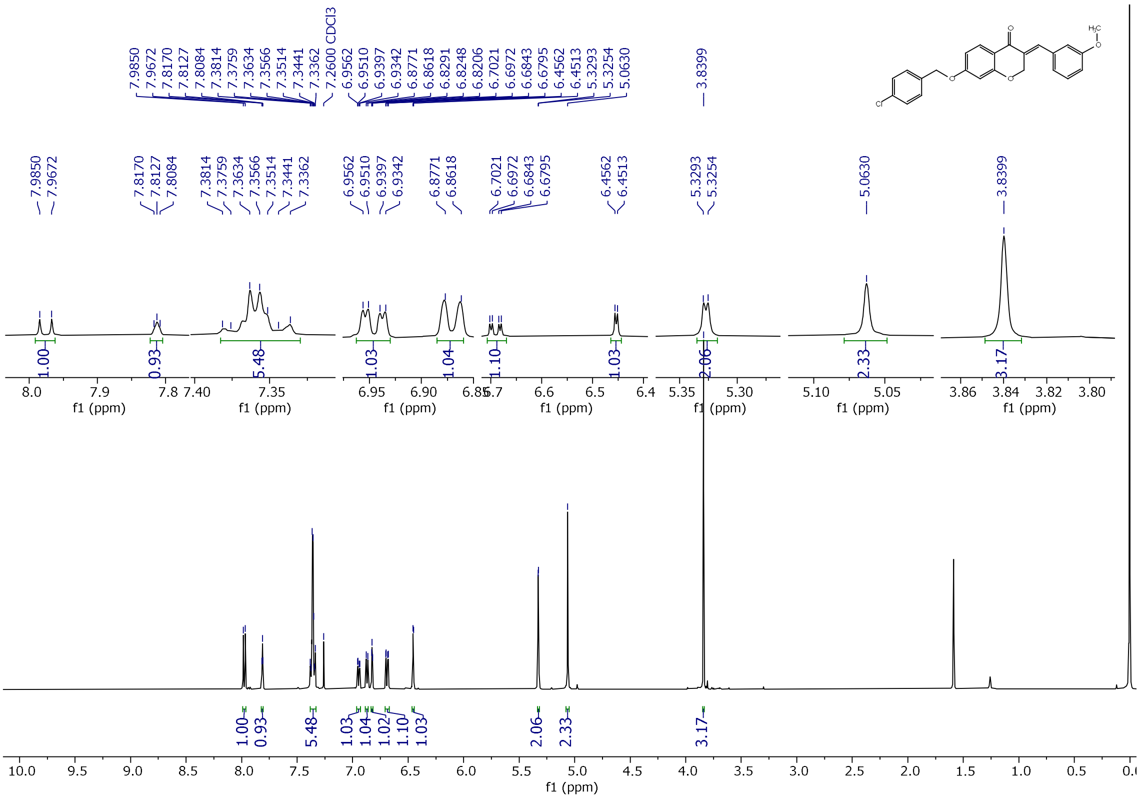

Figure S81: ^{1}H NMR spectrum (500 MHz, $CDCl_3$) of compound **23**.

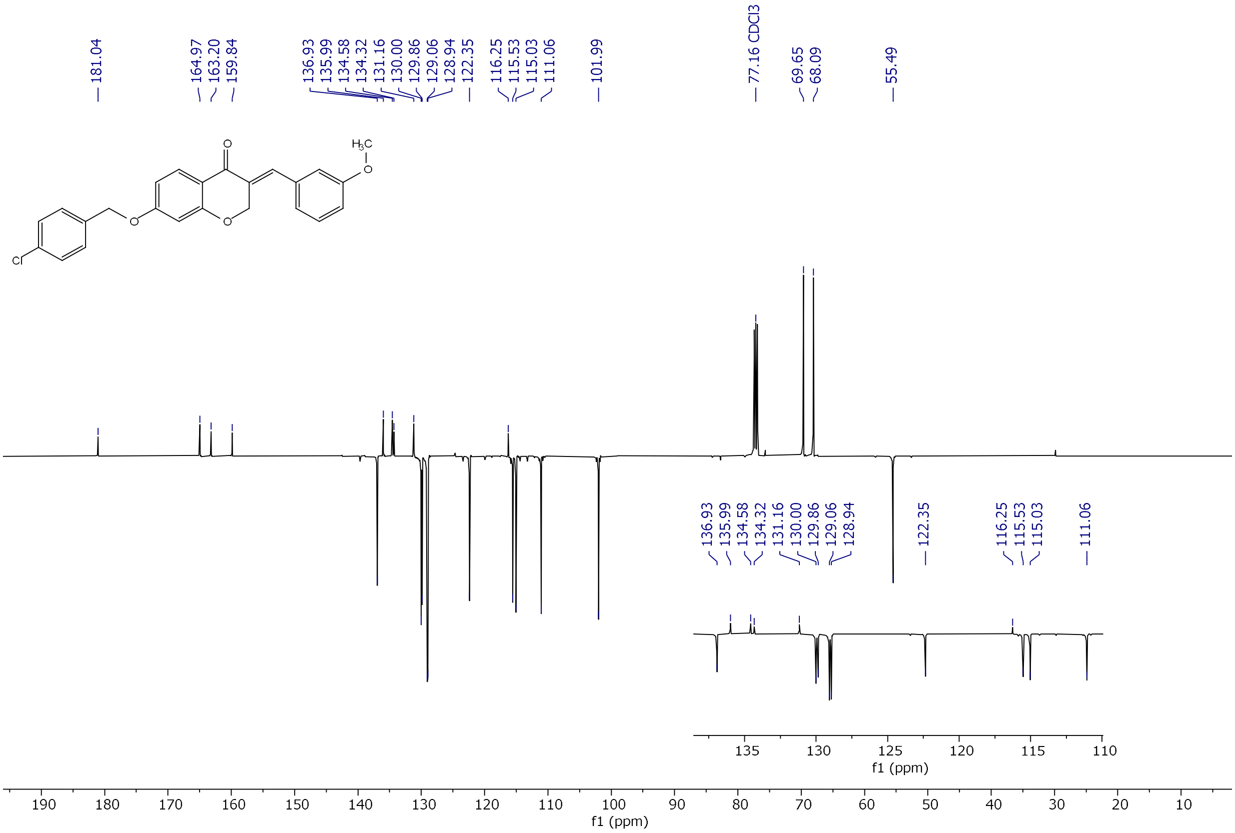

Figure S82: ^{13}C NMR-APT spectrum (125 MHz, $CDCl_3$) of compound **23**.

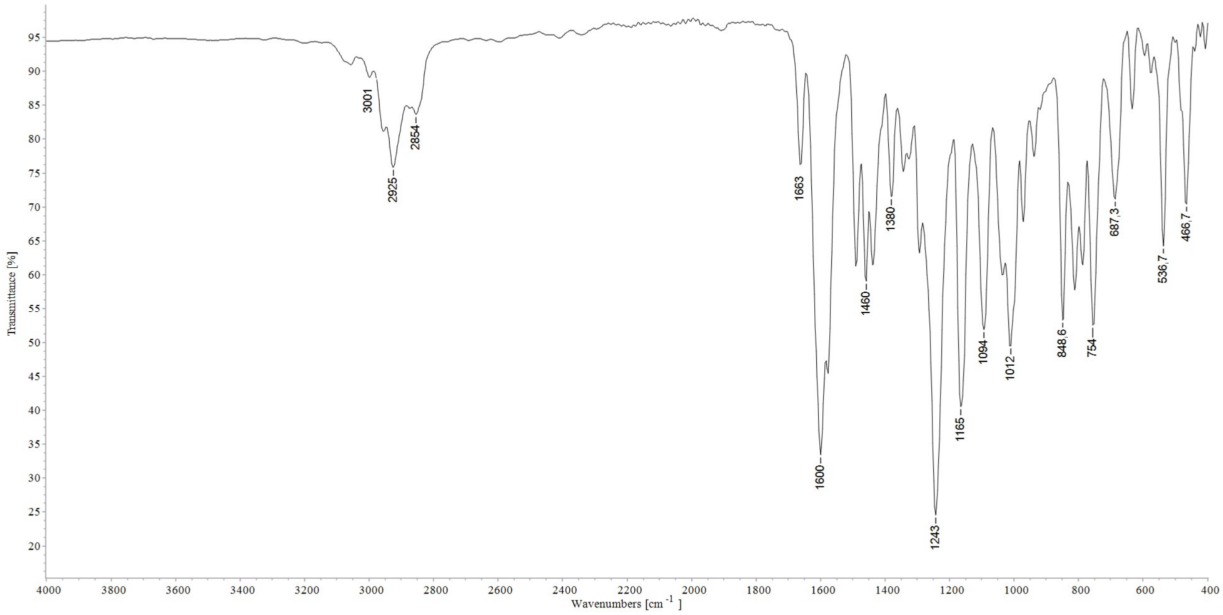

Figure S83: Infrared spectrum υmax of compound **23**.

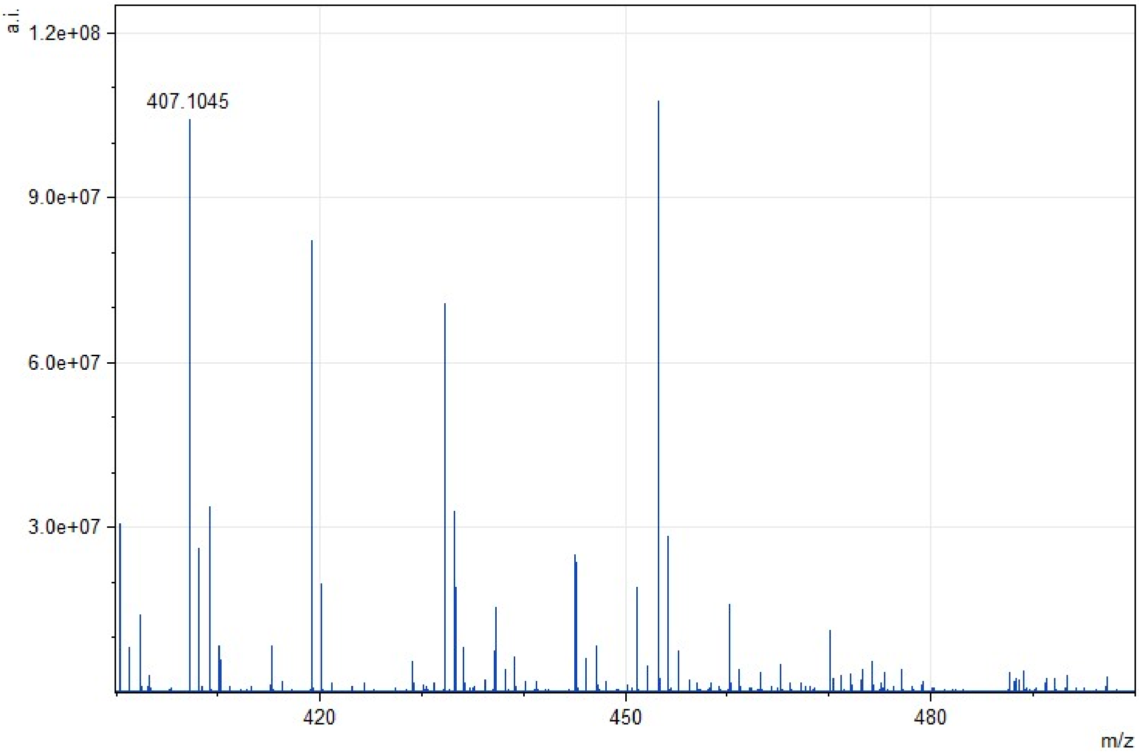

Figure S84: HRMS spectrum of compound **23**.

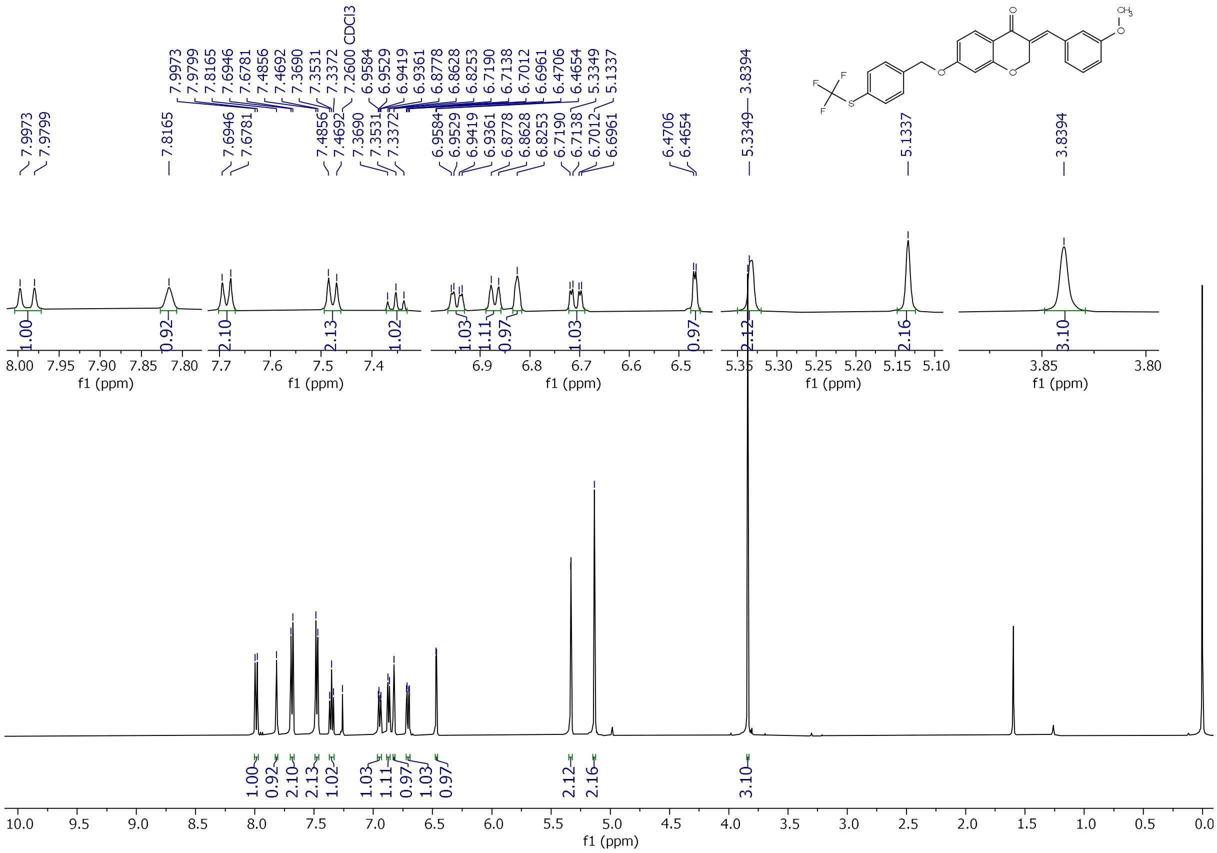

Figure S85: ^{1}H NMR spectrum (500 MHz, $CDCl_3$) of compound **24**.

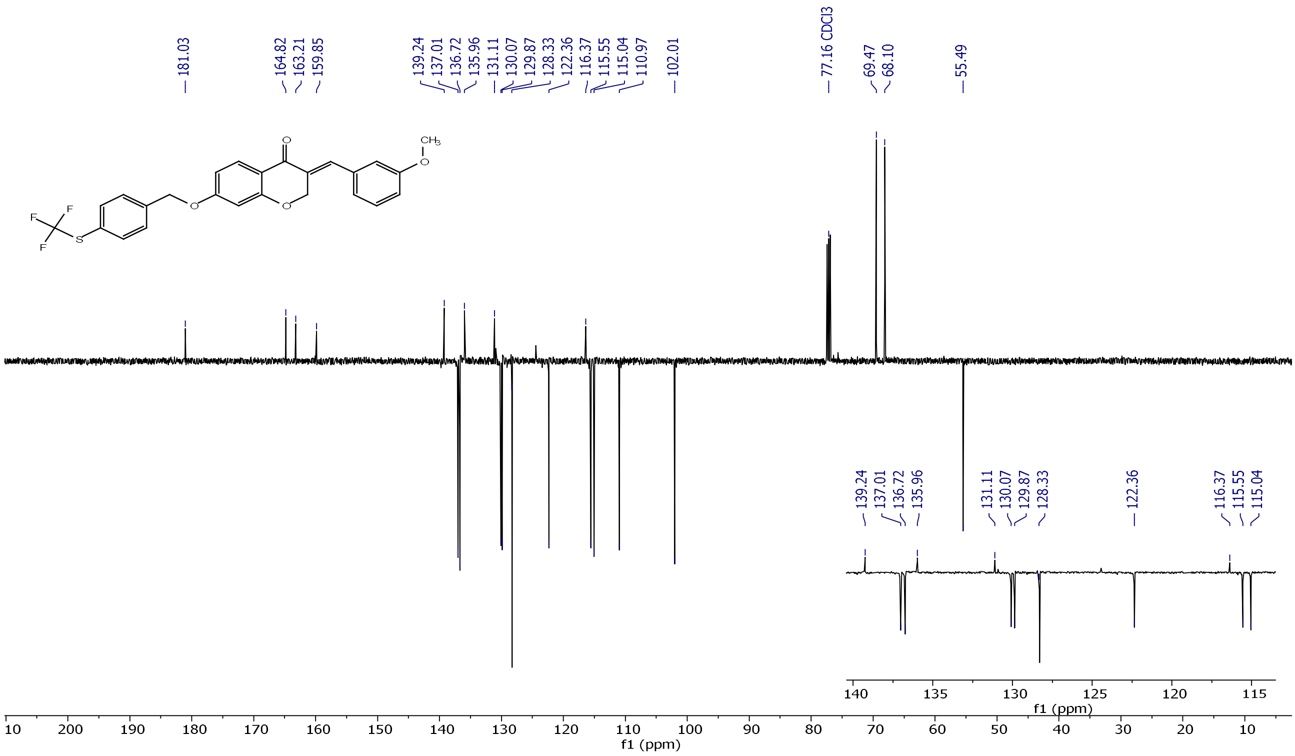

Figure S86: ^{13}C NMR-APT spectrum (125 MHz, $CDCl_3$) of compound **24**.

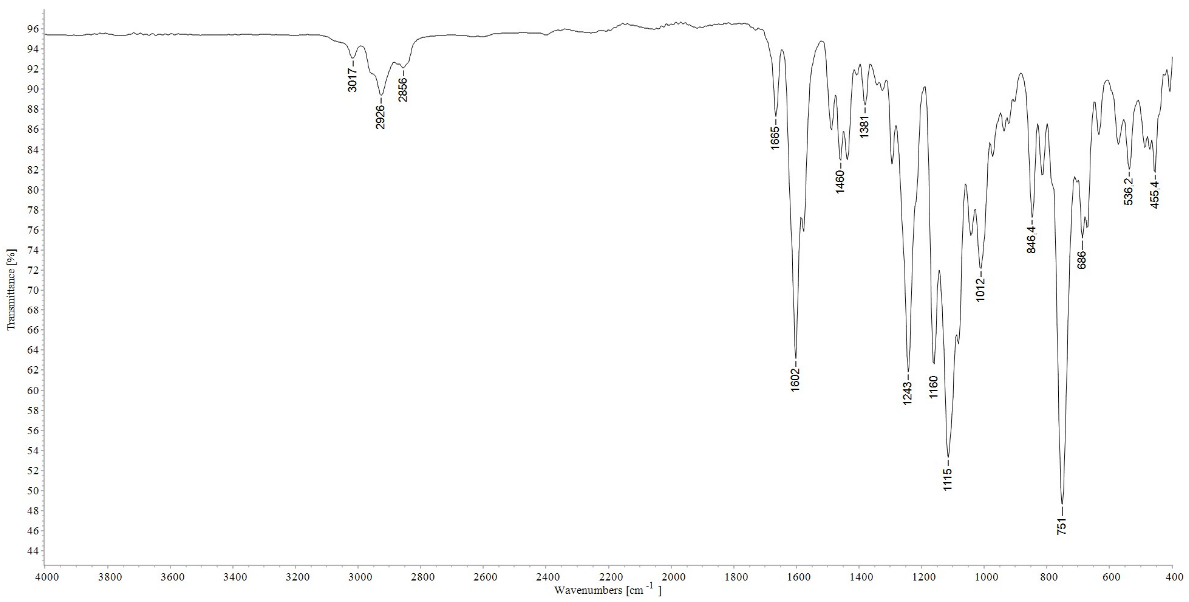

Figure S87: Infrared spectrum υmax of compound **24**.

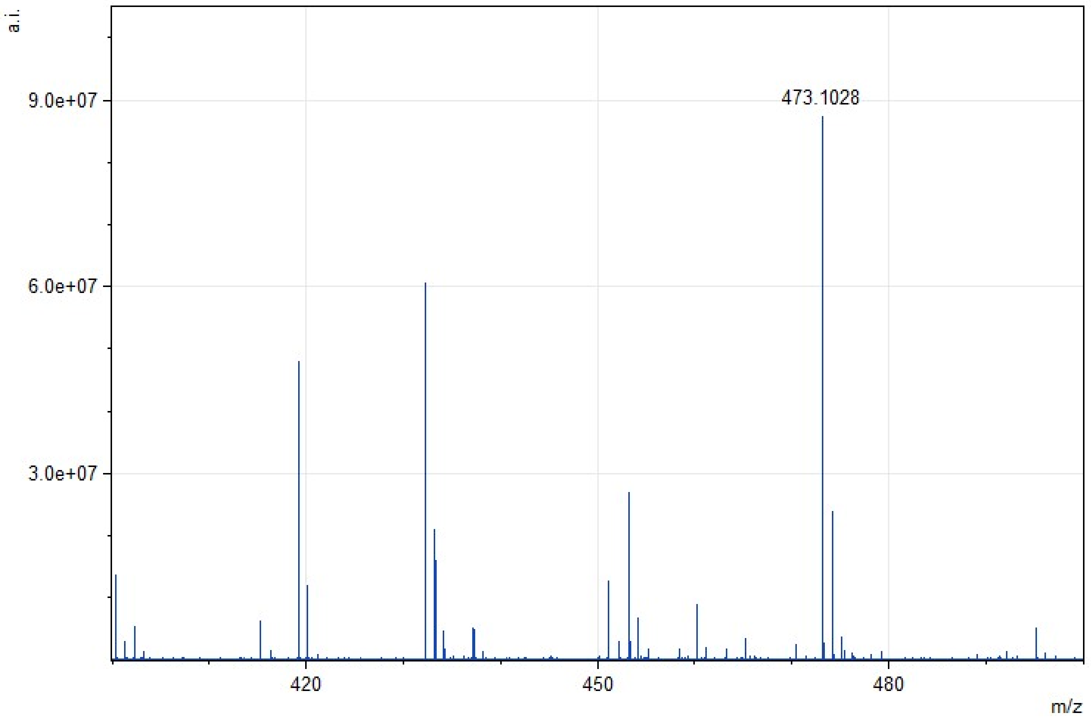

Figure S88: HRMS spectrum of compound **24**.

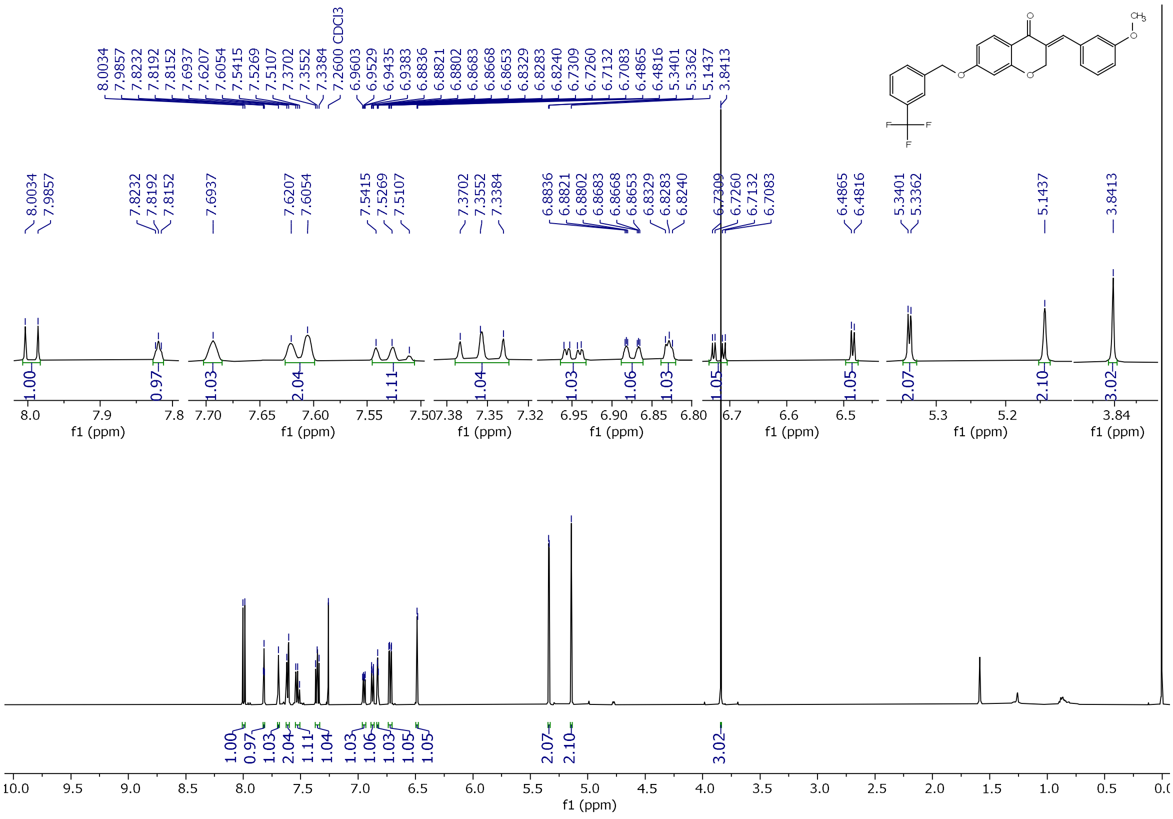

Figure S89: ^{1}H NMR spectrum (500 MHz, $CDCl_3$) of compound **25**.

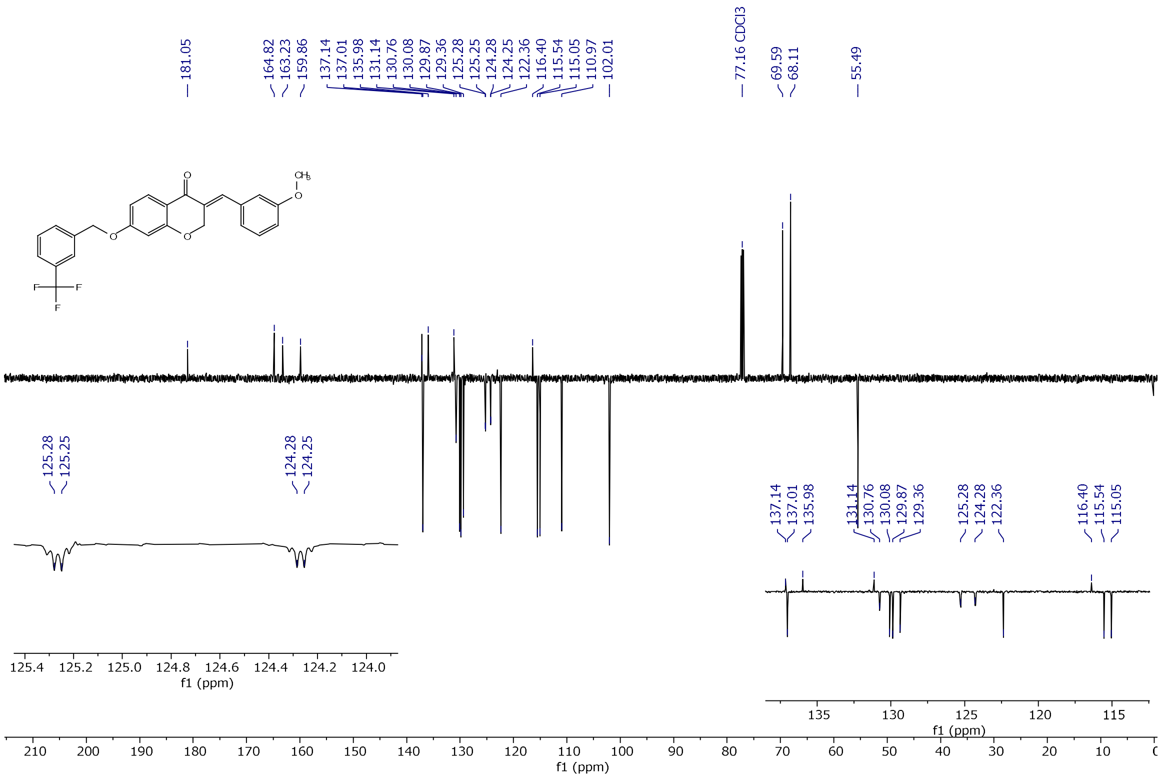

Figure S90: ^{13}C NMR-APT spectrum (125 MHz, $CDCl_3$) of compound **25**.

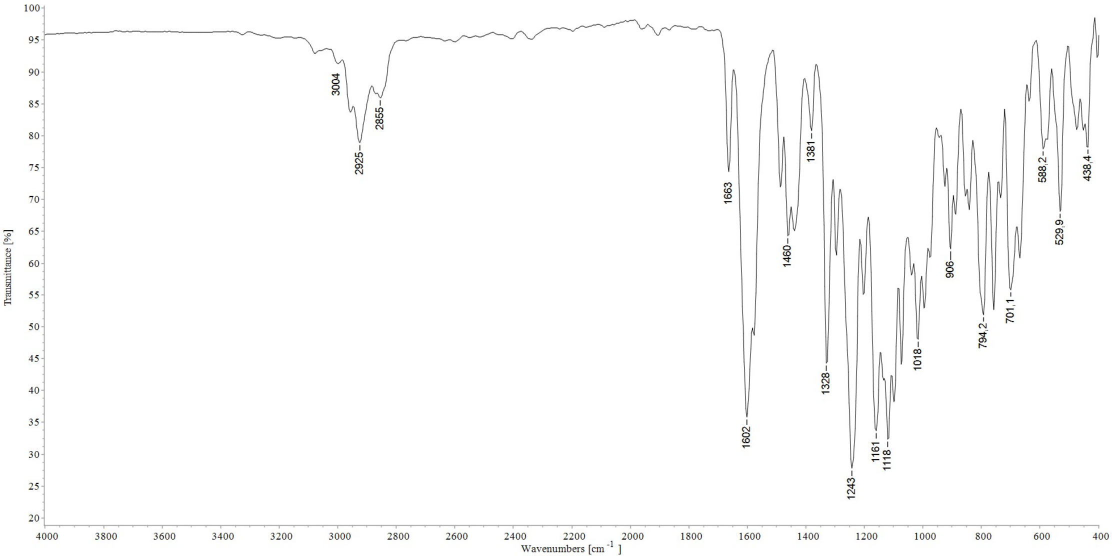

Figure S91: Infrared spectrum υmax of compound **25**.

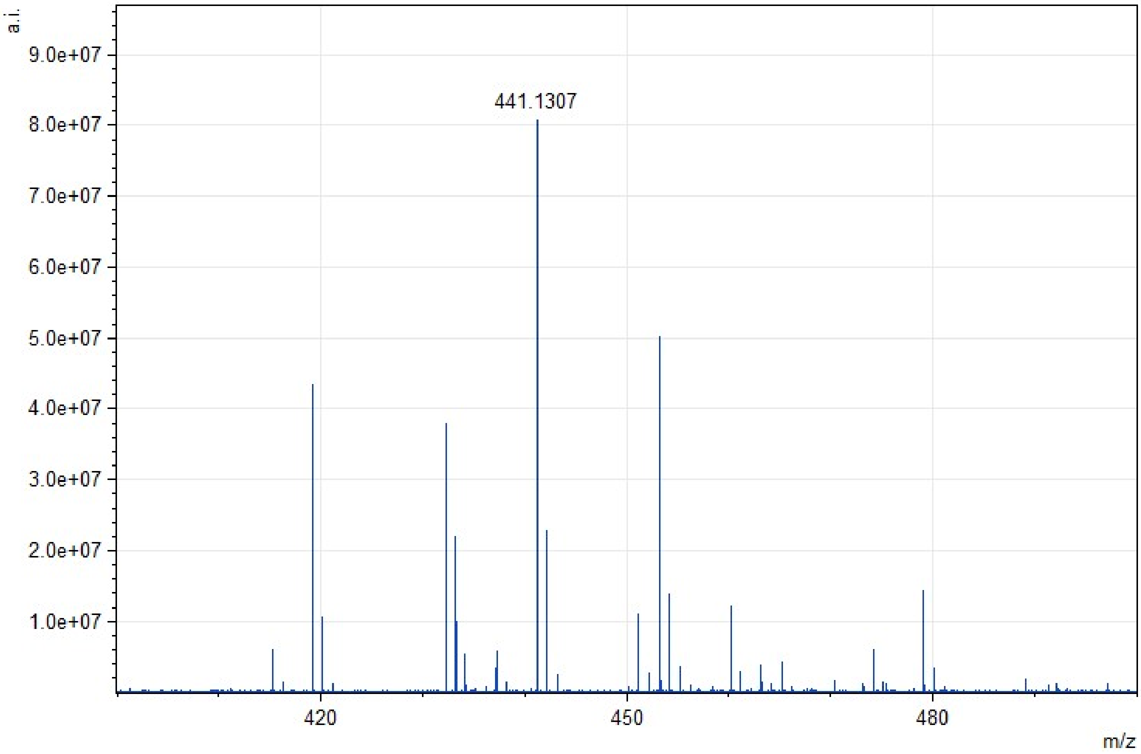

Figure S92: HRMS spectrum of compound **25**.

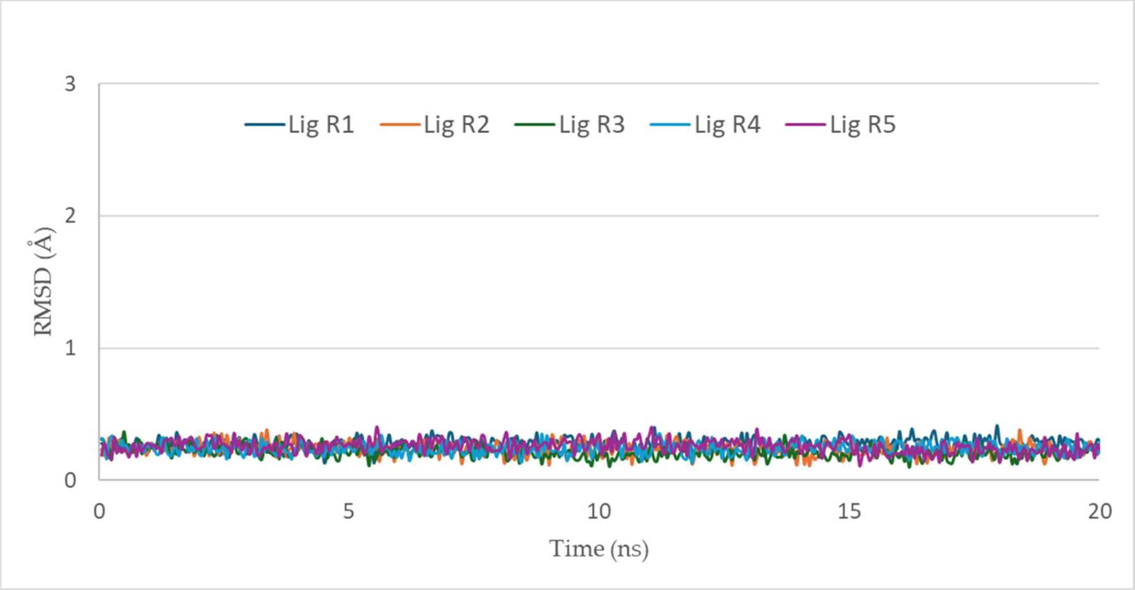

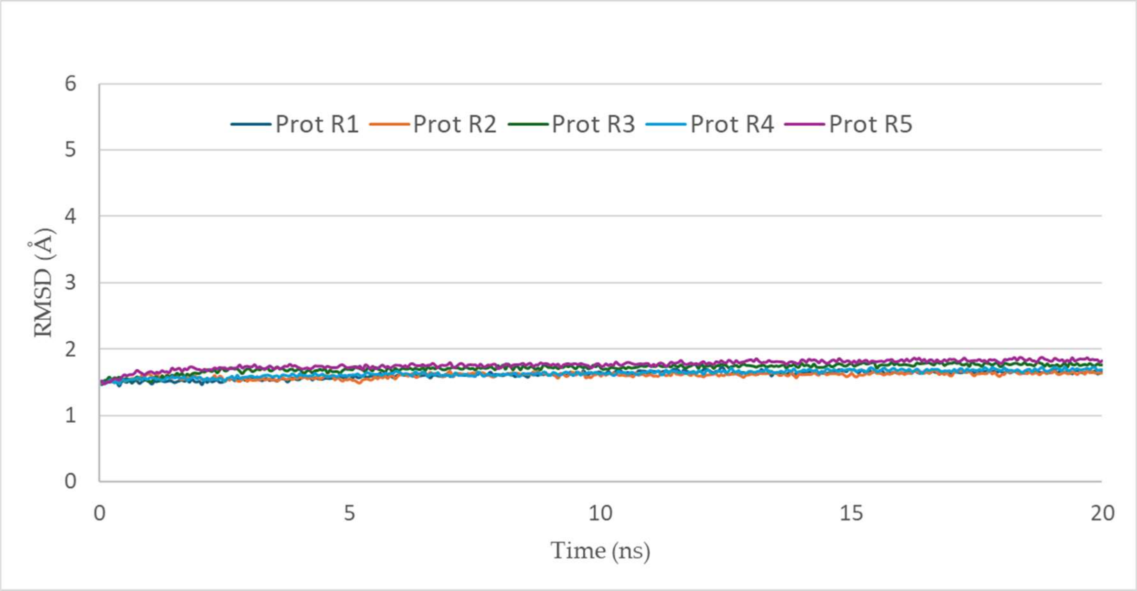

Figure S93: RMSD plots for the predicted complex of compound **1** with CS. Separate plots are provided for the compound (top) and protein (bottom). The five MD replicas are labelled R1 to R5.

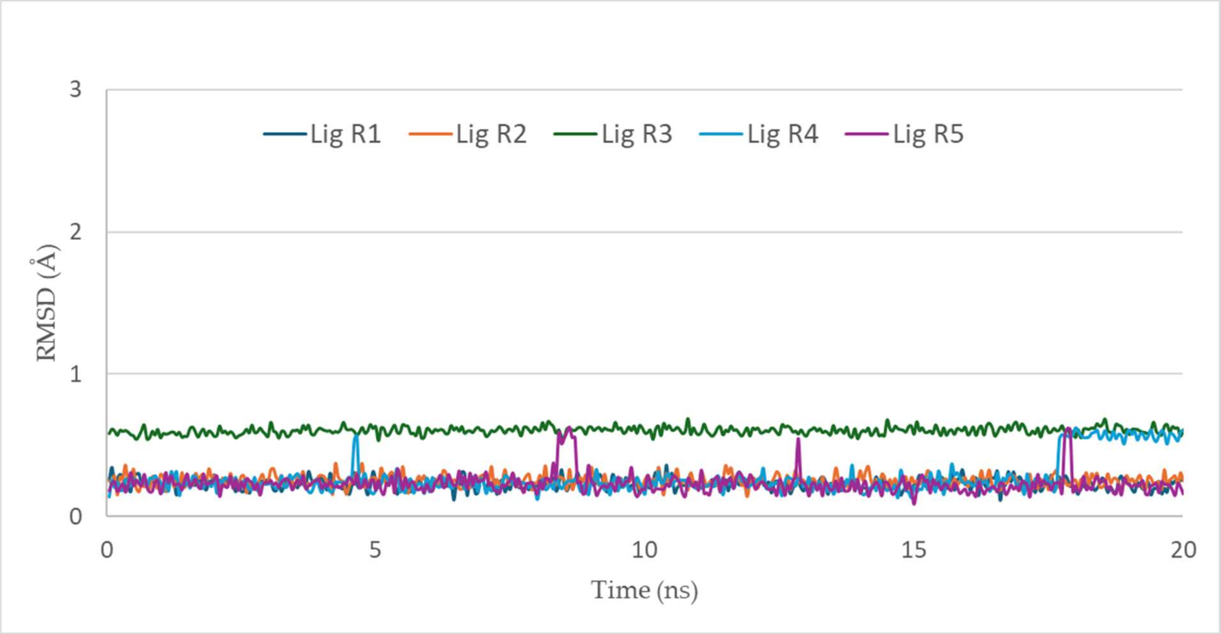

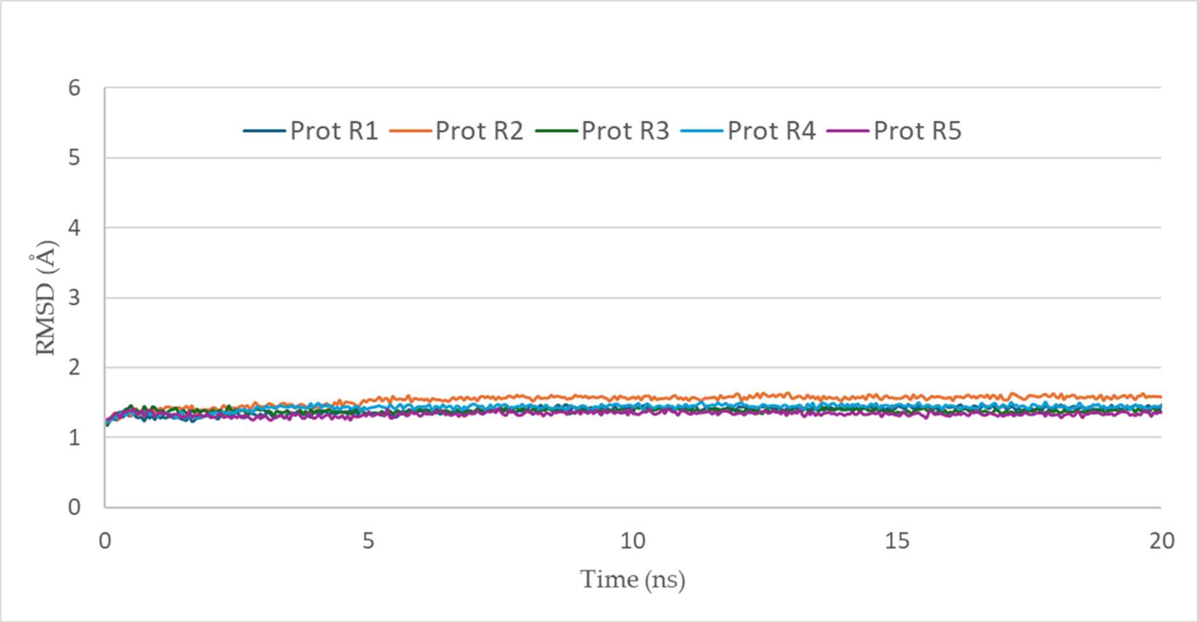

Figure S94: RMSD plots for the predicted complex of compound **2** with HOG1. Separate plots are provided for the compound (top) and protein (bottom). The five MD replicas are labelled R1 to R5.

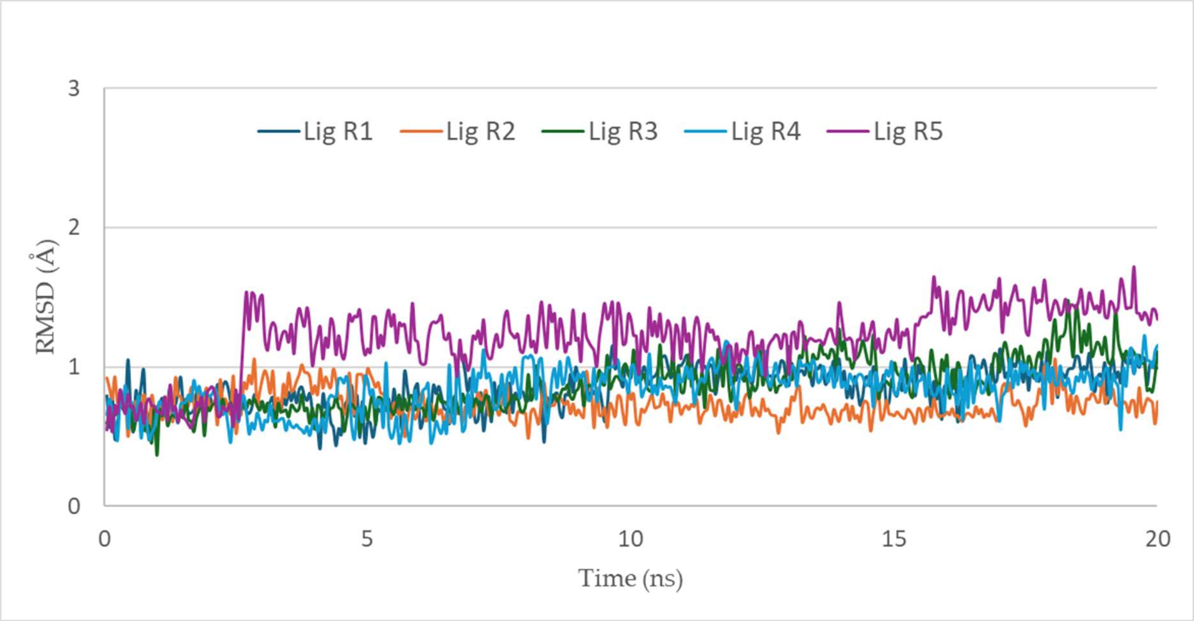

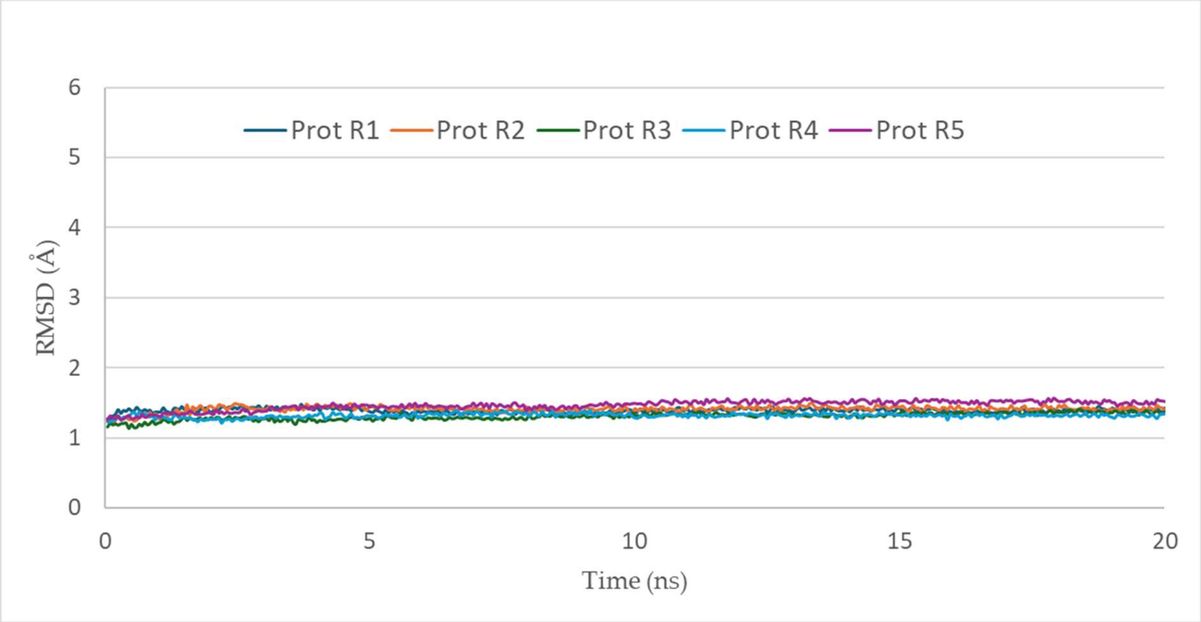

Figure S95: RMSD plots for the predicted complex of compound **21** with HOG1. Separate plots are provided for the compound (top) and protein (bottom). The five MD replicas are labelled R1 to R5.

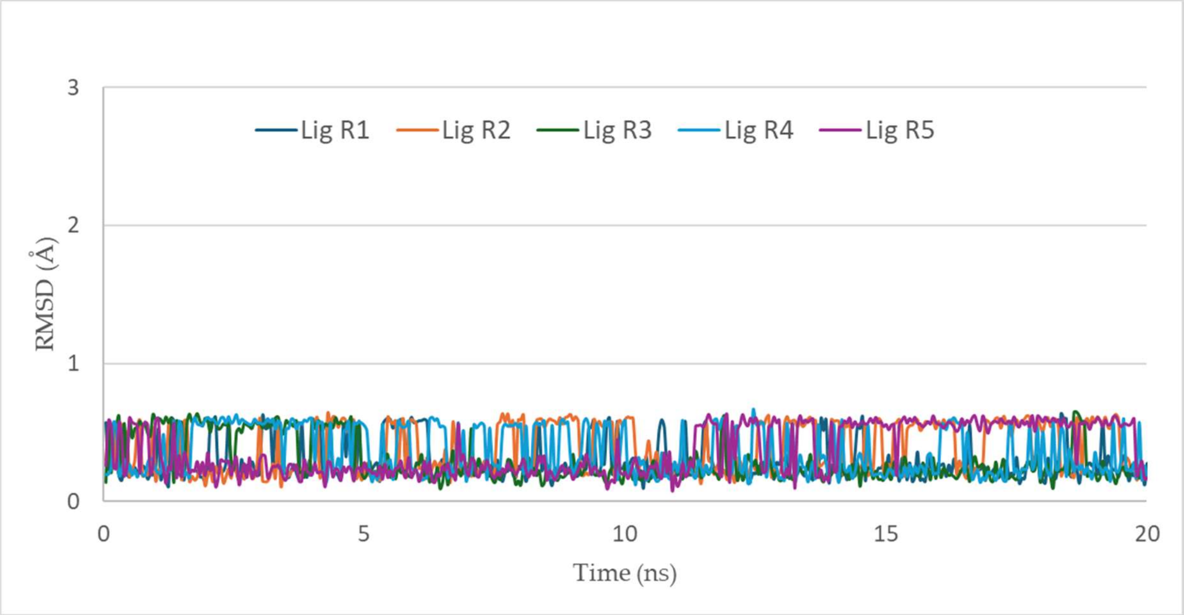

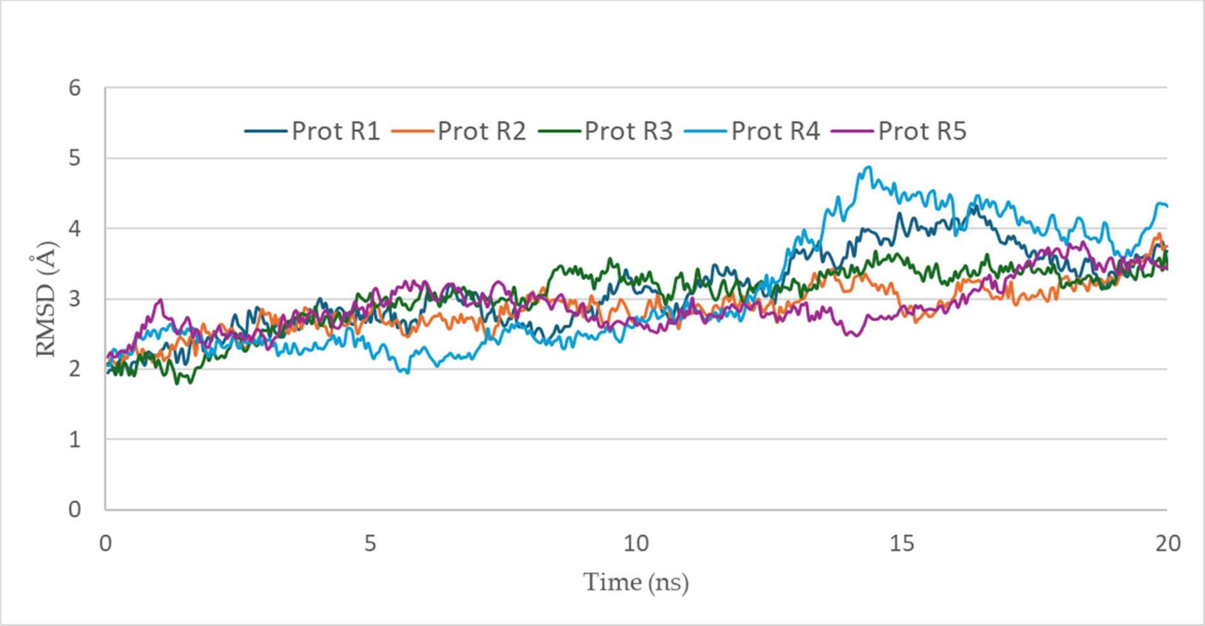

Figure S96: RMSD plots for the predicted complex of compound **2** with FBA1. Separate plots are provided for the compound (top) and protein (bottom). The five MD replicas are labelled R1 to R5.

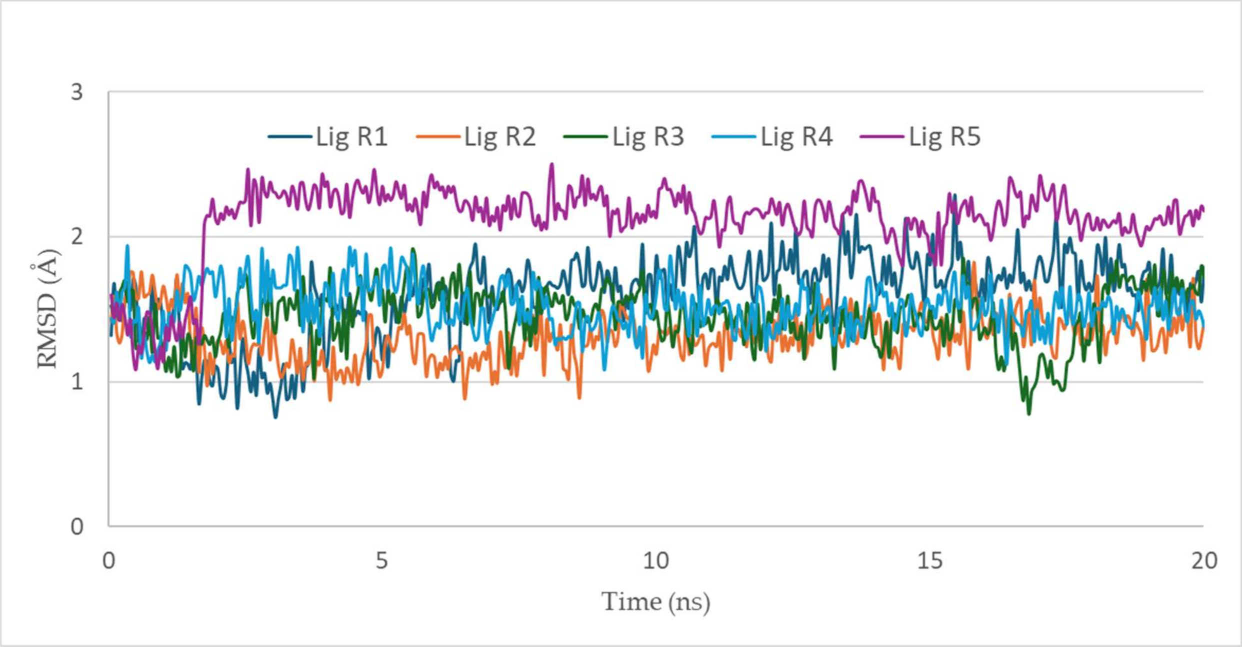

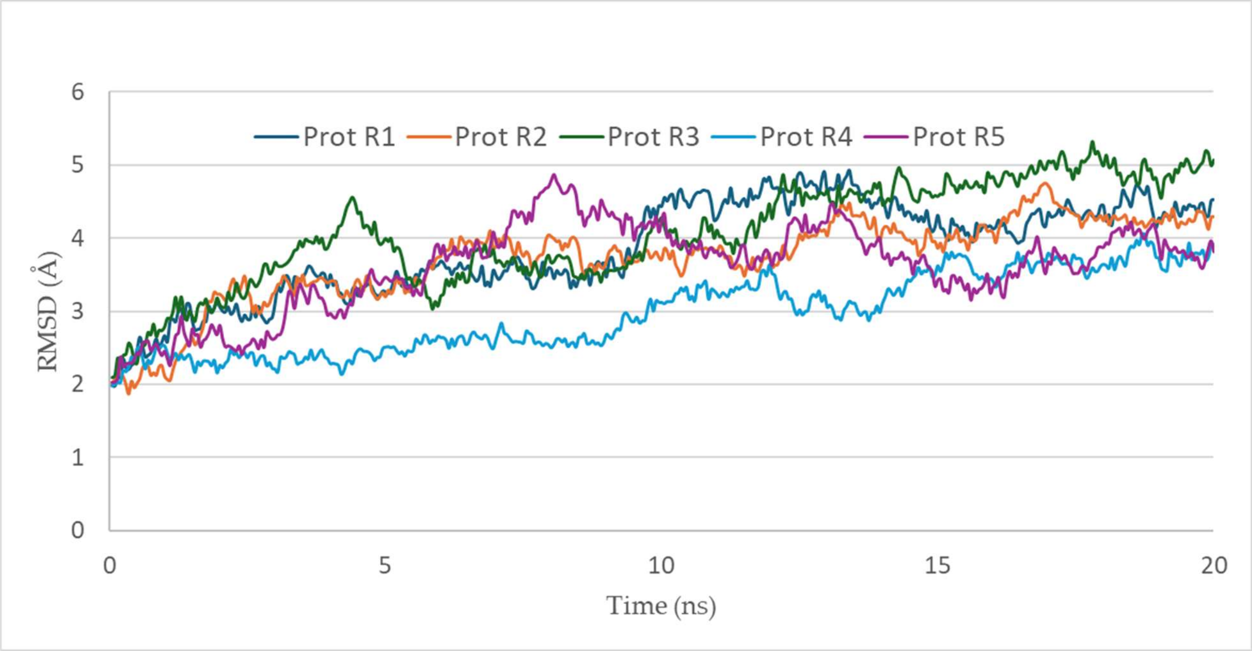

Figure S97: RMSD plots for the predicted complex of compound **21** with FBA1. Separate plots are provided for the compound (top) and protein (bottom). The five MD replicas are labelled R1 to R5.

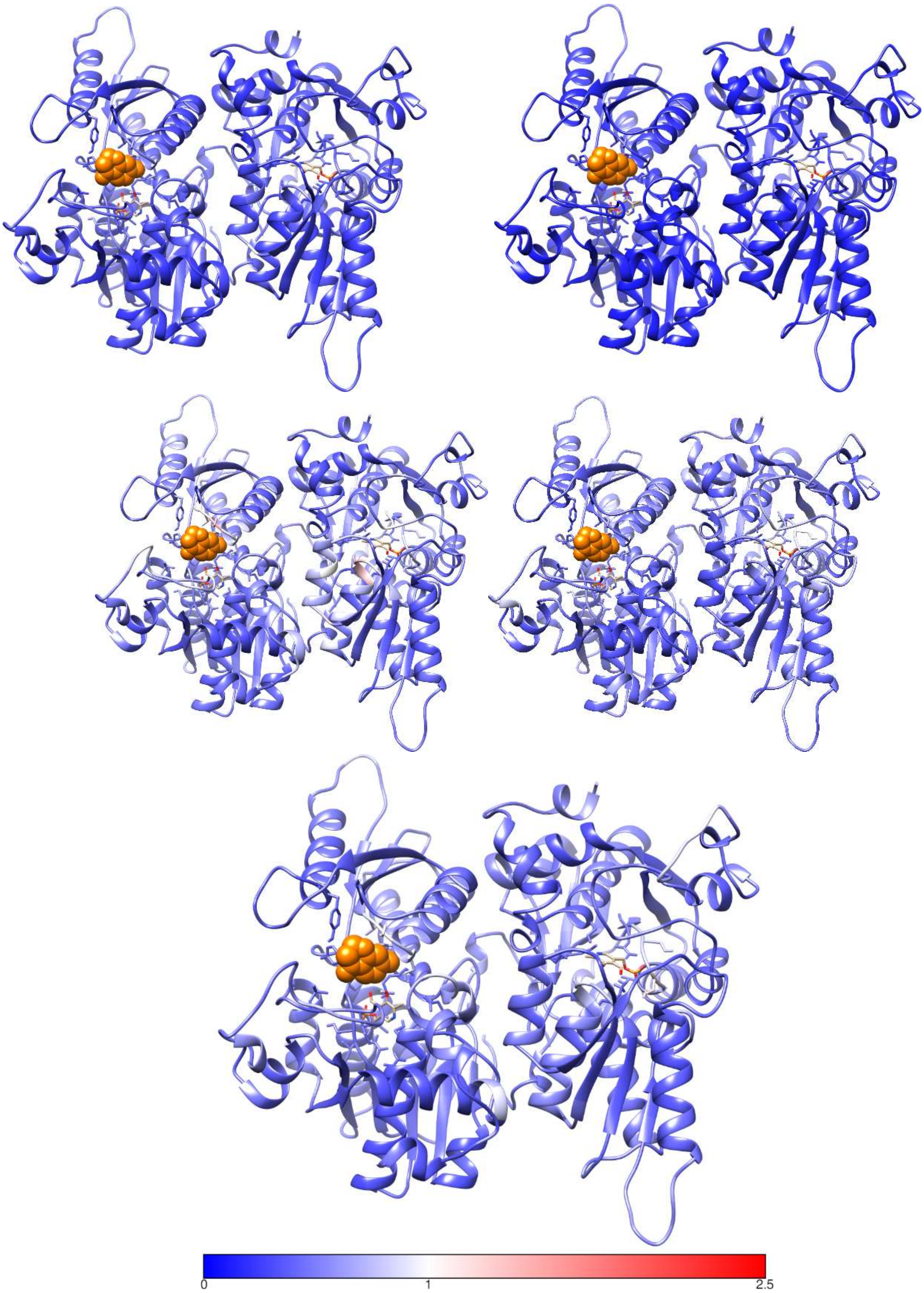

Figure S98: RMSFs of the protein in the complex of compound **1** with CS. One figure is provided per MD replica. The compound is represented as orange spheres.

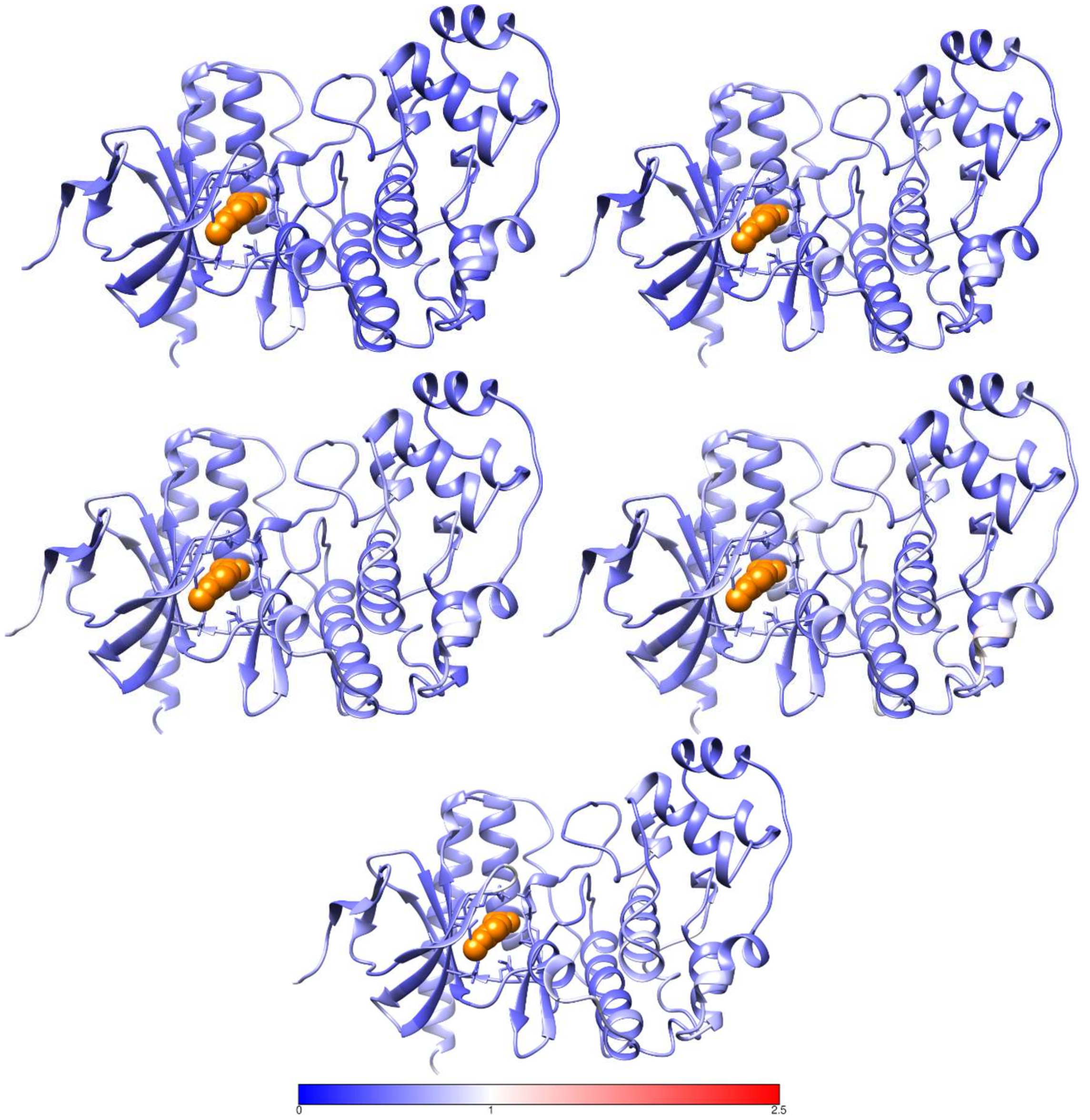

Figure S99: RMSFs of the protein in the complex of compound **2** with HOG1. One figure is provided per MD replica. The compound is represented as orange spheres.

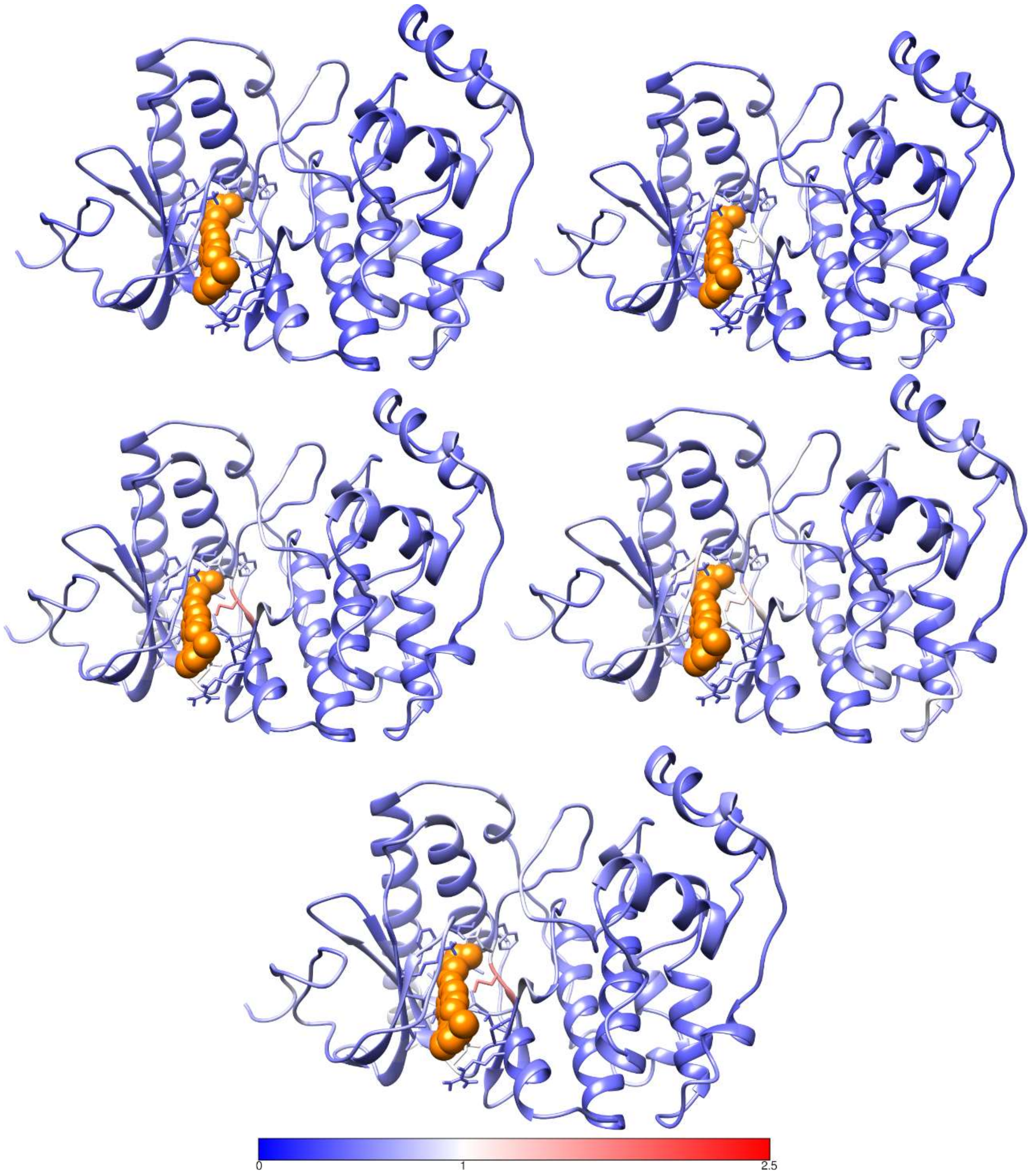

Figure S100: RMSFs of the protein in the complex of compound **21** with HOG1. One figure is provided per MD replica. The compound is represented as orange spheres.

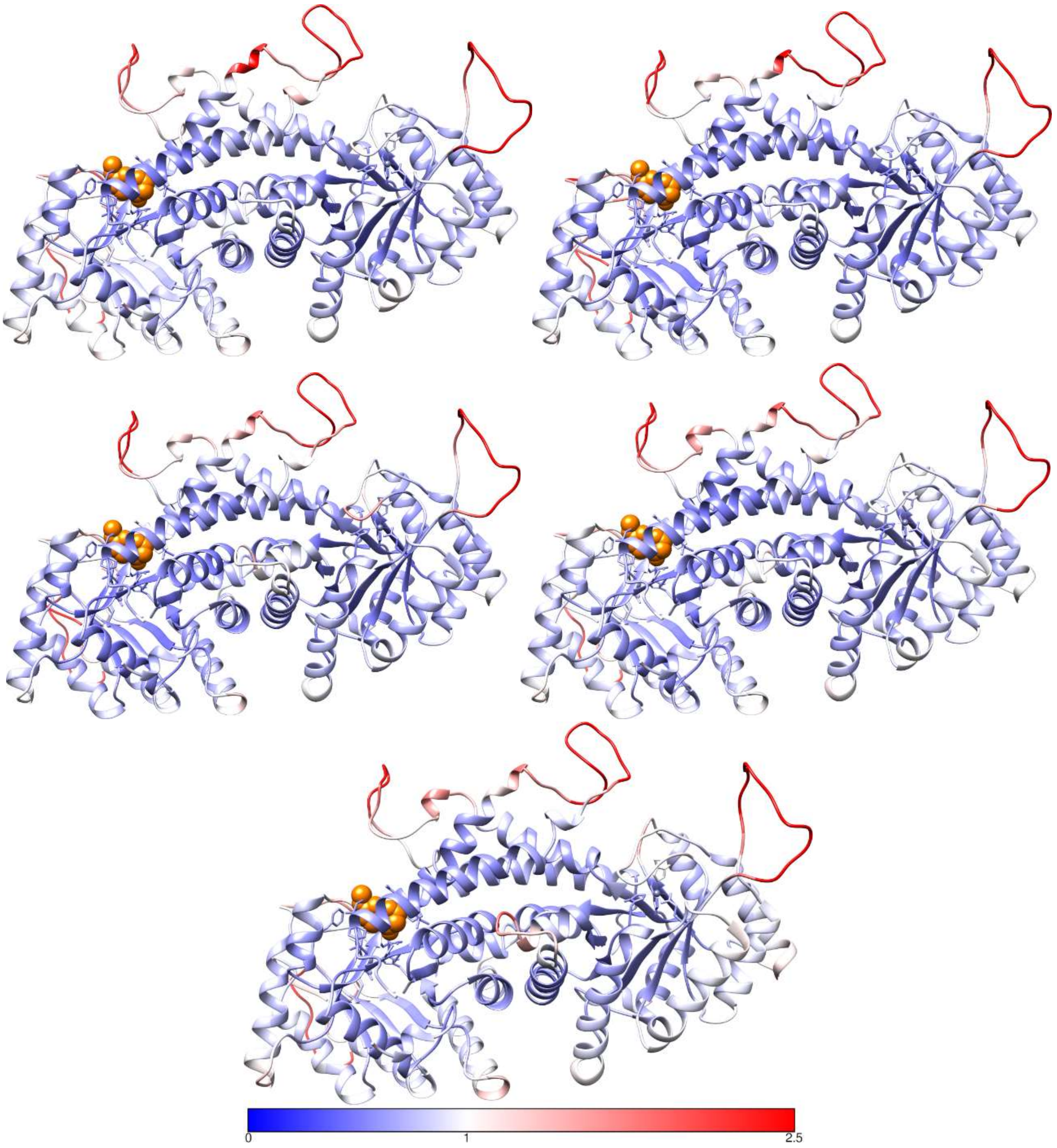

Figure S101: RMSFs of the protein in the complex of compound **2** with FBA1. One figure is provided per MD replica. The compound is represented as orange spheres.

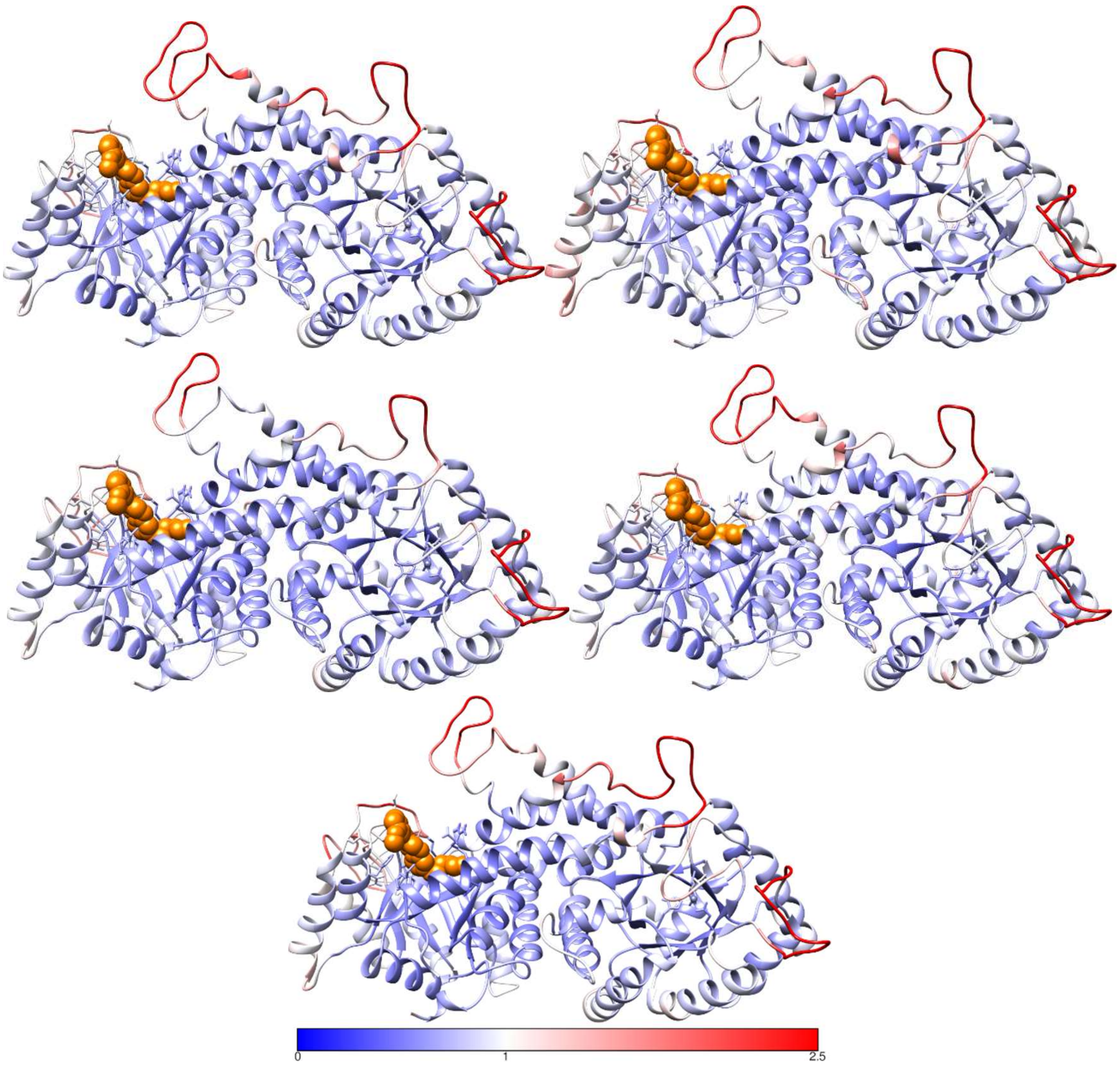

Figure S102: RMSFs of the protein in the complex of compound **21** with FBA1. One figure is provided per MD replica. The compound is represented as orange spheres.

Table S1. Docking scores for the selected complexes.

Target	Compound	Pose	PLP	Z_PLP	GS	Z_GS	CS	Z_CS	ASP	Z_ASP	Aggregated Score
LTP1	1	1	49.89	2.25	24.68	0.42	18.94	1.01	25.61	0.66	1
	2	1	49.24	1.6	33	1.41	22.25	2.68	25.01	0.79	1.62
		2	47.37	1.11	26.81	0.89	19.27	1.4	27.56	1.63	1.26
	3	1	66.63	1.32	10.98	0.19	24.34	1.58	38.48	1.76	1.21
		2	64.33	0.96	21.71	1.19	21.77	0.84	36.17	1.28	1.07
CAR1	1	1	34.36	1.98	23.01	1.35	15.83	2.33	18.53	1.37	1.76
		2	35.24	2.38	21.54	1.08	12.67	0.54	18.55	1.38	1.34
	2	1	32.43	1.78	20.94	2.02	12.4	1.54	15.53	2.79	2.03
	3	1	48.58	1.15	26.69	1.74	19.28	2.13	18.61	1.17	1.55
		2	52.34	2.2	0.22	-0.68	17.14	1.12	19.28	1.43	1.02
BMH1	1	1	37.02	1.41	27.98	1.76	13.12	0.25	22.81	1.49	1.23
		2	37.07	1.43	23.88	0.97	14.75	1.18	19.66	0.5	1.02
	2	1	36.83	0.99	29.71	2.3	13.75	0.49	21.73	1.26	1.26
		2	37.82	1.23	25.99	1.62	15.67	1.38	19.65	0.32	1.14
	3	1	57.97	2.05	28.16	1.33	20.5	1.18	27.04	0.59	1.29
		2	53.22	1.05	24.31	0.85	18.83	0.54	32.46	1.81	1.06
ARA1	1	1	27.69	2.1	10.44	2.18	15.68	2.25	24.64	1.78	2.08
		2	28.7	2.3	-4.91	1.25	17.12	2.55	23.01	1.56	1.92
		3	29.45	2.45	-9.77	0.96	13.52	1.8	25.1	1.84	1.76
		4	22.46	1.09	4.77	1.83	13.12	1.71	14.96	0.48	1.28
	2	1	25.28	2.84	-0.59	3.32	10.63	2.33	19.09	1.67	2.54
	3	1	58.54	3.43	-12.84	1.48	19.32	2.18	19.45	0.98	2.02
		2	40.1	0.53	6.01	2.46	18.03	1.86	21.97	1.44	1.57
		3	45.31	1.35	6.88	2.5	14.16	0.92	17.03	0.53	1.33

		1	45.89	2.07	22.3	1.24	21.48	2.38	33.68	1.71	1.85
	1	2	44.82	1.84	21.49	1.07	19.77	1.71	35.01	2.03	1.66
		3	46.79	2.26	19.36	0.6	18.89	1.37	32	1.32	1.39
		1	47.09	1.8	23.46	1.19	19.32	1.62	37.86	2.56	1.79
	2	2	47.22	1.83	25.63	1.63	16.85	0.68	30.99	0.66	1.2
GRE3		3	45.17	1.42	18.48	0.2	18.37	1.26	34.54	1.64	1.13
		4	41.28	0.65	20.12	0.52	20.51	2.08	31.64	0.84	1.02
		1	73.55	1.58	38.45	2.19	29.83	1.61	46.92	1.71	1.77
	3	2	77.57	2.08	26.28	0.98	31.65	2.08	47.94	1.85	1.75
		3	68.02	0.88	35.24	1.87	28.58	1.29	42.98	1.17	1.3
		4	72.4	1.43	27.65	1.12	27.89	1.11	44.25	1.34	1.25
	1	1	30.85	1.68	13.33	1.05	13.07	1.58	15.79	2.12	1.6
		2	30.73	1.62	11.49	0.5	12.54	1.37	14.66	1.67	1.29
CS	2	1	33.63	2.54	3.17	-1.5	11.72	2.13	13.25	2.03	1.3
	3	1	57.01	2.1	6.26	0.99	20.7	1.62	22.04	1.45	1.54
		2	57.95	2.33	8.29	1.13	17.17	0.93	19.91	1.09	1.37
	1	1	32.11	1.08	25.03	1.64	11.53	1.39	17.98	2.37	1.62
		2	33.84	1.78	23.34	1.39	10.72	1.06	16.18	1.77	1.5
		1	34.75	1.97	21.23	1.49	13.92	2.77	12.25	0.62	1.71
	2	2	35.4	2.27	12.37	0.11	8.14	0.17	16.78	2.65	1.3
CS1		3	34.09	1.66	17.22	0.86	13.31	2.5	11.05	0.08	1.28
		1	55.12	1.95	10.45	0.97	11.56	0.69	25.68	1.63	1.31
	3	2	50.95	1.28	20.96	1.34	14.87	1.23	20.85	1.03	1.22
		3	49.45	1.04	11.94	1.03	16.26	1.46	20.93	1.04	1.14
		4	49.02	0.97	-1.96	0.55	16.12	1.43	23.76	1.39	1.08
	1	1	35.52	1.61	17.71	0.89	15.36	1.12	16.84	0.75	1.09
ORD1	2	1	34.66	1.45	18.65	1.36	13.17	1.52	19.77	2.25	1.65
		2	36.96	1.94	17.21	1.08	9.75	-0.1	19.58	2.2	1.28

		3	35.82	1.7	23.72	2.38	10.48	0.24	14.47	0.71	1.26
		4	34.91	1.51	15.91	0.82	11.84	0.89	15.87	1.12	1.08
	3	1	52.21	2.59	4.59	0.43	18.49	1.41	21.86	1.88	1.58
		2	40.99	0.53	13.26	1.27	19.84	1.61	19.59	1.5	1.22
CSY4	1	1	35.4	2.23	26.16	2.48	8.2	0.17	19.04	1.99	1.72
	2	1	36.3	2.8	16.08	0.8	11.83	1.67	17.21	1.95	1.8
		2	32.06	1.37	18.77	1.31	11.71	1.61	16.26	1.51	1.45
	3	1	57.7	2.05	21.08	0.29	15.52	1.34	23.37	1.33	1.25
APR1	1	1	38.02	3.03	16.09	0.43	18.47	1.56	22.56	1.13	1.53
		2	35.68	2.15	15.78	0.36	18.26	1.46	24.2	1.59	1.39
	2	1	32.73	0.51	7.67	-0.73	21.22	2.53	24.07	1.61	0.98
	3	1	57.45	1.18	23.82	1.33	29.8	2.23	28.57	0.73	1.37
		2	56.84	1	7.37	-0.36	28.41	1.79	31.87	1.67	1.02
GCY	1	1	46.59	2.8	15.26	0.31	18.04	0.87	30.42	1.43	1.35
		2	42.02	1.52	23.61	1.67	18.32	0.98	27.59	0.39	1.14
		3	37.16	0.16	20.29	1.13	20.7	1.92	29.53	1.1	1.08
	2	1	42.96	1.34	25.52	2.3	18.25	0.77	32.49	1.64	1.51
		2	45.57	2.19	15.35	0.71	17.21	0.39	29.92	0.87	1.04
		3	42.91	1.33	9.82	-0.15	20.16	1.47	32.09	1.52	1.04
		4	40.62	0.58	13.53	0.43	20.15	1.46	32.35	1.6	1.02
	3	1	67.17	1.1	19.52	1.06	30.13	1.08	39.64	0.94	1.04
GCY1	1	1	42.65	2.42	21.56	1.16	20.04	1.52	30.39	0.78	1.47
	2	1	44.8	2.46	24.97	1.82	19.29	1.76	28.66	0.2	1.56
		2	42.47	1.54	24.98	1.83	18.88	1.37	32.82	1.18	1.48
		3	43.58	1.98	21.33	0.9	18.83	1.32	29.95	0.51	1.18
	3	1	70.96	1.3	21.61	0.59	33.84	1.22	52.38	1.87	1.24
FBA1	1	1	31.53	1.54	15	1.81	16.69	2.06	20.42	2.8	2.06
		2	27.86	0.53	6.58	0.57	14.34	1.31	17.77	1.88	1.07

	2	1	29.48	1.38	6.88	0.56	16.46	2.9	18.83	2.56	1.85
		1	53.71	1.82	23.41	2.19	17.26	1.43	19.45	1.18	1.66
	3	2	47.89	0.42	14.68	1.5	16.19	1.16	21.77	1.89	1.24
		3	54.7	2.06	-0.56	0.29	16.4	1.21	19.1	1.07	1.16
	1	1	47.36	2.52	27.69	1.89	19.88	1.65	24.3	1.96	2
		2	46.11	2.08	24.21	1.18	20.17	1.84	20.97	0.42	1.38
FBA1 Allosteric	2	1	45.2	1.57	27.1	1.25	18.52	1.05	22.99	1.08	1.24
		2	48.12	2.68	24.12	0.83	18.61	1.12	20	-0.19	1.11
	3	1	68.07	2.33	26.73	1.67	26.32	0.89	29.44	1.53	1.6
		2	68.48	2.45	12.4	0.01	26.59	1.06	27.53	0.73	1.06
	1	1	33.94	0.94	24.36	1.3	17.92	1.84	18.37	1.94	1.51
		2	34.41	1.12	22	0.85	16.66	1.34	15.96	0.87	1.04
		1	35.73	2	21.53	0.86	16.03	2.01	14.42	1.04	1.48
CEK2	2	2	34.41	1.39	22.63	1.02	14.87	1.4	15.1	1.38	1.3
		3	33.51	0.97	24.96	1.36	13.63	0.76	15.05	1.35	1.11
		4	35.23	1.77	21.18	0.81	12.76	0.31	14.88	1.27	1.04
	3	1	56.72	1.17	24.85	1.12	21.61	1.17	27.37	1.51	1.24
		2	56.81	1.19	29.69	1.54	21.95	1.32	24.54	0.65	1.18
		1	45.97	3.62	19.37	0.61	22.89	2.77	25.69	2.96	2.49
	1	2	34.2	0.66	28.65	2.41	19.77	1.38	23.33	2.2	1.66
		3	41.19	2.42	22.41	1.2	17.21	0.23	17.94	0.48	1.08
CDK1	2	1	33.38	0.69	16.82	0.6	18.02	1.63	18.51	1.52	1.11
		2	31.74	0.13	20.78	1.17	15.99	0.71	20.41	2.33	1.09
	3	1	59.45	2.14	16.06	0.87	23.6	1.3	25.8	1.73	1.51
		2	56.01	1.25	13.04	0.58	24.62	1.72	26.05	1.81	1.34
		1	39.43	2.12	20.93	0.32	20.45	2.48	17.53	0.7	1.41
ERK1	1	2	39.2	2.05	21.2	0.38	19.45	1.98	18.64	1.22	1.41
		3	37.77	1.58	31.02	2.5	17.91	1.21	16.67	0.3	1.4

		4	37.6	1.53	23.99	0.98	16.42	0.47	18.75	1.27	1.06
	2	1	36.83	1.61	20.69	0.56	20.03	2.95	17.9	1.28	1.6
		2	38.37	2.17	18.03	0.09	17.82	1.87	17.52	1.12	1.31
		1	58.91	1.67	25.68	0.95	23.96	1.22	27.24	1.4	1.31
	3	2	59.11	1.72	28.61	1.25	21.66	0.37	26.93	1.3	1.16
		3	55.41	0.63	29.22	1.31	22.29	0.6	28.62	1.82	1.09
		1	40.13	1.51	27.14	1.09	19.23	1.84	18.29	1.34	1.45
		2	38.31	0.89	30.82	1.74	17.99	1.19	17.43	0.94	1.19
	1	3	39.52	1.31	22.92	0.35	20.02	2.26	17.01	0.74	1.17
		4	39.69	1.36	35.63	2.58	16.54	0.43	15.33	-0.04	1.08
		5	36.59	0.3	27.44	1.15	17.51	0.94	19.48	1.9	1.07
MKC1		1	40.34	2.34	28.8	1.77	17.44	2.03	16.48	1.67	1.95
	2	2	38.43	1.61	20.59	0.18	17.07	1.86	17.89	2.37	1.5
		3	38.07	1.47	26.09	1.25	16.4	1.55	13.33	0.11	1.09
		1	61.06	1.32	36.56	2.06	24.66	1.77	24.67	1.56	1.68
	3	2	63.56	1.93	16.22	0.49	21.75	1.02	22.77	1.06	1.13
		3	60.79	1.25	22.94	1.01	22.48	1.21	20.99	0.59	1.02
	1	1	39.18	1.88	25.61	0.88	21.37	2.27	22.44	2.26	1.82
		1	41.86	2.35	23.69	1.13	19.93	2.18	17.45	1.33	1.75
HOG1	2	2	38.02	0.78	28.67	2.12	17.6	0.8	16.9	0.97	1.17
		3	36.3	0.07	24.9	1.37	15.86	-0.22	19.89	2.93	1.04
	3	1	64.9	0.35	37.18	1.85	29.63	2.16	27.47	1.2	1.39
		2	65.73	0.62	37.52	1.89	27.69	1.35	25.35	0.41	1.07

Table S2. Results of MM-PBSA calculations.

Target	Compound	Conformer	MM-PBSA Component							ΔG binding
			VD WAALS	EEL	EPB	ENPOLAR	EDISPER	ΔG gas	ΔG solv	
LTP1	1	1	-17.98	-13.55	25.29	-15.31	23.11	-31.53	33.09	1.56
	2	1	-23.73	-10.74	24.78	-17.47	27.66	-34.47	34.97	0.50
		2	-23.20	-3.89	19.61	-16.55	25.84	-27.08	28.91	1.82
	3	1	-42.10	-14.43	43.21	-31.40	51.16	-56.52	62.97	6.44
		2	-39.31	-10.89	34.93	-27.96	47.02	-50.20	53.99	3.79
CAR1	1	1	-15.23	-26.04	37.08	-13.59	22.99	-41.27	46.47	5.21
		2	-17.99	-18.11	39.48	-14.46	25.89	-36.09	50.91	14.82
	2	1	-31.50	-9.59	30.12	-20.03	33.50	-41.09	43.59	2.50
	3	1	-48.75	-17.45	44.28	-34.17	57.87	-66.20	67.99	1.79
		2	-45.35	-12.86	40.21	-34.46	57.75	-58.21	63.49	5.29
BMH1	1	1	-18.81	-8.81	26.28	-14.60	24.28	-27.62	35.96	8.34
		2	-15.68	-22.56	34.84	-14.88	24.17	-38.23	44.13	5.90
	2	1	-23.13	-14.17	32.78	-17.09	29.88	-37.30	45.57	8.27
		2	-20.48	-11.39	27.75	-15.70	28.64	-31.87	40.69	8.82
	3	1	-33.30	-27.37	48.07	-26.57	47.67	-60.67	69.16	8.49
		2	-38.42	-17.11	49.80	-30.97	54.59	-55.52	73.42	17.90
ARA1	1	1	-12.98	-5.61	17.20	-11.86	20.44	-18.58	25.78	7.20
		2	-16.92	-25.64	42.06	-15.36	24.48	-42.56	51.18	8.61
		3	-18.56	-26.94	40.45	-16.83	26.02	-45.50	49.64	4.15
		4	-19.05	-27.14	39.90	-16.94	25.72	-46.19	48.69	2.50
	2	1	-26.02	-8.69	29.96	-19.21	29.11	-34.71	39.86	5.15
	3	1	-26.06	-8.12	21.49	-19.90	34.67	-34.18	36.27	2.09
		2	-37.97	-13.45	40.18	-27.50	48.63	-51.42	61.31	9.89
		3	-33.60	-6.27	37.05	-26.25	46.31	-39.87	57.10	17.24

GRE3	1	1	-17.13	-18.04	34.14	-15.78	26.65	-35.17	45.00	9.84
		2	-19.67	-32.63	42.32	-17.06	27.67	-52.31	52.93	0.62
		3	-19.67	-28.41	39.05	-16.91	26.66	-48.08	48.80	0.72
	2	1	-23.66	-11.14	29.63	-17.84	30.86	-34.81	42.65	7.84
		2	-24.91	-15.50	30.42	-19.39	31.24	-40.41	42.27	1.87
		3	-23.05	-12.79	29.51	-17.90	30.62	-35.84	42.23	6.39
		4	-19.08	-4.22	19.56	-15.38	26.33	-23.29	30.51	7.22
	3	1	-32.68	-9.25	34.60	-26.18	46.85	-41.92	55.27	13.35
		2	-37.73	-7.17	31.02	-28.08	44.70	-44.89	47.64	2.75
		3	-34.18	-10.20	33.88	-25.57	45.50	-44.38	53.81	9.44
		4	-35.37	-8.98	33.71	-26.64	46.80	-44.34	53.87	9.53
CS	1	1	-24.20	-21.24	33.47	-17.57	27.85	-45.44	43.75	-1.69
		2	-20.53	-11.61	29.56	-15.04	28.64	-32.14	43.16	11.01
	2	1	-25.92	-15.79	31.88	-17.78	32.14	-41.71	46.24	4.53
	3	1	-54.04	-16.06	48.47	-38.87	66.38	-70.10	75.97	5.87
		2	-49.87	-19.88	56.74	-35.08	62.54	-69.75	84.20	14.45
CS1	1	1	-22.49	-26.15	39.24	-17.84	30.09	-48.64	51.49	2.85
		2	-21.84	-28.35	40.52	-17.46	29.66	-50.19	52.73	2.54
	2	1	-25.56	-13.40	27.62	-19.45	33.42	-38.97	41.58	2.61
		2	-24.01	-17.77	31.10	-16.86	30.38	-41.78	44.62	2.84
		3	-28.61	-6.99	26.03	-19.74	32.24	-35.60	38.53	2.92
	3	1	-53.45	-10.48	46.11	-37.72	65.12	-63.93	73.52	9.58
		2	-46.20	-12.81	40.85	-32.84	56.82	-59.00	64.83	5.82
		3	-54.57	-12.78	46.52	-37.58	66.72	-67.35	75.66	8.32
		4	-50.90	-14.21	46.66	-36.56	62.82	-65.11	72.93	7.82
ORD1	1	1	-17.19	-25.07	34.63	-14.74	23.70	-42.26	43.60	1.33
	2	1	-30.70	-7.13	30.28	-20.20	34.04	-37.84	44.11	6.28
		2	-31.99	-10.86	28.32	-20.54	34.11	-42.85	41.89	-0.96

		3	-19.43	-6.20	23.80	-14.47	23.78	-25.63	33.10	7.47
		4	-21.34	3.76	31.78	-15.12	24.30	-17.59	40.96	23.38
	3	1	-31.51	-8.37	36.14	-24.73	41.58	-39.88	52.99	13.11
		2	-28.55	-2.38	25.04	-22.51	40.85	-30.92	43.38	12.46
CSY4	1	1	-26.13	-27.27	43.70	-18.53	29.49	-53.40	54.66	1.26
	2	1	-28.86	-20.20	41.41	-20.14	34.17	-49.06	55.45	6.38
		2	-30.20	-27.04	43.29	-20.58	34.49	-57.24	57.19	-0.05
	3	1	-56.45	-16.38	54.44	-36.80	59.98	-72.83	77.62	4.79
APR1	1	1	-9.77	-6.70	12.54	-8.85	16.28	-16.47	19.97	3.51
		2	-22.90	-9.72	21.85	-17.52	28.11	-32.62	32.44	-0.18
	2	1	-16.46	-11.40	26.27	-14.32	25.78	-27.85	37.73	9.87
	3	1	-32.94	-10.12	28.30	-27.42	50.12	-43.06	51.00	7.94
		2	-37.36	-14.92	41.51	-27.72	52.79	-52.29	66.58	14.29
GCY	1	1	-23.83	-10.16	24.89	-17.18	26.53	-33.99	34.24	0.25
		2	-19.32	-19.29	31.86	-15.72	26.14	-38.60	42.27	3.67
		3	-15.65	-6.67	21.26	-13.17	23.16	-22.33	31.25	8.93
	2	1	-21.26	-11.92	29.13	-16.71	28.47	-33.18	40.89	7.71
		2	-20.13	-12.18	29.22	-15.94	27.22	-32.31	40.51	8.20
		3	-23.88	-12.08	27.43	-17.74	29.04	-35.95	38.73	2.77
		4	-21.84	-7.47	18.56	-16.96	26.60	-29.32	28.21	-1.11
	3	1	-34.15	-6.30	27.40	-27.56	45.07	-40.45	44.91	4.46
GCY1	1	1	-18.98	-14.34	26.70	-15.19	24.23	-33.31	35.75	2.43
	2	1	-20.22	-3.54	13.94	-16.56	24.53	-23.76	21.91	-1.86
		2	-22.43	-14.50	28.20	-16.49	27.29	-36.93	38.99	2.06
		3	-20.59	-7.34	23.32	-15.94	26.94	-27.93	34.33	6.40
	3	1	-36.82	-12.60	36.15	-28.29	48.03	-49.42	55.89	6.47
FBA1	1	1	-24.89	-3.55	21.83	-17.96	29.38	-28.44	33.26	4.83
		2	-20.63	-9.92	25.80	-14.41	25.90	-30.55	37.28	6.74

	2	1	-29.62	-2.29	16.47	-20.23	33.46	-31.91	29.69	-2.22
	3	1	-44.05	-12.94	42.38	-31.09	53.28	-56.99	64.57	7.58
		2	-29.91	-10.59	36.14	-23.14	41.34	-40.50	54.34	13.83
		3	-38.79	-12.15	41.02	-28.04	48.57	-50.94	61.55	10.61
FBA1 Allosteric	1	1	-24.72	-14.71	28.42	-18.32	29.02	-39.43	39.11	-0.32
		2	-22.83	-3.69	20.22	-17.32	28.13	-26.52	31.03	4.51
	2	1	-30.40	-1.83	16.10	-20.15	33.67	-32.22	29.62	-2.61
		2	-29.18	-5.95	18.83	-19.93	33.66	-35.14	32.56	-2.57
	3	1	-46.57	-8.03	30.98	-31.18	53.11	-54.59	52.91	-1.69
		2	-45.27	1.17	25.00	-32.37	54.43	-44.10	47.05	2.95
CEK2	1	1	-23.60	-14.55	29.92	-17.72	29.53	-38.15	41.73	3.57
		2	-19.63	-25.69	35.95	-17.51	28.73	-45.31	47.17	1.86
	2	1	-23.02	-8.76	22.03	-17.03	30.36	-31.78	35.36	3.58
		2	-28.20	-10.59	30.45	-20.15	33.23	-38.79	43.53	4.74
		3	-25.64	-9.62	26.94	-19.56	31.80	-35.26	39.18	3.92
		4	-24.61	-7.89	25.13	-18.60	31.61	-32.50	38.14	5.63
	3	1	-48.48	-10.48	45.07	-34.89	60.27	-58.96	70.45	11.50
		2	-46.69	-17.49	54.85	-35.67	58.89	-64.18	78.06	13.88
CDK1	1	1	-22.75	-15.87	31.40	-18.34	31.08	-38.62	44.14	5.52
		2	-21.86	-21.82	36.09	-17.86	30.20	-43.68	48.43	4.75
		3	-18.82	-20.80	33.29	-16.29	28.64	-39.62	45.65	6.03
	2	1	-26.15	-8.37	23.30	-20.17	32.66	-34.52	35.79	1.27
		2	-22.78	-18.24	34.88	-18.23	31.72	-41.01	48.37	7.35
	3	1	-37.83	-20.59	44.34	-28.32	48.87	-58.42	64.89	6.47
		2	-42.24	-20.34	43.20	-30.81	50.85	-62.58	63.24	0.66
ERK1	1	1	-22.01	-8.63	22.87	-16.72	28.85	-30.64	35.00	4.37
		2	-22.50	-16.29	33.66	-17.86	29.77	-38.79	45.57	6.78
		3	-18.42	-11.14	21.89	-14.86	26.60	-29.55	33.63	4.08

		4	-23.14	-16.99	32.46	-18.17	29.59	-40.13	43.88	3.75
	2	1	-23.59	-6.36	19.85	-18.14	31.33	-29.94	33.04	3.10
		2	-23.88	-5.57	20.53	-18.16	31.60	-29.45	33.97	4.52
		1	-52.05	-2.76	36.70	-35.84	62.30	-54.81	63.16	8.35
	3	2	-46.56	-5.16	34.82	-34.72	58.89	-51.71	59.00	7.28
		3	-48.17	-4.68	34.33	-34.90	61.69	-52.85	61.12	8.27
		1	-23.33	-21.65	34.37	-18.43	29.91	-44.98	45.85	0.87
		2	-21.10	-24.07	44.17	-18.25	29.30	-45.17	55.23	10.06
	1	3	-23.22	-16.18	33.82	-18.04	28.10	-39.40	43.88	4.48
		4	-20.68	-16.72	33.21	-16.89	27.14	-37.40	43.46	6.06
		5	-15.80	-20.70	35.72	-13.45	26.44	-36.50	48.71	12.20
MKC1		1	-29.76	-4.54	22.72	-20.98	32.70	-34.30	34.44	0.14
	2	2	-26.41	-0.84	29.45	-18.49	32.66	-27.25	43.62	16.36
		3	-24.29	-7.17	32.67	-17.36	31.03	-31.47	46.34	14.88
		1	-49.60	-6.03	39.26	-37.11	61.76	-55.62	63.91	8.29
	3	2	-46.01	-12.34	42.76	-34.68	59.67	-58.35	67.75	9.40
		3	-43.71	-8.97	40.30	-31.02	55.47	-52.68	64.75	12.07
	1	1	-20.97	-16.53	27.41	-17.41	28.91	-37.51	38.91	1.40
		1	-25.39	-15.69	28.54	-19.61	32.91	-41.08	41.84	0.75
HOG1	2	2	-28.81	-6.89	22.08	-20.43	32.94	-35.70	34.59	-1.11
		3	-30.89	-15.90	29.14	-20.80	34.20	-46.78	42.55	-4.23
	3	1	-53.05	-12.04	35.46	-38.03	61.85	-65.10	59.28	-5.82
		2	-50.93	-10.57	32.85	-36.87	59.81	-61.50	55.79	-5.71

References

[1] Koch, K.; Biggers, M. S. General Preparation of 7-Substituted 4-Chromanones: Synthesis of a Potent Aldose Reductase Inhibitor. *J Org Chem* **1994**, *59* (5), 1216–1218. https://doi.org/10.1021/jo00084a050.

[2] Foroumadi, A.; Samzadeh-Kermani, A.; Emami, S.; Dehghan, G.; Sorkhi, M.; Arabsorkhi, F.; Heidari, M. R.; Abdollahi, M.; Shafiee, A. Synthesis and Antioxidant Properties of Substituted 3-Benzylidene-7-Alkoxychroman-4-Ones. *Bioorg Med Chem Lett* **2007**, *17* (24), 6764–6769. https://doi.org/10.1016/j.bmcl.2007.10.034.

[3] Pifferi, G.; Da Re, P.; Valenti, P.; Bisi, A. Synthesis of the 3-Homologue of Ipriflavone. *Il Farmaco (Pavia)* **1996**, *51* (10), 689–691.

[4] Cloete, S. J.; N'Da, C. I.; Legoabe, L. J.; Petzer, A.; Petzer, J. P. The Evaluation of 1-Tetralone and 4-Chromanone Derivatives as Inhibitors of Monoamine Oxidase. *Mol Divers* **2021**, *25* (1), 491–507. https://doi.org/10.1007/s11030-020-10143-w.

[5] Takao, K.; Yamashita, M.; Yashiro, A.; Sugita, Y. Synthesis and Biological Evaluation of 3-Benzylidene-4-Chromanone Derivatives as Free Radical Scavengers and α-Glucosidase Inhibitors. *Chem Pharm Bull (Tokyo)* **2016**, *64* (8), 1203–1207. https://doi.org/10.1248/cpb.c16-00327.
